# Supplementary material for: FF12MC: A revised AMBER forcefield and new protein simulation protocol
Source: Proteins. 2016 Jul 21;84(10):1490–516. doi: 10.1002/prot.25094 (PMC5129589; doi:10.1002/prot.25094)
Supplement: Supplementary file 2 — Supporting Information [file PROT-84-1490-s002.pdf]

Supporting Equations, Tables, and Note

FF12MC: A revised AMBER forcefield and new protein simulation protocol

Yuan-Ping Pang

Computer-Aided Molecular Design Laboratory, Mayo Clinic, Rochester, MN 55905, USA

Equations. ***Page* 2.**

Table S1. Numbers of TIP3P waters and ions, initial solvation box size, protonation state of ionizable residue, and computers used in molecular dynamics simulations of peptides, miniproteins, and folded globular proteins. ***Page* 3.**

Table S2. The 30 unique seed numbers for initial velocities of Simulations 1–30. ***Page* 3.**

Table S3. Conformational cluster analysis of molecular dynamics simulations of AAQAA, chignolin, CLN025, and Trp-Cage (TC10b). ***Page* 4.**

Table S4. Experimental and calculated Lipari-Szabo order parameters of backbone N–H bonds in folded globular proteins. ***Pages* 5–9.**

Table S5. Experimental and calculated crystallographic B-factors of folded globular proteins. ***Pages* 10–19.**

Table S6. Conformational cluster analysis of molecular dynamics simulations of TMR01, TMR04, and TMR07. ***Pages* 20–28.**

Table S7. Effect of timestep size on the quality of 20 or 30 distinct and independent isobaric–isothermal molecular dynamics simulations. ***Pages* 29–31.**

Table S8. Experimental and calculated *J*-coupling constants of Ala3, Ala5, Ala7, and Val3 in water at 300 K. ***Pages* 31–36.**

Table S9. Different parameter sets of the Karplus Eqs. S1–S5 and S9–S20. ***Page* 37.**

Table S10. Effects of the van der Waals interaction cutoff and simulation time on mean square deviation (χ^2) between experimental and calculated *J*-coupling constants of Val3. ***Page* 38.**

Table S11. Experimental and calculated *J*-coupling constants of GB3, BPTI, ubiquitin, and lysozyme in water at 298–309 K. ***Pages* 39–54.**

Table S12. Torsion cluster analysis of C14–C38 in the simulations of BPTI and its mutant using FF12MC and FF14SBlm. ***Page* 55.**

Table S13. Root mean square deviations and standard errors between experimental and calculated Lipari-Szabo order parameters of backbone N–H bonds in GB3, BPTI, ubiquitin, and lysozyme and the experimentally determined overall tumbling correlation times of the four proteins. ***Page* 56.**

Table S14. Root mean square deviations and standard errors between experimental and calculated crystallographic Cα and Cγ B-factors of GB3, BPTI, ubiquitin, and lysozyme. ***Page* 57.**

Note S1. The organizers' assessment of the TMR01 refinement in the first CASPR experiment in 2006. ***Pages* 58–59.**

References. ***Page* 60.**

Equations S1–S21

$^3J(\mathrm{H_N},\mathrm{H}\alpha) = 7.09\cos^2(\phi_i - 60°) - 1.42\cos(\phi_i - 60°) + 1.55$ [S1]

$^3J(\mathrm{H_N},\mathrm{C'}) = 4.29\cos^2(\phi_i + 180°) - 1.01\cos(\phi_i + 180°)$ [S2]

$^3J(\mathrm{H}\alpha,\mathrm{C'}) = 3.72\cos^2(\phi_i + 120°) - 2.18\cos(\phi_i + 120°) + 1.28$ [S3]

$^3J(\mathrm{C'},\mathrm{C'}) = 1.36\cos^2(\phi_i) - 0.93\cos(\phi_i) + 0.60$ [S4]

$^3J(\mathrm{H_N},\mathrm{C}\beta) = 3.06\cos^2(\phi_i + 60°) - 0.74\cos(\phi_i + 60°) + 0.13$ [S5]

$^1J(\mathrm{N},\mathrm{C}\alpha) = 1.7040\cos^2(\psi_i) - 0.9799\cos(\psi_i) + 9.5098$ [S6]

$^2J(\mathrm{N},\mathrm{C}\alpha) = -0.6616\cos^2(\psi_{i-1}) - 1.5176\cos(\psi_{i-1}) + 7.8509$ [S7]

$^3J(\mathrm{H_N},\mathrm{C}\alpha) = -0.2280\cos(\phi_i) - 0.2026\cos(\psi_{i-1}) + 0.0680\sin(\phi_i) + 0.0802\sin(\psi_{i-1}) +$

$0.0664\cos(\phi_i)\cos(\psi_{i-1}) + 0.1209\cos(\phi_i)\sin(\psi_{i-1}) - 0.0798\sin(\phi_i)\cos(\psi_{i-1}) -$

$0.1367\sin(\phi_i)\sin(\psi_{i-1}) + 0.5390$ [S8]

$^3J(\mathrm{H}\alpha,\mathrm{H}\beta) = 3.47\cos^2(\chi_i) - 1.61\cos(\chi_i) + 4.33$ [S9]

$^3J(\mathrm{N'},\mathrm{H}\beta) = 2.21\cos^2(\chi_i) - 0.33\cos(\chi_i) + 1.05$ [S10]

$^3J(\mathrm{C'},\mathrm{H}\beta) = 2.81\cos^2(\chi_i) - 0.97\cos(\chi_i) + 1.60$ [S11]

$^3J(\mathrm{H}\alpha,\mathrm{C}\gamma) = 2.70\cos^2(\chi_i) - 0.79\cos(\chi_i) + 1.77$ [S12]

$^3J(\mathrm{N'},\mathrm{C}\gamma) = 0.86\cos^2(\chi_i) - 0.41\cos(\chi_i) + 0.59$ [S13]

$^3J(\mathrm{C'},\mathrm{C}\gamma) = 1.25\cos^2(\chi_i) - 0.74\cos(\chi_i) + 1.36$ [S14]

$^3J(\mathrm{N},\mathrm{C}\gamma,\mathrm{Thr}) = 2.12\cos^2(\chi_i + 13°) + 0.23\cos(\chi_i + 13°) - 0.15$ [S15]

$^3J(\mathrm{C'},\mathrm{C}\gamma,\mathrm{Thr}) = 2.97\cos^2(\chi_i + 25°) - 0.83\cos(\chi_i + 25°) - 0.02$ [S16]

$^3J(\mathrm{N},\mathrm{C}\gamma1,\mathrm{Val/Ile}) = 2.22\cos^2(\chi_i + 3°) + 0.15\cos(\chi_i + 3°) - 0.06$ [S17]

$^3J(\mathrm{N},\mathrm{C}\gamma2,\mathrm{Val/Ile}) = 2.24\cos^2(\chi_i - 9°) + 0.15\cos(\chi_i - 9°) - 0.03$ [S18]

$^3J(\mathrm{C'},\mathrm{C}\gamma1,\mathrm{Val/Ile}) = 3.31\cos^2(\chi_i + 16°) - 0.91\cos(\chi_i + 16°) + 0.01$ [S19]

$^3J(\mathrm{C'},\mathrm{C}\gamma2,\mathrm{Val/Ile}) = 3.30\cos^2(\chi_i + 4°) - 0.51\cos(\chi_i + 4°) + 0.04$ [S20]

$\chi^2 = \mathrm{N}^{-1}\sum_{i=1}^{\mathrm{N}}(<J_i>_{\mathrm{md}} - J_{i,\mathrm{nmr}})^2\sigma_i^{-2}$ [S21]

Table S1. Numbers of TIP3P waters and ions, initial solvation box size, protonation state of ionizable residue, and computers used in molecular dynamics simulations.

Sequence	# of H_2O	# of Na^+	# of Cl^-	Box size ($Å^3$)	Expt pH	Ionizable Residue Protonation status	Computers used for simulations
Ala3	573	0	0	37x29x28	2.0	ALC	Xserve
Ala5	682	0	0	41x28x29	2.0	ALC	Xserve
Ala7	800	0	0	51x28x27	2.0	ALC	Xserve
Val3	615	0	0	35x31x29	2.0	VAC	Xserve
Chignolin	1281	5	3	33x34x53	5.5	ASP,GLU	MacPros & Xserve
CLN025	1532	6	4	35x37x55	5.7	ASP,GLU	MacPros & Xserve
AAQAA	1619	0	0	78x33x30	7.0	–	MacPros & Xserve
Trp-cage	2512	1	1	77x41x35	7.0	ARG,ASP,LYS	MacPros
TMR01	4470	0	3	60x50x66	6.5	ARG,ASP,GLU,LYS	MacPros
TMR04	3504	0	4	63x49x52	9.2	ARG,ASP,GLU,LYS,HIE	MacPros
TMR07	4044	0	0	66x52x54	4.2	ARG,ASP,GLU,LYS,HID	MacPros
GB3	2528	2	0	45x57x47	5.8	ASP,GLU,LYS	MacPros
BPTI (5PTI)	3067	0	6	49x47x62	4.6	ARG,ASP,GLU,LYS	MacPros
BPTI (1QLQ)	3423	0	6	50x47x65	7.5	ARG,ASP,GLU,LYS	MacPros
BPTI (1PIT)	3108	0	6	47x46x65	4.6	ARG,ASP,GLU,LYS	MacPros
Ubiquitin	3881	0	1	50x66x53	4.7	ARG,ASP,GLU,LYS,HIP	MacPros
Lysozyme	5849	0	12	60x61x69	3.8	ARG,ASP,ASH^{101},GLH,LYS,HIP	MacPros

ALC: Ala with both amino and carboxylate groups protonated. VAC: Val with both amino and carboxylate groups protonated. Xserve: a cluster of Apple Xserves with 400 G5 processors (2.2/2.4 GHz). MacPros: a cluster of 100 12-core Apple Mac Pros with Intel Westmere (2.40/2.93 GHz).

Table S2. The 30 unique seed numbers for initial velocities of Simulations 1–30.

Simulation ID	Seed number	Simulation ID	Seed number	Simulation ID	Seed number
1	1804289383	11	1025202362	21	35005211
2	846930886	12	1350490027	22	521595368
3	1681692777	13	783368690	23	294702567
4	1714636915	14	1102520059	24	1726956429
5	1957747793	15	2044897763	25	336465782
6	424238335	16	1967513926	26	861021530
7	719885386	17	1365180540	27	278722862
8	1649760492	18	1540383426	28	233665123
9	596516649	19	304089172	29	2145174067
10	1189641421	20	1303455736	30	468703135

Table S3. Conformational cluster analysis of molecular dynamics simulations of AAQAA, chignolin, CLN025, and Trp-Cage (TC10b).

Forcefield Temperature	IDs of the simulations that captured a folding event	Total number of frames	Number of clusters identified	Top-3 largest cluster ID	Number of frames in the cluster	Occurrence of the cluster (%)	CRMSD (Å)or helix info
			Chignolin				
FF12MC 277 K	1–20	20000[a]	466	5	9788	48.9	1.62
				37	4690	23.4	3.27
				4	512	2.6	3.65
FF12MC 300 K	1–20	20000[a]	842	63	6679	33.4	1.63
				38	4253	21.3	3.33
				39	738	3.7	3.64
FF12SB 277 K	6,9,13,14,18	5000[a]	437	127	913	18.3	3.32
				144	710	14.2	1.54
				128	319	6.4	3.98
FF14SB 277 K	2,4,6–8,10, 12–15	10000[a]	569	301	3878	38.8	1.33
				401	991	9.9	3.19
				484	370	3.7	5.05
			CLN025				
FF12MC 277 K	1–20	20000[a]	564	28	14092	70.5	1.72
				7	894	4.5	4.82
				10	621	3.1	4.80
FF12MC 300 K	1–20	20000[a]	700	26	13072	65.4	1.69
				13	1352	6.8	4.65
				12	818	4.1	4.81
FF12SB 277 K	5,8,11,13	4000[a]	256	95	1043	26.1	1.76
				93	360	9.0	4.79
				84	317	7.9	4.77
FF14SB 277 K	1,5–8,10,16,19	8000[a]	524	103	1396	17.4	1.70
				101	524	6.6	4.63
				95	262	3.3	5.78
			AAQAA				
FF12MC 274K	1–20	20000[a]	2833	72	8349	41.7	full α
				71	1591	8.0	π + α
				68	698	3.5	partial α
FF12MC 300K	1–20	20000[a]	7275	13	3770	18.9	full α
				46	1154	5.8	π + α
				12	598	3.0	partial α
FF12MC 310K	1–20	20000[a]	9090	93	2872	14.4	full α
				176	1127	5.6	π + α
				191	428	2.1	partial α
			Trp-cage				
FF12MC 280 K	1–30	21000[b]	4417	20	5214	24.8	1.53
				21	2926	13.9	1.85
				32	1952	9.3	5.13

[a] All conformations were obtained at every 10^6 timesteps of N one-billion–time-step molecular dynamics simulations as described in Methods and Table S1, wherein N is the number of simulations each of which has an aggregated native state population of >1.5%. [b] All conformations were obtained at every 4×10^6 timesteps of 30 2.8-billion–time-step molecular dynamics simulations as described in Methods and Table S1.

Table S4A. Experimental and calculated Lipari-Szabo order parameters of backbone N–H bonds in GB3 at 297 K.

Residue	Experimental	FF12MCsm		FF14SB	
ID	symmetric[1]	mean	SE	mean	SE
3	0.83207	0.845	0.004	0.850	0.014
4	0.86468	0.903	0.002	0.908	0.002
5	0.87703	0.899	0.002	0.920	0.002
6	0.87195	0.904	0.002	0.912	0.001
7	0.82951	0.901	0.003	0.910	0.002
8	0.85378	0.908	0.002	0.913	0.002
9	0.83390	0.890	0.004	0.878	0.012
10	0.80560	0.864	0.008	0.897	0.004
11	0.76550	0.854	0.009	0.843	0.007
12	0.66389	0.837	0.018	0.843	0.009
13	0.76469	0.843	0.006	0.857	0.007
14	0.73548	0.744	0.020	0.755	0.032
16	0.83245	0.852	0.007	0.905	0.002
17	0.80067	0.844	0.003	0.876	0.003
18	0.84683	0.878	0.007	0.914	0.001
19	0.77430	0.860	0.004	0.876	0.003
20	0.75592	0.844	0.008	0.857	0.028
21	0.83688	0.885	0.004	0.906	0.002
22	0.86523	0.829	0.012	0.887	0.003
23	0.91565	0.897	0.002	0.915	0.001
24	0.81067	0.886	0.003	0.901	0.002
26	0.91109	0.917	0.002	0.919	0.001
28	0.89256	0.925	0.001	0.921	0.002
29	0.89660	0.912	0.002	0.910	0.002
30	0.88859	0.922	0.001	0.921	0.002
31	0.90887	0.929	0.001	0.932	0.001
32	0.88288	0.919	0.001	0.916	0.002
33	0.89936	0.920	0.002	0.923	0.002
34	0.91283	0.925	0.002	0.929	0.001
36	0.88759	0.911	0.002	0.915	0.002
37	0.83187	0.893	0.002	0.828	0.003
38	0.78800	0.789	0.010	0.878	0.003
39	0.84571	0.841	0.013	0.874	0.004
40	0.72839	0.860	0.008	0.876	0.010
41	0.49980	0.829	0.007	0.740	0.026
42	0.83328	0.878	0.004	0.874	0.004
43	0.85602	0.881	0.004	0.897	0.002
44	0.86163	0.848	0.007	0.910	0.002
45	0.81883	0.816	0.010	0.899	0.002
46	0.85252	0.862	0.003	0.870	0.003
47	0.81357	0.887	0.002	0.879	0.002
48	0.75027	0.890	0.003	0.888	0.002
49	0.82074	0.897	0.003	0.835	0.005
50	0.88485	0.841	0.005	0.890	0.002
51	0.86070	0.900	0.002	0.901	0.002
52	0.87057	0.910	0.001	0.922	0.002
53	0.84356	0.878	0.005	0.928	0.001
54	0.89230	0.900	0.003	0.916	0.002
55	0.82414	0.898	0.003	0.919	0.001
56	0.85034	0.904	0.002	0.893	0.004

Table S4B. Experimental and calculated Lipari-Szabo order parameters of backbone N–H bonds in BPTI at 298 K.

Residue ID	Experimental	FF12MCsm		FF14SB	
	Beeser et al.[2]	mean	SE	mean	SE
3	0.82850	0.863	0.003	0.873	0.003
4	0.87620	0.870	0.005	0.899	0.002
5	0.87450	0.879	0.004	0.910	0.002
6	0.88130	0.810	0.021	0.899	0.003
7	0.80920	0.774	0.031	0.843	0.018
10	0.82990	0.825	0.004	0.870	0.005
11	0.87430	0.885	0.002	0.898	0.002
12	0.83340	0.880	0.003	0.906	0.002
14	0.85880	0.822	0.008	0.877	0.005
15	0.75480	0.843	0.005	0.890	0.002
16	0.83280	0.723	0.031	0.810	0.022
17	0.73830	0.807	0.028	0.878	0.004
18	0.77710	0.851	0.006	0.854	0.010
19	0.83430	0.866	0.004	0.878	0.002
20	0.82020	0.872	0.004	0.905	0.001
21	0.86810	0.894	0.002	0.908	0.002
22	0.89320	0.911	0.002	0.928	0.001
23	0.81280	0.914	0.001	0.927	0.001
24	0.85440	0.909	0.002	0.907	0.002
25	0.77840	0.866	0.004	0.880	0.003
26	0.71690	0.858	0.007	0.857	0.004
27	0.72160	0.872	0.003	0.822	0.013
28	0.85430	0.835	0.004	0.875	0.002
29	0.81310	0.874	0.004	0.891	0.002
30	0.77390	0.879	0.003	0.893	0.002
31	0.83810	0.880	0.004	0.887	0.008
32	0.80350	0.868	0.003	0.883	0.003
33	0.88530	0.892	0.003	0.922	0.001
34	0.83000	0.864	0.003	0.880	0.003
35	0.88210	0.875	0.005	0.903	0.004
36	0.90930	0.870	0.005	0.909	0.001
38	0.88920	0.682	0.023	0.878	0.002
39	0.85020	0.874	0.004	0.914	0.001
40	0.78060	0.809	0.012	0.877	0.002
41	0.81920	0.818	0.011	0.868	0.005
42	0.85560	0.753	0.039	0.887	0.004
43	0.84770	0.902	0.002	0.910	0.003
44	0.81140	0.900	0.002	0.918	0.001
45	0.78510	0.892	0.002	0.896	0.004
46	0.84520	0.885	0.004	0.916	0.002
47	0.81540	0.796	0.006	0.847	0.007
48	0.79970	0.898	0.002	0.916	0.002
49	0.82740	0.900	0.002	0.912	0.002
50	0.89270	0.904	0.004	0.917	0.002
51	0.82690	0.917	0.002	0.920	0.001
52	0.91920	0.920	0.001	0.928	0.001
53	0.86500	0.906	0.002	0.918	0.002
54	0.85710	0.870	0.015	0.888	0.003
55	0.88330	0.890	0.006	0.912	0.001
56	0.87510	0.814	0.019	0.872	0.006

Table S4C. Experimental and calculated Lipari-Szabo order parameters of backbone N–H bonds in ubiquitin at 300 K.

Residue ID	Experimental	FF12MCsm		FF14SB	
	aniso[3]	mean	SE	mean	SE
2	0.837	0.852	0.007	0.881	0.006

3	0.883	0.883	0.005	0.909	0.002
4	0.898	0.893	0.002	0.911	0.002
5	0.830	0.856	0.007	0.925	0.001
6	0.860	0.882	0.006	0.901	0.003
7	0.859	0.877	0.005	0.899	0.004
8	0.772	0.843	0.016	0.891	0.003
9	0.731	0.829	0.015	0.836	0.009
10	0.736	0.661	0.042	0.792	0.014
11	0.709	0.697	0.036	0.780	0.018
12	0.756	0.821	0.013	0.859	0.004
13	0.845	0.838	0.006	0.886	0.004
14	0.830	0.840	0.006	0.887	0.002
15	0.822	0.843	0.006	0.907	0.003
16	0.777	0.857	0.005	0.868	0.005
17	0.876	0.867	0.009	0.894	0.007
18	0.847	0.850	0.012	0.883	0.014
20	0.842	0.848	0.007	0.895	0.002
21	0.900	0.837	0.010	0.912	0.003
22	0.852	0.876	0.005	0.900	0.002
26	0.858	0.917	0.001	0.925	0.001
27	0.917	0.932	0.001	0.933	0.001
28	0.897	0.920	0.001	0.920	0.002
29	0.890	0.918	0.001	0.924	0.001
30	0.887	0.923	0.001	0.916	0.001
32	0.895	0.909	0.002	0.910	0.002
33	0.855	0.886	0.002	0.823	0.009
34	0.851	0.881	0.004	0.858	0.004
35	0.855	0.699	0.022	0.896	0.002
36	0.783	0.568	0.040	0.823	0.007
39	0.852	0.885	0.004	0.895	0.002
40	0.865	0.858	0.006	0.861	0.006
41	0.853	0.850	0.006	0.859	0.005
42	0.832	0.883	0.004	0.857	0.009
43	0.828	0.869	0.005	0.856	0.006
44	0.838	0.896	0.002	0.906	0.003
45	0.872	0.901	0.003	0.926	0.001
46	0.840	0.847	0.008	0.893	0.002
47	0.821	0.817	0.009	0.853	0.006
48	0.843	0.744	0.017	0.835	0.006
49	0.752	0.864	0.006	0.879	0.003
50	0.836	0.866	0.005	0.884	0.003
51	0.796	0.848	0.008	0.848	0.006
52	0.788	0.816	0.024	0.876	0.006
54	0.856	0.723	0.015	0.829	0.013
55	0.868	0.854	0.006	0.903	0.002
56	0.902	0.914	0.002	0.922	0.002
57	0.871	0.896	0.004	0.907	0.002
58	0.892	0.887	0.003	0.906	0.002
59	0.866	0.889	0.004	0.863	0.004
60	0.884	0.816	0.006	0.888	0.002
61	0.857	0.848	0.008	0.896	0.003
62	0.703	0.832	0.005	0.853	0.004
63	0.817	0.856	0.004	0.906	0.002
64	0.878	0.865	0.005	0.918	0.003
65	0.871	0.719	0.024	0.866	0.003
66	0.841	0.859	0.003	0.884	0.002
67	0.843	0.836	0.007	0.894	0.003
68	0.873	0.827	0.006	0.878	0.005
69	0.847	0.853	0.005	0.871	0.004
70	0.910	0.890	0.003	0.887	0.004
71	0.796	0.840	0.007	0.875	0.004
73	0.565	0.792	0.011	0.863	0.005

Table S4D. Experimental and calculated Lipari-Szabo order parameters of backbone N–H bonds in lysozyme at 308 K.

Residue	Experimental	FF12MCsm		FF14SB	
ID	Buck et al.[4]	mean	SE	mean	SE
2	0.830	0.837	0.005	0.849	0.003
3	0.830	0.811	0.009	0.861	0.004
4	0.830	0.834	0.005	0.878	0.002
5	0.850	0.871	0.003	0.892	0.002
6	0.860	0.903	0.002	0.914	0.001
7	0.880	0.898	0.002	0.907	0.002
8	0.890	0.918	0.001	0.918	0.001
9	0.930	0.919	0.001	0.926	0.001
10	0.890	0.923	0.002	0.926	0.001
11	0.890	0.916	0.001	0.919	0.001
12	0.910	0.923	0.001	0.927	0.001
13	0.920	0.925	0.001	0.925	0.001
14	0.820	0.918	0.001	0.925	0.001
15	0.840	0.879	0.013	0.782	0.015
17	0.890	0.783	0.015	0.799	0.022
18	0.860	0.709	0.033	0.844	0.006
19	0.840	0.809	0.013	0.834	0.003
20	0.850	0.782	0.013	0.881	0.003
21	0.890	0.884	0.003	0.908	0.001
22	0.990	0.823	0.012	0.861	0.004
23	0.880	0.704	0.012	0.837	0.007
24	0.890	0.876	0.008	0.892	0.002
25	0.870	0.904	0.003	0.921	0.002
26	0.910	0.913	0.002	0.912	0.002
27	0.940	0.904	0.002	0.922	0.001
28	0.870	0.903	0.003	0.909	0.002
29	0.900	0.922	0.001	0.918	0.001
31	0.930	0.928	0.001	0.927	0.001
32	0.940	0.927	0.001	0.920	0.002
33	0.910	0.912	0.001	0.905	0.001
34	0.920	0.901	0.002	0.891	0.002
35	0.880	0.872	0.003	0.848	0.009
36	0.860	0.861	0.007	0.883	0.003
37	0.960	0.867	0.003	0.899	0.002
38	0.900	0.889	0.003	0.909	0.001
39	0.890	0.822	0.006	0.880	0.002
40	0.910	0.901	0.003	0.901	0.002
41	0.860	0.849	0.006	0.889	0.006
42	0.870	0.696	0.020	0.835	0.008
43	0.830	0.845	0.008	0.892	0.002
44	0.830	0.803	0.010	0.872	0.015
45	0.780	0.848	0.005	0.854	0.007
46	0.830	0.798	0.009	0.811	0.009
47	0.780	0.848	0.005	0.869	0.003
48	0.770	0.850	0.006	0.818	0.008
49	0.820	0.831	0.012	0.852	0.007
51	0.890	0.846	0.007	0.895	0.005
52	0.890	0.908	0.002	0.918	0.001
53	0.870	0.912	0.002	0.915	0.002
54	0.910	0.908	0.002	0.917	0.001
55	0.940	0.913	0.002	0.927	0.001
56	0.920	0.909	0.001	0.924	0.001
57	0.940	0.836	0.007	0.909	0.002
58	0.900	0.877	0.005	0.880	0.002
59	0.910	0.905	0.002	0.914	0.001
60	0.930	0.909	0.002	0.919	0.001
61	0.950	0.865	0.010	0.916	0.001
62	0.850	0.848	0.012	0.858	0.007
63	0.900	0.893	0.003	0.898	0.002
64	0.910	0.898	0.003	0.873	0.002

65	0.860	0.889	0.002	0.913	0.002
66	0.890	0.876	0.007	0.894	0.003
67	0.850	0.785	0.010	0.878	0.003
68	0.780	0.557	0.040	0.830	0.013
69	0.760	0.867	0.004	0.867	0.012
71	0.720	0.785	0.012	0.832	0.005
72	0.760	0.718	0.022	0.838	0.003
73	0.880	0.765	0.017	0.774	0.008
74	0.870	0.879	0.004	0.906	0.002
75	0.940	0.889	0.011	0.927	0.001
76	0.920	0.893	0.003	0.902	0.003
77	0.900	0.877	0.004	0.910	0.002
78	0.910	0.879	0.007	0.838	0.039
80	0.910	0.899	0.002	0.910	0.002
81	0.860	0.906	0.002	0.907	0.002
82	0.880	0.873	0.005	0.910	0.002
83	0.830	0.894	0.003	0.893	0.003
84	0.830	0.881	0.007	0.865	0.004
85	0.550	0.706	0.040	0.829	0.010
86	0.800	0.835	0.008	0.861	0.005
87	0.800	0.817	0.019	0.778	0.019
88	0.800	0.867	0.007	0.873	0.004
89	0.920	0.876	0.005	0.916	0.001
90	0.910	0.911	0.002	0.920	0.002
91	0.850	0.911	0.001	0.907	0.001
92	0.930	0.923	0.001	0.917	0.001
93	0.930	0.923	0.001	0.924	0.001
94	0.920	0.922	0.001	0.918	0.002
95	0.920	0.927	0.001	0.925	0.001
96	0.920	0.925	0.001	0.925	0.001
97	0.940	0.915	0.002	0.911	0.002
98	0.920	0.921	0.002	0.925	0.001
100	0.890	0.894	0.005	0.904	0.002
101	0.850	0.878	0.006	0.881	0.003
102	0.720	0.687	0.039	0.739	0.008
103	0.820	0.646	0.043	0.670	0.013
104	0.810	0.771	0.018	0.835	0.004
105	0.880	0.803	0.016	0.866	0.003
106	0.960	0.900	0.003	0.906	0.002
107	0.910	0.883	0.004	0.903	0.002
108	0.840	0.853	0.006	0.850	0.004
109	0.850	0.884	0.003	0.896	0.003
111	0.840	0.890	0.002	0.892	0.001
112	0.890	0.920	0.002	0.923	0.002
113	0.890	0.886	0.003	0.895	0.003
114	0.870	0.853	0.005	0.828	0.008
115	0.790	0.779	0.011	0.811	0.007
116	0.840	0.872	0.005	0.885	0.007
117	0.810	0.733	0.023	0.822	0.004
118	0.720	0.521	0.023	0.676	0.012
119	0.800	0.799	0.030	0.883	0.002
120	0.800	0.773	0.009	0.839	0.009
121	0.910	0.881	0.006	0.904	0.002
122	0.920	0.895	0.004	0.918	0.001
123	0.900	0.882	0.003	0.899	0.002
124	0.900	0.884	0.003	0.895	0.003
125	0.870	0.809	0.014	0.873	0.002
126	0.820	0.765	0.016	0.840	0.009
127	0.770	0.642	0.037	0.743	0.018
128	0.760	0.800	0.011	0.831	0.006
129	0.600	0.702	0.016	0.582	0.017

Table S5A. Experimental and calculated crystallographic Cα B-factors of GB3 at 297 K.

Residue	Experimental	FF12MCsm		FF14SB	
ID	PDB ID: 1IGD	mean	SE	mean	SE
6	9.37	9.0	0.7	7.1	0.4
7	7.26	5.9	0.4	4.5	0.2
8	5.67	2.6	0.1	1.9	0.0
9	5.25	3.1	0.1	2.4	0.1
10	4.92	3.1	0.1	2.6	0.1
11	4.74	3.1	0.3	2.5	0.1
12	6.55	3.9	0.4	2.8	0.1
13	11.08	4.3	0.2	3.9	0.2
14	10.70	6.1	0.3	5.3	0.2
15	13.87	10.6	0.9	8.3	0.7
16	10.83	16.1	1.6	12.2	1.7
17	7.46	9.7	0.7	6.5	0.4
18	6.02	6.9	0.6	4.9	0.2
19	5.14	10.8	1.3	6.8	0.4
20	4.57	6.2	0.5	4.6	0.4
21	5.79	5.3	0.4	4.6	0.3
22	5.73	5.0	0.3	3.3	0.2
23	6.63	6.4	0.3	4.4	0.4
24	8.78	7.5	0.4	5.4	0.5
25	8.12	7.0	0.4	5.1	0.3
26	9.17	10.6	0.8	8.4	0.5
27	8.29	7.2	0.4	5.2	0.4
28	7.92	5.8	0.3	4.3	0.3
29	8.29	5.6	0.3	5.7	0.3
30	8.62	5.1	0.4	4.7	0.3
31	7.76	3.5	0.2	3.1	0.1
32	7.49	3.7	0.1	3.5	0.1
33	8.72	4.9	0.3	4.7	0.2
34	8.52	4.4	0.2	3.9	0.1
35	5.83	3.1	0.1	2.4	0.1
36	8.04	4.0	0.2	3.4	0.2
37	8.49	5.3	0.2	4.4	0.2
38	6.97	5.0	0.3	4.1	0.2
39	7.52	4.5	0.2	3.9	0.2
40	10.58	6.2	0.4	5.6	0.4
41	10.70	7.9	0.7	6.5	0.3
42	8.19	8.0	0.6	6.0	0.5
43	12.66	8.6	0.5	7.7	0.8
44	10.80	4.8	0.3	3.8	0.2
45	13.78	6.3	0.3	7.0	0.9
46	10.92	8.6	0.5	6.8	0.6
47	6.35	5.0	0.3	4.5	0.3
48	5.23	3.9	0.2	3.8	0.2
49	5.24	4.2	0.2	3.2	0.1
50	5.26	4.0	0.3	3.0	0.1
51	6.92	4.3	0.2	3.7	0.1
52	8.34	6.8	0.4	5.6	0.2
53	14.44	9.7	0.7	8.3	0.4
54	8.53	6.8	0.5	5.7	0.2
55	7.33	3.8	0.4	3.1	0.1
56	5.13	3.1	0.2	2.5	0.1
57	4.37	2.6	0.1	2.0	0.1
58	5.03	3.2	0.1	2.3	0.1
59	5.94	3.4	0.2	2.7	0.1
60	6.83	3.7	0.2	3.2	0.2
61	9.61	5.4	0.3	5.2	0.3

Table S5B. Experimental and calculated crystallographic Cγ B-factors of GB3 at 297 K.

Residue ID	Experimental	FF12MCsm		FF14SB	
	PDB ID: 1IGD	mean	SE	mean	SE
6	15.33	20.0	3.5	10.6	0.4
7	13.69	17.9	2.7	9.6	0.3
8	7.01	3.4	0.1	2.5	0.0
9	8.78	13.0	1.7	4.9	0.2
10	6.19	5.3	0.2	4.4	0.1
11	8.60	11.0	1.9	6.1	0.2
12	12.84	13.4	2.4	6.4	0.3
13	17.08	12.0	1.0	9.3	0.6
15	34.94	19.5	1.4	16.2	1.4
16	16.96	46.2	5.1	28.0	3.9
17	8.14	21.1	1.8	12.3	0.7
18	14.80	20.0	1.6	9.6	0.8
20	8.47	16.8	1.7	10.4	1.6
21	8.14	14.2	1.3	8.4	0.4
22	9.24	16.0	1.6	6.9	0.2
23	10.26	14.1	1.2	7.7	0.5
24	28.78	23.6	1.4	15.1	1.9
26	11.75	29.1	3.7	17.1	1.1
27	10.37	10.7	0.9	8.0	0.7
29	39.72	14.6	1.4	10.4	0.6
30	10.79	12.3	0.8	9.1	0.4
32	8.71	9.4	0.5	7.8	0.3
33	20.51	15.9	2.1	10.1	0.9
35	5.99	4.1	0.2	2.8	0.1
36	18.85	11.7	1.0	7.0	0.6
37	17.37	18.5	1.9	9.5	0.5
38	6.92	7.3	0.5	6.3	0.3
40	42.35	14.6	1.5	14.0	1.6
41	11.80	16.6	1.4	11.8	0.6
42	11.29	15.5	1.4	10.3	0.8
44	11.81	13.7	1.4	9.8	0.7
45	44.17	16.1	1.0	20.5	3.2
47	10.98	17.9	1.8	10.8	1.4
48	6.51	5.1	0.2	5.1	0.2
49	6.81	15.9	2.1	7.8	0.3
50	6.65	5.4	0.3	3.9	0.1
51	16.64	8.7	0.4	8.7	0.3
52	25.01	16.3	1.3	15.9	1.3
54	16.48	13.3	0.8	11.8	0.5
55	11.18	9.5	1.3	6.0	0.2
56	8.87	7.5	0.4	5.4	0.1
57	5.42	4.1	0.1	3.2	0.1
58	6.18	9.8	1.3	5.7	0.1
59	8.12	7.2	0.3	5.4	0.1
60	10.01	11.7	1.2	7.4	0.3
61	12.85	8.0	0.8	6.0	0.4

Table S5C. Experimental and calculated crystallographic Cα B-factors of BPTI at 297 K.

Residue ID	Experimental	FF12MCsm		FF14SB	
	PDB ID: 4PTI	mean	SE	mean	SE
1	17.63	16.4	1.3	11.9	1.7
2	9.21	11.8	1.3	7.9	0.7
3	10.58	11.6	1.1	7.6	0.9
4	17.01	7.6	0.8	5.6	0.5
5	9.61	6.3	0.9	3.8	0.2
6	16.06	5.9	0.4	4.0	0.2
7	13.10	5.2	0.2	4.3	0.3

8	12.90	5.8	0.2	4.8	0.2
9	9.50	5.3	0.2	4.0	0.1
10	10.93	4.2	0.1	3.2	0.1
11	10.34	4.2	0.3	2.9	0.1
12	8.45	7.7	0.3	5.2	0.1
13	16.32	12.9	0.7	8.7	0.6
14	12.04	8.3	0.5	4.8	0.2
15	19.55	12.9	0.9	7.5	0.4
16	9.98	9.1	0.4	6.4	0.3
17	10.09	8.2	0.5	6.5	0.5
18	13.44	5.5	0.3	4.1	0.2
19	7.64	4.8	0.2	3.9	0.1
20	7.78	3.4	0.2	2.5	0.1
21	12.57	3.0	0.1	2.5	0.1
22	4.10	3.1	0.1	2.4	0.1
23	4.91	2.6	0.1	2.0	0.0
24	9.31	4.5	0.3	3.7	0.2
25	15.09	11.9	1.0	9.9	0.8
26	14.48	17.9	1.5	11.9	0.8
27	11.73	14.0	0.9	8.7	0.5
28	8.56	9.6	0.6	8.0	0.4
29	10.25	5.4	0.3	4.6	0.3
30	7.89	3.8	0.2	2.8	0.1
31	9.28	4.2	0.2	2.9	0.1
32	10.49	4.7	0.2	3.4	0.1
33	10.15	4.1	0.2	3.0	0.1
34	7.47	4.8	0.2	3.3	0.1
35	8.02	4.7	0.5	2.9	0.1
36	8.05	5.2	0.3	3.2	0.1
37	7.09	9.9	0.5	5.0	0.1
38	6.52	8.9	0.5	4.7	0.2
39	11.96	10.5	0.8	6.7	0.5
40	15.30	8.8	0.7	5.3	0.2
41	12.46	5.3	0.3	3.8	0.1
42	7.00	4.9	0.2	3.8	0.2
43	6.83	2.9	0.1	2.3	0.1
44	10.77	2.9	0.1	2.3	0.0
45	5.68	3.1	0.1	2.3	0.1
46	8.58	6.1	0.4	4.3	0.2
47	7.49	8.6	0.7	5.0	0.2
48	6.49	7.2	0.3	4.9	0.2
49	6.16	7.2	0.5	5.5	0.2
50	10.77	5.7	0.4	4.0	0.3
51	12.87	3.1	0.1	2.3	0.1
52	8.29	4.9	0.3	3.5	0.2
53	11.60	7.2	0.5	4.9	0.3
54	9.55	7.1	0.4	3.8	0.2
55	10.08	6.4	0.5	3.2	0.1
56	16.38	14.8	3.1	9.1	0.7
57	28.53	26.6	3.9	22.7	1.9
58	45.05	53.8	6.6	51.6	6.8

Table S5D. Experimental and calculated crystallographic Cγ B-factors of BPTI at 297 K.

Residue ID	Experimental	FF12MCsm		FF14SB	
	PDB ID: 4PTI	mean	SE	mean	SE
1	13.42	20.0	2.5	10.5	1.0
2	11.42	17.1	1.5	11.8	0.9
3	34.90	21.4	2.9	16.5	2.0
4	13.77	9.7	1.2	6.5	0.5
6	20.58	17.2	1.2	8.7	0.5
7	29.71	10.3	1.1	7.3	0.6
8	27.88	10.8	0.5	12.6	0.6

9	10.65	7.7	0.3	6.6	0.2
10	9.25	6.1	0.4	4.9	0.2
11	23.47	11.0	1.5	5.6	0.2
13	15.31	20.0	1.3	13.6	0.7
15	32.27	34.6	1.8	17.5	1.2
17	9.73	19.6	2.2	17.4	2.1
18	11.08	12.0	1.1	7.5	0.4
19	14.39	16.5	2.0	9.8	0.3
20	11.46	5.1	0.5	2.8	0.2
21	5.50	5.1	0.3	5.0	0.2
22	10.98	4.4	0.2	3.2	0.1
23	6.41	4.7	0.3	3.4	0.1
24	22.83	9.6	0.7	6.6	0.3
26	26.23	46.7	4.4	24.5	2.4
29	14.01	17.1	1.1	14.4	0.9
31	17.66	13.6	1.3	5.7	0.2
32	14.64	13.2	0.5	8.1	0.2
33	6.68	4.5	0.2	3.1	0.1
34	15.81	18.9	2.1	10.6	0.4
35	10.18	5.6	0.5	3.7	0.1
39	12.04	24.0	1.9	14.5	1.2
41	14.28	11.1	0.7	6.5	0.6
42	21.62	9.5	0.8	6.9	0.3
43	7.77	3.0	0.1	2.4	0.1
44	7.36	4.8	0.8	2.8	0.1
45	10.01	4.1	0.2	2.7	0.1
46	22.12	20.3	1.6	10.6	1.7
49	5.98	20.5	1.6	11.4	0.4
50	31.36	9.4	0.9	7.5	0.7
52	9.44	20.7	2.3	7.7	0.5
53	29.13	16.6	1.7	10.1	1.0
54	12.27	13.3	0.8	7.0	0.3

Table S5E. Experimental and calculated crystallographic Cα B-factors of ubiquitin at 297 K.

Residue	Experimental	FF12MCsm		FF14SB	
ID	PDB ID: 1UBQ	mean	SE	mean	SE
1	10.38	9.1	0.8	4.9	0.3
2	9.07	5.9	0.3	3.7	0.2
3	5.07	5.0	0.3	2.8	0.1
4	4.68	4.2	0.2	2.7	0.1
5	3.87	3.5	0.2	2.6	0.1
6	6.12	4.3	0.3	3.0	0.1
7	7.48	6.2	0.5	5.0	0.3
8	14.15	10.7	1.0	9.7	0.8
9	19.24	20.9	2.3	13.1	0.7
10	18.74	22.2	4.3	10.0	0.5
11	11.91	9.9	0.8	6.6	0.6
12	9.85	6.9	0.5	5.1	0.2
13	11.84	6.6	0.5	4.5	0.3
14	9.63	5.6	0.4	3.9	0.3
15	9.03	4.3	0.3	3.2	0.2
16	11.50	5.4	0.5	3.6	0.2
17	8.85	5.0	0.3	3.5	0.2
18	7.08	6.8	0.4	4.2	0.3
19	7.07	9.7	0.9	4.7	0.2
20	6.28	8.2	0.7	4.2	0.2
21	7.70	3.9	0.2	3.0	0.2
22	6.01	3.9	0.3	3.1	0.2
23	9.92	3.8	0.3	2.9	0.1
24	11.81	5.1	0.4	3.7	0.2
25	10.96	4.4	0.3	3.6	0.2
26	5.53	3.2	0.1	3.1	0.1

27	4.14	3.1	0.2	2.7	0.1
28	7.74	3.6	0.2	3.1	0.1
29	7.90	3.5	0.2	3.2	0.2
30	5.58	3.7	0.2	3.0	0.1
31	8.67	4.7	0.4	3.6	0.2
32	14.01	6.4	0.4	5.3	0.3
33	14.00	8.3	0.7	5.5	0.3
34	10.07	9.3	0.9	5.9	0.4
35	6.29	9.5	0.9	6.3	0.4
36	6.07	7.5	0.8	5.3	0.3
37	9.18	8.2	0.8	5.4	0.4
38	9.08	7.0	0.9	4.7	0.3
39	14.96	8.7	0.8	6.2	0.5
40	10.76	5.9	0.4	4.4	0.3
41	3.87	3.9	0.2	3.4	0.2
42	6.97	4.4	0.3	3.6	0.1
43	3.51	4.1	0.2	3.0	0.1
44	5.55	4.1	0.1	3.7	0.1
45	4.70	4.6	0.2	4.4	0.2
46	7.15	16.3	1.5	12.6	1.0
47	11.68	17.1	1.5	13.2	0.7
48	8.82	9.0	0.5	7.7	0.5
49	7.18	5.8	0.3	5.2	0.3
50	7.41	4.9	0.2	4.4	0.2
51	11.90	5.8	0.3	4.9	0.3
52	16.56	5.3	0.2	4.6	0.2
53	11.77	6.8	0.3	6.2	0.3
54	9.05	4.4	0.2	3.4	0.2
55	9.03	4.4	0.1	3.2	0.1
56	8.29	4.4	0.2	3.2	0.1
57	9.00	6.9	0.3	4.6	0.1
58	7.91	7.6	0.4	4.6	0.1
59	8.45	7.9	0.4	5.6	0.3
60	13.94	8.7	0.5	7.0	0.5
61	11.78	6.2	0.4	5.0	0.2
62	15.52	10.1	1.4	6.3	0.3
63	11.97	9.3	0.9	4.8	0.2
64	10.94	7.7	0.6	4.7	0.2
65	6.90	6.6	0.5	4.0	0.1
66	3.80	6.1	0.4	3.8	0.1
67	3.85	5.5	0.3	3.4	0.1
68	4.17	3.6	0.1	2.8	0.1
69	3.97	3.8	0.2	3.5	0.1
70	6.26	5.4	0.4	4.3	0.2
71	16.06	7.7	0.5	6.4	0.4
72	25.83	10.6	1.0	7.6	0.6
73	30.76	20.2	2.7	12.2	1.0
74	35.33	24.7	2.8	14.3	1.4
75	36.07	54.8	8.0	41.8	3.6
76	36.19	85.9	8.3	91.3	10.7

Table S5F. Experimental and calculated crystallographic Cγ B-factors of ubiquitin at 297 K.

Residue	Experimental	FF12MCsm		FF14SB	
ID	PDB ID: 1UBQ	mean	SE	mean	SE
1	16.29	15.8	1.1	7.1	0.4
2	17.01	16.0	1.4	8.5	0.5
3	5.58	10.7	0.5	6.4	0.2
4	7.97	7.4	0.4	4.6	0.2
5	9.13	8.5	0.2	6.4	0.2
6	11.12	14.1	1.8	8.3	1.3
7	9.17	16.3	1.5	9.7	0.6
8	18.88	26.1	4.1	16.1	2.0

9	19.70	48.2	5.7	26.3	1.6
11	16.69	26.2	2.0	10.5	0.8
12	9.63	13.6	0.7	10.3	0.4
13	17.08	15.9	1.5	10.2	0.7
14	11.66	19.1	1.8	12.2	0.7
15	15.79	8.0	1.0	7.9	1.1
16	23.33	19.9	1.7	9.6	0.8
17	10.54	11.8	1.1	7.4	0.6
18	12.65	17.7	1.5	10.6	0.9
19	8.16	16.2	1.1	11.1	0.7
21	15.32	6.1	0.4	4.9	0.3
22	9.65	13.0	0.9	6.8	0.3
23	10.90	7.9	0.3	6.3	0.2
24	27.76	15.0	1.9	8.4	0.8
25	22.31	7.3	0.6	7.1	0.6
26	8.12	8.1	0.2	7.0	0.3
27	7.45	6.9	0.4	3.6	0.2
29	14.94	9.3	1.5	4.9	0.5
30	2.78	6.7	0.5	4.7	0.1
31	10.76	8.8	0.7	5.6	0.2
32	24.33	15.8	1.7	11.3	1.1
33	24.00	17.9	1.4	6.9	0.6
34	18.75	17.7	1.8	9.7	0.6
36	7.36	16.7	3.2	7.7	0.7
37	9.27	13.2	1.3	9.3	0.6
38	10.81	12.9	1.4	11.9	1.0
39	31.06	14.8	1.3	9.7	0.8
40	14.85	15.4	1.6	7.6	0.7
41	3.20	4.7	0.7	3.2	0.1
42	21.27	15.2	1.8	6.5	0.4
43	6.32	9.8	0.6	6.4	0.6
44	7.39	15.4	1.9	8.4	0.2
45	5.98	7.2	0.4	7.7	0.6
48	14.14	25.7	2.3	19.9	1.8
49	15.82	16.2	1.7	8.8	0.5
50	7.53	7.3	0.5	5.1	0.2
51	26.06	19.7	2.1	9.9	0.8
52	25.12	9.3	0.7	8.1	0.5
54	9.62	14.6	1.1	8.9	0.7
55	11.71	8.8	0.2	6.3	0.2
56	7.73	8.6	0.7	5.4	0.4
58	11.50	14.9	1.5	6.2	0.3
59	6.91	6.6	0.3	5.4	0.3
60	22.65	19.6	1.5	16.4	1.1
61	13.29	12.5	0.8	9.3	0.4
62	26.38	20.4	1.6	14.6	1.0
63	16.98	19.2	1.9	7.6	0.3
64	24.16	18.0	1.8	11.2	1.3
66	3.40	16.1	0.8	8.6	0.3
67	9.67	13.9	1.1	6.0	0.3
68	9.95	9.1	0.6	6.6	0.3
69	7.37	7.0	0.5	5.4	0.2
70	8.54	18.7	1.9	9.1	0.3
71	19.37	18.0	1.9	15.1	2.4
72	31.79	20.1	2.1	13.1	1.6
73	30.16	58.2	7.3	33.4	2.8
74	38.62	39.0	3.9	21.5	2.8

Table S5G. Experimental and calculated crystallographic Cα B-factors of lysozyme at 295 K.

Residue ID	Experimental	FF12MCsm		FF14SB	
	PDB ID: 4LZT	mean	SE	mean	SE
1	11.32	13.7	1.8	7.6	0.4
2	8.52	8.7	0.5	5.7	0.3
3	7.86	6.0	0.4	4.7	0.2
4	8.74	8.4	0.5	6.2	0.3
5	9.46	6.7	0.3	4.9	0.2
6	8.44	5.5	0.4	4.0	0.1
7	7.83	5.7	0.4	4.5	0.2
8	6.84	4.2	0.2	3.1	0.1
9	7.71	4.3	0.2	3.0	0.1
10	8.79	4.7	0.2	3.6	0.1
11	8.45	5.2	0.3	4.0	0.1
12	7.70	4.7	0.2	3.1	0.1
13	9.64	5.9	0.3	3.7	0.1
14	10.42	7.5	0.4	5.3	0.2
15	11.72	9.1	0.8	5.5	0.3
16	13.62	10.3	0.8	6.9	0.3
17	9.59	7.4	0.5	4.9	0.2
18	9.89	7.8	0.4	5.9	0.4
19	8.60	7.1	0.3	6.2	0.3
20	8.45	7.2	0.5	5.4	0.3
21	9.39	9.6	0.8	7.0	0.4
22	9.97	12.0	1.0	9.5	0.6
23	8.44	6.6	0.3	5.8	0.3
24	9.19	6.1	0.3	4.6	0.3
25	8.38	4.3	0.2	3.1	0.1
26	8.78	4.5	0.2	3.2	0.1
27	7.49	3.9	0.2	2.6	0.1
28	6.96	4.3	0.2	3.1	0.1
29	6.86	3.4	0.2	2.6	0.1
30	7.58	3.2	0.1	2.5	0.1
31	7.56	4.3	0.2	3.4	0.1
32	6.66	4.6	0.2	3.3	0.1
33	7.18	4.4	0.2	3.3	0.1
34	7.91	4.5	0.2	3.6	0.1
35	6.84	4.2	0.3	3.1	0.1
36	8.04	5.4	0.2	3.7	0.1
37	9.17	7.5	0.6	4.0	0.1
38	7.56	5.6	0.4	3.2	0.1
39	8.13	5.4	0.2	3.7	0.2
40	7.32	5.0	0.3	3.9	0.2
41	8.55	6.5	0.5	4.4	0.2
42	7.95	6.4	0.3	4.3	0.1
43	8.35	6.4	0.5	4.2	0.1
44	8.61	7.0	0.6	5.3	0.3
45	8.37	8.8	0.7	7.5	0.6
46	9.47	10.9	0.8	8.4	0.5
47	10.18	23.2	2.1	15.4	0.8
48	12.81	16.5	1.2	12.1	0.7
49	14.58	12.9	0.9	10.4	0.5
50	7.85	5.9	0.4	4.5	0.2
51	6.11	3.6	0.2	2.7	0.1
52	5.99	3.2	0.2	2.6	0.1
53	5.88	2.9	0.1	2.4	0.1
54	6.70	4.1	0.1	3.2	0.1
55	6.60	3.9	0.2	2.8	0.1
56	6.46	4.6	0.3	3.4	0.1
57	5.69	4.0	0.3	2.7	0.1
58	6.08	3.7	0.2	2.7	0.1
59	6.66	3.6	0.2	2.9	0.1
60	6.45	3.4	0.2	2.6	0.1
61	6.87	4.6	0.2	4.0	0.2

62	8.61	5.6	0.4	4.7	0.2
63	5.54	3.7	0.2	3.0	0.1
64	7.21	4.0	0.2	3.2	0.1
65	8.33	6.0	0.4	4.0	0.2
66	7.86	6.4	0.4	5.3	0.3
67	12.96	14.0	1.3	10.9	0.7
68	14.52	11.7	0.6	9.9	0.5
69	8.91	8.5	0.4	7.7	0.4
70	11.54	14.6	1.2	15.8	1.1
71	15.83	18.4	2.0	17.5	1.4
72	10.25	10.8	1.1	8.1	0.4
73	7.63	8.7	0.9	6.6	0.4
74	6.19	6.3	0.6	4.3	0.2
75	6.16	6.4	0.6	4.8	0.2
76	5.84	7.0	0.6	4.3	0.2
77	6.35	9.0	0.8	5.6	0.3
78	7.13	7.7	0.6	4.9	0.2
79	7.37	6.6	0.4	4.4	0.2
80	6.23	4.1	0.2	3.0	0.1
81	7.42	6.8	0.4	5.1	0.2
82	7.49	8.8	0.7	5.3	0.3
83	7.35	6.8	0.7	3.8	0.1
84	8.22	6.4	0.5	4.4	0.2
85	13.32	9.2	0.7	5.8	0.3
86	13.52	9.1	0.8	6.4	0.3
87	12.57	6.7	0.5	5.7	0.3
88	8.57	4.6	0.4	4.2	0.2
89	10.16	5.2	0.5	3.7	0.2
90	8.21	5.4	0.4	4.3	0.2
91	6.50	4.5	0.2	3.5	0.1
92	7.88	4.5	0.2	3.5	0.2
93	8.16	4.6	0.2	3.7	0.2
94	6.27	3.8	0.2	3.0	0.1
95	6.93	4.4	0.2	3.8	0.1
96	8.22	4.7	0.2	4.1	0.2
97	10.17	4.5	0.2	4.5	0.3
98	9.90	4.7	0.3	4.2	0.2
99	14.59	5.5	0.4	4.6	0.2
100	19.08	7.7	0.6	6.3	0.4
101	16.35	9.0	0.7	7.9	0.4
102	26.69	14.3	1.2	12.9	0.7
103	18.77	12.1	1.5	7.6	0.4
104	17.38	12.1	1.3	10.6	0.9
105	9.91	7.8	0.5	6.4	0.7
106	16.70	9.9	0.9	6.9	0.6
107	13.93	11.1	1.2	8.7	1.0
108	9.30	6.7	0.6	5.5	0.6
109	10.67	9.6	1.1	7.2	0.6
110	10.52	9.3	1.0	7.1	0.5
111	9.74	6.9	0.5	4.8	0.3
112	11.51	8.0	0.6	5.7	0.3
113	12.38	9.9	0.8	8.7	0.6
114	13.70	7.6	0.5	6.4	0.3
115	10.90	5.5	0.3	4.1	0.2
116	12.73	9.3	0.7	6.9	0.4
117	28.58	23.5	1.9	17.0	1.0
118	18.53	14.9	1.2	8.1	0.4
119	22.82	12.8	0.9	8.2	0.4
120	16.41	7.7	0.4	5.5	0.2
121	17.92	9.0	0.6	5.9	0.3
122	17.95	9.2	0.7	5.6	0.2
123	15.88	8.9	0.6	6.0	0.3
124	13.70	9.6	0.7	6.1	0.3
125	17.70	11.3	0.8	7.4	0.4
126	24.68	16.1	1.2	11.1	0.6
127	17.40	12.0	1.0	6.9	0.3

128	24.46	22.1	1.8	13.3	0.8
129	31.79	22.4	2.0	13.1	0.9

Table S5H. Experimental and calculated crystallographic Cγ B-factors of lysozyme at 295 K.

Residue	Experimental	FF12MCsm		FF14SB	
ID	PDB ID: 4LZT	mean	SE	mean	SE
1	12.35	25.9	3.4	8.1	0.3
2	14.09	25.9	2.4	10.7	0.4
3	7.97	6.5	0.4	4.7	0.1
5	20.89	10.4	0.6	7.3	0.3
7	9.87	17.4	2.1	9.5	0.5
8	7.83	6.3	0.2	4.1	0.1
12	7.50	7.1	0.4	3.8	0.1
13	18.22	12.5	0.6	6.7	0.3
14	15.89	17.7	1.8	8.1	0.3
15	15.32	10.6	1.4	7.8	0.4
17	10.87	8.6	0.5	5.4	0.2
18	13.04	12.5	0.9	11.3	1.0
19	8.80	15.9	0.9	13.3	0.8
20	8.92	7.9	0.6	6.2	0.4
21	13.48	20.7	2.3	11.0	0.7
23	9.19	8.4	0.5	8.0	0.5
25	10.31	7.5	0.5	4.4	0.1
27	9.25	5.9	0.3	4.2	0.3
28	7.21	4.6	0.2	3.7	0.1
29	9.67	7.4	0.4	5.0	0.1
33	11.22	10.3	0.7	6.3	0.2
34	9.38	5.9	0.4	4.5	0.2
35	8.52	6.2	0.6	4.6	0.3
37	19.55	16.9	1.6	9.2	0.9
38	8.29	5.5	0.3	3.6	0.1
39	12.07	8.9	0.3	6.0	0.3
40	10.96	9.5	0.8	7.2	0.3
41	19.79	16.8	2.6	9.9	1.1
43	14.25	15.8	1.9	7.8	0.8
44	13.80	11.5	1.5	11.4	0.8
45	15.18	20.5	1.7	12.9	0.9
46	11.42	10.8	0.7	8.8	0.4
47	13.42	55.1	5.0	30.5	1.7
48	14.97	16.4	1.8	11.4	0.6
51	8.01	8.1	1.1	4.5	0.2
52	8.99	7.7	0.6	5.5	0.3
53	6.53	3.6	0.1	3.0	0.1
55	11.36	8.0	0.3	5.7	0.6
56	10.45	12.9	0.8	7.3	0.2
57	7.02	4.8	0.2	3.5	0.1
58	8.11	8.5	0.8	5.6	0.2
59	7.70	5.8	0.3	4.2	0.1
61	11.91	13.7	2.0	8.6	0.7
62	12.45	12.8	1.8	9.7	0.6
63	7.45	5.4	0.4	3.8	0.1
65	15.36	15.6	1.6	8.3	0.4
66	7.29	7.9	1.0	6.0	0.3
68	22.58	25.7	3.4	17.4	2.2
69	8.97	11.9	0.6	10.3	0.5
70	18.37	21.9	1.7	27.4	2.2
73	9.24	21.0	1.5	12.6	1.5
74	7.80	8.5	0.8	5.8	0.2
75	7.30	12.5	1.6	7.6	0.3
77	9.07	20.7	2.3	11.9	0.4
78	12.43	23.7	4.5	9.6	0.3
79	11.19	12.0	0.6	9.4	0.5

83	8.87	6.6	0.4	3.9	0.1
84	8.75	8.0	0.8	5.3	0.2
87	27.18	12.0	1.0	10.6	0.8
88	14.78	9.7	0.5	9.1	0.4
89	14.81	12.7	1.5	7.9	0.4
92	15.53	10.1	0.9	7.1	0.3
93	13.45	10.1	0.6	8.2	0.4
96	9.84	7.7	0.4	5.1	0.3
97	17.00	13.0	1.2	7.5	0.4
98	14.36	9.0	0.5	6.9	0.2
99	22.69	10.2	1.0	7.7	0.2
101	13.06	19.3	1.7	9.7	0.6
103	24.15	24.0	5.2	11.2	0.6
105	9.92	12.7	0.9	10.2	0.8
106	31.54	21.4	1.7	13.8	1.8
108	8.85	7.3	0.5	6.4	0.6
109	16.69	28.0	4.8	17.5	2.1
111	10.08	9.4	1.0	5.7	0.4
112	39.74	20.7	2.4	9.8	0.5
113	24.29	18.4	1.4	18.0	1.4
114	16.26	17.2	1.7	10.3	0.5
116	16.02	19.2	1.1	10.9	0.7
118	23.00	26.6	2.4	12.4	0.5
119	44.67	19.9	1.7	13.3	0.7
120	17.87	15.3	2.0	9.3	0.3
121	35.69	21.3	2.1	10.2	0.4
123	16.42	9.3	0.5	6.5	0.2
124	20.24	16.2	1.0	10.8	0.5
125	24.75	24.8	2.1	11.8	0.8
128	37.18	48.2	3.9	28.1	1.8
129	18.26	19.0	3.1	10.8	0.6

Table S6A. Conformational cluster analysis of molecular dynamics simulations of TMR01 using FF12MC at 340 K.

Number of timesteps (x10^6)	Number of frames	Number of clusters	Three largest cluster ID	Number of frames in cluster	Occurrence of the cluster (%)	CαRMSD (Å)	GDT-TS	GDT-HA	RPF9	LDDT15
	200	17	2	64	32.0	3.349	0.813	0.679	0.768	0.704
1	200	17	10	20	10.0	5.098	0.830	0.712	0.742	0.696
	200	17	1	17	8.5	4.078	0.846	0.736	0.749	0.700
	400	22	3	116	29.0	3.421	0.827	0.692	0.772	0.708
2	400	22	2	44	11.0	4.380	0.846	0.742	0.754	0.711
	400	22	1	36	9.0	3.134	0.863	0.742	0.795	0.739
	600	27	5	130	21.7	3.102	0.841	0.701	0.784	0.716
3	600	27	1	65	10.8	2.551	0.879	0.764	0.823	0.761
	600	27	2	52	8.7	4.182	0.852	0.747	0.757	0.713
	800	34	1	152	19.0	2.706	0.849	0.709	0.794	0.725
4	800	34	5	84	10.5	4.745	0.800	0.673	0.732	0.679
	800	34	2	82	10.2	2.277	0.887	0.764	0.827	0.767
	1000	37	7	153	15.3	2.541	0.849	0.717	0.792	0.728
5	1000	37	1	142	14.2	2.470	0.876	0.753	0.821	0.757
	1000	37	5	125	12.5	4.606	0.805	0.681	0.738	0.682
	1200	41	1	369	30.7	2.309	0.879	0.758	0.817	0.752
6	1200	41	6	130	10.8	4.654	0.800	0.673	0.733	0.677
	1200	41	12	84	7.0	4.598	0.783	0.657	0.734	0.668
	1400	44	0	250	17.9	2.439	0.879	0.761	0.823	0.758
7	1400	44	6	221	15.8	3.862	0.813	0.679	0.754	0.697
	1400	44	2	196	14.0	2.351	0.860	0.750	0.803	0.745
	1600	47	3	315	19.7	2.198	0.890	0.772	0.826	0.765
8	1600	47	7	284	17.7	4.035	0.816	0.690	0.753	0.701
	1600	47	2	207	12.9	2.187	0.863	0.750	0.806	0.749
	1800	45	2	371	20.6	2.106	0.896	0.772	0.827	0.768
9	1800	45	1	283	15.7	2.348	0.863	0.753	0.803	0.745
	1800	45	8	268	14.9	3.833	0.805	0.673	0.752	0.695
	2000	47	2	417	20.8	1.965	0.898	0.778	0.832	0.777
10	2000	47	1	399	20.0	2.409	0.868	0.758	0.804	0.743
	2000	47	8	240	12.0	4.498	0.800	0.668	0.736	0.681
	4000	66	2	1824	45.6	1.680	0.907	0.791	0.838	0.781
20	4000	66	17	267	6.7	4.191	0.800	0.668	0.755	0.688
	4000	66	10	198	5.0	4.733	0.797	0.676	0.732	0.679
	6000	94	1	2939	49.0	1.554	0.909	0.791	0.847	0.787
30	6000	94	22	289	4.8	3.981	0.791	0.659	0.752	0.682
	6000	94	0	251	4.2	3.555	0.835	0.695	0.770	0.716
	8000	112	1	4128	51.6	1.536	0.904	0.791	0.847	0.787
40	8000	112	28	417	5.2	3.363	0.830	0.698	0.775	0.711
	8000	112	36	319	4.0	4.132	0.786	0.651	0.751	0.680
	10000	135	1	5300	53.0	1.534	0.907	0.791	0.847	0.786
50	10000	135	43	397	4.0	4.177	0.800	0.668	0.754	0.682
	10000	135	51	362	3.6	4.664	0.728	0.571	0.690	0.621
	20000	202	1	11581	57.9	1.453	0.920	0.797	0.851	0.791
100	20000	202	50	890	4.4	4.849	0.695	0.536	0.680	0.613
	20000	202	40	775	3.9	4.441	0.720	0.555	0.693	0.627

Table S6B. Conformational cluster analysis of molecular dynamics simulations of TMR01 using FF14SBlm at 340 K.

Number of timesteps (x10^6)	Number of frames	Number of clusters	Three largest cluster ID	Number of frames in cluster	Occurrence of the cluster (%)	CαRMSD (Å)	GDT-TS	GDT-HA	RPF9	LDDT15
	200	6	0	148	74.0	4.364	0.827	0.695	0.738	0.706
1	200	6	1	24	12.0	5.885	0.778	0.637	0.714	0.662
	200	6	5	10	5.0	6.440	0.725	0.566	0.696	0.649
	400	5	0	262	65.5	4.247	0.830	0.695	0.744	0.712
2	400	5	1	81	20.3	5.611	0.797	0.670	0.724	0.679
	400	5	3	20	5.0	5.435	0.750	0.593	0.693	0.642
	600	8	0	364	60.7	4.257	0.832	0.701	0.746	0.712
3	600	8	2	95	15.8	5.490	0.772	0.635	0.712	0.666
	600	8	5	47	7.8	5.553	0.819	0.698	0.749	0.705
	800	7	0	471	58.9	4.338	0.827	0.692	0.746	0.711
4	800	7	2	165	20.6	5.564	0.800	0.670	0.732	0.684
	800	7	3	60	7.5	4.533	0.797	0.657	0.736	0.682
	1000	10	0	518	51.8	4.274	0.830	0.692	0.749	0.713
5	1000	10	2	175	17.5	5.697	0.775	0.640	0.712	0.668
	1000	10	5	95	9.5	5.337	0.819	0.698	0.754	0.709
	1200	11	0	641	53.4	4.326	0.827	0.692	0.749	0.712
6	1200	11	2	161	13.4	5.909	0.772	0.635	0.711	0.664
	1200	11	5	132	11.0	5.113	0.821	0.701	0.756	0.709
	1400	13	0	692	49.4	4.291	0.830	0.692	0.749	0.712
7	1400	13	2	217	15.5	4.880	0.830	0.712	0.764	0.717
	1400	13	3	165	11.8	6.013	0.772	0.637	0.712	0.667
	1600	11	0	747	46.7	4.241	0.827	0.687	0.750	0.712
8	1600	11	2	295	18.4	4.828	0.827	0.706	0.767	0.718
	1600	11	3	274	17.1	5.447	0.767	0.615	0.708	0.659
	1800	11	0	800	44.4	4.230	0.827	0.687	0.750	0.712
9	1800	11	2	395	21.9	4.814	0.832	0.709	0.771	0.721
	1800	11	3	281	15.6	5.426	0.761	0.607	0.708	0.659
	2000	13	0	821	41.0	4.192	0.827	0.687	0.751	0.713
10	2000	13	2	497	24.8	4.680	0.841	0.712	0.773	0.723
	2000	13	3	314	15.7	5.302	0.767	0.610	0.708	0.662
	4000	23	3	1512	37.8	4.074	0.838	0.695	0.776	0.727
20	4000	23	0	984	24.6	4.240	0.827	0.690	0.749	0.712
	4000	23	4	316	7.9	5.176	0.769	0.615	0.709	0.661
	6000	24	2	3176	52.9	3.668	0.841	0.701	0.782	0.732
30	6000	24	0	982	16.4	4.241	0.827	0.690	0.748	0.712
	6000	24	15	400	6.7	4.071	0.841	0.725	0.757	0.727
	8000	24	3	4816	60.2	3.543	0.843	0.703	0.787	0.738
40	8000	24	0	981	12.3	4.247	0.827	0.690	0.749	0.712
	8000	24	10	511	6.4	3.091	0.854	0.717	0.794	0.737
	10000	26	3	6738	67.4	3.371	0.843	0.706	0.792	0.741
50	10000	26	0	983	9.8	4.241	0.827	0.690	0.749	0.712
	10000	26	16	589	5.9	4.281	0.854	0.753	0.758	0.734
	20000	33	3	15161	75.8	3.014	0.849	0.717	0.805	0.750
100	20000	33	1	1084	5.4	3.983	0.854	0.750	0.764	0.731
	20000	33	0	977	4.9	4.238	0.827	0.690	0.749	0.712

Table S6C. Conformational cluster analysis of molecular dynamics simulations of TMR01 using FF96lm at 340 K.

Number of timesteps (x10^6)	Number of frames	Number of clusters	Three largest cluster ID	Number of frames in cluster	Occurrence of the cluster (%)	CαRMSD (Å)	GDT-TS	GDT-HA	RPF9	LDDT15
	200	15	3	73	36.5	4.476	0.810	0.670	0.724	0.678
1	200	15	7	19	9.5	5.382	0.786	0.626	0.720	0.655
	200	15	11	15	7.5	2.999	0.846	0.706	0.769	0.714
	400	24	3	123	30.7	4.467	0.824	0.684	0.737	0.690
2	400	24	7	37	9.2	4.596	0.783	0.651	0.727	0.663
	400	24	12	27	6.8	5.584	0.769	0.621	0.716	0.649
	600	28	3	153	25.5	4.587	0.827	0.692	0.742	0.694
3	600	28	9	77	12.8	4.161	0.813	0.654	0.746	0.688
	600	28	7	63	10.5	4.479	0.772	0.643	0.722	0.661
	800	30	3	137	17.1	4.606	0.816	0.681	0.737	0.689
4	800	30	15	132	16.5	3.352	0.857	0.717	0.777	0.726
	800	30	7	111	13.9	4.341	0.780	0.646	0.732	0.671
	1000	30	3	154	15.4	4.634	0.821	0.684	0.737	0.689
5	1000	30	17	152	15.2	3.391	0.854	0.717	0.780	0.725
	1000	30	9	112	11.2	3.925	0.824	0.687	0.759	0.701
	1200	33	11	245	20.4	3.607	0.860	0.723	0.779	0.727
6	1200	33	4	206	17.2	4.229	0.786	0.648	0.740	0.677
	1200	33	3	177	14.7	4.666	0.821	0.690	0.740	0.690
	1400	34	10	412	29.4	4.046	0.841	0.695	0.764	0.707
7	1400	34	3	165	11.8	4.700	0.819	0.684	0.739	0.690
	1400	34	5	152	10.9	4.288	0.800	0.662	0.742	0.683
	1600	36	10	504	31.5	4.034	0.841	0.695	0.762	0.705
8	1600	36	4	283	17.7	4.543	0.786	0.648	0.735	0.672
	1600	36	3	187	11.7	4.496	0.821	0.684	0.745	0.695
	1800	38	11	639	35.5	3.934	0.841	0.695	0.766	0.709
9	1800	38	4	211	11.7	4.668	0.783	0.654	0.725	0.665
	1800	38	8	172	9.6	4.469	0.802	0.670	0.740	0.682
	2000	37	10	639	32.0	4.020	0.838	0.692	0.762	0.705
10	2000	37	4	428	21.4	4.506	0.789	0.651	0.738	0.675
	2000	37	11	247	12.3	3.390	0.898	0.778	0.791	0.749
	4000	44	4	1784	44.6	3.940	0.852	0.712	0.769	0.715
20	4000	44	6	534	13.4	4.264	0.813	0.684	0.751	0.690
	4000	44	19	389	9.7	3.625	0.904	0.789	0.794	0.755
	6000	48	4	3078	51.3	3.997	0.852	0.714	0.771	0.718
30	6000	48	6	904	15.1	4.339	0.810	0.676	0.751	0.689
	6000	48	13	647	10.8	3.798	0.901	0.780	0.790	0.754
	8000	44	4	4297	53.7	3.923	0.852	0.717	0.773	0.721
40	8000	44	7	1103	13.8	4.257	0.813	0.676	0.750	0.687
	8000	44	13	1087	13.6	3.817	0.904	0.783	0.790	0.755
	10000	45	8	4753	47.5	3.930	0.852	0.714	0.774	0.720
50	10000	45	5	1919	19.2	3.862	0.904	0.783	0.792	0.756
	10000	45	7	1303	13.0	4.116	0.816	0.679	0.751	0.687
	20000	53	6	10143	50.7	3.908	0.854	0.712	0.776	0.723
100	20000	53	15	4883	24.4	3.685	0.901	0.783	0.790	0.754
	20000	53	4	2562	12.8	3.919	0.813	0.679	0.749	0.687

Table S6D. Conformational cluster analysis of molecular dynamics simulations of TMR04 using FF12MC at 340 K.

Number of timesteps (x10⁶)	Number of frames	Number of clusters	Three largest cluster ID	Number of frames in cluster	Occurrence of the cluster (%)	CαRMSD (Å)	GDT-TS	GDT-HA	RPF9	LDDT15
	200	6	0	121	60.5	1.333	0.939	0.814	0.801	0.769
1	200	6	2	45	22.5	2.057	0.754	0.550	0.730	0.650
	200	6	1	25	12.5	2.461	0.700	0.504	0.704	0.624
	400	6	0	252	63.0	1.373	0.936	0.814	0.802	0.770
2	400	6	2	89	22.3	1.913	0.789	0.589	0.747	0.665
	400	6	1	56	14.0	2.357	0.714	0.518	0.711	0.639
	600	6	0	512	85.3	1.428	0.921	0.750	0.794	0.751
3	600	6	1	82	13.7	2.219	0.732	0.536	0.723	0.651
	600	6	5	3	0.5	2.684	0.686	0.479	0.679	0.599
	800	7	0	685	85.6	1.506	0.929	0.761	0.793	0.750
4	800	7	1	99	12.4	2.224	0.736	0.539	0.723	0.653
	800	7	3	12	1.5	2.382	0.714	0.521	0.723	0.641
	1000	11	0	829	82.9	1.545	0.929	0.768	0.791	0.752
5	1000	11	1	113	11.3	2.169	0.743	0.539	0.732	0.660
	1000	11	8	27	2.7	2.152	0.800	0.611	0.752	0.678
	1200	11	0	995	82.9	1.548	0.929	0.771	0.791	0.754
6	1200	11	1	132	11.0	2.121	0.746	0.546	0.737	0.665
	1200	11	5	32	2.7	2.390	0.725	0.536	0.720	0.642
	1400	9	0	1160	82.9	1.552	0.925	0.779	0.792	0.757
7	1400	9	1	132	9.4	2.175	0.739	0.539	0.731	0.658
	1400	9	4	72	5.1	2.090	0.768	0.564	0.750	0.663
	1600	9	0	1336	83.5	1.553	0.929	0.782	0.792	0.757
8	1600	9	1	143	8.9	2.164	0.739	0.536	0.735	0.661
	1600	9	4	70	4.4	2.093	0.771	0.571	0.750	0.664
	1800	10	0	1511	83.9	1.544	0.929	0.786	0.793	0.757
9	1800	10	1	181	10.1	2.113	0.754	0.554	0.738	0.667
	1800	10	4	71	3.9	2.108	0.779	0.575	0.747	0.664
	2000	10	0	1677	83.9	1.555	0.929	0.782	0.794	0.756
10	2000	10	6	184	9.2	2.179	0.746	0.546	0.734	0.663
	2000	10	1	56	2.8	1.863	0.829	0.646	0.757	0.681
	4000	17	0	3551	88.8	1.508	0.936	0.796	0.799	0.762
20	4000	17	5	256	6.4	2.182	0.750	0.550	0.731	0.661
	4000	17	1	96	2.4	1.991	0.814	0.621	0.768	0.689
	6000	29	0	5280	88.0	1.527	0.929	0.807	0.800	0.763
30	6000	29	2	317	5.3	2.119	0.757	0.557	0.736	0.667
	6000	29	1	113	1.9	2.014	0.818	0.625	0.764	0.688
	8000	30	0	6959	87.0	1.538	0.929	0.807	0.801	0.764
40	8000	30	1	433	5.4	2.085	0.761	0.561	0.738	0.669
	8000	30	2	147	1.8	2.028	0.796	0.600	0.766	0.682
	10000	44	0	8832	88.3	1.513	0.932	0.807	0.801	0.764
50	10000	44	1	450	4.5	2.103	0.761	0.564	0.737	0.667
	10000	44	2	139	1.4	1.984	0.818	0.629	0.771	0.690
	20000	68	0	17676	88.4	1.520	0.932	0.811	0.800	0.762
100	20000	68	6	828	4.1	2.259	0.743	0.546	0.724	0.655
	20000	68	1	385	1.9	2.325	0.789	0.593	0.749	0.670

Table S6E. Conformational cluster analysis of molecular dynamics simulations of TMR04 using FF14SBlm at 340 K.

Number of timesteps (x10^6)	Number of frames	Number of clusters	Three largest cluster ID	Number of frames in cluster	Occurrence of the cluster (%)	CαRMSD (Å)	GDT-TS	GDT-HA	RPF9	LDDT15
	200	8	0	140	70.0	1.216	0.932	0.804	0.791	0.767
1	200	8	4	20	10.0	3.326	0.657	0.468	0.666	0.608
	200	8	1	10	5.0	2.564	0.714	0.511	0.697	0.634
	400	10	0	279	69.8	1.212	0.936	0.804	0.796	0.771
2	400	10	3	35	8.7	3.608	0.625	0.439	0.653	0.591
	400	10	1	20	5.0	2.409	0.721	0.521	0.711	0.647
	600	11	0	443	73.8	1.199	0.936	0.807	0.798	0.771
3	600	11	4	55	9.2	3.516	0.632	0.443	0.654	0.594
	600	11	2	40	6.7	2.524	0.689	0.493	0.704	0.637
	800	10	0	602	75.2	1.200	0.939	0.811	0.800	0.771
4	800	10	1	81	10.1	2.368	0.711	0.514	0.721	0.650
	800	10	3	68	8.5	3.538	0.625	0.436	0.655	0.594
	1000	12	0	744	74.4	1.217	0.939	0.807	0.799	0.769
5	1000	12	2	93	9.3	2.346	0.711	0.514	0.721	0.652
	1000	12	4	76	7.6	3.579	0.625	0.436	0.654	0.594
	1200	11	0	935	77.9	1.159	0.946	0.818	0.800	0.770
6	1200	11	2	98	8.2	2.333	0.711	0.518	0.721	0.653
	1200	11	4	86	7.2	3.590	0.625	0.436	0.654	0.593
	1400	12	0	1099	78.5	1.131	0.946	0.818	0.801	0.771
7	1400	12	4	97	6.9	3.541	0.621	0.432	0.653	0.594
	1400	12	2	96	6.9	2.330	0.711	0.514	0.720	0.653
	1600	11	0	1278	79.9	1.114	0.950	0.821	0.802	0.772
8	1600	11	4	107	6.7	3.496	0.625	0.432	0.654	0.595
	1600	11	2	96	6.0	2.330	0.711	0.514	0.720	0.653
	1800	12	0	1446	80.3	1.106	0.950	0.825	0.800	0.772
9	1800	12	4	116	6.4	3.498	0.625	0.432	0.651	0.594
	1800	12	2	97	5.4	2.330	0.711	0.514	0.721	0.653
	2000	13	0	1612	80.6	1.110	0.950	0.825	0.801	0.771
10	2000	13	5	123	6.1	3.507	0.629	0.436	0.651	0.593
	2000	13	3	100	5.0	2.333	0.714	0.518	0.720	0.653
	4000	16	0	3456	86.4	1.165	0.943	0.811	0.796	0.768
20	4000	16	5	162	4.1	3.410	0.632	0.439	0.653	0.594
	4000	16	12	96	2.4	1.888	0.839	0.664	0.731	0.683
	6000	26	0	5331	88.8	1.150	0.939	0.807	0.798	0.768
30	6000	26	6	162	2.7	3.410	0.632	0.439	0.653	0.594
	6000	26	22	106	1.8	1.806	0.839	0.668	0.737	0.690
	8000	29	0	7221	90.3	1.149	0.936	0.807	0.800	0.769
40	8000	29	8	162	2.0	3.410	0.632	0.439	0.653	0.594
	8000	29	2	114	1.4	1.775	0.843	0.671	0.741	0.695
	10000	29	0	9199	92.0	1.148	0.936	0.807	0.800	0.769
50	10000	29	22	178	1.8	3.276	0.718	0.536	0.654	0.604
	10000	29	6	162	1.6	3.410	0.632	0.439	0.653	0.594
	20000	40	0	18632	93.2	1.082	0.939	0.818	0.805	0.776
100	20000	40	23	423	2.1	3.356	0.711	0.529	0.648	0.596
	20000	40	6	162	0.8	3.410	0.632	0.439	0.653	0.594

Table S6F. Conformational cluster analysis of molecular dynamics simulations of TMR04 using FF96lm at 340 K.

Number of timesteps ($x10^6$)	Number of frames	Number of clusters	Three largest cluster ID	Number of frames in cluster	Occurrence of the cluster (%)	CαRMSD (Å)	GDT-TS	GDT-HA	RPF9	LDDT15
	200	4	0	179	89.5	1.223	0.900	0.750	0.779	0.743
1	200	4	1	10	5.0	3.644	0.661	0.461	0.630	0.589
	200	4	2	10	5.0	4.359	0.632	0.443	0.601	0.548
	400	6	0	366	91.5	1.245	0.907	0.739	0.782	0.744
2	400	6	3	11	2.8	3.263	0.646	0.443	0.648	0.597
	400	6	4	11	2.8	4.314	0.643	0.450	0.608	0.554
	600	4	0	568	94.7	1.316	0.900	0.732	0.780	0.740
3	600	4	1	19	3.2	3.402	0.657	0.457	0.644	0.600
	600	4	2	11	1.8	4.313	0.643	0.450	0.608	0.554
	800	6	0	641	80.1	1.276	0.929	0.771	0.783	0.752
4	800	6	1	119	14.9	2.184	0.757	0.554	0.724	0.663
	800	6	3	19	2.4	3.402	0.657	0.457	0.644	0.600
	1000	8	0	815	81.5	1.307	0.932	0.779	0.784	0.754
5	1000	8	1	126	12.6	2.204	0.761	0.557	0.721	0.663
	1000	8	5	19	1.9	3.402	0.657	0.457	0.644	0.600
	1200	8	0	978	81.5	1.344	0.929	0.779	0.785	0.755
6	1200	8	1	169	14.1	2.225	0.757	0.561	0.723	0.665
	1200	8	5	19	1.6	3.402	0.657	0.457	0.644	0.600
	1400	7	0	1358	97.0	1.432	0.914	0.746	0.780	0.744
7	1400	7	4	19	1.4	3.402	0.657	0.457	0.644	0.600
	1400	7	6	11	0.8	4.313	0.643	0.450	0.608	0.554
	1600	10	0	1526	95.4	1.448	0.918	0.754	0.781	0.746
8	1600	10	6	23	1.4	3.240	0.664	0.471	0.668	0.607
	1600	10	5	19	1.2	3.402	0.657	0.457	0.644	0.600
	1800	10	0	1554	86.3	1.439	0.925	0.775	0.783	0.752
9	1800	10	1	204	11.3	2.093	0.768	0.571	0.731	0.675
	1800	10	5	19	1.1	3.402	0.657	0.457	0.644	0.600
	2000	11	0	1948	97.4	1.496	0.921	0.754	0.780	0.745
10	2000	11	5	19	0.9	3.402	0.657	0.457	0.644	0.600
	2000	11	4	9	0.4	2.942	0.775	0.579	0.693	0.650
	4000	10	0	3745	93.6	1.562	0.921	0.771	0.778	0.749
20	4000	10	1	173	4.3	2.065	0.775	0.579	0.733	0.674
	4000	10	7	24	0.6	3.202	0.664	0.468	0.671	0.608
	6000	13	0	5716	95.3	1.570	0.921	0.761	0.779	0.748
30	6000	13	1	153	2.5	2.283	0.743	0.546	0.719	0.660
	6000	13	6	35	0.6	1.740	0.839	0.646	0.740	0.694
	8000	15	0	7803	97.5	1.580	0.921	0.771	0.779	0.746
40	8000	15	4	39	0.5	1.944	0.829	0.639	0.730	0.680
	8000	15	2	34	0.4	1.929	0.789	0.596	0.763	0.695
	10000	15	0	9642	96.4	1.601	0.925	0.775	0.778	0.746
50	10000	15	1	190	1.9	2.214	0.775	0.575	0.730	0.672
	10000	15	5	38	0.4	1.947	0.821	0.629	0.730	0.679
	20000	28	0	19397	97.0	1.645	0.921	0.771	0.777	0.744
100	20000	28	1	192	1.0	2.237	0.757	0.557	0.724	0.666
	20000	28	2	103	0.5	1.992	0.789	0.586	0.749	0.689

Table S6G. Conformational cluster analysis of molecular dynamics simulations of TMR07 using FF12MC at 340 K.

Number of timesteps (x10^6)	Number of frames	Number of clusters	Three largest cluster ID	Number of frames in cluster	Occurrence of the cluster (%)	CαRMSD (Å)	GDT-TS	GDT-HA	RPF9	LDDT15
	200	39	0	10	5.0	2.457	0.741	0.537	0.730	0.662
1	200	39	5	10	5.0	3.067	0.797	0.608	0.739	0.674
	200	39	16	10	5.0	2.585	0.787	0.608	0.749	0.691
	400	66	0	20	5.0	2.388	0.743	0.544	0.742	0.668
2	400	66	10	20	5.0	2.848	0.811	0.631	0.751	0.690
	400	66	41	20	5.0	2.284	0.778	0.584	0.738	0.670
	600	100	86	24	4.0	2.830	0.729	0.521	0.734	0.663
3	600	100	48	23	3.8	3.128	0.736	0.535	0.709	0.642
	600	100	13	21	3.5	2.846	0.813	0.633	0.750	0.690
	800	122	102	34	4.3	2.775	0.741	0.537	0.743	0.673
4	800	122	55	33	4.1	3.150	0.734	0.535	0.713	0.647
	800	122	1	28	3.5	2.382	0.766	0.570	0.739	0.669
	1000	138	60	43	4.3	3.106	0.741	0.544	0.715	0.649
5	1000	138	1	38	3.8	2.373	0.752	0.549	0.738	0.665
	1000	138	114	37	3.7	2.773	0.741	0.535	0.744	0.674
	1200	151	68	53	4.4	3.072	0.741	0.540	0.715	0.649
6	1200	151	1	45	3.7	2.296	0.755	0.551	0.743	0.670
	1200	151	92	40	3.3	2.351	0.773	0.579	0.752	0.678
	1400	163	72	55	3.9	3.040	0.738	0.533	0.713	0.646
7	1400	163	100	50	3.6	2.381	0.773	0.577	0.751	0.675
	1400	163	21	49	3.5	2.435	0.792	0.601	0.736	0.680
	1600	177	80	65	4.1	2.985	0.738	0.540	0.715	0.646
8	1600	177	109	60	3.7	2.347	0.771	0.572	0.753	0.676
	1600	177	22	55	3.4	2.468	0.794	0.605	0.735	0.680
	1800	183	84	75	4.2	2.989	0.741	0.544	0.715	0.646
9	1800	183	113	70	3.9	2.333	0.776	0.577	0.755	0.676
	1800	183	22	50	2.8	2.439	0.792	0.601	0.735	0.680
	2000	191	86	85	4.3	3.005	0.741	0.544	0.715	0.645
10	2000	191	118	80	4.0	3.005	0.741	0.544	0.715	0.645
	2000	191	78	60	3.0	2.317	0.783	0.589	0.755	0.677
	4000	269	134	198	5.0	2.328	0.851	0.682	0.813	0.765
20	4000	269	172	182	4.5	2.193	0.801	0.617	0.764	0.691
	4000	269	226	157	3.9	3.378	0.750	0.568	0.739	0.675
	6000	353	80	450	7.5	2.342	0.865	0.699	0.813	0.767
30	6000	353	223	278	4.6	2.187	0.801	0.617	0.762	0.692
	6000	353	110	217	3.6	3.020	0.822	0.661	0.753	0.723
	8000	412	89	687	8.6	2.375	0.858	0.696	0.806	0.760
40	8000	412	261	378	4.7	2.375	0.858	0.696	0.000	0.760
	8000	412	72	311	3.9	2.986	0.757	0.551	0.691	0.647
	10000	504	108	809	8.1	2.392	0.851	0.678	0.807	0.761
50	10000	504	311	397	4.0	2.142	0.806	0.617	0.768	0.696
	10000	504	73	314	3.1	2.985	0.755	0.549	0.691	0.647
	20000	858	24	2422	12.1	2.403	0.846	0.680	0.815	0.762
100	20000	858	237	2355	11.8	2.400	0.841	0.664	0.804	0.755
	20000	858	550	610	3.0	2.096	0.818	0.624	0.776	0.709

Table S6H. Conformational cluster analysis of molecular dynamics simulations of TMR07 using FF14SBlm at 340 K.

Number of timesteps ($x10^6$)	Number of frames	Number of clusters	Three largest cluster ID	Number of frames in cluster	Occurrence of the cluster (%)	CαRMSD (Å)	GDT-TS	GDT-HA	RPF9	LDDT15
	200	29	0	10	5.0	3.243	0.750	0.554	0.705	0.643
1	200	29	3	10	5.0	2.655	0.785	0.601	0.696	0.660
	200	29	4	10	5.0	3.071	0.757	0.558	0.685	0.631
	400	40	0	20	5.0	3.063	0.787	0.610	0.720	0.670
2	400	40	4	20	5.0	2.594	0.808	0.624	0.706	0.668
	400	40	24	20	5.0	2.869	0.797	0.624	0.728	0.685
	600	54	0	30	5.0	3.045	0.801	0.624	0.729	0.685
3	600	54	4	30	5.0	2.417	0.813	0.636	0.715	0.675
	600	54	37	30	5.0	3.119	0.752	0.568	0.668	0.617
	800	69	0	40	5.0	2.999	0.811	0.636	0.731	0.689
4	800	69	4	40	5.0	2.280	0.815	0.638	0.721	0.678
	800	69	37	34	4.3	3.034	0.792	0.626	0.726	0.688
	1000	86	0	50	5.0	2.948	0.815	0.640	0.731	0.690
5	1000	86	5	50	5.0	2.217	0.808	0.629	0.727	0.682
	1000	86	76	44	4.4	2.118	0.785	0.598	0.720	0.674
	1200	97	6	60	5.0	2.168	0.808	0.629	0.730	0.685
6	1200	97	0	52	4.3	2.951	0.815	0.643	0.731	0.689
	1200	97	19	52	4.3	3.927	0.594	0.383	0.620	0.556
	1400	108	6	69	4.9	2.159	0.811	0.633	0.732	0.689
7	1400	108	24	62	4.4	3.920	0.598	0.390	0.621	0.555
	1400	108	28	61	4.4	4.060	0.682	0.488	0.677	0.615
	1600	119	6	78	4.9	2.145	0.811	0.636	0.734	0.691
8	1600	119	29	72	4.5	3.933	0.596	0.388	0.621	0.554
	1600	119	33	71	4.4	4.085	0.680	0.486	0.677	0.616
	1800	135	8	88	4.9	2.119	0.815	0.633	0.736	0.692
9	1800	135	37	82	4.6	3.946	0.603	0.395	0.621	0.553
	1800	135	41	81	4.5	4.073	0.682	0.486	0.678	0.618
	2000	145	9	98	4.9	2.114	0.818	0.636	0.735	0.691
10	2000	145	41	92	4.6	3.955	0.598	0.388	0.620	0.554
	2000	145	45	92	4.6	4.080	0.680	0.484	0.676	0.619
	4000	211	18	251	6.3	2.023	0.827	0.647	0.745	0.698
20	4000	211	1	160	4.0	2.402	0.806	0.633	0.726	0.683
	4000	211	95	153	3.8	3.452	0.701	0.509	0.651	0.609
	6000	268	36	352	5.9	1.992	0.825	0.647	0.749	0.701
30	6000	268	1	310	5.2	2.192	0.818	0.645	0.740	0.694
	6000	268	130	253	4.2	3.452	0.699	0.505	0.651	0.609
	8000	323	56	452	5.7	1.979	0.825	0.650	0.750	0.702
40	8000	323	1	410	5.1	2.086	0.820	0.647	0.748	0.701
	8000	323	169	353	4.4	3.468	0.699	0.502	0.652	0.608
	10000	400	90	556	5.6	1.963	0.827	0.650	0.752	0.703
50	10000	400	1	506	5.1	1.999	0.832	0.659	0.753	0.706
	10000	400	220	458	4.6	3.488	0.699	0.502	0.651	0.606
	20000	597	1	1469	7.3	1.789	0.832	0.654	0.762	0.713
100	20000	597	320	917	4.6	—	—	—	—	—
	20000	597	366	910	4.5	—	—	—	—	—

Table S6I. Conformational cluster analysis of molecular dynamics simulations of TMR07 using FF96lm at 340 K.

Number of timesteps (x10^6)	Number of frames	Number of clusters	Three largest cluster ID	Number of frames in cluster	Occurrence of the cluster (%)	CαRMSD (Å)	GDT-TS	GDT-HA	RPF9	LDDT15
	200	59	11	10	5.0	3.094	0.708	0.500	0.704	0.625
1	200	59	12	10	5.0	2.550	0.748	0.547	0.721	0.651
	200	59	28	10	5.0	3.706	0.610	0.402	0.608	0.544
	400	99	16	20	5.0	3.021	0.720	0.509	0.708	0.630
2	400	99	71	20	5.0	3.568	0.764	0.586	0.678	0.639
	400	99	17	19	4.8	2.481	0.769	0.565	0.725	0.660
	600	142	33	26	4.3	2.458	0.766	0.570	0.731	0.665
3	600	142	21	25	4.2	3.571	0.645	0.439	0.664	0.585
	600	142	1	23	3.8	3.121	0.706	0.495	0.706	0.634
	800	169	1	30	3.7	3.162	0.706	0.495	0.707	0.635
4	800	169	26	29	3.6	3.523	0.643	0.437	0.665	0.589
	800	169	41	27	3.4	2.444	0.766	0.570	0.732	0.666
	1000	196	1	32	3.2	3.173	0.703	0.493	0.706	0.635
5	1000	196	31	28	2.8	3.515	0.645	0.437	0.664	0.588
	1000	196	48	27	2.7	2.444	0.766	0.570	0.732	0.666
	1200	220	1	32	2.7	3.173	0.703	0.493	0.706	0.635
6	1200	220	35	28	2.3	3.515	0.645	0.437	0.664	0.588
	1200	220	58	27	2.2	2.444	0.766	0.570	0.732	0.666
	1400	258	1	32	2.3	3.173	0.703	0.493	0.706	0.635
7	1400	258	121	32	2.3	3.691	0.703	0.505	0.701	0.633
	1400	258	40	28	2.0	3.515	0.645	0.437	0.664	0.588
	1600	284	253	37	2.3	3.515	0.645	0.437	0.000	0.588
8	1600	284	136	32	2.0	3.691	0.703	0.505	0.701	0.633
	1600	284	1	32	2.0	3.173	0.703	0.493	0.706	0.635
	1800	302	269	43	2.4	3.173	0.703	0.493	0.000	0.635
9	1800	302	98	41	2.3	2.951	0.757	0.558	0.726	0.670
	1800	302	300	34	1.9	2.951	0.757	0.558	0.000	0.670
	2000	325	106	48	2.4	2.999	0.750	0.554	0.727	0.669
10	2000	325	289	47	2.4	2.999	0.750	0.554	0.000	0.669
	2000	325	321	38	1.9	2.999	0.750	0.554	0.000	0.669
	4000	536	7	129	3.2	2.318	0.839	0.657	0.781	0.742
20	4000	536	214	128	3.2	3.968	0.713	0.526	0.682	0.631
	4000	536	252	127	3.2	3.968	0.713	0.526	0.000	0.631
	6000	688	7	257	4.3	2.367	0.846	0.680	0.790	0.752
30	6000	688	292	200	3.3	2.367	0.846	0.680	0.000	0.752
	6000	688	280	185	3.1	2.367	0.846	0.680	0.000	0.752
	8000	808	7	358	4.5	2.350	0.858	0.699	0.794	0.760
40	8000	808	680	249	3.1	2.350	0.858	0.699	0.000	0.760
	8000	808	388	193	2.4	2.350	0.858	0.699	0.000	0.760
	10000	897	7	362	3.6	2.331	0.858	0.703	0.794	0.762
50	10000	897	756	291	2.9	2.331	0.858	0.703	0.000	0.762
	10000	897	443	270	2.7	2.331	0.858	0.703	0.000	0.762
	20000	1291	8	1154	5.8	2.736	0.872	0.710	0.793	0.765
100	20000	1291	703	526	2.6	–	–	–	–	–
	20000	1291	713	526	2.6	–	–	–	–	–

Table S7. Effect of timestep size on the quality of 20 or 30 distinct and independent isothermal-isobaric molecular dynamics simulations.

Forcefield	Temp (K)	Nonbonded Cutoff (Å)	Timestep Size (fs^{smt})	Number of Timesteps	Simulation Time (ns^{smt})	Number of Simulations	$\langle \Delta E^2 \rangle^{1/2} / \langle \Delta KE^2 \rangle^{1/2}$	
							mean	SE
Ala3								
FF14SB	300	8	1.00	100000	100	20	0.4097	0.0012
FF14SB	300	8	1.00	200000	200	20	0.4096	0.0007
FF12MC	300	8	1.00	31646	100	20	0.2890	0.0014
FF12MC	300	8	1.00	63292	200	20	0.2636	0.0007
FF12MC	300	8	1.00	100000	316	20	0.2540	0.0006
FF12MC	300	8	1.00	200000	632	20	0.2452	0.0005
Ala5								
FF14SB	300	8	1.00	100000	100	20	0.4224	0.0008
FF14SB	300	8	1.00	200000	200	20	0.4226	0.0007
FF12MC	300	8	1.00	31646	100	20	0.3138	0.0023
FF12MC	300	8	1.00	63292	200	20	0.2810	0.0013
FF12MC	300	8	1.00	100000	316	20	0.2681	0.0009
FF12MC	300	8	1.00	200000	632	20	0.2565	0.0005
Ala7								
FF14SB	300	8	1.00	100000	100	20	0.4291	0.0010
FF14SB	300	8	1.00	200000	200	20	0.4312	0.0009
FF12MC	300	8	1.00	31646	100	20	0.3294	0.0018
FF12MC	300	8	1.00	63292	200	20	0.2935	0.0011
FF12MC	300	8	1.00	100000	316	20	0.2792	0.0008
FF12MC	300	8	1.00	200000	632	20	0.2655	0.0004
Val3								
FF14SB	300	8	1.00	100000	100	20	0.4229	0.0010
FF14SB	300	8	1.00	200000	200	20	0.4227	0.0007
FF14SB	300	8	1.00	300000	300	20	0.4225	0.0006
FF14SB	300	8	1.00	400000	400	20	0.4224	0.0005
FF12MC	300	8	1.00	31646	100	20	0.3019	0.0020
FF12MC	300	8	1.00	63292	200	20	0.2754	0.0012
FF12MC	300	8	1.00	100000	316	20	0.2647	0.0010
FF12MC	300	8	1.00	200000	632	20	0.2558	0.0006
FF14SB	300	9	1.00	100000	100	20	0.4148	0.0013
FF14SB	300	9	1.00	200000	200	20	0.4151	0.0008
FF14SB	300	9	1.00	300000	300	20	0.4150	0.0006
FF14SB	300	9	1.00	400000	400	20	0.4152	0.0004
FF14SB	300	9	1.00	500000	500	20	0.4153	0.0004
FF12MC	300	9	1.00	31646	100	20	0.2410	0.0007
FF12MC	300	9	1.00	63292	200	20	0.2406	0.0007
FF12MC	300	9	1.00	100000	316	20	0.2406	0.0005
FF12MC	300	9	1.00	200000	632	20	0.2405	0.0004
FF12MC	300	9	1.00	300000	948	20	0.2407	0.0003
FF12MC	300	9	1.00	400000	1264	20	0.2408	0.0003
FF12MC	300	9	1.00	500000	1580	20	0.2409	0.0002
AAQAA								
FF14SB	274	8	1.00	100000	100	20	0.5048	0.0016
FF14SB	274	8	1.00	200000	200	20	0.5062	0.0013
FF14SB	274	8	1.00	300000	300	20	0.5064	0.0009
FF14SB	274	8	1.00	1000000	1000	20	0.5054	0.0005
FF12SB	274	8	1.00	100000	100	20	0.5014	0.0018
FF12SB	274	8	1.00	200000	200	20	0.5036	0.0018
FF12SB	274	8	1.00	300000	300	20	0.5044	0.0014
FF12SB	274	8	1.00	1000000	1000	20	0.5042	0.0009
FF12MC	274	8	1.00	31646	100	20	0.3131	0.0040
FF12MC	274	8	1.00	63292	200	20	0.3228	0.0039
FF12MC	274	8	1.00	100000	316	20	0.3277	0.0036
FF12MC	274	8	1.00	300000	948	20	0.3275	0.0029
FF12MC	274	8	1.00	1000000	3160	20	0.3206	0.0097
FF12SB	300	8	1.00	100000	100	20	0.4423	0.0018
FF12SB	300	8	1.00	200000	200	20	0.4416	0.0011
FF12SB	300	8	1.00	300000	300	20	0.4429	0.0012
FF12SB	300	8	1.00	1000000	1000	20	0.4430	0.0006

FF12MC	300	8	1.00	31646	100	20	0.2786	0.0030
FF12MC	300	8	1.00	63292	200	20	0.2834	0.0026
FF12MC	300	8	1.00	100000	316	20	0.2870	0.0015
FF12MC	300	8	1.00	300000	948	20	0.2925	0.0008
FF12MC	300	8	1.00	1000000	3160	20	0.2929	0.0006
FF12SB	310	8	1.00	100000	100	20	0.4272	0.0009
FF12SB	310	8	1.00	200000	200	20	0.4278	0.0007
FF12SB	310	8	1.00	300000	300	20	0.4277	0.0006
FF12SB	310	8	1.00	1000000	1000	20	0.4276	0.0005
FF12MC	310	8	1.00	31646	100	20	0.2728	0.0025
FF12MC	310	8	1.00	63292	200	20	0.2806	0.0019
FF12MC	310	8	1.00	100000	316	20	0.2811	0.0013
FF12MC	310	8	1.00	300000	948	20	0.2816	0.0008
FF12MC	310	8	1.00	1000000	3160	20	0.2830	0.0005
Chignolin								
FF14SB	277	8	1.00	100000	100	20	0.4933	0.0010
FF14SB	277	8	1.00	200000	200	20	0.4934	0.0009
FF14SB	277	8	1.00	300000	300	20	0.4939	0.0007
FF14SB	277	8	1.00	1000000	1000	20	0.4932	0.0005
FF12MC	277	8	1.00	31646	100	20	0.3685	0.0032
FF12MC	277	8	1.00	63292	200	20	0.3378	0.0024
FF12MC	277	8	1.00	100000	316	20	0.3138	0.0032
FF12MC	277	8	1.00	300000	948	20	0.3058	0.0014
FF12MC	277	8	1.00	1000000	3160	20	0.3024	0.0008
FF12MC	300	8	1.00	31646	100	20	0.3413	0.0020
FF12MC	300	8	1.00	63292	200	20	0.3094	0.0014
FF12MC	300	8	1.00	100000	316	20	0.2972	0.0012
FF12MC	300	8	1.00	300000	948	20	0.2810	0.0007
FF12MC	300	8	1.00	500000	1580	20	0.2778	0.0006
FF12MC	300	8	1.00	1000000	3160	20	0.2757	0.0005
CLN025								
FF14SB	277	8	1.00	100000	100	20	0.4942	0.0013
FF14SB	277	8	1.00	200000	200	20	0.4942	0.0013
FF14SB	277	8	1.00	300000	300	20	0.4941	0.0010
FF14SB	277	8	1.00	1000000	1000	20	0.4936	0.0006
FF12MC	277	8	1.00	31646	100	20	0.3527	0.0029
FF12MC	277	8	1.00	63292	200	20	0.3298	0.0019
FF12MC	277	8	1.00	100000	316	20	0.3193	0.0013
FF12MC	277	8	1.00	300000	948	20	0.3071	0.0006
FF12MC	277	8	1.00	1000000	3160	20	0.3020	0.0006
FF14SBlm	277	8	1.00	100000	316	20	0.3036	0.0008
FF14SBlm	277	8	1.00	300000	948	20	0.3020	0.0008
FF14SBlm	277	8	1.00	1000000	3160	20	0.2984	0.0009
Trp-cage								
FF12MC	280	8	1.00	1000000	3160	30	0.3025	0.0010
FF12MC	280	8	1.00	2000000	6320	30	0.3019	0.0010
FF12MC	280	8	1.00	2800000	8848	30	0.3007	0.0010
1IGD								
FF14SB	297	8	1.00	100000	100	20	0.5008	0.0016
FF14SBlm	297	8	1.00	100000	316	20	0.3238	0.0013
FF14SBlm	297	8	1.00	200000	632	20	0.3237	0.0009
FF14SBlm	297	8	1.00	300000	948	20	0.3240	0.0006
FF12MCsm	297	8	1.00	100000	100	20	0.5011	0.0013
FF12MC	297	8	1.00	100000	316	20	0.3199	0.0006
FF12MC	297	8	1.00	200000	632	20	0.3200	0.0004
FF12MC	297	8	1.00	300000	948	20	0.3202	0.0004
1UBQ								
FF14SB	300	8	1.00	100000	100	20	0.4893	0.0012
FF14SBlm	300	8	1.00	100000	316	20	0.3165	0.0010
FF14SBlm	300	8	1.00	200000	632	20	0.3177	0.0007
FF14SBlm	300	8	1.00	300000	948	20	0.3172	0.0007
FF12MCsm	300	8	1.00	100000	100	20	0.4945	0.0014
FF12MC	300	8	1.00	100000	316	20	0.3240	0.0019
FF12MC	300	8	1.00	200000	632	20	0.3258	0.0016
FF12MC	300	8	1.00	300000	948	20	0.3262	0.0013

1PIT								
FF14SBlm	309	8	1.00	100000	316	20	0.3115	0.0030
FF12MC	309	8	1.00	100000	316	20	0.2998	0.0012
1QLQ								
FF14SBlm	290	8	1.00	100000	316	20	0.3115	0.0010
FF12MC	290	8	1.00	100000	316	20	0.3108	0.0015
4LZT								
FF14SBlm	308	8	1.00	100000	316	20	0.3087	0.0013
FF12MC	308	8	1.00	100000	316	20	0.3213	0.0022
TMR01								
FF14SBlm	340	8	1.00	100000	316	20	0.3111	0.0027
FF12MC	340	8	1.00	100000	316	20	0.2927	0.0014
TMR04								
FF14SBlm	340	8	1.00	100000	316	20	0.3021	0.0014
FF12MC	340	8	1.00	100000	316	20	0.2940	0.0013
TMR07								
FF14SBlm	340	8	1.00	100000	316	20	0.3501	0.0042
FF12MC	340	8	1.00	100000	316	20	0.3379	0.0039

$\langle \Delta E^2 \rangle^{1/2} / \langle \Delta KE^2 \rangle^{1/2}$ is a ratio of the root mean square fluctuation of the total energy of the simulation system to the root mean square fluctuation of the kinetic energy of the system.

Table S8A. Experimental and calculated *J*-coupling constants of Ala3 in water at 300 K.

		FF12MC (4 μs^{lmt})				FF12SB (4 μs^{smt})				FF14SB (4 μs^{smt})			
	EXPT[5]	ORIG	SCHM	DFT1	DFT2	ORIG	SCHM	DFT1	DFT2	ORIG	SCHM	DFT1	DFT2
Residue 2 of Ala3													
3JHnHa	5.68	6.44	5.74	6.19	6.75	5.88	5.14	5.47	6.10	5.88	5.13	5.46	6.10
3JHnC'	1.13	1.14	1.44	1.17	0.93	1.22	1.54	1.25	0.92	1.22	1.54	1.24	0.91
3JHaC'	1.84	1.88	1.88	1.73	1.93	1.71	1.69	1.51	1.68	1.69	1.67	1.49	1.67
3JC'C'	0.25	0.89	0.84	0.79	0.86	0.79	0.72	0.66	0.73	0.79	0.73	0.66	0.74
3JHnCb	2.39	1.71	1.78	3.25	2.55	1.90	1.97	3.64	2.89	1.90	1.97	3.65	2.89
1JNCa	11.34	10.63	10.63	10.63	10.63	11.44	11.44	11.44	11.44	11.43	11.43	11.43	11.43
2JNCa	9.14	8.52	8.52	8.52	8.52	8.54	8.54	8.54	8.54	8.54	8.54	8.54	8.54
3JHnCa	0.70	0.67	0.67	0.67	0.67	0.65	0.65	0.65	0.65	0.65	0.65	0.65	0.65
Residue 3 of Ala3													
3JHnHa	6.52	6.72	6.05	6.57	7.15	5.60	4.87	5.13	5.78	5.61	4.89	5.15	5.78
3JHnC'	1.29	0.96	1.25	0.93	0.73	1.23	1.59	1.23	0.91	1.24	1.60	1.24	0.93
3JHaC'	2.14	2.02	2.02	1.90	2.12	1.81	1.74	1.57	1.74	1.86	1.78	1.61	1.78
3JHnCb	2.02	1.72	1.79	3.29	2.56	2.02	2.09	3.89	3.09	2.01	2.08	3.86	3.07
1JNCa	11.47	10.48	10.48	10.48	10.48	10.72	10.72	10.72	10.72	10.71	10.71	10.71	10.71
2JNCa	8.45	8.10	8.10	8.10	8.10	8.37	8.37	8.37	8.37	8.36	8.36	8.36	8.36
3JHnCa	0.65	0.57	0.57	0.57	0.57	0.58	0.58	0.58	0.58	0.58	0.58	0.58	0.58

Each *J*-coupling constant was calculated according to Eqs. S1–S8 with parameters in Table S9A and backbone torsions obtained from 20 unique and independent 200-million–timestep NPT MD simulations in water at 300 K using a cutoff of 8.0 Å for nonbonded interactions and the Particle Mesh Ewald method to calculate electrostatic interactions of two atoms at separations of >8.0 Å and additional conditions specified in Methods and Table S1.

Table S8B. Experimental and calculated *J*-coupling constants of Ala5 in water at 300 K.

		FF12MC (4 μs^{lmt})				FF12SB (4 μs^{smt})				FF14SB (4 μs^{smt})			
	EXPT[5]	ORIG	SCHM	DFT1	DFT2	ORIG	SCHM	DFT1	DFT2	ORIG	SCHM	DFT1	DFT2
						Residue 2 of Ala5							
3JHnHa	5.59	6.50	5.81	6.28	6.83	5.87	5.13	5.45	6.11	5.84	5.10	5.42	6.08
3JHnC'	1.13	1.12	1.41	1.14	0.91	1.20	1.52	1.21	0.88	1.20	1.53	1.21	0.88
3JHaC'	1.85	1.91	1.91	1.77	1.97	1.69	1.67	1.48	1.66	1.67	1.65	1.47	1.64
3JC'C'	0.19	0.89	0.84	0.79	0.85	0.78	0.71	0.63	0.71	0.77	0.70	0.62	0.70
3JHnCb	2.30	1.70	1.77	3.23	2.53	1.92	1.99	3.69	2.92	1.93	2.00	3.72	2.94
1JNCa	11.36	10.64	10.64	10.64	10.64	11.41	11.41	11.41	11.41	11.41	11.41	11.41	11.41
2JNCa	9.20	8.51	8.51	8.51	8.51	8.54	8.54	8.54	8.54	8.54	8.54	8.54	8.54
3JHnCa	0.67	0.67	0.67	0.67	0.67	0.65	0.65	0.65	0.65	0.65	0.65	0.65	0.65
						Residue 3 of Ala5							
3JHnHa	5.74	6.48	5.79	6.26	6.83	5.90	5.18	5.51	6.15	5.91	5.20	5.52	6.14
3JHaC'	1.86	1.96	1.95	1.82	2.02	1.79	1.76	1.59	1.77	1.87	1.82	1.66	1.84
3JHnCb	2.24	1.75	1.82	3.33	2.61	1.93	2.00	3.72	2.94	1.92	1.99	3.68	2.91
1JNCa	11.26	10.46	10.46	10.46	10.46	11.20	11.20	11.20	11.20	11.15	11.15	11.15	11.15
2JNCa	8.55	8.11	8.11	8.11	8.11	8.30	8.30	8.30	8.30	8.31	8.31	8.31	8.31
3JHnCa	0.68	0.57	0.57	0.57	0.57	0.59	0.59	0.59	0.59	0.59	0.59	0.59	0.59
						Residue 4 of Ala5							
3JHnHa	5.98	6.46	5.77	6.23	6.81	6.03	5.34	5.69	6.25	6.05	5.37	5.72	6.27
3JHnC'	1.15	1.08	1.38	1.09	0.85	1.19	1.52	1.20	0.94	1.19	1.52	1.21	0.96
3JHaC'	1.89	1.94	1.93	1.79	2.00	1.99	1.93	1.78	1.97	1.99	1.94	1.80	1.98
3JHnCb	2.14	1.74	1.81	3.33	2.61	1.87	1.93	3.54	2.80	1.85	1.92	3.50	2.77
1JNCa	11.25	10.43	10.43	10.43	10.43	11.16	11.16	11.16	11.16	11.16	11.16	11.16	11.16
2JNCa	8.40	7.95	7.95	7.95	7.95	8.06	8.06	8.06	8.06	7.97	7.97	7.97	7.97
3JHnCa	0.69	0.54	0.54	0.54	0.54	0.57	0.57	0.57	0.57	0.56	0.56	0.56	0.56
						Residue 5 of Ala5							
3JHnHa	6.54	6.78	6.11	6.64	7.21	5.69	4.98	5.25	5.88	5.69	4.97	5.24	5.88
3JHnC'	1.16	0.96	1.25	0.94	0.75	1.23	1.59	1.23	0.93	1.22	1.58	1.23	0.92
3JHaC'	2.19	2.04	2.05	1.93	2.15	1.87	1.80	1.63	1.81	1.85	1.78	1.61	1.79
3JHnCb	1.96	1.69	1.76	3.22	2.51	1.98	2.05	3.80	3.01	1.99	2.05	3.81	3.03
1JNCa	11.49	10.43	10.43	10.43	10.43	10.68	10.68	10.68	10.68	10.68	10.68	10.68	10.68
2JNCa	8.27	7.94	7.94	7.94	7.94	7.98	7.98	7.98	7.98	7.97	7.97	7.97	7.97
3JHnCa	0.73	0.55	0.55	0.55	0.55	0.55	0.55	0.55	0.55	0.55	0.55	0.55	0.55

Each J-coupling constant was calculated according to Eqs. S1–S8 with parameters in Table S9A and backbone torsions obtained from 20 unique and independent 200-million–timestep NPT MD simulations in water at 300 K using a cutoff of 8.0 Å for nonbonded interactions and the Particle Mesh Ewald method to calculate electrostatic interactions of two atoms at separations of >8.0 Å and additional conditions specified in Methods and Table S1.

Table S8C. Experimental and calculated *J*-coupling constants of Ala7 in water at 300 K.

		FF12MC (4 μs^{lmt})				FF12SB (4 μs^{smt})				FF14SB (4 μs^{smt})			
	EXPT[5]	ORIG	SCHM	DFT1	DFT2	ORIG	SCHM	DFT1	DFT2	ORIG	SCHM	DFT1	DFT2
						Residue 2 of Ala7							
3JHnHa	5.61	6.41	5.71	6.16	6.71	5.83	5.08	5.40	6.05	5.83	5.09	5.40	6.04
3JHnC'	1.15	1.16	1.45	1.19	0.94	1.21	1.54	1.23	0.90	1.22	1.55	1.23	0.91
3JHaC'	1.89	1.89	1.89	1.74	1.94	1.69	1.67	1.49	1.66	1.73	1.70	1.52	1.70
3JHnCb	2.31	1.71	1.78	3.26	2.56	1.93	2.00	3.71	2.94	1.93	2.00	3.70	2.93
1JNCa	11.37	10.62	10.62	10.62	10.62	11.41	11.41	11.41	11.41	11.39	11.39	11.39	11.39
2JNCa	9.17	8.51	8.51	8.51	8.51	8.54	8.54	8.54	8.54	8.54	8.54	8.54	8.54
3JHnCa	0.71	0.67	0.67	0.67	0.67	0.65	0.65	0.65	0.65	0.65	0.65	0.65	0.65
						Residue 3 of Ala7							
3JHnHa	5.66	6.32	5.63	6.05	6.62	5.87	5.18	5.49	6.07	5.86	5.17	5.48	6.06
3JHnC'	1.20	1.14	1.45	1.15	0.91	1.20	1.54	1.20	0.94	1.21	1.55	1.21	0.94
3JHaC'	1.85	1.91	1.90	1.75	1.95	1.98	1.90	1.75	1.94	1.98	1.90	1.76	1.94
3JHnCb	2.20	1.76	1.83	3.37	2.65	1.93	1.99	3.66	2.90	1.93	1.99	3.66	2.90
1JNCa	11.27	10.41	10.41	10.41	10.41	11.08	11.08	11.08	11.08	11.05	11.05	11.05	11.05
2JNCa	8.52	8.08	8.08	8.08	8.08	8.31	8.31	8.31	8.31	8.29	8.29	8.29	8.29
3JHnCa	0.66	0.56	0.56	0.56	0.56	0.59	0.59	0.59	0.59	0.59	0.59	0.59	0.59
						Residue 4 of Ala7							
3JHnHa	5.77	6.38	5.69	6.13	6.69	5.95	5.24	5.58	6.19	5.95	5.23	5.57	6.20
3JHnC'	1.20	1.13	1.43	1.14	0.90	1.17	1.50	1.17	0.89	1.16	1.49	1.16	0.87
3JHaC'	1.80	1.94	1.92	1.78	1.98	1.87	1.82	1.67	1.85	1.83	1.79	1.63	1.81
3JHnCb	2.23	1.75	1.82	3.34	2.61	1.91	1.98	3.65	2.89	1.92	1.98	3.67	2.90
1JNCa	11.22	10.38	10.38	10.38	10.38	11.02	11.02	11.02	11.02	11.03	11.03	11.03	11.03
2JNCa	8.29	7.89	7.89	7.89	7.89	7.87	7.87	7.87	7.87	7.81	7.81	7.81	7.81
3JHnCa	0.56	0.53	0.53	0.53	0.53	0.54	0.54	0.54	0.54	0.53	0.53	0.53	0.53
						Residue 5 of Ala7							
3JHnHa	5.92	6.41	5.72	6.17	6.73	6.00	5.27	5.62	6.29	6.04	5.33	5.69	6.32
3JHnC'	1.19	1.11	1.41	1.12	0.88	1.12	1.44	1.11	0.80	1.13	1.45	1.12	0.83
3JHaC'	1.56	1.94	1.93	1.79	1.99	1.76	1.74	1.56	1.75	1.84	1.81	1.65	1.84
3JHnCb	2.23	1.75	1.82	3.33	2.61	1.92	1.99	3.70	2.92	1.90	1.97	3.64	2.88
1JNCa	11.29	10.35	10.35	10.35	10.35	11.06	11.06	11.06	11.06	11.04	11.04	11.04	11.04
2JNCa	8.22	7.84	7.84	7.84	7.84	7.75	7.75	7.75	7.75	7.77	7.77	7.77	7.77
						Residue 6 of Ala7							
3JHnHa	6.04	6.41	5.71	6.16	6.73	6.09	5.41	5.77	6.33	6.06	5.37	5.73	6.32
3JHnC'	1.10	1.11	1.41	1.12	0.87	1.15	1.48	1.15	0.91	1.14	1.47	1.15	0.88
3JHaC'	1.67	1.91	1.90	1.76	1.96	2.01	1.95	1.81	2.00	1.93	1.89	1.74	1.93
3JHnCb	2.21	1.75	1.82	3.34	2.62	1.86	1.93	3.53	2.79	1.88	1.94	3.58	2.83
1JNCa	11.29	10.36	10.36	10.36	10.36	11.10	11.10	11.10	11.10	11.10	11.10	11.10	11.10
2JNCa	8.24	7.82	7.82	7.82	7.82	7.81	7.81	7.81	7.81	7.77	7.77	7.77	7.77
						Residue 7 of Ala7							
3JHnHa	6.60	6.79	6.13	6.66	7.22	5.70	4.99	5.26	5.87	5.69	4.98	5.25	5.88
3JHnC'	1.25	0.97	1.25	0.95	0.76	1.24	1.59	1.24	0.95	1.23	1.58	1.23	0.93
3JHaC'	2.03	2.04	2.05	1.93	2.15	1.91	1.83	1.67	1.84	1.87	1.79	1.63	1.81
3JHnCb	1.99	1.68	1.75	3.21	2.49	1.97	2.04	3.78	3.00	1.98	2.05	3.80	3.02
1JNCa	11.51	10.41	10.41	10.41	10.41	10.66	10.66	10.66	10.66	10.67	10.67	10.67	10.67
2JNCa	8.18	7.84	7.84	7.84	7.84	7.87	7.87	7.87	7.87	7.87	7.87	7.87	7.87
3JHnCa	0.59	0.53	0.53	0.53	0.53	0.53	0.53	0.53	0.53	0.53	0.53	0.53	0.53

Each *J*-coupling constant was calculated according to Eqs. S1–S8 with parameters in Table S9A and backbone torsions obtained from 20 unique and independent 200-million–timestep NPT MD simulations in water at 300 K using a cutoff of 8.0 Å for nonbonded interactions and the Particle Mesh Ewald method to calculate electrostatic interactions of two atoms at separations of >8.0 Å and additional conditions specified in Methods and Table S1.

Table S8D. Experimental and calculated *J*-coupling constants of Val3 in water at 300 K.

	EXPT[5]	FF12MC (4 µs^{lmt}) ORIG	SCHM	DFT1	DFT2	FF12SB (4 µs^{smt}) ORIG	SCHM	DFT1	DFT2	FF14SB (4 µs^{smt}) ORIG	SCHM	DFT1	DFT2
Residue 2 of Val3													
3JHnHa	7.94	6.82	6.15	6.69	7.36	6.03	5.30	5.66	6.34	6.00	5.25	5.61	6.34
3JHnC'	0.58	0.83	1.12	0.76	0.55	1.09	1.42	1.07	0.77	1.06	1.39	1.04	0.71
3JHaC'	2.42	1.95	1.98	1.85	2.08	1.79	1.77	1.60	1.79	1.66	1.66	1.47	1.66
3JC'C'	0.34	0.77	0.70	0.59	0.64	0.71	0.63	0.52	0.60	0.71	0.64	0.53	0.60
3JHnCb	1.38	1.76	1.84	3.42	2.65	1.93	2.00	3.73	2.95	1.95	2.03	3.81	3.01
1JNCa	10.80	10.58	10.58	10.58	10.58	11.13	11.13	11.13	11.13	11.08	11.08	11.08	11.08
2JNCa	8.35	8.52	8.52	8.52	8.52	8.47	8.47	8.47	8.47	8.50	8.50	8.50	8.50
3JHnCa	0.77	0.69	0.69	0.69	0.69	0.66	0.66	0.66	0.66	0.67	0.67	0.67	0.67
Residue 3 of Val3													
3JHnHa	7.91	7.71	7.10	7.85	8.26	6.18	5.65	5.99	6.21	6.12	5.45	5.81	6.37
3JHnC'	1.01	0.92	1.14	0.94	0.86	1.36	1.72	1.40	1.36	1.17	1.50	1.18	0.94
3JHaC'	2.45	2.23	2.30	2.22	2.47	2.86	2.65	2.62	2.82	2.03	1.97	1.84	2.03
3JHnCb	1.40	1.31	1.39	2.46	1.85	1.71	1.76	3.04	2.41	1.84	1.90	3.49	2.75
1JNCa	11.02	10.48	10.48	10.48	10.48	10.43	10.43	10.43	10.43	10.54	10.54	10.54	10.54
2JNCa	7.80	8.20	8.20	8.20	8.20	8.31	8.31	8.31	8.31	8.39	8.39	8.39	8.39
3JHnCa	0.75	0.67	0.67	0.67	0.67	0.61	0.61	0.61	0.61	0.63	0.63	0.63	0.63

Each *J*-coupling constant was calculated according to Eqs. S1–S8 with parameters in Table S9A and backbone torsions obtained from 20 unique and independent 200-million–timestep NPT MD simulations in water at 300 K using a cutoff of 8.0 Å for nonbonded interactions and the Particle Mesh Ewald method to calculate electrostatic interactions of two atoms at separations of >8.0 Å and additional conditions specified in Methods and Table S1.

Table S8E. Calculated *J*-coupling constants of Val3 in water at 300 K with different simulation times.

	EXPT[5]	FF14SB (4 µs^{smt}) ORIG	SCHM	DFT1	DFT2	FF14SB (6 µs^{smt}) ORIG	SCHM	DFT1	DFT2	FF14SB (8 µs^{smt}) ORIG	SCHM	DFT1	DFT2
Residue 2 of Val3													
3JHnHa	7.94	6.00	5.25	5.61	6.34	5.99	5.24	5.60	6.33	5.99	5.25	5.61	6.33
3JHnC'	0.58	1.06	1.39	1.04	0.71	1.07	1.39	1.04	0.71	1.07	1.39	1.04	0.71
3JHaC'	2.42	1.66	1.66	1.47	1.66	1.67	1.66	1.48	1.67	1.67	1.66	1.48	1.67
3JC'C'	0.34	0.71	0.64	0.53	0.60	0.71	0.63	0.52	0.60	0.71	0.63	0.52	0.60
3JHnCb	1.38	1.95	2.03	3.81	3.01	1.95	2.03	3.81	3.00	1.96	2.03	3.81	3.00
1JNCa	10.80	11.08	11.08	11.08	11.08	11.08	11.08	11.08	11.08	11.08	11.08	11.08	11.08
2JNCa	8.35	8.50	8.50	8.50	8.50	8.50	8.50	8.50	8.50	8.51	8.51	8.51	8.51
3JHnCa	0.77	0.67	0.67	0.67	0.67	0.67	0.67	0.67	0.67	0.67	0.67	0.67	0.67
Residue 3 of Val3													
3JHnHa	7.91	6.12	5.45	5.81	6.37	6.12	5.44	5.81	6.37	6.12	5.43	5.80	6.37
3JHnC'	1.01	1.17	1.50	1.18	0.94	1.17	1.50	1.17	0.93	1.17	1.50	1.17	0.93
3JHaC'	2.45	2.03	1.97	1.84	2.03	2.01	1.95	1.81	2.01	2.00	1.95	1.81	2.00
3JHnCb	1.40	1.84	1.90	3.49	2.75	1.84	1.91	3.50	2.76	1.84	1.91	3.51	2.77
1JNCa	11.02	10.54	10.54	10.54	10.54	10.54	10.54	10.54	10.54	10.54	10.54	10.54	10.54
2JNCa	7.80	8.39	8.39	8.39	8.39	8.40	8.40	8.40	8.40	8.39	8.39	8.39	8.39
3JHnCa	0.75	0.63	0.63	0.63	0.63	0.64	0.64	0.64	0.64	0.63	0.63	0.63	0.63

Each *J*-coupling constant was calculated according to Eqs. S1–S8 with parameters in Table S9A and backbone torsions obtained from 20 unique and independent 400-million–timestep NPT MD simulations in water at 300 K using a cutoff of 8.0 Å for nonbonded interactions and the Particle Mesh Ewald method to calculate electrostatic interactions of two atoms at separations of >8.0 Å and additional conditions specified in Methods and Table S1.

Table S8Fa. Calculated *J*-coupling constants of Val3 in water at 300 K with cutoff of 9.0 Å and different simulation times.

		FF12MC (4 μs[lmt])				FF12MC (6 μs[lmt])			
	EXPT[5]	ORIG	SCHM	DFT1	DFT2	ORIG	SCHM	DFT1	DFT2
					Residue 2 of Val3				
3JHnHa	7.94	6.83	6.16	6.70	7.35	6.83	6.16	6.71	7.36
3JHnC'	0.58	0.83	1.12	0.76	0.56	0.83	1.12	0.76	0.56
3JHaC'	2.42	1.99	2.01	1.89	2.12	1.99	2.01	1.89	2.12
3JC'C'	0.34	0.77	0.70	0.58	0.64	0.77	0.70	0.58	0.64
3JHnCb	1.38	1.76	1.83	3.40	2.64	1.76	1.83	3.40	2.64
1JNCa	10.80	10.58	10.58	10.58	10.58	10.57	10.57	10.57	10.57
2JNCa	8.35	8.52	8.52	8.52	8.52	8.52	8.52	8.52	8.52
3JHnCa	0.77	0.69	0.69	0.69	0.69	0.69	0.69	0.69	0.69
					Residue 3 of Val3				
3JHnHa	7.91	7.70	7.09	7.84	8.25	7.70	7.10	7.84	8.25
3JHnC'	1.01	0.93	1.15	0.95	0.87	0.93	1.15	0.94	0.87
3JHaC'	2.45	2.25	2.31	2.24	2.48	2.25	2.31	2.24	2.49
3JHnCb	1.40	1.31	1.38	2.45	1.85	1.31	1.38	2.45	1.85
1JNCa	11.02	10.48	10.48	10.48	10.48	10.48	10.48	10.48	10.48
2JNCa	7.80	8.20	8.20	8.20	8.20	8.20	8.20	8.20	8.20
3JHnCa	0.75	0.67	0.67	0.67	0.67	0.67	0.67	0.67	0.67

Each *J*-coupling constant was calculated according to Eqs. S1–S8 with parameters in Table S9A and backbone torsions obtained from 20 unique and independent 300-million–timestep NPT MD simulations in water at 300 K using a cutoff of 9.0 Å for nonbonded interactions and the Particle Mesh Ewald method to calculate electrostatic interactions of two atoms at separations of >9.0 Å and other conditions specified in Methods and Table S1.

Table S8Fb. Calculated *J*-coupling constants of Val3 in water at 300 K with cutoff of 9.0 Å and different simulation times.

		FF12MC (8 μs[lmt])				FF12MC (10 μs[lmt])			
	EXPT[5]	ORIG	SCHM	DFT1	DFT2	ORIG	SCHM	DFT1	DFT2
					Residue 2 of Val3				
3JHnHa	7.94	6.83	6.16	6.70	7.35	6.83	6.15	6.70	7.35
3JHnC'	0.58	0.83	1.12	0.76	0.56	0.83	1.12	0.76	0.56
3JHaC'	2.42	1.99	2.01	1.88	2.11	1.99	2.01	1.88	2.11
3JC'C'	0.34	0.77	0.70	0.58	0.64	0.77	0.70	0.58	0.64
3JHnCb	1.38	1.76	1.83	3.40	2.64	1.76	1.83	3.41	2.64
1JNCa	10.80	10.57	10.57	10.57	10.57	10.57	10.57	10.57	10.57
2JNCa	8.35	8.52	8.52	8.52	8.52	8.52	8.52	8.52	8.52
3JHnCa	0.77	0.69	0.69	0.69	0.69	0.69	0.69	0.69	0.69
					Residue 3 of Val3				
3JHnHa	7.91	7.70	7.09	7.84	8.25	7.70	7.09	7.84	8.25
3JHnC'	1.01	0.93	1.15	0.94	0.87	0.93	1.15	0.94	0.87
3JHaC'	2.45	2.24	2.31	2.23	2.48	2.24	2.31	2.23	2.48
3JHnCb	1.40	1.31	1.38	2.45	1.85	1.31	1.39	2.45	1.85
1JNCa	11.02	10.48	10.48	10.48	10.48	10.48	10.48	10.48	10.48
2JNCa	7.80	8.20	8.20	8.20	8.20	8.20	8.20	8.20	8.20
3JHnCa	0.75	0.67	0.67	0.67	0.67	0.67	0.67	0.67	0.67

Each *J*-coupling constant was calculated according to Eqs. S1–S8 with parameters in Table S9A and backbone torsions obtained from 20 unique and independent 500-million–timestep NPT MD simulations in water at 300 K using a cutoff of 9.0 Å for nonbonded interactions and the Particle Mesh Ewald method to calculate electrostatic interactions of two atoms at separations of >9.0 Å and other conditions specified in Methods and Table S1.

Table S8Ga. Calculated *J*-coupling constants of Val3 in water at 300 K with cutoff of 9.0 Å and different simulation times.

		FF14SB (4 μs^{smt})				FF14SB (6 μs^{smt})			
	EXPT[5]	ORIG	SCHM	DFT1	DFT2	ORIG	SCHM	DFT1	DFT2
				Residue 2 of Val3					
3JHnHa	7.94	6.03	5.29	5.65	6.37	6.03	5.29	5.65	6.37
3JHnC'	0.58	1.07	1.39	1.05	0.72	1.07	1.39	1.04	0.72
3JHaC'	2.42	1.69	1.68	1.50	1.69	1.69	1.69	1.51	1.70
3JC'C'	0.34	0.72	0.64	0.54	0.61	0.72	0.64	0.53	0.61
3JHnCb	1.38	1.94	2.01	3.78	2.98	1.94	2.01	3.78	2.98
1JNCa	10.80	11.08	11.08	11.08	11.08	11.08	11.08	11.08	11.08
2JNCa	8.35	8.49	8.49	8.49	8.49	8.49	8.49	8.49	8.49
3JHnCa	0.77	0.67	0.67	0.67	0.67	0.67	0.67	0.67	0.67
				Residue 3 of Val3					
3JHnHa	7.91	6.08	5.39	5.75	6.34	6.09	5.40	5.76	6.34
3JHnC'	1.01	1.16	1.49	1.16	0.90	1.16	1.50	1.17	0.92
3JHaC'	2.45	1.96	1.91	1.76	1.96	1.97	1.92	1.78	1.97
3JHnCb	1.40	1.86	1.93	3.56	2.81	1.86	1.92	3.54	2.80
1JNCa	11.02	10.54	10.54	10.54	10.54	10.54	10.54	10.54	10.54
2JNCa	7.80	8.38	8.38	8.38	8.38	8.37	8.37	8.37	8.37
3JHnCa	0.75	0.63	0.63	0.63	0.63	0.63	0.63	0.63	0.63

Each *J*-coupling constant was calculated according to Eqs. S1–S8 with parameters in Table S9A and backbone torsions obtained from 20 unique and independent 300-million–timestep NPT MD simulations in water at 300 K using a cutoff of 9.0 Å for nonbonded interactions and the Particle Mesh Ewald method to calculate electrostatic interactions of two atoms at separations of >9.0 Å and other conditions specified in Methods and Table S1.

Table S8Gb. Calculated *J*-coupling constants of Val3 in water at 300 K with cutoff of 9.0 Å and different simulation times.

		FF14SB (8 μs^{smt})				FF14SB (10 μs^{smt})			
	EXPT[5]	ORIG	SCHM	DFT1	DFT2	ORIG	SCHM	DFT1	DFT2
				Residue 2 of Val3					
3JHnHa	7.94	6.03	5.29	5.66	6.37	6.03	5.29	5.65	6.37
3JHnC'	0.58	1.07	1.39	1.04	0.73	1.07	1.39	1.04	0.72
3JHaC'	2.42	1.70	1.69	1.51	1.70	1.69	1.69	1.51	1.70
3JC'C'	0.34	0.72	0.64	0.53	0.61	0.72	0.64	0.53	0.61
3JHnCb	1.38	1.94	2.01	3.77	2.97	1.94	2.01	3.78	2.98
1JNCa	10.80	11.08	11.08	11.08	11.08	11.07	11.07	11.07	11.07
2JNCa	8.35	8.49	8.49	8.49	8.49	8.49	8.49	8.49	8.49
3JHnCa	0.77	0.67	0.67	0.67	0.67	0.67	0.67	0.67	0.67
				Residue 3 of Val3					
3JHnHa	7.91	6.08	5.40	5.76	6.33	6.10	5.42	5.78	6.34
3JHnC'	1.01	1.17	1.51	1.18	0.93	1.17	1.50	1.17	0.93
3JHaC'	2.45	2.00	1.94	1.80	1.99	2.02	1.96	1.82	2.01
3JHnCb	1.40	1.85	1.92	3.53	2.79	1.85	1.91	3.52	2.78
1JNCa	11.02	10.54	10.54	10.54	10.54	10.53	10.53	10.53	10.53
2JNCa	7.80	8.37	8.37	8.37	8.37	8.36	8.36	8.36	8.36
3JHnCa	0.75	0.63	0.63	0.63	0.63	0.63	0.63	0.63	0.63

Each *J*-coupling constant was calculated according to Eqs. S1–S8 with parameters in Table S9A and backbone torsions obtained from 20 unique and independent 300-million–timestep NPT MD simulations in water at 300 K using a cutoff of 9.0 Å for nonbonded interactions and the Particle Mesh Ewald method to calculate electrostatic interactions of two atoms at separations of >9.0 Å and other conditions specified in Methods and Table S1.

Table S9A. Different parameters of the Karplus equations S1–S5.

$J = A\cos^2(\theta+D) + B\cos(\theta+D) + C$	A (Hz)	B (Hz)	C (Hz)	D (°)
	Original [6-9]			
$^3J(H_N,H\alpha)$	7.09	–1.42	1.55	–60
$^3J(H_N,C')$	4.29	–1.01	0	180
$^3J(H\alpha,C')$	3.72	–2.18	1.28	120
$^3J(C',C')$	1.36	–0.93	0.60	0
$^3J(H_N,C\beta)$	3.06	–0.74	0.13	60
	Schmidt [7-10]			
$^3J(H_N,H\alpha)$	7.90	–1.05	0.65	–60
$^3J(H_N,C')$	4.41	–1.36	0.24	180
$^3J(H\alpha,C')$	3.76	–1.63	0.89	120
$^3J(C',C')$	1.51	–1.09	0.52	0
$^3J(H_N,C\beta)$	2.90	–0.56	0.18	60
	DFT1 [7-9,11]			
$^3J(H_N,H\alpha)$	9.44	–1.53	–0.07	–60
$^3J(H_N,C')$	5.58	–1.06	–0.30	180
$^3J(H\alpha,C')$	4.38	–1.87	0.56	120
$^3J(C',C')$	2.39	–1.25	0.26	0
$^3J(H_N,C\beta)$	5.15	0.01	–0.32	60
	DFT2 [7-9,11]			
$^3J(H_N,H\alpha)$	9.14	–2.28	–0.29	–64.51
$^3J(H_N,C')$	5.34	–1.46	–0.29	172.49
$^3J(H\alpha,C')$	4.77	–1.85	0.49	118.61
$^3J(C',C')$	2.71	–0.91	0.21	–2.56
$^3J(H_N,C\beta)$	4.58	–0.36	–0.31	58.18

Table S9B. Different parameters of the Karplus equations S9–S20.

$J = A\cos^2(\chi_i+D) + B\cos(\chi_i+D) + C$	A (Hz)	B (Hz)	C (Hz)	D (°)
	Original [12]			
$^3J(H\alpha,H\beta)$	3.47	– 1.61	4.33	0
$^3J(N',H\beta)$	2.21	– 0.33	1.05	0
$^3J(C',H\beta)$	2.81	– 0.97	1.60	0
$^3J(H\alpha,C\gamma)$	2.70	– 0.79	1.77	0
$^3J(N',C\gamma)$	0.86	– 0.41	0.59	0
$^3J(C',C\gamma)$	1.25	– 0.74	1.36	0
	Schmidt [12]			
$^3J(H\alpha,H\beta)$	7.23	– 1.37	2.22	0
$^3J(N',H\beta)$	2.30	– 0.75	1.07	0
$^3J(C',H\beta)$	4.02	– 1.58	1.32	0
$^3J(H\alpha,C\gamma)$	5.34	– 0.96	0.79	0
$^3J(N',C\gamma)$	1.29	– 0.49	0.37	0
$^3J(C',C\gamma)$	2.31	– 0.87	0.55	0
	Best-Fit [13]			
$^3J(N,C\gamma,Thr)$	2.01	0.21	– 0.12	7
$^3J(C',C\gamma,Thr)$	2.76	– 0.67	0.19	17
$^3J(N,C\gamma,Val/Ile)$	2.64	0.26	– 0.22	6
$^3J(C',C\gamma, Val/Ile)$	3.42	– 0.59	0.17	5
	DFT [13]			
$^3J(N,C\gamma,Thr)$	2.12	0.23	– 0.15	13
$^3J(C',C\gamma,Thr)$	2.97	– 0.83	– 0.02	25
$^3J(N,C\gamma1,Val/Ile)$	2.22	0.15	– 0.06	3
$^3J(N,C\gamma2,Val/Ile)$	2.24	0.15	– 0.03	– 9
$^3J(C',C\gamma1, Val/Ile)$	3.31	– 0.91	0.01	16
$^3J(C',C\gamma2, Val/Ile)$	3.30	– 0.51	0.04	4

Table S10. Effects of the van der Waals interaction cutoff and simulation time on mean square deviation (χ^2) between experimental and calculated *J*-coupling constants of Val3.

Forcefield	Cutoff (Å)	AggTime (μs^{smt})	χ^2 for Val3 (mean ± standard error)			
			Original	*Schmidt*	*DFT1*	*DFT2*
FF14SB	8.0	4.00	1.74±0.03	2.38±0.03	6.35±0.10	3.17±0.05
FF14SB	8.0	6.00	1.72±0.02	2.38±0.02	6.36±0.08	3.16±0.04
FF14SB	8.0	8.00	1.72±0.02	2.38±0.03	6.37±0.08	3.16±0.04
FF14SB	9.0	4.00	1.75±0.03	2.41±0.04	6.42±0.10	3.20±0.06
FF14SB	9.0	6.00	1.72±0.03	2.38±0.03	6.36±0.09	3.16±0.05
FF14SB	9.0	8.00	1.71±0.03	2.37±0.03	6.31±0.09	3.14±0.05
FF14SB	9.0	10.00	1.70±002	2.35±0.03	6.27±0.08	3.11±0.04
FF12MC	8.0	6.32	0.76±0.01	0.95±0.01	2.90±0.02	1.22±0.01
FF12MC	8.0	12.64	0.76±0.00	0.95±0.00	2.90±0.01	1.22±0.01
FF12MC	9.0	6.32	0.75±0.01	0.94±0.01	2.86±0.02	1.20±0.01
FF12MC	9.0	12.64	0.72±0.00	0.86±0.00	2.82±0.01	1.17±0.00
FF12MC	9.0	18.96	0.74±0.00	0.93±0.00	2.84±0.01	1.20±0.00
FF12MC	9.0	25.28	0.75±0.00	0.93±0.00	2.85±0.01	1.20±0.00
FF12MC	9.0	31.60	0.75±0.00	0.93±0.00	2.85±0.01	1.20±0.00

Cutoff: Cutoff for nonbonded interaction. AggTime: Aggregated simulation time. ^{smt}: Standard-mass time. The experimental and calculated *J*-coupling constants are listed in Tables S8*D*–*G*. The mean and standard error of each χ^2 or RMSD were obtained from 20 distinct and independent NPT MD simulations in water at Δt = 1.00 fs^{smt}, 1 atm, and 300 K.

Table S11A. Experimental and calculated *J*-coupling constants of GB3 in water at 298 K.

		FF12MC (12.64 μs[smt])				FF14SB (12.64 μs[smt])			
	EXPT[14]	ORIG	SCHM	BFIT	DFT	ORIG	SCHM	BFIT	DFT
				Residue 3					
3JHnHa	9.80	7.87	7.24	8.04	8.72	9.30	8.78	9.91	10.02
3JHnC'	0.75	0.45	0.69	0.30	0.21	0.91	1.01	1.00	1.13
3JHnCb	0.74	1.57	1.65	3.08	2.33	0.62	0.71	1.08	0.68
3JHaHb2	11.81	9.03	10.13	10.13	10.13	9.31	10.64	10.64	10.64
3JHaHb3	1.95	4.94	4.84	4.84	4.84	4.50	3.61	3.61	3.61
				Residue 4					
3JHnHa	9.80	9.44	8.93	10.10	10.43	9.60	9.10	10.30	10.36
3JHnC'	0.22	0.48	0.62	0.43	0.56	0.89	0.98	1.00	1.17
3JHnCb	1.16	0.85	0.95	1.62	1.09	0.50	0.59	0.83	0.47
				Residue 5					
3JHnHa	9.60	9.45	8.94	10.10	10.41	9.72	9.23	10.45	10.57
3JHnC'	0.71	0.52	0.65	0.47	0.61	0.76	0.86	0.83	1.01
3JHnCb	0.57	0.83	0.92	1.57	1.05	0.53	0.63	0.93	0.54
				Residue 6					
3JHnHa	9.80	8.85	8.29	9.32	9.86	9.08	8.55	9.62	10.15
3JHnC'	0.22	0.32	0.50	0.18	0.22	0.27	0.44	0.12	0.19
3JHnCb	1.10	1.23	1.32	2.40	1.74	1.16	1.25	2.28	1.63
3JNCg1	1.99	1.68	1.91	1.74	1.74	1.80	2.06	1.92	1.92
3JNCg2	0.53	0.71	0.60	0.57	0.57	0.64	0.51	0.34	0.34
3JC'Cg1	0.77	1.50	1.00	0.49	0.49	1.40	0.90	0.22	0.22
3JC'Cg2	3.68	1.39	0.84	3.32	3.32	1.32	0.68	3.67	3.67
				Residue 7					
3JHnHa	9.60	8.53	7.96	8.91	9.53	9.22	8.69	9.80	10.27
3JHnC'	0.23	0.31	0.50	0.14	0.13	0.32	0.48	0.19	0.28
3JHnCb	1.29	1.38	1.47	2.71	2.00	1.07	1.16	2.08	1.47
3JNCg2	1.97	0.82	0.77	1.49	1.49	0.62	0.47	1.93	1.93
3JC'Cg2	0.58	2.68	2.71	1.01	1.01	3.28	3.62	0.63	0.63
				Residue 8					
3JHnHa	9.90	8.93	8.38	9.42	9.72	9.15	8.62	9.71	9.78
3JHnC'	0.66	0.72	0.86	0.73	0.79	1.02	1.12	1.15	1.26
3JHnCb	0.87	0.91	1.00	1.69	1.18	0.61	0.69	1.00	0.64
3JHaHb2	6.09	4.66	3.54	3.54	3.54	6.38	6.14	6.14	6.14
3JHaHb3	7.07	4.68	4.04	4.04	4.04	4.62	3.92	3.92	3.92
				Residue 10					
3JHnHa	5.00	4.95	4.24	4.33	4.87	5.11	4.30	4.45	5.33
3JHnC'	0.94	1.55	1.97	1.61	1.29	1.16	1.55	1.12	0.67
3JHnCb	2.87	2.09	2.15	3.98	3.19	2.27	2.35	4.49	3.58
				Residue 11					
3JHnHa	10.30	7.69	7.07	7.82	8.46	8.96	8.42	9.46	9.52
3JHnC'	0.35	0.54	0.78	0.40	0.31	1.11	1.22	1.28	1.35
3JHnCb	0.89	1.59	1.67	3.09	2.35	0.62	0.70	1.02	0.66
3JNCgT	0.90	1.16	1.18	0.88	0.88	1.08	1.09	1.04	1.04
3JC'CgT	2.11	2.20	1.96	1.90	1.90	2.26	2.10	1.80	1.80
				Residue 12					
3JHnHa	7.80	7.17	6.50	7.14	7.79	7.49	6.86	7.56	7.93
3JHnC'	0.82	0.74	1.00	0.66	0.48	1.04	1.26	1.10	0.98
3JHnCb	1.42	1.67	1.75	3.24	2.50	1.32	1.39	2.42	1.85
				Residue 13					
3JHnHa	9.90	8.94	8.42	9.45	9.58	8.61	8.05	9.01	8.94
3JHnC'	1.06	0.94	1.08	1.03	1.14	1.43	1.52	1.71	1.73
3JHnCb	0.46	0.76	0.84	1.31	0.89	0.55	0.62	0.79	0.51
				Residue 15					
3JHnHa	8.40	8.33	7.75	8.65	8.43	8.16	7.55	8.42	7.99
3JHnC'	1.67	1.70	1.77	2.07	2.06	2.11	2.15	2.65	2.61
3JHnCb	0.35	0.49	0.55	0.53	0.35	0.27	0.32	-0.01	-0.05
				Residue 16					
3JHnHa	6.80	8.44	7.85	8.78	8.57	7.68	7.04	7.79	7.25
3JHnC'	2.61	1.67	1.74	2.05	2.05	2.42	2.45	3.07	2.95
3JHnCb	0.19	0.45	0.52	0.49	0.30	0.26	0.30	-0.15	-0.12
3JNCgT	0.21	1.05	1.04	0.62	0.62	0.59	0.43	0.04	0.04
3JC'CgT	1.78	1.48	0.97	1.04	1.04	1.35	0.77	1.31	1.31
				Residue 17					
3JHnHa	9.00	9.38	8.87	10.02	10.23	9.27	8.74	9.86	9.75
3JHnC'	1.52	0.71	0.83	0.74	0.87	1.28	1.35	1.52	1.64
3JHnCb	0.17	0.72	0.81	1.30	0.85	0.37	0.45	0.48	0.22
3JNCgT	0.56	1.15	1.17	0.84	0.84	0.78	0.68	0.62	0.62

3JC'CgT	2.80	2.16	1.91	1.91	1.91	2.10	1.87	1.91	1.91
Residue 18									
3JHnHa	6.70	8.90	8.35	9.39	9.19	8.05	7.43	8.28	7.78
3JHnC'	2.69	1.53	1.59	1.86	1.93	2.26	2.29	2.85	2.80
3JHnCb	0.21	0.35	0.43	0.36	0.16	0.21	0.26	-0.17	-0.17
3JC'CgT	1.69	1.56	1.24	1.41	1.41	1.46	1.08	1.68	1.68
Residue 19									
3JHnHa	9.40	7.34	6.68	7.36	8.18	8.53	7.95	8.90	9.54
3JHnC'	0.53	0.39	0.66	0.19	0.02	0.28	0.48	0.10	0.09
3JHnCb	1.19	1.84	1.92	3.64	2.80	1.40	1.49	2.75	2.03
Residue 20									
3JHnHa	7.30	8.26	7.66	8.55	8.22	7.57	6.92	7.64	7.17
3JHnC'	2.20	1.92	1.97	2.38	2.35	2.36	2.41	2.97	2.84
3JHnCb	0.10	0.36	0.42	0.22	0.11	0.35	0.39	0.07	0.06
Residue 21									
3JHnHa	4.30	7.49	6.84	7.55	8.18	5.67	4.89	5.18	6.09
3JHnC'	1.81	0.69	0.95	0.61	0.47	0.89	1.25	0.78	0.39
3JHnCb	2.72	1.56	1.64	3.06	2.33	2.22	2.30	4.39	3.47
3JNCg1	0.69	1.16	1.19	1.08	1.08	0.85	0.77	0.71	0.71
3JNCg2	1.08	1.14	1.18	0.94	0.94	1.01	1.02	1.34	1.34
3JC'Cg1	2.52	2.13	1.92	1.96	1.96	1.89	1.58	1.77	1.77
3JC'Cg2	1.09	1.57	1.16	1.90	1.90	2.32	2.21	1.13	1.13
Residue 22									
3JHnHa	6.30	7.85	7.22	8.01	8.27	7.68	7.03	7.78	7.28
3JHnC'	2.79	1.13	1.29	1.25	1.16	2.37	2.40	3.00	2.88
3JHnCb	0.22	1.10	1.17	1.94	1.46	0.30	0.34	-0.06	-0.04
3JHaHb2	3.99	5.12	5.18	5.18	5.18	4.57	3.73	3.73	3.73
3JHaHb3	2.13	5.46	4.58	4.58	4.58	8.80	9.85	9.85	9.85
Residue 23									
3JHnHa	3.10	3.68	2.79	2.61	3.36	4.80	3.96	4.04	4.96
3JHnC'	2.10	1.95	2.42	2.09	1.52	1.21	1.62	1.17	0.68
3JHnCb	3.34	2.35	2.42	4.64	3.76	2.37	2.44	4.69	3.75
Residue 24									
3JHnHa	4.00	4.32	3.46	3.44	4.28	5.17	4.36	4.53	5.46
3JHnC'	1.53	1.52	1.96	1.56	1.04	1.04	1.43	0.96	0.51
3JHnCb	3.19	2.36	2.43	4.67	3.75	2.33	2.40	4.61	3.67
Residue 25									
3JHnHa	5.40	3.89	3.01	2.88	3.67	5.39	4.59	4.81	5.75
3JHnC'	0.74	1.80	2.26	1.91	1.34	0.95	1.32	0.84	0.42
3JHnCb	3.35	2.36	2.43	4.66	3.76	2.30	2.38	4.55	3.62
3JNCgT	1.75	1.58	1.77	1.28	1.28	1.74	1.99	1.46	1.46
3JC'CgT	2.60	1.64	1.18	0.69	0.69	1.44	0.89	0.24	0.24
Residue 26									
3JHnHa	4.40	3.68	2.79	2.60	3.38	4.63	3.79	3.83	4.73
3JHnC'	1.19	1.91	2.38	2.05	1.46	1.30	1.72	1.29	0.78
3JHnCb	3.35	2.37	2.44	4.69	3.79	2.38	2.45	4.71	3.78
Residue 28									
3JHnHa	3.70	3.22	2.31	2.02	2.71	4.40	3.55	3.54	4.42
3JHnC'	1.84	2.25	2.75	2.47	1.86	1.44	1.86	1.45	0.92
3JHnCb	2.91	2.33	2.41	4.62	3.75	2.39	2.46	4.72	3.79
Residue 29									
3JHnHa	4.80	3.70	2.81	2.63	3.40	4.80	3.96	4.05	4.96
3JHnC'	0.81	1.91	2.38	2.05	1.46	1.23	1.63	1.19	0.70
3JHnCb	3.28	2.36	2.44	4.68	3.78	2.36	2.44	4.67	3.74
Residue 30									
3JHnHa	4.80	4.08	3.21	3.13	3.96	5.36	4.56	4.77	5.72
3JHnC'	0.67	1.65	2.10	1.72	1.17	0.94	1.32	0.84	0.41
3JHnCb	2.96	2.38	2.45	4.70	3.79	2.32	2.39	4.59	3.65
3JHaHb2	11.33	8.96	10.03	10.03	10.03	9.21	10.45	10.45	10.45
3JHaHb3	0.75	5.07	5.20	5.20	5.20	4.90	4.76	4.76	4.76
Residue 31									
3JHnHa	4.10	3.53	2.63	2.42	3.16	4.90	4.07	4.18	5.11
3JHnC'	1.17	2.03	2.51	2.20	1.61	1.16	1.56	1.11	0.62
3JHnCb	2.87	2.35	2.43	4.66	3.77	2.36	2.44	4.67	3.74
Residue 32									
3JHnHa	5.30	3.48	2.58	2.35	3.08	4.72	3.88	3.94	4.84
3JHnC'	0.95	2.06	2.54	2.23	1.64	1.26	1.68	1.24	0.74
3JHnCb	3.18	2.36	2.43	4.66	3.78	2.37	2.44	4.69	3.76
Residue 33									
3JHnHa	3.20	3.43	2.53	2.28	3.01	4.22	3.35	3.30	4.16
3JHnC'	1.58	2.10	2.59	2.29	1.69	1.55	1.98	1.59	1.04
3JHnCb	3.45	2.35	2.42	4.64	3.76	2.39	2.46	4.73	3.81
Residue 34									
3JHnHa	3.70	3.57	2.68	2.47	3.22	4.61	3.76	3.80	4.71
3JHnC'	1.27	2.00	2.48	2.16	1.57	1.31	1.73	1.30	0.79

3JHnCb	3.49	2.36	2.43	4.66	3.77	2.39	2.46	4.71	3.78
Residue 35									
3JHaHb2	7.92	4.77	4.01	4.01	4.01	6.43	6.29	6.29	6.29
3JHaHb3	7.15	4.63	3.71	3.71	3.71	4.67	4.07	4.07	4.07
Residue 36									
3JHnHa	3.70	4.22	3.36	3.30	4.12	5.05	4.24	4.38	5.29
3JHnC'	2.11	1.62	2.06	1.68	1.15	1.12	1.51	1.06	0.59
3JHnCb	2.38	2.34	2.42	4.63	3.73	2.33	2.40	4.60	3.67
Residue 37									
3JHnHa	9.30	4.93	4.11	4.23	5.10	8.36	7.77	8.68	9.31
3JHnC'	0.02	1.23	1.64	1.21	0.74	0.36	0.57	0.20	0.17
3JHnCb	1.59	2.30	2.37	4.55	3.63	1.41	1.50	2.77	2.06
3JHaHb2	12.27	5.51	4.69	4.69	4.69	9.03	10.16	10.16	10.16
3JHaHb3	1.94	4.98	4.85	4.85	4.85	4.85	4.61	4.61	4.61
Residue 39									
3JHnHa	9.00	9.19	8.66	9.76	9.76	7.44	6.78	7.48	8.20
3JHnC'	0.19	1.13	1.22	1.31	1.42	0.54	0.80	0.40	0.24
3JHnCb	1.53	0.51	0.59	0.78	0.47	1.69	1.78	3.33	2.55
3JNCg1	1.86	1.48	1.63	1.46	1.46	1.59	1.78	1.67	1.67
3JNCg2	0.58	0.81	0.72	0.83	0.83	0.78	0.69	0.51	0.51
3JC'Cg1	0.86	1.64	1.18	0.88	0.88	1.61	1.20	0.73	0.73
3JC'Cg2	3.27	1.56	1.14	2.75	2.75	1.45	0.89	3.12	3.12
Residue 40									
3JHnHa	8.40	8.86	8.31	9.33	9.33	8.12	7.51	8.37	8.23
3JHnC'	1.80	1.22	1.31	1.42	1.48	1.69	1.79	2.06	2.01
3JHnCb	0.36	0.59	0.67	0.91	0.59	0.58	0.64	0.76	0.53
Residue 42									
3JHnHa	8.70	7.50	6.85	7.56	8.36	6.96	6.27	6.86	7.74
3JHnC'	0.07	0.39	0.66	0.20	0.05	0.43	0.72	0.23	-0.01
3JHnCb	1.69	1.77	1.85	3.49	2.67	1.98	2.06	3.92	3.04
3JNCg1	1.73	1.23	1.28	1.17	1.17	1.40	1.52	1.43	1.43
3JNCg2	0.74	1.08	1.10	0.87	0.87	0.82	0.75	0.72	0.72
3JC'Cg1	1.27	2.05	1.81	1.75	1.75	1.64	1.25	0.92	0.92
3JC'Cg2	3.04	1.56	1.11	2.11	2.11	1.70	1.26	2.63	2.63
Residue 43									
3JHnHa	8.70	8.42	7.84	8.76	9.47	8.49	7.91	8.85	9.55
3JHnC'	-0.05	0.19	0.41	-0.02	-0.05	0.18	0.39	-0.04	-0.06
3JHnCb	1.74	1.50	1.59	2.98	2.22	1.49	1.58	2.94	2.19
3JHaHb2	11.13	8.77	9.69	9.69	9.69	9.18	10.39	10.39	10.39
3JHaHb3	1.78	5.12	5.31	5.31	5.31	4.89	4.72	4.72	4.72
Residue 44									
3JHnHa	8.90	9.26	8.74	9.85	9.91	8.23	7.63	8.51	8.07
3JHnC'	1.66	1.00	1.10	1.14	1.26	2.13	2.16	2.67	2.64
3JHnCb	0.14	0.57	0.65	0.93	0.57	0.23	0.28	-0.09	-0.13
3JNCgT	0.47	0.96	0.91	0.76	0.76	0.69	0.55	0.43	0.43
3JC'CgT	2.88	2.47	2.38	2.40	2.40	2.06	1.82	2.03	2.03
Residue 45									
3JHnHa	8.80	8.87	8.32	9.35	9.47	8.84	8.29	9.31	8.98
3JHnC'	1.17	1.01	1.12	1.14	1.19	1.76	1.81	2.18	2.25
3JHnCb	0.27	0.73	0.81	1.24	0.84	0.21	0.28	0.03	-0.09
3JHaHb2	4.41	4.58	3.86	3.86	3.86	4.50	3.61	3.61	3.61
3JHaHb3	11.18	4.30	2.88	2.88	2.88	4.31	2.97	2.97	2.97
Residue 46									
3JHnHa	9.80	9.20	8.67	9.78	10.20	9.36	8.85	9.99	9.91
3JHnC'	0.69	0.42	0.57	0.32	0.42	1.20	1.27	1.41	1.55
3JHnCb	0.79	1.01	1.10	1.94	1.36	0.38	0.47	0.53	0.25
3JHaHb2	3.41	4.51	3.59	3.59	3.59	4.62	3.88	3.88	3.88
3JHaHb3	11.81	4.40	3.25	3.25	3.25	4.32	3.02	3.02	3.02
Residue 47									
3JHnHa	4.10	4.88	4.08	4.18	5.02	5.40	4.60	4.83	5.76
3JHnC'	1.44	1.24	1.64	1.21	0.76	0.95	1.33	0.85	0.43
3JHnCb	2.89	2.32	2.39	4.55	3.64	2.29	2.37	4.54	3.61
3JHaHb2	3.58	4.37	2.99	2.99	2.99	4.36	2.92	2.92	2.92
3JHaHb3	11.10	4.73	4.20	4.20	4.20	4.90	4.49	4.49	4.49
Residue 48									
3JHnHa	4.30	3.44	2.55	2.31	3.00	4.94	4.12	4.24	5.15
3JHnC'	1.48	2.15	2.64	2.35	1.77	1.16	1.56	1.11	0.64
3JHnCb	2.81	2.31	2.38	4.56	3.70	2.34	2.42	4.64	3.70
Residue 49									
3JHnHa	10.00	7.44	6.78	7.48	8.33	9.33	8.81	9.94	10.24
3JHnC'	0.03	0.32	0.59	0.11	-0.06	0.58	0.71	0.55	0.67
3JHnCb	1.45	1.84	1.93	3.65	2.80	0.84	0.93	1.58	1.07
3JNCgT	0.77	0.63	0.49	0.94	0.94	0.62	0.48	1.00	1.00
3JC'CgT	3.05	3.21	3.50	3.40	3.40	3.27	3.61	3.36	3.36
Residue 50									

3JHnHa	7.00	6.91	7.11	7.39	6.23	6.98	7.22	7.52	6.06
3JHnC'	3.02	1.42	1.85	1.44	2.26	2.25	2.75	2.47	3.44
3JHnCb	0.95	1.50	1.41	1.38	1.25	0.88	0.84	0.48	0.43
				Residue 51					
3JHnHa	10.30	9.63	9.14	10.34	10.37	9.66	9.17	10.37	10.61
3JHnC'	0.35	0.94	1.02	1.06	1.24	0.57	0.69	0.56	0.73
3JHnCb	0.78	0.45	0.54	0.73	0.38	0.70	0.79	1.30	0.83
3JNCgT	1.61	1.81	2.08	1.47	1.47	1.84	2.12	1.60	1.60
3JC'CgT	0.52	1.33	0.71	0.05	0.05	1.36	0.82	-0.02	-0.02
				Residue 52					
3JHnHa	9.40	7.65	7.01	7.75	8.60	7.25	6.58	7.23	8.14
3JHnC'	0.14	0.23	0.49	-0.01	-0.15	0.28	0.56	0.03	-0.17
3JHnCb	1.55	1.81	1.90	3.60	2.75	1.96	2.04	3.89	3.00
				Residue 53					
3JHnHa	9.70	9.03	8.49	9.56	9.83	9.36	8.85	9.99	9.80
3JHnC'	1.10	0.72	0.86	0.74	0.82	1.39	1.45	1.69	1.83
3JHnCb	0.30	0.86	0.95	1.59	1.09	0.25	0.33	0.22	0.01
3JNCgT	1.57	1.69	1.91	1.35	1.35	1.81	2.08	1.53	1.53
3JC'CgT	0.45	1.48	0.93	0.42	0.42	1.33	0.74	0.02	0.02
				Residue 54					
3JHnHa	10.00	9.45	8.94	10.10	10.48	9.67	9.18	10.39	10.53
3JHnC'	1.08	0.42	0.55	0.33	0.47	0.74	0.84	0.79	0.96
3JHnCb	0.22	0.90	0.99	1.73	1.17	0.57	0.67	1.02	0.61
3JNCg1	0.83	1.19	1.25	1.17	1.17	0.67	0.54	0.46	0.46
3JNCg2	1.85	0.63	0.49	1.35	1.35	0.67	0.57	1.90	1.90
3JC'Cg1	0.70	1.33	0.68	0.48	0.48	1.32	0.71	0.96	0.96
3JC'Cg2	1.01	2.34	2.27	2.04	2.04	3.16	3.45	0.66	0.66
				Residue 55					
3JHnHa	9.90	9.13	8.60	9.69	10.13	9.33	8.82	9.95	9.93
3JHnC'	0.26	0.40	0.55	0.29	0.37	1.11	1.20	1.30	1.43
3JHnCb	0.98	1.05	1.14	2.04	1.44	0.46	0.54	0.69	0.38
3JNCgT	1.51	1.66	1.87	1.34	1.34	1.61	1.81	1.30	1.30
				Residue 56					
3JHnHa	8.30	6.72	6.02	6.55	7.38	6.95	6.26	6.85	7.58
3JHnC'	0.23	0.62	0.92	0.47	0.22	0.68	0.96	0.57	0.35
3JHnCb	2.25	1.95	2.03	3.85	3.00	1.80	1.88	3.53	2.73

Each *J*-coupling constant was calculated according to Eqs. S9–S20 with parameters in Table S9B and torsions obtained from 20 unique and independent 316-ns^{smt} NPT MD simulations in water at 298 K using a cutoff of 8.0 Å for nonbonded interactions and the Particle Mesh Ewald method to calculate electrostatic interactions of two atoms at separations of >8.0 Å and other conditions specified in Methods and Table S1.

Table S11B. Experimental and calculated *J*-coupling constants of BPTI in water at 309 K.

		FF12MC (12.64 μs^{smt})				FF14SB (12.64 μs^{smt})			
	EXPT[15]	ORIG	SCHM	BFIT	DFT	ORIG	SCHM	BFIT	DFT
				Residue 4					
3JHaHb2	4.50	6.52	6.42	6.42	6.42	4.61	3.88	3.88	3.88
3JHaHb3	4.50	5.72	5.75	5.75	5.75	9.19	10.45	10.45	10.45
				Residue 5					
3JHaHb2	12.00	5.85	5.59	5.59	5.59	9.11	10.28	10.28	10.28
3JHaHb3	3.00	5.81	5.55	5.55	5.55	4.74	4.28	4.28	4.28
				Residue 11					
3JHaHb2	8.20	6.19	8.08	8.08	8.08	6.19	8.08	8.08	8.08
				Residue 14					
3JHaHb2	12.00	6.26	7.71	7.71	7.71	9.13	10.32	10.32	10.32
3JHaHb3	3.00	5.82	4.79	4.79	4.79	4.80	4.47	4.47	4.47
				Residue 15					
3JHaHb2	11.00	6.19	8.08	8.08	8.08	7.86	8.45	8.45	8.45
3JHaHb3	3.50	6.00	4.71	4.71	4.71	5.33	4.98	4.98	4.98
				Residue 18					
3JHaHb2	10.50	6.19	8.08	8.08	8.08	6.19	8.08	8.08	8.08
				Residue 19					
3JHaHb2	11.00	6.19	8.08	8.08	8.08	6.19	8.08	8.08	8.08
				Residue 20					

3JHaHb2	12.50	6.19	8.08	8.08	8.08	9.01	10.12	10.12	10.12
3JHaHb3	2.50	6.00	4.71	4.71	4.71	4.76	4.37	4.37	4.37
				Residue 22					
3JHaHb2	4.40	6.19	8.08	8.08	8.08	5.20	5.59	5.59	5.59
3JHaHb3	3.50	6.00	4.71	4.71	4.71	8.99	10.04	10.04	10.04
				Residue 23					
3JHaHb2	3.80	6.19	8.08	8.08	8.08	4.34	3.09	3.09	3.09
3JHaHb3	12.50	6.00	4.71	4.71	4.71	4.46	3.51	3.51	3.51
				Residue 24					
3JHaHb2	3.50	6.19	8.08	8.08	8.08	4.37	3.20	3.20	3.20
3JHaHb3	12.00	6.00	4.71	4.71	4.71	4.46	3.49	3.49	3.49
				Residue 30					
3JHaHb2	12.00	6.19	8.08	8.08	8.08	9.17	10.38	10.38	10.38
3JHaHb3	2.50	6.00	4.71	4.71	4.71	4.85	4.61	4.61	4.61
				Residue 31					
3JHaHb2	11.00	6.19	8.08	8.08	8.08	8.67	9.65	9.65	9.65
3JHaHb3	3.50	6.00	4.71	4.71	4.71	4.85	4.42	4.42	4.42
				Residue 32					
3JHaHb2	2.50	6.19	8.08	8.08	8.08	6.19	8.08	8.08	8.08
				Residue 33					
3JHaHb2	4.50	6.19	8.08	8.08	8.08	5.23	5.65	5.65	5.65
3JHaHb3	3.00	6.00	4.71	4.71	4.71	8.92	9.93	9.93	9.93
				Residue 34					
3JHaHb2	10.50	6.19	8.08	8.08	8.08	6.19	8.08	8.08	8.08
				Residue 35					
3JHaHb2	6.50	6.19	8.08	8.08	8.08	4.71	4.24	4.24	4.24
3JHaHb3	11.50	6.00	4.71	4.71	4.71	4.31	2.74	2.74	2.74
				Residue 38					
3JHaHb2	6.50	6.19	8.08	8.08	8.08	4.80	4.35	4.35	4.35
3JHaHb3	1.50	6.00	4.71	4.71	4.71	9.16	10.41	10.41	10.41
				Residue 41					
3JHaHb2	12.50	6.19	8.08	8.08	8.08	9.14	10.35	10.35	10.35
3JHaHb3	2.50	6.00	4.71	4.71	4.71	4.70	4.16	4.16	4.16
				Residue 43					
3JHaHb2	3.00	6.19	8.08	8.08	8.08	4.25	2.61	2.61	2.61
3JHaHb3	12.00	6.00	4.71	4.71	4.71	4.82	4.54	4.54	4.54
				Residue 44					
3JHaHb2	4.00	6.19	8.08	8.08	8.08	4.50	3.43	3.43	3.43
3JHaHb3	11.00	6.00	4.71	4.71	4.71	4.54	3.57	3.57	3.57
				Residue 45					
3JHaHb2	11.80	6.19	8.08	8.08	8.08	9.22	10.48	10.48	10.48
3JHaHb3	4.00	6.00	4.71	4.71	4.71	4.37	3.19	3.19	3.19
				Residue 46					
3JHaHb2	9.50	6.19	8.08	8.08	8.08	6.04	5.79	5.79	5.79
3JHaHb3	4.00	6.00	4.71	4.71	4.71	5.82	5.67	5.67	5.67
				Residue 47					
3JHaHb2	3.00	6.19	8.08	8.08	8.08	4.76	4.34	4.34	4.34
3JHaHb3	2.80	6.00	4.71	4.71	4.71	8.09	8.73	8.73	8.73
				Residue 51					
3JHaHb2	5.50	6.19	8.08	8.08	8.08	4.60	3.90	3.90	3.90
3JHaHb3	11.50	6.00	4.71	4.71	4.71	4.29	2.84	2.84	2.84
				Residue 52					
3JHaHb2	3.00	6.19	8.08	8.08	8.08	6.13	5.89	5.89	5.89
3JHaHb3	10.20	6.00	4.71	4.71	4.71	4.69	4.01	4.01	4.01
				Residue 54					
3JHaHb2	10.00	6.19	8.08	8.08	8.08	6.19	8.08	8.08	8.08
				Residue 55					
3JHaHb2	11.00	6.19	8.08	8.08	8.08	9.21	10.46	10.46	10.46
3JHaHb3	1.50	6.00	4.71	4.71	4.71	4.75	4.33	4.33	4.33

Each *J*-coupling constant was calculated according to Eqs. S9–S20 with parameters in Table S9B and torsions obtained from 20 unique and independent 316-ns^{smt} NPT MD simulations in water at 309 K using a cutoff of 8.0 Å for nonbonded interactions and the Particle Mesh Ewald method to calculate electrostatic interactions of two atoms at separations of >8.0 Å and other conditions specified in Methods and Table S1.

Table S11C. Experimental and calculated *J*-coupling constants of ubiquitin in water at 303 K.

		FF12MC (12.64 μs^{smt})				FF14SB (12.64 μs^{smt})			
	EXPT[14]	ORIG	SCHM	BFIT	DFT	ORIG	SCHM	BFIT	DFT
Residue 2									
3JC'Cg	2.40	2.25	2.03	2.03	2.03	1.61	1.16	1.16	1.16
Residue 3									
3JHnHa	9.27	9.05	8.51	9.58	10.01	9.15	8.61	9.71	9.43
3JHnC'	1.88	0.46	0.62	0.38	0.46	1.59	1.64	1.95	2.07
3JHaC'	2.98	2.50	2.65	2.64	2.95	2.52	2.68	2.67	2.92
3JHnCb	-0.24	1.04	1.13	2.00	1.41	0.20	0.28	0.07	-0.09
3JNCg2	0.39	0.72	0.63	0.39	0.39	0.80	0.70	0.65	0.65
3JC'Cg2	3.68	1.52	0.91	3.18	3.18	1.80	1.35	2.72	2.72
Residue 4									
3JHnHa	10.04	8.46	7.88	8.81	9.48	9.66	9.17	10.38	10.64
3JHnC'	-0.13	0.25	0.46	0.06	0.04	0.54	0.65	0.50	0.68
3JHaC'	3.14	2.31	2.44	2.39	2.71	2.69	2.87	2.90	3.22
3JHnCb	0.85	1.45	1.54	2.86	2.12	0.72	0.82	1.36	0.87
3JHaHb2	11.90	8.20	8.76	8.76	8.76	9.18	10.40	10.40	10.40
3JHaHb3	3.60	4.91	4.57	4.57	4.57	4.40	3.23	3.23	3.23
3JC'Cg	4.00	2.87	2.96	2.96	2.96	3.30	3.65	3.65	3.65
Residue 5									
3JHnHa	10.00	8.18	7.58	8.44	9.14	9.34	8.83	9.96	9.77
3JHnC'	0.71	0.32	0.54	0.14	0.08	1.41	1.47	1.71	1.85
3JHnCb	0.10	1.52	1.61	3.00	2.25	0.24	0.32	0.20	-0.00
3JNCg1	1.83	1.79	2.05	1.85	1.85	1.54	1.72	1.58	1.58
3JNCg2	0.46	0.59	0.41	0.66	0.66	0.62	0.47	0.82	0.82
3JC'Cg2	3.66	1.39	0.87	3.59	3.59	1.78	1.41	2.97	2.97
Residue 6									
3JHnHa	9.30	7.31	6.64	7.31	8.13	8.18	7.58	8.44	9.10
3JHnC'	-0.06	0.41	0.68	0.21	0.03	0.39	0.60	0.23	0.17
3JHaC'	2.29	1.97	2.05	1.93	2.20	2.23	2.35	2.28	2.58
3JHnCb	1.43	1.84	1.92	3.65	2.81	1.48	1.56	2.90	2.17
3JHaHb2	6.20	6.22	5.76	5.76	5.76	6.74	6.60	6.60	6.60
3JHaHb3	7.00	4.93	4.72	4.72	4.72	4.81	4.47	4.47	4.47
3JC'Cg	2.00	2.05	1.74	1.74	1.74	2.25	2.04	2.04	2.04
Residue 7									
3JHnHa	8.93	6.85	6.16	6.72	7.55	7.11	6.43	7.05	7.77
3JHnC'	-0.04	0.57	0.87	0.42	0.18	0.65	0.92	0.53	0.33
3JHaC'	2.42	1.86	1.91	1.77	2.01	1.92	1.98	1.86	2.10
3JHnCb	1.74	1.92	2.00	3.80	2.95	1.76	1.84	3.44	2.65
3JNCgT	1.08	1.11	1.12	0.96	0.96	0.65	0.53	1.07	1.07
3JC'CgT	2.67	2.28	2.10	1.95	1.95	3.07	3.31	2.86	2.86
Residue 8									
3JHnHa	4.15	5.78	5.30	5.51	5.57	4.62	3.78	3.82	4.71
3JHnC'	1.59	1.57	1.96	1.66	1.65	1.34	1.75	1.33	0.82
3JHaC'	1.43	3.16	2.86	2.86	3.04	1.28	1.20	0.94	1.08
3JHnCb	2.15	1.76	1.78	2.97	2.40	2.36	2.44	4.67	3.75
3JHaHb2	8.70	6.17	5.58	5.58	5.58	7.06	7.22	7.22	7.22
3JHaHb3	4.10	5.23	5.38	5.38	5.38	4.68	4.00	4.00	4.00
3JC'Cg	2.60	2.00	1.64	1.64	1.64	2.39	2.29	2.29	2.29
Residue 9									
3JHnHa	8.63	6.63	6.09	6.54	6.77	6.58	5.86	6.36	7.24
3JHnC'	0.11	1.29	1.60	1.35	1.30	0.57	0.89	0.40	0.12
3JHaC'	2.32	2.69	2.56	2.52	2.73	1.76	1.80	1.64	1.88
3JHnCb	1.26	1.55	1.60	2.75	2.16	2.04	2.12	4.05	3.16
3JNCgT	0.81	1.07	1.06	0.77	0.77	0.69	0.56	0.96	0.96
3JC'CgT	3.00	2.27	2.06	2.13	2.13	3.10	3.35	3.15	3.15
Residue 11									
3JHnHa	7.26	5.59	4.87	5.11	5.55	5.75	4.99	5.29	6.15
3JHnC'	0.31	1.65	2.00	1.79	1.51	0.94	1.29	0.85	0.48
3JHnCb	2.00	1.73	1.80	3.29	2.61	2.15	2.22	4.24	3.35
3JHaHb2	5.70	5.77	5.19	5.19	5.19	5.93	5.53	5.53	5.53
3JHaHb3	6.70	5.92	6.11	6.11	6.11	5.18	4.70	4.70	4.70
Residue 12									
3JHnHa	9.67	8.28	7.70	8.58	9.00	8.66	8.09	9.07	9.62
3JHnC'	0.35	0.72	0.91	0.70	0.68	0.40	0.58	0.26	0.28
3JHaC'	2.87	2.33	2.44	2.39	2.66	2.38	2.52	2.48	2.79
3JHnCb	0.62	1.20	1.28	2.26	1.67	1.26	1.35	2.46	1.80
3JNCgT	1.61	1.68	1.90	1.23	1.23	1.80	2.06	1.48	1.48
3JC'CgT	0.39	1.40	0.77	0.41	0.41	1.34	0.73	0.07	0.07

Residue 13									
3JHnHa	9.91	8.91	8.36	9.40	9.81	8.89	8.34	9.37	9.81
3JHnC'	0.31	0.54	0.70	0.48	0.54	0.50	0.67	0.42	0.48
3JHaC'	2.57	2.46	2.61	2.58	2.89	2.45	2.60	2.57	2.88
3JHnCb	1.17	1.04	1.13	1.99	1.42	1.08	1.18	2.09	1.49
3JNCg2	1.44	0.74	0.66	0.48	0.48	0.61	0.46	1.58	1.58
3JC'Cg2	1.71	1.59	1.02	3.00	3.00	2.88	3.00	1.30	1.30
Residue 14									
3JHnHa	9.62	7.73	7.09	7.86	8.64	8.79	8.23	9.24	9.77
3JHnC'	-0.14	0.31	0.57	0.11	-0.02	0.39	0.56	0.26	0.29
3JHaC'	2.42	2.10	2.19	2.10	2.38	2.42	2.56	2.53	2.84
3JHnCb	1.14	1.72	1.81	3.41	2.59	1.21	1.30	2.35	1.70
3JNCgT	1.50	1.36	1.46	0.92	0.92	1.49	1.64	1.14	1.14
3JC'CgT	0.76	1.82	1.38	1.33	1.33	1.39	0.82	0.44	0.44
Residue 15									
3JHnHa	9.95	9.05	8.52	9.59	9.89	9.43	8.91	10.07	9.97
3JHnC'	0.78	0.66	0.80	0.66	0.74	1.22	1.29	1.45	1.60
3JHaC'	3.02	2.50	2.66	2.64	2.95	2.61	2.79	2.79	3.07
3JHnCb	0.35	0.90	0.99	1.67	1.16	0.34	0.42	0.44	0.18
3JHaHb2	8.40	5.22	3.97	3.97	3.97	8.90	9.98	9.98	9.98
3JHaHb3	1.40	5.30	5.59	5.59	5.59	4.73	3.99	3.99	3.99
3JC'Cg	2.20	1.65	1.08	1.08	1.08	3.16	3.42	3.42	3.42
Residue 16									
3JHnHa	9.77	8.01	7.40	8.23	8.94	9.08	8.54	9.62	10.01
3JHnC'	0.40	0.36	0.59	0.18	0.10	0.49	0.65	0.42	0.50
3JHaC'	3.08	2.18	2.29	2.22	2.51	2.50	2.66	2.65	2.96
3JHnCb	0.93	1.57	1.65	3.09	2.33	1.01	1.10	1.93	1.36
3JHaHb2	5.50	6.32	5.86	5.86	5.86	4.97	4.30	4.30	4.30
3JHaHb3	8.20	5.20	5.34	5.34	5.34	4.48	3.35	3.35	3.35
Residue 17									
3JHnHa	9.82	9.24	8.71	9.82	9.69	8.72	8.16	9.15	8.78
3JHnC'	1.46	1.32	1.39	1.58	1.70	1.88	1.92	2.34	2.38
3JHaC'	2.66	2.55	2.72	2.71	2.98	2.39	2.53	2.50	2.71
3JHnCb	0.09	0.35	0.43	0.43	0.19	0.19	0.25	-0.06	-0.15
3JNCg1	0.25	0.64	0.47	0.34	0.34	0.68	0.54	0.65	0.65
3JNCg2	0.68	1.67	1.89	1.40	1.40	1.70	1.93	0.97	0.97
3JC'Cg1	3.93	2.92	3.04	3.73	3.73	3.07	3.30	3.68	3.68
3JC'Cg2	0.96	1.55	1.23	0.47	0.47	1.44	0.94	0.83	0.83
Residue 18									
3JHnHa	9.56	6.81	6.11	6.66	7.48	8.38	7.79	8.71	9.35
3JHnC'	0.43	0.60	0.90	0.44	0.20	0.32	0.53	0.15	0.12
3JHaC'	2.77	1.85	1.89	1.75	1.99	2.29	2.42	2.36	2.67
3JHnCb	0.86	1.93	2.01	3.81	2.96	1.44	1.52	2.82	2.10
3JHaHb2	9.30	6.61	6.49	6.49	6.49	7.66	8.24	8.24	8.24
3JHaHb3	2.50	5.15	4.94	4.94	4.94	4.61	3.77	3.77	3.77
Residue 19									
3JHaC'	1.22	1.13	0.99	0.69	0.77	1.16	1.05	0.76	0.85
3JHaHb2	6.80	6.45	5.52	5.52	5.52	7.41	7.29	7.29	7.29
3JHaHb3	7.30	5.79	4.43	4.43	4.43	5.34	3.88	3.88	3.88
3JC'Cg	2.20	2.26	1.93	1.93	1.93	2.56	2.43	2.43	2.43
Residue 20									
3JHnHa	7.62	6.74	6.04	6.58	7.47	7.17	6.49	7.13	8.02
3JHnC'	0.18	0.49	0.80	0.30	0.05	0.34	0.62	0.12	-0.09
3JHaC'	2.16	1.81	1.86	1.71	1.96	1.92	1.99	1.87	2.13
3JHaHb2	9.40	8.06	8.58	8.58	8.58	5.39	4.73	4.73	4.73
3JHaHb3	3.00	5.30	4.76	4.76	4.76	8.19	8.92	8.92	8.92
Residue 21									
3JHnHa	5.52	8.40	7.82	8.74	8.68	6.16	5.42	5.82	6.70
3JHaC'	1.46	2.33	2.45	2.40	2.63	1.66	1.67	1.49	1.71
3JHnCb	2.25	0.65	0.72	0.94	0.65	2.11	2.19	4.17	3.27
3JC'Cg	5.50	1.47	0.76	0.76	0.76	2.82	2.90	2.90	2.90
Residue 22									
3JHnHa	7.57	5.95	5.20	5.55	6.38	6.04	5.28	5.66	6.58
3JHnC'	0.01	0.92	1.26	0.83	0.49	0.72	1.06	0.56	0.22
3JHaC'	1.65	1.63	1.63	1.44	1.64	1.62	1.63	1.44	1.65
3JHnCb	2.05	2.08	2.15	4.10	3.23	2.18	2.26	4.32	3.40
3JNCgT	0.75	0.78	0.67	0.69	0.69	0.69	0.57	0.81	0.81
3JC'CgT	3.39	2.42	2.29	2.35	2.35	2.82	2.93	2.82	2.82
Residue 23									
3JHnHa	3.94	3.52	2.65	2.42	3.06	4.30	3.44	3.40	4.28
3JHnC'	1.54	2.17	2.65	2.37	1.82	1.48	1.92	1.51	0.97
3JHaC'	1.52	1.23	1.05	0.76	0.81	1.20	1.11	0.83	0.94
3JHnCb	2.75	2.26	2.34	4.46	3.62	2.40	2.47	4.74	3.81
3JNCg2	2.11	0.90	0.85	0.75	0.75	0.66	0.50	1.67	1.67
3JC'Cg2	0.94	1.81	1.37	2.27	2.27	3.03	3.22	1.23	1.23

Residue 24									
3JHaC'	0.82	1.13	0.94	0.63	0.66	1.16	1.06	0.77	0.86
3JC'Cg	1.50	2.24	2.02	2.02	2.02	1.76	1.35	1.35	1.35
Residue 25									
3JHnHa	5.50	4.12	3.26	3.18	4.00	5.69	4.91	5.20	6.15
3JHnC'	0.67	1.65	2.10	1.73	1.18	0.80	1.16	0.67	0.27
3JHaC'	1.46	1.20	1.09	0.80	0.90	1.52	1.51	1.30	1.49
3JHnCb	2.34	2.36	2.43	4.66	3.75	2.27	2.35	4.50	3.56
3JHaHb2	8.40	4.84	4.36	4.36	4.36	5.97	5.58	5.58	5.58
3JHaHb3	5.30	4.99	4.12	4.12	4.12	4.60	3.83	3.83	3.83
3JC'Cg	3.40	1.46	1.02	1.02	1.02	1.96	1.65	1.65	1.65
Residue 26									
3JHnHa	5.72	3.40	2.50	2.24	2.95	4.39	3.54	3.53	4.41
3JHnC'	0.56	2.15	2.64	2.35	1.75	1.44	1.87	1.45	0.93
3JHaC'	1.30	1.10	0.93	0.62	0.66	1.22	1.14	0.86	0.98
3JHnCb	2.65	2.33	2.40	4.60	3.73	2.39	2.46	4.72	3.79
3JNCg1	2.16	1.74	1.98	1.80	1.80	1.82	2.09	1.93	1.93
3JNCg2	0.62	0.61	0.45	0.67	0.67	0.62	0.48	0.35	0.35
3JC'Cg1	0.82	1.37	0.81	0.22	0.22	1.39	0.89	0.20	0.20
3JC'Cg2	4.18	1.42	0.89	3.50	3.50	1.31	0.65	3.71	3.71
Residue 27									
3JHnHa	4.28	3.30	2.40	2.13	2.83	4.29	3.42	3.39	4.26
3JHnC'	1.00	2.18	2.67	2.38	1.78	1.49	1.93	1.52	0.98
3JHaC'	1.02	1.07	0.90	0.58	0.62	1.20	1.11	0.83	0.94
3JHnCb	2.55	2.35	2.42	4.64	3.77	2.40	2.47	4.74	3.82
3JHaHb2	10.60	8.89	9.90	9.90	9.90	9.21	10.46	10.46	10.46
3JHaHb3	2.40	4.99	4.98	4.98	4.98	4.78	4.43	4.43	4.43
Residue 28									
3JHnHa	4.57	3.63	2.74	2.55	3.31	4.86	4.03	4.13	5.05
3JHaC'	0.98	1.11	0.97	0.66	0.73	1.32	1.26	1.01	1.16
3JHnCb	2.92	2.37	2.44	4.69	3.79	2.37	2.44	4.68	3.74
Residue 29									
3JHnHa	5.55	3.73	2.84	2.67	3.45	5.04	4.22	4.36	5.29
3JHnC'	0.80	1.89	2.35	2.02	1.44	1.10	1.49	1.03	0.56
3JHaC'	1.54	1.13	0.99	0.69	0.76	1.36	1.31	1.07	1.23
3JHnCb	2.40	2.37	2.44	4.68	3.79	2.35	2.42	4.64	3.71
3JHaHb2	9.20	7.86	8.08	8.08	8.08	8.95	10.01	10.01	10.01
3JHaHb3	1.90	5.44	6.15	6.15	6.15	4.86	4.65	4.65	4.65
3JC'Cg	4.00	2.61	2.51	2.51	2.51	3.10	3.31	3.31	3.31
Residue 30									
3JHnHa	5.77	4.08	3.21	3.12	3.95	5.17	4.36	4.53	5.47
3JHnC'	0.66	1.65	2.09	1.72	1.16	1.04	1.42	0.95	0.50
3JHaC'	0.99	1.18	1.06	0.78	0.88	1.39	1.35	1.12	1.28
3JHnCb	2.67	2.38	2.45	4.71	3.80	2.33	2.40	4.61	3.68
3JNCg2	2.10	0.64	0.49	1.75	1.75	0.56	0.37	1.81	1.81
3JC'Cg2	0.96	3.08	3.30	1.15	1.15	3.22	3.51	1.14	1.14
Residue 31									
3JHnC'	1.19	2.21	2.70	2.42	1.81	1.57	2.01	1.62	1.06
3JHaC'	0.76	1.06	0.89	0.57	0.61	1.17	1.07	0.78	0.89
3JHnCb	2.79	2.35	2.42	4.64	3.77	2.40	2.47	4.75	3.83
3JHaHb2	3.80	5.05	4.36	4.36	4.36	4.39	3.20	3.20	3.20
3JHaHb3	11.30	4.77	4.04	4.04	4.04	4.53	3.63	3.63	3.63
3JC'Cg	0.60	1.57	1.12	1.12	1.12	1.33	0.73	0.73	0.73
Residue 32									
3JHnHa	3.82	4.08	3.21	3.12	3.93	4.90	4.07	4.18	5.09
3JHnC'	1.72	1.69	2.14	1.78	1.23	1.19	1.59	1.14	0.66
3JHaC'	1.06	1.20	1.08	0.80	0.89	1.33	1.28	1.03	1.18
3JHnCb	2.57	2.35	2.42	4.65	3.75	2.35	2.42	4.64	3.71
3JHaHb2	7.40	4.58	3.61	3.61	3.61	6.88	6.89	6.89	6.89
3JHaHb3	5.30	4.57	3.66	3.66	3.66	4.69	4.14	4.14	4.14
3JC'Cg	3.30	1.39	0.86	0.86	0.86	2.30	2.14	2.14	2.14
Residue 33									
3JHnHa	7.13	5.59	4.81	5.08	5.97	8.18	7.58	8.45	9.08
3JHnC'	0.26	0.94	1.31	0.85	0.46	0.42	0.63	0.27	0.22
3JHaC'	1.98	1.52	1.50	1.29	1.48	2.24	2.35	2.29	2.58
3JHnCb	2.04	2.21	2.29	4.38	3.47	1.45	1.54	2.84	2.13
3JHaHb2	9.00	6.55	6.13	6.13	6.13	7.89	8.39	8.39	8.39
3JHaHb3	4.00	5.18	5.44	5.44	5.44	4.83	4.46	4.46	4.46
3JC'Cg	2.80	2.13	1.82	1.82	1.82	2.69	2.71	2.71	2.71
Residue 34									
3JHnHa	9.54	8.60	8.03	8.99	9.58	9.33	8.81	9.94	9.95
3JHnC'	-0.05	0.34	0.54	0.19	0.20	1.07	1.16	1.23	1.36
3JHaC'	2.89	2.36	2.49	2.45	2.76	2.58	2.75	2.75	3.04
3JHaHb2	10.20	7.52	7.65	7.65	7.65	6.38	6.11	6.11	6.11
3JHaHb3	4.40	5.25	5.64	5.64	5.64	4.60	3.90	3.90	3.90

3JC'Cg	2.90	2.51	2.39	2.39	2.39	2.12	1.89	1.89	1.89
Residue 36									
3JHnHa	7.98	5.77	5.04	5.34	5.51	5.28	4.48	4.68	5.58
3JHaC'	2.12	1.80	1.75	1.58	1.70	1.44	1.40	1.18	1.35
3JHnCb	1.86	1.38	1.44	2.48	1.98	2.27	2.35	4.49	3.57
3JNCg2	2.08	1.32	1.44	1.14	1.14	0.82	0.76	1.81	1.81
3JC'Cg2	0.71	1.88	1.58	0.95	0.95	2.97	3.18	0.55	0.55
Residue 37									
3JHaC'	1.71	1.27	1.19	0.93	1.06	1.25	1.16	0.89	1.02
3JHaHb2	6.50	6.31	5.29	5.29	5.29	7.16	6.86	6.86	6.86
3JHaHb3	7.00	5.93	4.66	4.66	4.66	5.55	4.20	4.20	4.20
Residue 38									
3JHaC'	1.06	1.08	0.84	0.51	0.51	1.14	1.01	0.71	0.79
3JHaHb2	7.80	7.37	7.13	7.13	7.13	7.71	7.83	7.83	7.83
3JHaHb3	5.40	5.08	3.32	3.32	3.32	5.19	3.72	3.72	3.72
3JC'Cg	2.70	2.64	2.54	2.54	2.54	2.68	2.63	2.63	2.63
Residue 39									
3JHnHa	5.15	3.94	3.10	2.97	3.67	5.23	4.43	4.61	5.53
3JHnC'	1.15	1.85	2.31	1.98	1.46	1.04	1.42	0.95	0.52
3JHaC'	1.65	1.35	1.19	0.93	1.01	1.42	1.38	1.15	1.32
3JHnCb	2.19	2.30	2.37	4.50	3.64	2.31	2.38	4.57	3.63
3JHaHb2	4.70	4.67	3.91	3.91	3.91	6.75	6.84	6.84	6.84
3JHaHb3	4.40	4.82	3.92	3.92	3.92	6.23	6.17	6.17	6.17
3JC'Cg	1.80	1.42	0.93	0.93	0.93	2.24	2.07	2.07	2.07
Residue 40									
3JHnHa	9.45	5.76	4.99	5.29	6.17	8.20	7.59	8.47	9.20
3JHnC'	0.59	0.91	1.27	0.81	0.44	0.24	0.46	0.02	-0.04
3JHnCb	1.27	2.17	2.25	4.29	3.39	1.58	1.66	3.12	2.34
3JHaHb2	12.20	7.39	7.42	7.42	7.42	7.74	8.17	8.17	8.17
3JHaHb3	2.00	5.21	5.48	5.48	5.48	4.65	3.95	3.95	3.95
3JC'Cg	3.40	2.48	2.34	2.34	2.34	2.67	2.70	2.70	2.70
Residue 41									
3JHnHa	8.41	5.87	5.11	5.45	6.32	7.47	6.81	7.52	8.25
3JHnC'	-0.19	0.88	1.23	0.78	0.42	0.51	0.76	0.36	0.21
3JHaC'	2.20	1.60	1.59	1.40	1.60	2.03	2.11	2.00	2.27
3JHnCb	1.44	2.14	2.21	4.22	3.33	1.70	1.78	3.33	2.55
3JC'Cg	3.50	2.74	2.77	2.77	2.77	3.13	3.39	3.39	3.39
Residue 42									
3JHnHa	9.81	8.45	7.86	8.79	9.45	9.08	8.54	9.62	9.40
3JHnC'	1.52	0.28	0.48	0.09	0.07	1.53	1.59	1.87	1.97
3JHaC'	2.81	2.31	2.44	2.39	2.70	2.50	2.66	2.65	2.89
3JHnCb	0.20	1.44	1.53	2.83	2.10	0.27	0.35	0.22	0.04
3JHaHb2	8.30	5.90	5.24	5.24	5.24	5.55	5.00	5.00	5.00
3JHaHb3	4.30	5.00	4.83	4.83	4.83	5.02	4.37	4.37	4.37
3JC'Cg	2.40	1.92	1.54	1.54	1.54	1.78	1.39	1.39	1.39
Residue 43									
3JHnHa	9.66	9.33	8.82	9.95	10.31	8.81	8.25	9.26	9.87
3JHnC'	0.25	0.47	0.61	0.40	0.52	0.23	0.42	0.05	0.08
3JHaC'	2.54	2.58	2.75	2.76	3.08	2.42	2.56	2.54	2.86
3JHnCb	0.99	0.91	1.01	1.75	1.20	1.31	1.40	2.58	1.88
3JHaHb2	9.90	8.03	8.57	8.57	8.57	9.10	10.26	10.26	10.26
3JHaHb3	1.50	5.02	4.89	4.89	4.89	4.84	4.57	4.57	4.57
3JC'Cg	3.00	2.77	2.83	2.83	2.83	3.17	3.43	3.43	3.43
Residue 44									
3JHnHa	10.27	9.25	8.73	9.85	9.93	9.47	8.96	10.13	10.00
3JHnC'	0.09	0.97	1.06	1.09	1.20	1.26	1.32	1.50	1.66
3JHaC'	3.01	2.56	2.73	2.72	3.01	2.63	2.80	2.81	3.09
3JHnCb	0.92	0.60	0.68	1.00	0.63	0.29	0.38	0.35	0.10
3JNCg2	1.62	0.79	0.73	1.49	1.49	0.60	0.42	1.79	1.79
3JC'Cg2	0.80	2.72	2.75	1.04	1.04	3.21	3.49	0.97	0.97
Residue 45									
3JHnHa	8.73	7.86	7.23	8.02	8.69	8.29	7.69	8.59	8.15
3JHnC'	1.67	0.47	0.70	0.33	0.23	2.11	2.14	2.65	2.63
3JHaC'	2.57	2.13	2.23	2.15	2.43	2.25	2.38	2.31	2.50
3JHnCb	0.04	1.56	1.64	3.04	2.30	0.21	0.27	-0.10	-0.14
3JHaHb2	3.50	6.75	6.62	6.62	6.62	4.42	3.31	3.31	3.31
3JHaHb3	9.80	5.52	5.80	5.80	5.80	4.46	3.42	3.42	3.42
Residue 46									
3JHnHa	6.47	7.28	7.05	7.57	6.96	6.96	7.19	7.48	6.03
3JHnC'	3.58	1.81	2.12	2.04	2.42	2.34	2.84	2.59	3.55
3JHaC'	7.12	4.34	3.97	4.15	4.37	6.91	6.03	6.52	6.76
3JHnCb	0.27	0.95	0.97	1.12	0.89	0.82	0.79	0.41	0.37
Residue 47									
3JHnC'	0.98	0.69	0.91	0.64	0.84	0.52	0.83	0.33	0.89
Residue 48									

3JHnHa	9.41	6.38	5.66	6.11	6.78	8.85	8.30	9.32	9.58
3JHnC'	0.11	1.02	1.33	1.00	0.73	0.82	0.96	0.86	0.91
3JHaC'	2.31	1.78	1.79	1.63	1.84	2.44	2.58	2.56	2.85
3JHnCb	0.96	1.81	1.89	3.53	2.76	0.88	0.96	1.60	1.12
3JC'Cg	2.50	2.12	1.84	1.84	1.84	2.55	2.51	2.51	2.51
Residue 49									
3JHnHa	6.97	6.51	5.80	6.28	7.08	5.69	4.91	5.20	6.14
3JHnC'	0.05	0.73	1.04	0.61	0.34	0.83	1.19	0.70	0.31
3JHaC'	1.61	1.80	1.82	1.67	1.89	1.53	1.51	1.31	1.50
3JHnCb	2.08	1.96	2.04	3.87	3.02	2.25	2.33	4.46	3.53
3JC'Cg	1.40	1.80	1.36	1.36	1.36	2.35	2.26	2.26	2.26
Residue 50									
3JHnHa	7.11	7.02	6.33	6.93	7.77	8.22	7.62	8.50	9.24
3JHnC'	-0.02	0.50	0.79	0.33	0.11	0.21	0.44	-0.01	-0.06
3JHaC'	2.02	1.90	1.96	1.82	2.08	2.24	2.36	2.30	2.60
3JHnCb	1.89	1.90	1.98	3.76	2.91	1.58	1.67	3.13	2.35
3JHaHb2	11.40	7.49	7.61	7.61	7.61	9.23	10.48	10.48	10.48
3JHaHb3	3.90	5.28	5.61	5.61	5.61	4.42	3.34	3.34	3.34
3JC'Cg	2.40	2.53	2.43	2.43	2.43	3.30	3.65	3.65	3.65
Residue 51									
3JHnHa	8.06	8.46	7.88	8.81	8.81	6.68	5.98	6.50	7.35
3JHnC'	-0.07	1.35	1.46	1.59	1.59	0.59	0.89	0.43	0.17
3JHaC'	2.30	2.32	2.45	2.40	2.64	1.80	1.84	1.69	1.93
3JHnCb	1.80	0.67	0.74	1.03	0.72	1.99	2.07	3.93	3.06
3JHaHb2	9.20	6.16	5.42	5.42	5.42	7.10	7.39	7.39	7.39
3JHaHb3	3.20	5.29	5.59	5.59	5.59	4.52	3.56	3.56	3.56
3JC'Cg	2.90	2.01	1.62	1.62	1.62	2.41	2.35	2.35	2.35
Residue 52									
3JHnHa	2.48	5.64	4.89	5.16	5.78	4.93	4.11	4.22	5.11
3JHnC'	2.92	1.35	1.70	1.41	1.06	1.21	1.61	1.18	0.71
3JHaC'	0.90	1.65	1.61	1.42	1.58	1.36	1.30	1.06	1.21
3JHnCb	2.38	1.91	1.98	3.67	2.92	2.32	2.39	4.58	3.66
3JHaHb2	10.00	4.97	4.28	4.28	4.28	9.10	10.26	10.26	10.26
3JHaHb3	1.30	5.65	5.34	5.34	5.34	4.46	3.34	3.34	3.34
Residue 54									
3JHnHa	9.59	8.16	7.57	8.43	8.74	7.89	7.27	8.07	8.75
3JHnC'	0.19	0.94	1.11	0.99	0.95	0.44	0.67	0.28	0.19
3JHaC'	2.13	2.26	2.37	2.31	2.57	2.15	2.25	2.17	2.45
3JHnCb	1.39	1.10	1.17	2.00	1.48	1.57	1.65	3.08	2.32
3JHaHb2	11.30	7.54	7.81	7.81	7.81	8.98	10.07	10.07	10.07
3JHaHb3	1.40	5.44	5.42	5.42	5.42	4.72	4.21	4.21	4.21
3JC'Cg	3.50	2.58	2.53	2.53	2.53	3.16	3.42	3.42	3.42
Residue 55									
3JHnHa	9.20	5.95	5.21	5.56	6.35	8.13	7.52	8.38	9.13
3JHnC'	-0.33	0.90	1.25	0.81	0.49	0.23	0.46	0.02	-0.06
3JHaC'	3.11	1.74	1.71	1.54	1.74	2.21	2.33	2.26	2.56
3JHnCb	1.60	2.09	2.16	4.09	3.23	1.61	1.69	3.18	2.39
3JNCgT	0.82	0.79	0.69	0.88	0.88	0.68	0.57	1.28	1.28
3JC'CgT	3.02	2.90	3.02	2.91	2.91	3.29	3.64	3.04	3.04
Residue 56									
3JHnHa	3.70	3.60	2.71	2.51	3.23	4.17	3.30	3.23	4.09
3JHnC'	1.94	2.03	2.51	2.21	1.63	1.57	2.01	1.62	1.07
3JHaC'	0.56	1.13	0.98	0.68	0.74	1.18	1.08	0.80	0.90
3JHnCb	2.25	2.32	2.39	4.59	3.71	2.39	2.46	4.73	3.81
3JHaHb2	9.90	5.22	4.29	4.29	4.29	9.04	10.17	10.17	10.17
3JHaHb3	2.10	4.96	4.68	4.68	4.68	4.81	4.51	4.51	4.51
3JC'Cg	2.80	1.63	1.14	1.14	1.14	3.17	3.43	3.43	3.43
Residue 57									
3JHnHa	3.70	3.48	2.59	2.36	3.05	5.18	4.37	4.54	5.47
3JHnC'	1.88	2.14	2.62	2.33	1.75	1.04	1.42	0.96	0.51
3JHaC'	1.17	1.13	0.97	0.66	0.71	1.40	1.36	1.12	1.29
Residue 58									
3JHnHa	4.14	4.65	3.82	3.87	4.70	5.13	4.32	4.48	5.42
3JHnC'	1.56	1.40	1.81	1.41	0.92	1.05	1.44	0.97	0.52
3JHaC'	1.36	1.35	1.26	1.01	1.14	1.38	1.34	1.10	1.27
3JHnCb	2.48	2.31	2.38	4.55	3.65	2.34	2.41	4.63	3.69
3JHaHb2	11.40	4.47	3.08	3.08	3.08	8.41	9.25	9.25	9.25
3JHaHb3	2.40	4.81	4.42	4.42	4.42	4.52	3.63	3.63	3.63
3JC'Cg	5.60	1.36	0.72	0.72	0.72	2.95	3.12	3.12	3.12
Residue 59									
3JHnHa	9.45	4.41	3.57	3.56	4.35	7.99	7.37	8.20	8.95
3JHnC'	0.07	1.59	2.02	1.66	1.15	0.28	0.51	0.07	-0.02
3JHaC'	2.41	1.28	1.19	0.92	1.03	2.17	2.28	2.20	2.50
3JHnCb	1.49	2.27	2.35	4.50	3.61	1.64	1.72	3.24	2.45
3JHaHb2	10.00	7.44	7.48	7.48	7.48	9.20	10.44	10.44	10.44

3JHaHb3	1.80	5.53	6.32	6.32	6.32	4.76	4.37	4.37	4.37
3JC'Cg	3.60	2.43	2.26	2.26	2.26	3.21	3.49	3.49	3.49
Residue 60									
3JHnHa	7.26	6.55	6.30	6.64	6.27	7.03	7.28	7.58	6.17
3JHnC'	2.52	1.33	1.67	1.38	1.65	2.07	2.56	2.25	3.21
3JHaC'	6.49	4.37	3.92	4.09	4.33	7.04	6.14	6.65	6.91
3JHnCb	0.80	1.63	1.61	2.24	1.88	0.99	0.94	0.61	0.56
3JHaHb2	6.70	4.60	3.52	3.52	3.52	7.50	7.77	7.77	7.77
3JHaHb3	5.50	4.83	4.24	4.24	4.24	4.63	3.88	3.88	3.88
3JC'Cg	2.00	1.39	0.82	0.82	0.82	2.59	2.56	2.56	2.56
Residue 61									
3JHnHa	7.34	7.88	7.31	8.09	8.52	5.70	4.92	5.22	6.15
3JHnC'	-0.11	0.77	1.01	0.73	0.71	0.82	1.18	0.69	0.30
3JHaC'	1.56	2.44	2.48	2.44	2.70	1.53	1.52	1.31	1.50
3JHnCb	2.08	1.34	1.42	2.54	1.90	2.25	2.33	4.46	3.53
3JNCg2	2.15	0.82	0.76	0.73	0.73	0.58	0.40	1.84	1.84
3JC'Cg2	0.98	1.88	1.46	2.35	2.35	3.24	3.54	0.99	0.99
Residue 62									
3JHnHa	9.01	6.76	6.12	6.64	7.09	8.18	7.58	8.45	9.11
3JHnC'	0.19	1.09	1.37	1.10	0.95	0.36	0.58	0.19	0.14
3JHaC'	2.79	2.19	2.17	2.06	2.28	2.23	2.35	2.28	2.58
3JHnCb	1.01	1.62	1.68	3.02	2.35	1.49	1.58	2.93	2.20
3JHaHb2	11.10	7.15	7.20	7.20	7.20	8.28	9.14	9.14	9.14
3JHaHb3	3.10	5.57	5.75	5.75	5.75	5.21	4.66	4.66	4.66
Residue 63									
3JHnHa	2.36	5.17	4.52	4.64	4.98	3.48	2.58	2.35	3.09
3JHnC'	2.62	1.68	2.08	1.80	1.56	2.05	2.53	2.22	1.62
3JHaC'	1.05	2.28	2.08	1.95	2.10	1.09	0.93	0.62	0.68
3JHnCb	2.34	1.91	1.96	3.51	2.83	2.36	2.43	4.67	3.79
3JHaHb2	3.50	6.14	5.67	5.67	5.67	4.77	4.19	4.19	4.19
3JHaHb3	4.40	5.44	5.50	5.50	5.50	8.53	9.46	9.46	9.46
3JC'Cg	1.20	1.98	1.65	1.65	1.65	1.39	0.85	0.85	0.85
Residue 64									
3JHnHa	7.19	6.91	6.38	6.90	7.17	7.11	7.38	7.69	6.36
3JHnC'	1.63	1.10	1.40	1.11	1.08	1.72	2.17	1.80	2.74
3JHaC'	7.17	2.73	2.62	2.59	2.82	7.10	6.20	6.72	7.00
3JHnCb	1.35	1.56	1.61	2.76	2.17	1.21	1.14	0.90	0.83
3JHaHb2	9.90	6.76	6.58	6.58	6.58	9.12	10.32	10.32	10.32
3JHaHb3	1.50	5.44	5.53	5.53	5.53	4.64	3.91	3.91	3.91
3JC'Cg	3.40	2.24	2.01	2.01	2.01	3.19	3.48	3.48	3.48
Residue 65									
3JHnHa	5.63	7.19	6.59	7.20	7.54	5.81	5.04	5.36	6.28
3JHnC'	0.74	1.06	1.29	1.10	0.99	0.81	1.16	0.68	0.31
3JHaC'	1.26	2.33	2.32	2.25	2.48	1.56	1.56	1.36	1.55
3JHaHb2	8.90	7.81	8.17	8.17	8.17	7.57	8.02	8.02	8.02
3JHaHb3	4.20	5.29	5.15	5.15	5.15	5.21	4.64	4.64	4.64
Residue 66									
3JHnHa	9.82	7.07	6.40	7.01	7.77	9.22	8.69	9.80	10.32
3JHnC'	-0.40	0.59	0.87	0.44	0.25	0.24	0.40	0.08	0.17
3JHaC'	3.07	1.97	2.02	1.90	2.15	2.55	2.71	2.71	3.04
3JHnCb	1.34	1.82	1.90	3.58	2.76	1.13	1.22	2.21	1.57
Residue 67									
3JHnHa	9.88	7.77	7.14	7.91	8.58	7.57	6.92	7.65	8.47
3JHnC'	0.15	0.51	0.75	0.38	0.27	0.32	0.58	0.10	-0.05
3JHaC'	1.92	2.12	2.21	2.13	2.40	2.05	2.13	2.03	2.31
3JHnCb	0.86	1.57	1.65	3.07	2.33	1.79	1.88	3.55	2.72
3JC'Cg	3.40	1.66	1.20	1.20	1.20	3.22	3.52	3.52	3.52
Residue 68									
3JHnHa	9.70	7.08	6.40	7.01	7.84	9.25	8.72	9.84	10.26
3JHnC'	0.36	0.49	0.77	0.31	0.10	0.40	0.55	0.30	0.40
3JHaC'	2.85	1.91	1.98	1.84	2.10	2.56	2.72	2.72	3.05
3JHnCb	0.87	1.89	1.97	3.73	2.88	1.00	1.09	1.93	1.35
3JHaHb2	10.10	7.89	8.32	8.32	8.32	8.78	9.79	9.79	9.79
3JHaHb3	2.00	5.04	4.93	4.93	4.93	4.83	4.48	4.48	4.48
3JC'Cg	0.80	2.72	2.74	2.74	2.74	3.06	3.28	3.28	3.28
Residue 69									
3JHnHa	9.59	8.86	8.31	9.33	9.84	9.00	8.46	9.51	9.73
3JHaC'	2.56	2.44	2.59	2.56	2.87	2.48	2.63	2.62	2.91
3JHnCb	1.17	1.17	1.26	2.27	1.64	0.81	0.90	1.46	1.00
3JHaHb2	4.70	4.73	3.79	3.79	3.79	4.78	4.09	4.09	4.09
3JHaHb3	9.50	4.66	3.85	3.85	3.85	4.40	3.10	3.10	3.10
3JC'Cg	0.80	1.44	0.92	0.92	0.92	1.47	1.02	1.02	1.02
Residue 70									
3JHnHa	9.88	9.41	8.90	10.05	10.23	9.27	8.75	9.87	9.70
3JHnC'	0.83	0.76	0.87	0.80	0.94	1.39	1.45	1.67	1.80

3JHaC'	2.87	2.61	2.78	2.79	3.10	2.56	2.73	2.73	2.99
3JHnCb	0.39	0.68	0.77	1.21	0.78	0.29	0.37	0.30	0.09
3JNCg2	0.50	1.06	1.06	0.94	0.94	1.36	1.49	0.99	0.99
3JC'Cg2	2.43	1.49	1.06	2.19	2.19	1.74	1.35	1.21	1.21
Residue 71									
3JHnHa	8.30	7.29	6.63	7.29	8.10	6.94	6.25	6.83	7.64
3JHnC'	0.12	0.43	0.71	0.25	0.07	0.58	0.87	0.42	0.20
3JHnCb	1.60	1.83	1.91	3.62	2.79	1.88	1.96	3.72	2.88
Residue 72									
3JHaC'	2.33	1.82	1.84	1.69	1.91	1.84	1.89	1.74	1.95
3JHaHb2	6.40	5.96	5.49	5.49	5.49	5.51	4.72	4.72	4.72
3JHaHb3	6.60	5.54	5.47	5.47	5.47	5.54	5.47	5.47	5.47
3JC'Cg	1.60	1.93	1.58	1.58	1.58	1.76	1.31	1.31	1.31
Residue 73									
3JHnHa	7.52	6.41	5.70	6.15	6.86	6.26	5.61	6.01	6.57
3JHaC'	2.11	1.83	1.84	1.69	1.90	2.21	2.13	2.02	2.23
3JHnCb	1.39	1.88	1.96	3.67	2.87	1.87	1.94	3.55	2.80
3JHaHb2	8.60	5.70	4.92	4.92	4.92	8.34	9.17	9.17	9.17
3JHaHb3	4.50	5.18	5.19	5.19	5.19	4.64	3.92	3.92	3.92
3JC'Cg	2.80	1.83	1.40	1.40	1.40	2.91	3.07	3.07	3.07
Residue 74									
3JHnHa	6.67	7.47	6.87	7.56	7.94	6.42	5.83	6.25	6.57
3JHaC'	2.44	2.32	2.35	2.28	2.52	2.47	2.36	2.29	2.49
3JHnCb	1.45	1.39	1.46	2.56	1.96	1.64	1.69	2.96	2.34
3JC'Cg	2.20	2.46	2.33	2.33	2.33	2.39	2.28	2.28	2.28

Each *J*-coupling constant was calculated according to Eqs. S9–S20 with parameters in Table S9B and torsions obtained from 20 unique and independent 316-ns^{smt} NPT MD simulations in water at 303 K using a cutoff of 8.0 Å for nonbonded interactions and the Particle Mesh Ewald method to calculate electrostatic interactions of two atoms at separations of >8.0 Å and other conditions specified in Methods and Table S1.

Table S11D. Experimental and calculated *J*-coupling constants of lysozyme in water at 308 K.

		FF12MC (12.64 μs^{smt})				FF14SB (12.64 μs^{smt})			
	EXPT[15,16]	ORIG	SCHM	BFIT	DFT	ORIG	SCHM	BFIT	DFT
Residue 2									
3JHnHa	9.90	7.28	6.62	7.28	8.00	8.37	7.78	8.70	9.30
3JHaHb2	9.80	6.19	8.08	8.08	8.08	6.19	8.08	8.08	8.08
3JC'Hb2	2.60	3.44	3.76	3.76	3.76	3.44	3.76	3.76	3.76
Residue 3									
3JHnHa	6.80	7.92	7.31	8.12	8.57	7.82	7.19	7.97	8.69
3JHaHb2	7.70	7.00	6.71	6.71	6.71	8.55	9.31	9.31	9.31
3JHaHb3	1.00	5.59	6.35	6.35	6.35	5.07	5.19	5.19	5.19
3JC'Hb2	1.40	2.66	2.88	2.88	2.88	1.94	1.78	1.78	1.78
3JC'Hb3	3.60	3.35	3.92	3.92	3.92	4.54	5.68	5.68	5.68
Residue 6									
3JHaHb2	12.60	7.09	7.02	7.02	7.02	9.30	10.61	10.61	10.61
3JHaHb3	0.90	5.12	5.13	5.13	5.13	4.52	3.68	3.68	3.68
3JC'Hb2	3.70	2.94	3.27	3.27	3.27	1.80	1.52	1.52	1.52
3JC'Hb3	2.60	3.58	4.24	4.24	4.24	5.27	6.76	6.76	6.76
Residue 7									
3JHnHa	3.30	3.37	2.47	2.22	2.91	4.68	3.84	3.89	4.79
Residue 8									
3JHnHa	5.30	3.97	3.09	2.98	3.79	5.00	4.17	4.30	5.23
3JHaHb2	1.20	4.68	3.44	3.44	3.44	4.47	3.38	3.38	3.38
3JC'Hb2	8.50	4.85	6.14	6.14	6.14	5.25	6.73	6.73	6.73
3JC'Hb3	3.10	2.03	1.87	1.87	1.87	1.99	1.77	1.77	1.77
Residue 9									
3JHnHa	4.10	3.31	2.41	2.14	2.85	4.09	3.22	3.14	3.99
Residue 10									
3JHnHa	4.30	3.40	2.50	2.25	2.97	4.54	3.69	3.72	4.61
Residue 11									
3JHnHa	4.90	3.39	2.49	2.24	2.96	4.64	3.80	3.85	4.75
Residue 12									
3JHnHa	4.50	3.50	2.61	2.38	3.12	4.85	4.02	4.12	5.03

3JHaHb2	12.60	8.10	8.60	8.60	8.60	9.11	10.28	10.28	10.28
3JHaHb3	1.00	5.13	5.34	5.34	5.34	4.90	4.76	4.76	4.76
3JC'Hb2	1.80	2.18	2.15	2.15	2.15	1.59	1.27	1.27	1.27
3JC'Hb3	2.20	4.24	5.23	5.23	5.23	5.02	6.39	6.39	6.39
Residue 13									
3JHnHa	4.30	3.41	2.51	2.26	2.96	4.58	3.73	3.76	4.66
Residue 15									
3JHaHb2	9.00	7.65	7.89	7.89	7.89	9.20	10.43	10.43	10.43
3JHaHb3	0.60	5.37	5.60	5.60	5.60	4.70	4.20	4.20	4.20
3JC'Hb2	2.20	2.36	2.41	2.41	2.41	1.67	1.37	1.37	1.37
Residue 17									
3JHnHa	7.90	7.74	7.12	7.88	7.82	7.17	6.49	7.13	7.96
3JHaHb2	9.50	5.00	3.75	3.75	3.75	7.00	6.94	6.94	6.94
3JHaHb3	1.00	5.31	5.47	5.47	5.47	4.81	4.50	4.50	4.50
3JC'Hb2	1.80	4.25	5.25	5.25	5.25	3.19	3.65	3.65	3.65
3JC'Hb3	2.20	2.12	2.03	2.03	2.03	3.54	4.16	4.16	4.16
Residue 18									
3JHnHa	5.40	8.29	7.76	8.63	8.84	4.74	3.91	3.98	4.87
3JHaHb2	1.70	4.70	3.55	3.55	3.55	4.37	3.13	3.13	3.13
3JHaHb3	13.20	4.90	4.48	4.48	4.48	5.08	4.46	4.46	4.46
3JC'Hb2	8.30	4.77	6.02	6.02	6.02	4.88	6.16	6.16	6.16
Residue 19									
3JHnHa	7.00	7.69	7.07	7.82	7.84	7.07	7.32	7.63	6.27
Residue 20									
3JHnHa	6.20	8.23	7.64	8.52	8.74	4.64	3.80	3.84	4.74
3JHaHb2	1.40	5.91	5.42	5.42	5.42	4.43	3.37	3.37	3.37
3JHaHb3	12.00	5.00	4.66	4.66	4.66	4.40	3.28	3.28	3.28
3JC'Hb2	8.10	3.85	4.63	4.63	4.63	5.29	6.79	6.79	6.79
3JC'Hb3	4.40	2.85	3.11	3.11	3.11	1.96	1.73	1.73	1.73
Residue 23									
3JHaHb2	8.30	7.89	8.31	8.31	8.31	9.00	10.14	10.14	10.14
3JHaHb3	0.20	4.90	4.48	4.48	4.48	4.60	3.93	3.93	3.93
3JC'Hb2	3.00	2.53	2.62	2.62	2.62	1.96	1.77	1.77	1.77
3JC'Hb3	2.60	4.24	5.22	5.22	5.22	5.04	6.41	6.41	6.41
Residue 24									
3JHaHb2	1.00	7.22	7.34	7.34	7.34	5.12	4.62	4.62	4.62
3JC'Hb2	1.30	2.27	2.25	2.25	2.25	2.57	2.68	2.68	2.68
3JC'Hb3	7.40	3.72	4.44	4.44	4.44	2.30	2.26	2.26	2.26
Residue 27									
3JHaHb2	12.10	5.76	4.98	4.98	4.98	8.94	10.00	10.00	10.00
3JHaHb3	0.50	5.40	5.76	5.76	5.76	4.83	4.55	4.55	4.55
3JC'Hb2	0.30	3.69	4.42	4.42	4.42	1.80	1.56	1.56	1.56
3JC'Hb3	1.10	2.63	2.79	2.79	2.79	4.92	6.24	6.24	6.24
Residue 28									
3JHnHa	6.80	4.11	3.25	3.17	3.99	5.33	4.53	4.74	5.67
Residue 29									
3JHnHa	4.90	4.14	3.27	3.20	4.03	5.16	4.34	4.51	5.45
Residue 30									
3JHaHb2	0.20	4.76	4.19	4.19	4.19	4.39	3.28	3.28	3.28
3JHaHb3	6.80	4.58	3.37	3.37	3.37	4.42	3.34	3.34	3.34
3JC'Hb2	7.70	4.92	6.25	6.25	6.25	5.31	6.82	6.82	6.82
3JC'Hb3	2.30	2.28	2.18	2.18	2.18	1.94	1.71	1.71	1.71
Residue 31									
3JHnHa	3.20	3.33	2.43	2.16	2.86	4.21	3.34	3.29	4.15
Residue 32									
3JHnHa	3.40	3.23	2.33	2.04	2.72	4.59	3.74	3.78	4.67
Residue 33									
3JHaHb2	2.10	5.04	3.92	3.92	3.92	4.43	3.33	3.33	3.33
3JHaHb3	7.90	4.98	4.81	4.81	4.81	4.46	3.44	3.44	3.44
3JC'Hb2	1.10	4.49	5.61	5.61	5.61	5.26	6.74	6.74	6.74
3JC'Hb3	12.50	2.22	2.16	2.16	2.16	1.97	1.74	1.74	1.74
Residue 34									
3JHnHa	7.20	4.79	3.97	4.05	4.87	7.36	6.70	7.38	8.24
3JHaHb2	12.10	7.58	7.90	7.90	7.90	9.22	10.50	10.50	10.50
3JHaHb3	1.60	5.32	5.65	5.65	5.65	4.62	3.98	3.98	3.98
3JC'Hb2	1.10	2.44	2.55	2.55	2.55	1.72	1.42	1.42	1.42
3JC'Hb3	2.20	3.80	4.56	4.56	4.56	5.14	6.56	6.56	6.56
Residue 35									
3JHnHa	6.40	5.56	4.79	5.05	5.90	7.58	6.93	7.66	8.39
Residue 38									
3JHnHa	5.80	7.14	6.86	7.36	7.05	7.08	7.34	7.65	6.42
3JHaHb2	11.90	7.09	7.16	7.16	7.16	9.09	10.24	10.24	10.24
3JC'Hb2	0.70	2.85	3.12	3.12	3.12	2.03	1.83	1.83	1.83
3JC'Hb3	3.00	3.65	4.33	4.33	4.33	5.29	6.79	6.79	6.79
Residue 39									

3JHnHa	8.80	7.85	7.22	8.01	8.52	7.70	7.06	7.82	8.63
3JHaHb2	2.20	4.61	3.63	3.63	3.63	4.88	3.94	3.94	3.94
3JHaHb3	12.70	4.72	3.97	3.97	3.97	4.53	3.69	3.69	3.69
3JC'Hb2	8.10	4.96	6.30	6.30	6.30	4.90	6.20	6.20	6.20
3JC'Hb3	2.20	2.03	1.84	1.84	1.84	2.21	2.13	2.13	2.13
Residue 40									
3JHnHa	5.20	4.98	4.18	4.30	5.06	5.46	4.67	4.90	5.84
3JC'Hb2	0.40	3.44	3.76	3.76	3.76	3.44	3.76	3.76	3.76
Residue 41									
3JHnHa	9.20	7.46	6.81	7.51	8.22	7.57	6.92	7.65	8.49
3JHaHb2	10.00	7.89	8.35	8.35	8.35	7.25	7.44	7.44	7.44
3JHaHb3	1.20	5.27	5.28	5.28	5.28	5.34	5.16	5.16	5.16
3JC'Hb2	1.90	2.25	2.24	2.24	2.24	2.64	2.81	2.81	2.81
Residue 43									
3JHnHa	9.30	8.51	8.01	8.93	8.78	7.14	6.46	7.09	6.51
3JHaHb2	3.90	6.19	8.08	8.08	8.08	6.19	8.08	8.08	8.08
3JC'Hb2	1.80	3.44	3.76	3.76	3.76	3.44	3.76	3.76	3.76
Residue 44									
3JHnHa	9.30	7.91	7.29	8.10	8.73	8.47	7.89	8.83	8.41
Residue 45									
3JHnHa	7.70	6.42	5.70	6.16	6.99	6.78	6.08	6.62	7.50
Residue 46									
3JHnHa	9.00	6.77	6.08	6.62	7.34	8.33	7.74	8.64	9.23
Residue 47									
3JHnHa	4.00	5.17	4.41	4.56	5.08	4.76	3.92	4.00	4.90
3JHaHb2	2.40	6.19	8.08	8.08	8.08	6.19	8.08	8.08	8.08
3JC'Hb2	1.70	3.44	3.76	3.76	3.76	3.44	3.76	3.76	3.76
Residue 48									
3JHaHb2	0.20	4.67	3.82	3.82	3.82	4.66	4.07	4.07	4.07
3JHaHb3	1.20	4.75	3.91	3.91	3.91	9.26	10.55	10.55	10.55
3JC'Hb2	0.70	4.88	6.19	6.19	6.19	1.81	1.53	1.53	1.53
3JC'Hb3	9.60	2.13	1.97	1.97	1.97	1.83	1.55	1.55	1.55
Residue 51									
3JHaHb2	13.30	6.19	8.08	8.08	8.08	6.19	8.08	8.08	8.08
3JC'Hb2	1.70	3.44	3.76	3.76	3.76	3.44	3.76	3.76	3.76
Residue 52									
3JHaHb2	10.30	8.92	9.93	9.93	9.93	9.23	10.49	10.49	10.49
3JHaHb3	0.30	4.65	3.87	3.87	3.87	4.44	3.40	3.40	3.40
3JC'Hb3	1.10	5.03	6.41	6.41	6.41	5.27	6.77	6.77	6.77
Residue 53									
3JHaHb2	12.50	8.68	9.48	9.48	9.48	9.26	10.55	10.55	10.55
3JHaHb3	0.80	5.31	5.84	5.84	5.84	4.75	4.35	4.35	4.35
3JC'Hb2	2.60	1.68	1.41	1.41	1.41	1.66	1.34	1.34	1.34
3JC'Hb3	1.90	4.56	5.72	5.72	5.72	5.13	6.55	6.55	6.55
Residue 55									
3JHaHb2	3.60	6.19	8.08	8.08	8.08	6.19	8.08	8.08	8.08
3JC'Hb2	7.00	3.44	3.76	3.76	3.76	3.44	3.76	3.76	3.76
Residue 56									
3JHnHa	9.70	7.52	6.87	7.58	8.40	9.50	8.99	10.16	10.60
Residue 59									
3JHaHb2	1.90	4.35	2.98	2.98	2.98	4.43	3.39	3.39	3.39
3JHaHb3	8.10	4.73	4.21	4.21	4.21	4.42	3.38	3.38	3.38
3JC'Hb2	8.50	5.18	6.63	6.63	6.63	5.30	6.81	6.81	6.81
Residue 64									
3JHnHa	9.00	8.11	7.51	8.36	8.94	9.46	8.96	10.12	10.10
3JHaHb2	0.90	4.77	3.89	3.89	3.89	4.67	4.14	4.14	4.14
3JHaHb3	0.50	7.37	7.83	7.83	7.83	9.32	10.65	10.65	10.65
3JC'Hb2	2.10	3.03	3.37	3.37	3.37	1.73	1.43	1.43	1.43
3JC'Hb3	8.00	2.00	1.82	1.82	1.82	1.86	1.60	1.60	1.60
Residue 65									
3JHnHa	9.40	6.75	6.07	6.60	7.36	8.28	7.68	8.57	9.34
3JHaHb2	2.10	4.78	4.08	4.08	4.08	4.53	3.62	3.62	3.62
3JHaHb3	13.40	4.67	3.75	3.75	3.75	4.50	3.43	3.43	3.43
3JC'Hb2	8.10	4.87	6.17	6.17	6.17	5.17	6.61	6.61	6.61
3JC'Hb3	1.00	2.19	2.07	2.07	2.07	2.02	1.82	1.82	1.82
Residue 66									
3JHnHa	10.10	8.24	7.65	8.53	8.73	9.11	8.57	9.65	10.14
3JHaHb2	0.10	4.56	3.67	3.67	3.67	4.62	3.97	3.97	3.97
3JHaHb3	2.20	4.51	3.45	3.45	3.45	9.30	10.62	10.62	10.62
3JC'Hb2	1.40	5.13	6.56	6.56	6.56	1.84	1.57	1.57	1.57
3JC'Hb3	10.90	2.07	1.88	1.88	1.88	1.76	1.47	1.47	1.47
Residue 68									
3JHnHa	9.60	5.76	5.03	5.32	5.97	8.92	8.38	9.42	9.35
Residue 69									
3JHaHb2	11.40	6.19	8.08	8.08	8.08	6.19	8.08	8.08	8.08

Residue 74									
3JHaHb2	1.70	4.62	3.62	3.62	3.62	4.35	3.05	3.05	3.05
3JHaHb3	12.60	4.75	3.98	3.98	3.98	4.56	3.80	3.80	3.80
3JC'Hb2	6.60	4.94	6.27	6.27	6.27	5.27	6.76	6.76	6.76
3JC'Hb3	0.10	2.06	1.88	1.88	1.88	1.88	1.62	1.62	1.62
Residue 76									
3JHnHa	8.80	6.67	5.96	6.48	7.29	8.07	7.46	8.30	9.11
Residue 77									
3JHnHa	7.50	6.30	6.16	6.40	5.92	7.05	7.30	7.61	6.21
Residue 78									
3JHnHa	7.60	5.97	5.22	5.57	5.67	8.60	8.03	9.00	8.64
Residue 80									
3JHaHb2	13.80	8.79	9.69	9.69	9.69	9.29	10.60	10.60	10.60
3JHaHb3	1.00	4.82	4.43	4.43	4.43	4.49	3.56	3.56	3.56
3JC'Hb2	2.80	1.95	1.76	1.76	1.76	1.82	1.55	1.55	1.55
3JC'Hb3	0.90	4.81	6.08	6.08	6.08	5.28	6.77	6.77	6.77
Residue 81									
3JHnHa	3.50	3.49	2.60	2.37	3.09	4.12	3.25	3.18	4.02
Residue 82									
3JHnHa	5.80	4.23	3.36	3.31	4.14	5.23	4.42	4.61	5.54
Residue 83									
3JHnHa	7.80	3.23	2.32	2.03	2.70	6.09	5.34	5.73	6.67
Residue 84									
3JHnHa	9.40	4.15	3.29	3.22	4.02	6.90	6.20	6.77	7.67
3JHaHb2	12.00	4.76	3.74	3.74	3.74	7.75	8.10	8.10	8.10
3JHaHb3	0.50	4.83	4.23	4.23	4.23	4.69	4.15	4.15	4.15
3JC'Hb2	2.60	4.76	6.01	6.01	6.01	2.75	2.96	2.96	2.96
3JC'Hb3	1.50	2.13	2.00	2.00	2.00	4.13	5.05	5.05	5.05
Residue 85									
3JHnHa	5.80	5.19	4.39	4.56	5.33	5.60	4.82	5.08	5.87
Residue 86									
3JHnHa	5.70	6.17	5.51	5.89	6.42	5.08	4.27	4.42	5.34
Residue 87									
3JHnHa	8.50	5.03	4.30	4.41	4.89	7.06	6.37	6.98	7.85
3JHaHb3	12.20	5.20	4.77	4.77	4.77	4.39	3.23	3.23	3.23
Residue 88									
3JHaHb2	2.00	6.19	8.08	8.08	8.08	6.19	8.08	8.08	8.08
Residue 89									
3JHaHb2	10.80	6.19	8.08	8.08	8.08	6.19	8.08	8.08	8.08
3JC'Hb2	3.30	3.44	3.76	3.76	3.76	3.44	3.76	3.76	3.76
Residue 90									
3JHnHa	4.50	3.85	2.97	2.83	3.62	4.62	3.77	3.82	4.72
Residue 91									
3JHnHa	5.50	3.42	2.52	2.27	2.98	4.64	3.80	3.84	4.74
Residue 92									
3JHnHa	5.20	4.14	3.27	3.20	4.04	5.30	4.49	4.69	5.63
3JC'Hb2	1.30	3.44	3.76	3.76	3.76	3.44	3.76	3.76	3.76
Residue 93									
3JHnHa	4.60	3.49	2.59	2.36	3.10	4.50	3.65	3.67	4.55
3JHaHb2	13.50	4.81	3.90	3.90	3.90	8.42	9.24	9.24	9.24
3JHaHb3	1.90	4.67	3.94	3.94	3.94	4.80	4.48	4.48	4.48
Residue 94									
3JHnHa	5.40	3.78	2.89	2.73	3.52	5.48	4.69	4.93	5.88
3JHaHb2	2.10	5.08	4.10	4.10	4.10	4.40	3.29	3.29	3.29
3JHaHb3	15.00	4.79	4.34	4.34	4.34	4.41	3.32	3.32	3.32
3JC'Hb2	9.80	4.57	5.72	5.72	5.72	5.31	6.81	6.81	6.81
3JC'Hb3	3.10	2.30	2.27	2.27	2.27	1.96	1.73	1.73	1.73
Residue 96									
3JHnHa	5.20	3.15	2.24	1.93	2.60	4.21	3.35	3.29	4.16
Residue 97									
3JHnHa	6.70	3.53	2.63	2.41	3.15	4.73	3.89	3.96	4.86
Residue 98									
3JHaHb2	15.30	6.19	8.08	8.08	8.08	6.19	8.08	8.08	8.08
3JC'Hb2	1.80	3.44	3.76	3.76	3.76	3.44	3.76	3.76	3.76
Residue 99									
3JHnHa	4.50	4.40	3.55	3.54	4.38	5.62	4.84	5.12	6.06
Residue 101									
3JHnHa	7.20	5.66	4.89	5.17	5.93	6.94	6.24	6.83	7.62
Residue 103									
3JHnHa	8.30	6.15	5.53	5.89	6.05	6.59	5.93	6.41	7.10
Residue 105									
3JHnHa	7.80	4.07	3.24	3.14	3.72	5.64	4.88	5.15	6.01
3JHaHb2	13.00	6.43	6.02	6.02	6.02	7.75	8.17	8.17	8.17
3JHaHb3	2.60	5.25	5.40	5.40	5.40	4.79	4.45	4.45	4.45
3JC'Hb2	1.10	3.31	3.85	3.85	3.85	2.66	2.85	2.85	2.85

3JC'Hb3	1.40	3.09	3.50	3.50	3.50	4.08	4.97	4.97	4.97
Residue 106									
3JHaHb2	13.20	4.82	3.98	3.98	3.98	6.70	6.61	6.61	6.61
3JHaHb3	3.10	5.06	4.53	4.53	4.53	5.16	4.81	4.81	4.81
3JC'Hb2	3.00	4.58	5.73	5.73	5.73	3.19	3.62	3.62	3.62
3JC'Hb3	3.00	2.17	2.05	2.05	2.05	3.39	3.93	3.93	3.93
Residue 107									
3JHnHa	4.40	5.19	4.40	4.57	5.32	4.66	3.82	3.87	4.74
Residue 111									
3JHnHa	7.30	4.52	3.69	3.70	4.49	5.21	4.40	4.58	5.48
3JHaHb2	1.70	5.27	4.64	4.64	4.64	4.45	3.45	3.45	3.45
Residue 112									
3JHnHa	4.60	3.77	2.90	2.73	3.39	4.16	3.29	3.22	4.07
Residue 113									
3JHnHa	6.70	6.15	5.41	5.81	6.59	7.47	6.81	7.52	8.32
Residue 114									
3JHnHa	9.90	7.06	6.38	6.99	7.63	9.25	8.73	9.85	9.97
3JHaHb3	1.80	5.20	5.41	5.41	5.41	4.57	3.73	3.73	3.73
Residue 116									
3JHaHb2	3.40	6.90	6.80	6.80	6.80	4.53	3.43	3.43	3.43
3JHaHb3	9.80	5.30	5.31	5.31	5.31	4.62	3.76	3.76	3.76
3JC'Hb2	8.50	2.94	3.27	3.27	3.27	5.08	6.47	6.47	6.47
3JC'Hb3	2.20	3.46	4.05	4.05	4.05	1.96	1.75	1.75	1.75
Residue 118									
3JHnHa	10.10	6.75	6.10	6.63	7.03	8.03	7.41	8.25	8.60
3JHaHb2	4.90	6.19	8.08	8.08	8.08	6.19	8.08	8.08	8.08
3JC'Hb2	1.80	3.44	3.76	3.76	3.76	3.44	3.76	3.76	3.76
Residue 119									
3JHnHa	6.70	6.31	5.59	6.03	6.71	5.72	4.95	5.24	6.17
3JHaHb2	4.50	4.62	3.63	3.63	3.63	4.53	3.49	3.49	3.49
3JHaHb3	13.30	4.68	3.91	3.91	3.91	4.46	3.43	3.43	3.43
Residue 120									
3JHnHa	4.60	4.96	4.16	4.27	4.98	5.06	4.24	4.39	5.26
3JHaHb2	4.50	6.19	8.08	8.08	8.08	6.19	8.08	8.08	8.08
3JC'Hb2	3.00	3.44	3.76	3.76	3.76	3.44	3.76	3.76	3.76
Residue 121									
3JHnHa	5.10	4.69	3.86	3.92	4.72	4.77	3.93	4.01	4.91
Residue 122									
3JHnHa	4.40	4.57	3.75	3.77	4.49	3.43	2.53	2.28	3.02
Residue 123									
3JHnHa	5.50	4.64	3.92	3.93	4.46	5.48	4.68	4.93	5.86
3JHaHb2	8.60	4.90	3.89	3.89	3.89	9.24	10.51	10.51	10.51
3JHaHb3	0.30	4.83	4.27	4.27	4.27	4.50	3.58	3.58	3.58
3JC'Hb2	1.10	4.69	5.89	5.89	5.89	1.83	1.56	1.56	1.56
3JC'Hb3	1.80	2.23	2.16	2.16	2.16	5.24	6.71	6.71	6.71
Residue 124									
3JHaHb2	4.20	6.19	8.08	8.08	8.08	6.19	8.08	8.08	8.08
3JC'Hb2	0.80	3.44	3.76	3.76	3.76	3.44	3.76	3.76	3.76
Residue 125									
3JHnHa	4.20	5.78	5.17	5.44	5.82	4.37	3.51	3.49	4.35
Residue 127									
3JHnHa	7.50	6.73	6.08	6.59	6.68	6.37	5.64	6.09	6.93
3JHaHb3	2.60	5.62	5.45	5.45	5.45	4.52	3.65	3.65	3.65
3JC'Hb3	0.80	2.40	2.42	2.42	2.42	5.26	6.75	6.75	6.75
Residue 128									
3JHnHa	7.80	7.20	6.54	7.18	7.85	7.16	6.49	7.13	7.93
Residue 129									
3JHnHa	9.00	7.67	7.04	7.78	8.38	7.78	7.26	8.00	8.19

Each *J*-coupling constant was calculated according to Eqs. S9–S20 with parameters in Table S9B and torsions obtained from 20 unique and independent 316-ns^{smt} NPT MD simulations in water at 308 K using a cutoff of 8.0 Å for nonbonded interactions and the Particle Mesh Ewald method to calculate electrostatic interactions of two atoms at separations of >8.0 Å and other conditions specified in Methods and Table S1.

Table S12. Torsion cluster analysis of C14–C38 in the simulations of BPTI and its mutant using FF12MC and FF14SBlm.

Forcefield Temperature	Duration (ns^{smt})	Total number of sets of 5 torsions	Number of clusters identified	Top-3 largest cluster ID	Number of sets of 5 torsions in the cluster	Occurrence of the cluster (%)	Torsion of C38 (degrees of arc)
BPTI (PDB ID: 1PIT)							
FF12MC 309 K	3.16	200	17	1	76	38	48.02
				2	61	31	–71.25
				3	21	11	–87.74
FF12MC 309 K	31.6	2000	26	1	629	31	49.29
				2	610	31	–73.65
				3	164	8	–86.66
FF12MC 309 K	316	20000	42	1	4086	20	50.86
				2	3487	17	–78.38
				3	1449	7	50.41
FF14SBlm 309 K	3.16	200	1	1	200	100	73.43
FF14SBlm 309 K	31.6	2000	1	1	2000	100	71.46
FF14SBlm 309 K	316	20000	11	1	19546	98	69.71
				2	278	1	–83.50
				3	80	0	–54.25
BPTI mutant (PDB ID: 1QLQ)							
FF12MC 290 K	3.16	200	19	1	44	22	55.78
				2	31	16	–87.39
				3	22	11	–44.38
FF12MC 290 K	31.6	2000	20	1	699	35	–73.14
				2	600	30	51.67
				3	151	8	–89.68
FF12MC 290 K	316	20000	24	1	6501	33	–74.62
				2	5991	30	50.18
				3	2302	12	–84.52
FF14SBlm 290 K	3.16	200	1	1	200	100	–58.89
FF14SBlm 290 K	31.6	2000	5	1	1946	97	–59.89
				2	21	1	73.49
				3	18	1	58.38
FF14SBlm 290 K	316	20000	12	1	18721	94	–59.24
				2	372	2	71.75
				3	298	1	–52.89

Torsion of C38 is defined as :38@SG :38@CB :38@CA :38@N and averaged from all members of a cluster.

Table S13. Root mean square deviations and standard errors between experimental and calculated Lipari-Szabo order parameters of backbone N–H bonds in GB3, BPTI, ubiquitin, and lysozyme and the experimentally determined overall tumbling correlation times of the proteins.

Time (ns)	GB3		BPTI		Ubiquitin		Lysozyme	
	Experimental τ_c = 3.3 ns[1]		Experimental τ_c = 2.0 ns[17]		Experimental τ_c = 4.1 ns[18]		Experimental τ_c = 5.7 ns[4]	
	Simulated at 297 K using PDB ID 1P7E		Simulated at 298 K using PDB ID 5PTI		Simulated at 300 K using PDB ID 1UBQ		Simulated at 308 K using PDB ID 4LZT	
	FF12MCsm	FF14SB	FF12MCsm	FF14SB	FF12MCsm	FF14SB	FF12MCsm	FF14SB
0.025	0.080±0.002	0.082±0.003	0.070±0.005	0.074±0.002	0.067±0.005	0.071±0.001	**0.058±0.005**	0.052±0.002
0.05	0.074±0.002	0.074±0.002	**0.069±0.005**	0.070±0.001	**0.066±0.005**	0.067±0.001	0.063±0.005	0.050±0.002
0.1	0.070±0.002	0.065±0.002	0.078±0.006	0.067±0.001	0.070±0.006	0.062±0.001	0.074±0.006	0.051±0.002
0.2	0.067±0.001	0.058±0.003	0.087±0.006	0.065±0.001	0.080±0.006	0.057±0.002	0.089±0.006	0.050±0.002
0.3	0.065±0.002	0.057±0.004	0.090±0.006	0.064±0.001	0.084±0.006	0.054±0.002	0.099±0.006	0.050±0.002
0.4	0.064±0.002	0.055±0.003	0.095±0.007	0.064±0.001	0.090±0.006	0.053±0.001	0.106±0.006	0.050±0.002
0.5	0.062±0.002	0.054±0.004	0.101±0.007	0.064±0.001	0.093±0.005	0.052±0.001	0.114±0.006	0.051±0.002
1	**0.058±0.003**	0.055±0.003	0.118±0.008	0.063±0.002	0.109±0.005	0.048±0.002	0.110±0.006	**0.050±0.002**
2	0.058±0.004	**0.051±0.005**	0.128±0.008	0.062±0.001	0.119±0.005	0.044±0.003	0.130±0.006	0.050±0.002
3	0.061±0.003	0.051±0.005	0.134±0.008	0.061±0.002	0.132±0.005	0.042±0.003	0.144±0.007	0.050±0.002
4	0.060±0.003	0.053±0.005	0.138±0.007	0.061±0.002	0.140±0.006	**0.041±0.003**	0.153±0.007	0.051±0.002
5	0.059±0.003	0.057±0.005	0.143±0.007	0.061±0.002	0.144±0.006	0.041±0.003	0.160±0.007	0.051±0.002
10	0.061±0.003	0.071±0.004	0.152±0.008	0.060±0.001	0.155±0.003	0.041±0.003	0.180±0.007	0.053±0.003
20	0.067±0.004	0.081±0.004	0.159±0.010	**0.058±0.002**	0.168±0.005	0.044±0.004	0.209±0.009	0.060±0.005
30	0.070±0.004	0.081±0.004	0.164±0.010	0.058±0.003	0.185±0.007	0.048±0.004	0.225±0.009	0.064±0.005
40	0.073±0.004	0.069±0.005	0.172±0.010	0.059±0.003	0.200±0.007	0.049±0.004	0.235±0.009	0.066±0.004
50	0.073±0.003	0.080±0.004	0.175±0.011	0.059±0.003	0.210±0.007	0.050±0.004	0.244±0.009	0.067±0.004
100	0.082±0.004	0.079±0.003	—	—	0.242±0.004	0.053±0.004	—	—
316	0.098±0.002	0.091±0.007	—	—	0.280±0.008	0.061±0.002	—	—
632	0.097±0.003	0.097±0.007	—	—	0.297±0.008	0.064±0.002	—	—
948	0.096±0.002	0.102±0.007	—	—	0.298±0.008	0.065±0.002	—	—

τ_c: overall tumbling correlation time. Time: the duration of 20 different and independent molecular dynamics simulations over which the S^2 parameters were calculated. The standard error of each S^2 was obtained from 20 different and independent molecular dynamics simulations. The experimental S^2 parameters of GB3[1], ubiquitin[3], lysozyme[4], and BPTI[2] were obtained from respective supporting information or corresponding authors.

Table S14. Root mean square deviations and standard errors between experimental and calculated crystallographic Cα and Cγ B-factors of GB3, BPTI, ubiquitin, and lysozyme.

B-factor root mean square deviation (RMSD) Å^2								
Time (ns)	GB3 (1IGD; 297 K)		Ubiquitin (1UBQ; 297 K)		BPTI (4PTI; 297 K)		Lysozyme (4LZT; 295K)	
	FF12MCsm	FF14SB	FF12MCsm	FF14SB	FF12MCsm	FF14SB	FF12MCsm	FF14SB
Cα								
0.025	3.7±0.2	4.4±0.1	6.5±0.3	7.8±0.6	5.9±0.4	7.0±0.2	5.3±0.2	6.7±0.1
**0.05**	**3.2±0.2**	**3.7±0.1**	**8±1**	**9±1**	**4.8±0.6**	**6.3±0.6**	**4.2±0.3**	**5.9±0.1**
0.1	3.0±0.3	3.5±0.2	16±2	15±2	7±1	8±2	3.5±0.5	5.4±0.1
0.2	3.6±0.4	3.3±0.2	29±3	22±3	12±2	12±2	4.3±0.6	5.1±0.1
0.3	4.8±0.6	3.2±0.2	35±3	25±3	15±2	13±1	5.1±0.6	4.9±0.1
0.4	5.3±0.5	3.4±0.3	41±3	28±3	19±3	18±2	6.0±0.7	4.9±0.1
0.5	5.6±0.5	3.5±0.5	50±5	33±4	24±4	20±2	7.0±0.7	4.8±0.1
0.6	5.9±0.5	3.6±0.6	—	—	—	—	7.1±0.7	4.8±0.1
0.7	6.2±0.5	3.8±0.7	—	—	—	—	7.9±0.7	4.6±0.1
0.8	6.4±0.6	4.2±0.8	—	—	—	—	8.8±0.7	4.5±0.2
0.9	6.5±0.7	4.6±0.7	—	—	—	—	9.8±0.8	4.5±0.3
1	6.8±0.7	5.2±0.9	—	—	—	—	10.3±0.8	4.5±0.3
2	—	—	—	—	—	—	—	4.4±0.3
3	—	—	—	—	—	—	—	4.4±0.2
4	—	—	—	—	—	—	—	4.3±0.2
5	—	—	—	—	—	—	—	4.2±0.2
6	—	—	—	—	—	—	—	4.3±0.3
7	—	—	—	—	—	—	—	4.4±0.4
8	—	—	—	—	—	—	—	4.6±0.4
9	—	—	—	—	—	—	—	4.7±0.5
10	—	—	—	—	—	—	—	4.7±0.5
15	—	—	—	—	—	—	—	5.2±0.8
20	—	—	—	—	—	—	—	5.4±0.8
Cγ								
0.025	9.7±0.4	10.4±0.3	7.6±0.5	9.5±0.2	9.4±0.4	11.2±0.1	7.3±0.5	9.5±0.1
**0.05**	**9.9±0.7**	**9.1±0.3**	**8±1**	**8.5±0.3**	**9.3±0.6**	**10.3±0.2**	**7.8±0.8**	**8.6±0.2**
0.1	11±1	9,2±0.6	11±1	7.8±0.9	11.0±0.9	9.5±0.2	9.9±1	8.3±0.2
0.2	14±1	8.6±0.5	16±2	8.4±0.9	13.5±0.8	8.9±0.4	13±1	8.3±0.4
0.3	17±1	8.7±0.7	20±2	10±1	16±2	8.5±0.5	15±1	8.5±0.4
0.4	19±1	9±1	25±3	12±2	18±2	8.9±0.5	17±1	8.7±0.4
0.5	20±1	10±1	29±3	14±2	19±2	9.0±0.6	19±1	8.7±0.4
0.6	21±1	10±1	—	—	—	—	19±1	8.8±0.4
0.7	22±1	11±2	—	—	—	—	21±1	8.8±0.7
0.8	22±1	12±2	—	—	—	—	23±1	9.±1
0.9	23±1	13±2	—	—	—	—	24±1	9±1
1	23±1	14±2	—	—	—	—	25±1	9±1
2	—	—	—	—	—	—	—	10.5±0.9
3	—	—	—	—	—	—	—	11.3±0.8
4	—	—	—	—	—	—	—	12.1±0.7
5	—	—	—	—	—	—	—	12.9±0.8
6	—	—	—	—	—	—	—	13.6±0.7
7	—	—	—	—	—	—	—	14.2±0.7
8	—	—	—	—	—	—	—	15.0±0.8
9	—	—	—	—	—	—	—	15±1
10	—	—	—	—	—	—	—	16±1
15	—	—	—	—	—	—	—	18±2
20	—	—	—	—	—	—	—	19±2

Time: the duration of 20 different and independent molecular dynamics simulations over which the B-factors were calculated. The standard error of each B-factor was obtained from 20 different and independent molecular dynamics simulations with conditions detailed in the text. The experimental B-factors of GB3, BPTI, ubiquitin, and lysozyme were obtained from crystal structures of PDB IDs 1IGD, 4PTI, 1UBQ, and 4LZT, respectively.

From: **CASP** casp@predictioncenter.org
Subject: CASPR
Date: May 4, 2006 at 8:19 PM
To: Pang, Yuan P., Ph.D. pang@mayo.edu

Dear Yuan-Ping:

Thanks for your participation in CASP-R. In all, thirteen groups submitted refined models of one or more of the seven CASP-R targets, providing a total of 151 models (61 model 1s), so that there is a lot of interesting data to understand. This message is about doing that.

We have done a preliminary analysis of the results, looking at the improvement (or otherwise) in model accuracy across groups and across targets. The table below shows the best improvement and next best improvement (best by a different group) for each target, model 1s only.

Target best next best
1 -2.9 -2.8
2 -1.9 -0.9
3 -0.5 -0.4
4 -1.0 -1.0
5 -0.6 -0.5
6 +0.1 +0.3
7 -0.4 -0.1

Performance by target varies a great deal, but overall, more than one group reported a significant improvement for five out of the seven targets. We should not over-interpret that, since the full results show a lot of variation within targets, and some best results may just be luck. And of course knowing the answer may some times help. Never-the-less, there clearly are real refinements here. Below are some action items that will enable this group to get the most out of the data. Please read on, and then respond.

1. Before we get into any community discussion of the results we would like to be sure that you agree with our numbers. Below is a table of CA RMSDs and delta RMSDs for your submissions obtained using LGA. Please give us a yes or no as to whether you agree, so we can get on to the interesting bits. Note that for targets 1, 2, 4, and 5 there may be some slight differences, since the experimental structure we used (from the time of CASP6) is not the current one in the PDB. Your results are as follows:

target	grp_mdl	crmsd	mdl-mdl0
TMR01	G27_1	1.722	-2.853

2. We are very keen to have as productive a discussion as possible of the results, but also keen to keep it non-competitive. If you feel you do not want your numbers released, we will respect that, but hope you will still take part in the discussion. Please let us know.

3. Performance by groups varies a good deal, which is expected, since you are all at different stages of development. There are also a few complicating factors – some people sent in their lowest energy structure as model 1, while others sent in their lowest RMSD structure, and these are often not the same. Please tell us the model number of your lowest energy structure and your lowest RMSD structure for each target. (Even if these numbers are greater than the number of models you sent). Please also indicate how you ranked your models.

4. In order to make maximum use of the expeience, we would like each group to go the FORCASP web site, and write a little about your experience, as a way of kicking off discussion. Please address at least some of the following:

A. Why were some targets relatively easy, some harder? For example, for target 6, we have now discovered that there are a large number of strange geometries (D amino acids, cis main chain conformations) in the starting model that may have caused difficulties. Nobody had any success with this target. At the other end of the scale, for target 1, five groups made substantial improvements. This may be because a major part of the error lay in the termini.

B. If you did not do all targets, how did choose the ones suitable for you?

C. How well did your energy function perform in picking the lowest RMSD structure?

D. Would more computer resources or time have made a difference? E.g. would longer trajectories have helped or hindered?

E. What did you learn – will this experience lead to improved performance next time, do you think? Is so, how?

F. Any comments on the CASP-R experiment, and lessons for target choice and refinement assessment in CASP7.

G. General comments on the nature of the refinement problem, as you currently understand it.

H. And… any thing else you think is appropriate and informative to each other and to us.

OK, enough for now. Please do respond and react to this message. And thanks for your help in making CASP-R worthwhile.

CASP-R organizers,
Andriy, Krzysztof and John.

References:

1. Hall, J. B. & Fushman, D. Characterization of the overall and local dynamics of a protein with intermediate rotational anisotropy: Differentiating between conformational exchange and anisotropic diffusion in the B3 domain of protein G. *J. Biomol. NMR* **27**, 261-275 (2003).
2. Beeser, S. A., Oas, T. G. & Goldenberg, D. P. Determinants of backbone dynamics in native BPTI: Cooperative influence of the 14-38 disulfide and the Tyr35 side-chain. *J. Mol. Biol.* **284**, 1581–1596 (1998).
3. Tjandra, N., Feller, S. E., Pastor, R. W. & Bax, A. Rotational diffusion anisotropy of human ubiquitin from N-15 NMR relaxation. *J. Am. Chem. Soc.* **117**, 12562–12566 (1995).
4. Buck, M. *et al.* Structural determinants of protein dynamics: Analysis of N-15 NMR relaxation measurements for main-chain and side-chain nuclei of hen egg-white lysozyme. *Biochemistry* **34**, 4041–4055 (1995).
5. Graf, J., Nguyen, P. H., Stock, G. & Schwalbe, H. Structure and dynamics of the homologous series of alanine peptides: A joint molecular dynamics/NMR study. *J. Am. Chem. Soc.* **129**, 1179–1189 (2007).
6. Hu, J. S. & Bax, A. Determination of φ and χ_1 angles in proteins from ^{13}C–^{13}C three-bond J couplings measured by three-dimensional heteronuclear NMR. How planar is the peptide bond? *J. Am. Chem. Soc.* **119**, 6360–6368 (1997).
7. Wirmer, J. & Schwalbe, H. Angular dependence of $^1J(N_i,C_{\alpha i})$ and $^2J(N_i,C_{\alpha(i-1)})$ coupling constants measured in J-modulated HSQCs. *J. Biomol. NMR* **23**, 47–55 (2002).
8. Ding, K. & Gronenborn, A. M. Protein Backbone $^1H^N$-$^{13}C^\alpha$ and ^{15}N-$^{13}C^\alpha$ residual dipolar and *J* couplings: New constraints for NMR structure determination. *J. Am. Chem. Soc.* **126**, 6232–6233 (2004).
9. Hennig, M., Bermel, W., Schwalbe, H. & Griesinger, C. Determination of Ψ torsion angle restraints from $^3J(C_\alpha,C_\alpha)$ and $^3J(C_\alpha,H^N)$ coupling constants in proteins. *J. Am. Chem. Soc.* **122**, 6268–6277 (2000).
10. Schmidt, J. M., Blumel, M., Lohr, F. & Ruterjans, H. Self-consistent ^3J coupling analysis for the joint calibration of Karplus coefficients and evaluation of torsion angles. *J. Biomol. NMR* **14**, 1–12 (1999).
11. Case, D. A., Scheurer, C. & Bruschweiler, R. Static and dynamic effects on vicinal scalar J couplings in proteins and peptides: A MD/DFT analysis. *J. Am. Chem. Soc.* **122**, 10390–10397 (2000).
12. Perez, C., Lohr, F., Ruterjans, H. & Schmidt, J. M. Self-consistent Karplus parametrization of ^3J couplings depending on the polypeptide side-chain torsion X_1. *J. Am. Chem. Soc.* **123**, 7081–7093 (2001).
13. Chou, J. J., Case, D. A. & Bax, A. Insights into the mobility of methyl-bearing side chains in proteins from $^3J_{CC}$ and $^3J_{CN}$ couplings. *J. Am. Chem. Soc.* **125**, 8959–8966 (2003).
14. Robertson, M. J., Tirado-Rives, J. & Jorgensen, W. L. Improved peptide and protein torsional energetics with the OPLS-AA force field. *J. Chem. Theory Comput.* **11**, 3499–3509 (2015).
15. Lindorff-Larsen, K. *et al.* Improved side-chain torsion potentials for the AMBER ff99SB protein force field. *Proteins* **78**, 1950–1958 (2010).
16. Smith, L. J., Sutcliffe, M. J., Redfield, C. & Dobson, C. M. *Biochemistry* **30**, 986–996 (1991).
17. Szyperski, T., Luginbuhl, P., Otting, G., Guntert, P. & Wuthrich, K. Protein dynamics studied by rotating frame ^{15}N spin relaxation times. *J. Biomol. NMR* **3**, 151–164 (1993).
18. Tjandra, N., Szabo, A. & Bax, A. Protein backbone dynamics and N-15 chemical shift anisotropy from quantitative measurement of relaxation interference effects. *J. Am. Chem. Soc.* **118**, 6986–6991 (1996).
