# Supplementary material for: FF12MC: A revised AMBER forcefield and new protein simulation protocol
Source: Proteins. 2016 Jul 21;84(10):1490–516. doi: 10.1002/prot.25094 (PMC5129589; doi:10.1002/prot.25094)
Supplement: Supplementary file 3 — Supporting Information [file PROT-84-1490-s003.pdf]

Supporting Figures

FF12MC: A revised AMBER forcefield and new protein simulation protocol

Yuan-Ping Pang

Computer-Aided Molecular Design Laboratory, Mayo Clinic, Rochester, MN 55905, USA

Fig. S1. Smoothed time series of CαβRMSD from NMR structure for chignolin, CLN025, Ac-(AAQAA)$_3$-NH$_2$, and Trp-cage. Ac-(AAQAA)$_3$-NH$_2$, the native state population, and the initial folding time are abbreviated as AAQAA, NSP, and IFT, respectively. Time step (Δt) and IFT are all converted to the standard-mass time. **A**: Folding AAQAA using FF12MC at 274, 300, and 310 K, ***Pages 2–4***. **B**: Folding AAQAA using FF12SB at 274, 300, and 310 K, ***Pages 5–7***. **C**: Folding AAQAA using FF14SB at 274 K, ***Page 8***. **D**: Folding chignolin and CLN025 using FF12MC at 277 and 300 K, ***Pages 9–12***. **E**: Folding chignolin and CLN025 using FF12SB at 277 K, ***Page 13***. **F**: Folding chignolin and CLN025 using FF14SB at 277 K, ***Pages 14–15***. **G**: Folding Trp-cage (TC10b) using FF12MC at 280 K, ***Pages 16–17***.

Fig. S2. Time series of mean square deviation (χ^2) between eight experimental *J*-coupling constants and the corresponding ones calculated from NPT MD simulations under different conditions, ***Page 18***.

Fig. S3. Time series of radius of gyration of GB3, BPTI, ubiquitin, and lysozyme each of which derived from 20 simulations using FF12MC or FF14SB. ***Pages 19–26***.

Fig. S4. Time series of CαRMSD and GDT-HA of the refined CASPR model TMR01 using FF12MC or FF14SBlm. The TMR01 model was refined with 20 unbiased and unrestricted 100-million–timestep of NPT MD simulations at 340K and Δt = 1.00 fs^{wct} using FF12MC or FF14SBlm. The CαRMSD and GDT-HA score were calculated relative to the crystal structure of Protein Data Bank of 1XE1. ***Page 27***.

Fig. S1A

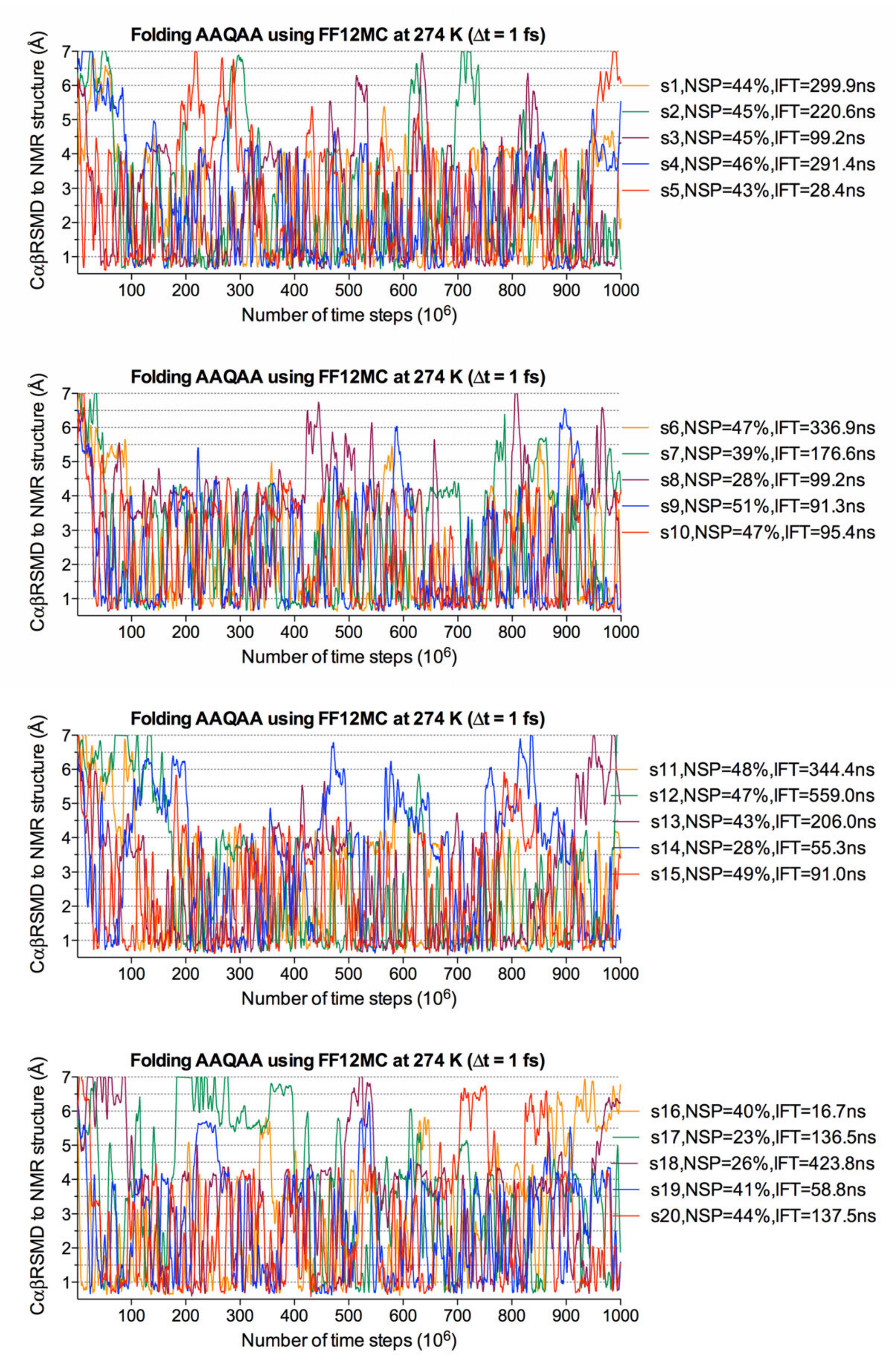
Folding AAQAA using FF12MC at 274 K (Δt = 1 fs)
CαβRSMD to NMR structure (Å)
Number of time steps (10^6)
s1,NSP=44%,IFT=299.9ns
s2,NSP=45%,IFT=220.6ns
s3,NSP=45%,IFT=99.2ns
s4,NSP=46%,IFT=291.4ns
s5,NSP=43%,IFT=28.4ns
Folding AAQAA using FF12MC at 274 K (Δt = 1 fs)
CαβRSMD to NMR structure (Å)
Number of time steps (10^6)
s6,NSP=47%,IFT=336.9ns
s7,NSP=39%,IFT=176.6ns
s8,NSP=28%,IFT=99.2ns
s9,NSP=51%,IFT=91.3ns
s10,NSP=47%,IFT=95.4ns
Folding AAQAA using FF12MC at 274 K (Δt = 1 fs)
CαβRSMD to NMR structure (Å)
Number of time steps (10^6)
s11,NSP=48%,IFT=344.4ns
s12,NSP=47%,IFT=559.0ns
s13,NSP=43%,IFT=206.0ns
s14,NSP=28%,IFT=55.3ns
s15,NSP=49%,IFT=91.0ns
Folding AAQAA using FF12MC at 274 K (Δt = 1 fs)
CαβRSMD to NMR structure (Å)
Number of time steps (10^6)
s16,NSP=40%,IFT=16.7ns
s17,NSP=23%,IFT=136.5ns
s18,NSP=26%,IFT=423.8ns
s19,NSP=41%,IFT=58.8ns
s20,NSP=44%,IFT=137.5ns

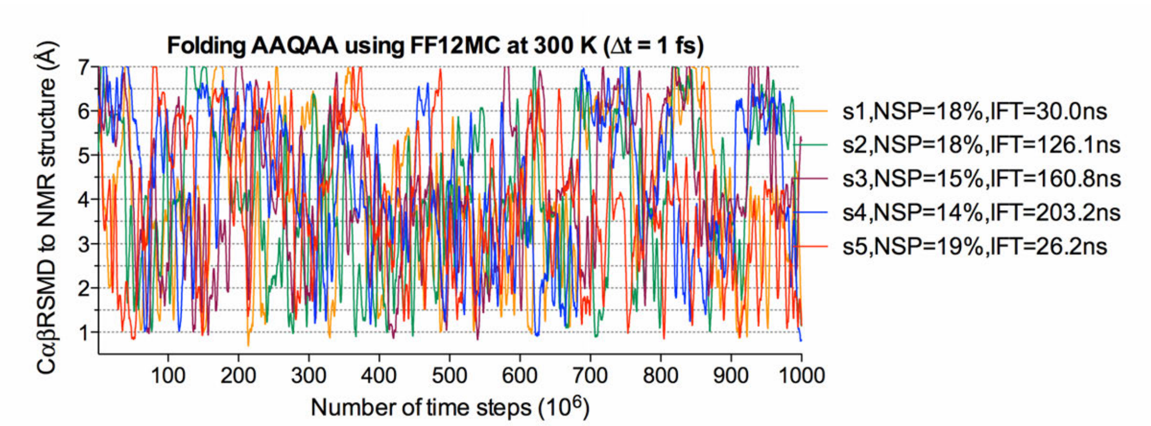
Folding AAQAA using FF12MC at 300 K (Δt = 1 fs)
CαβRSMD to NMR structure (Å)
Number of time steps (10^6)
s1,NSP=18%,IFT=30.0ns
s2,NSP=18%,IFT=126.1ns
s3,NSP=15%,IFT=160.8ns
s4,NSP=14%,IFT=203.2ns
s5,NSP=19%,IFT=26.2ns

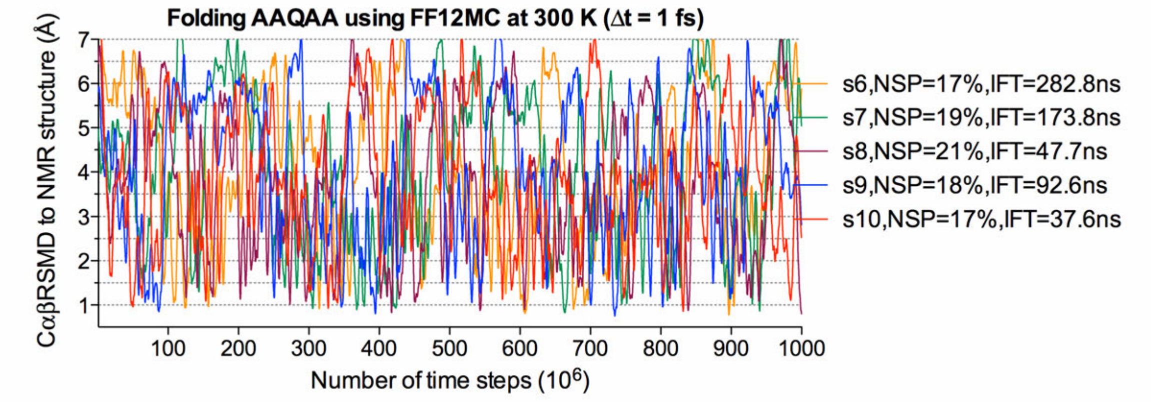
Folding AAQAA using FF12MC at 300 K (Δt = 1 fs)
CαβRSMD to NMR structure (Å)
Number of time steps (10^6)
s6,NSP=17%,IFT=282.8ns
s7,NSP=19%,IFT=173.8ns
s8,NSP=21%,IFT=47.7ns
s9,NSP=18%,IFT=92.6ns
s10,NSP=17%,IFT=37.6ns

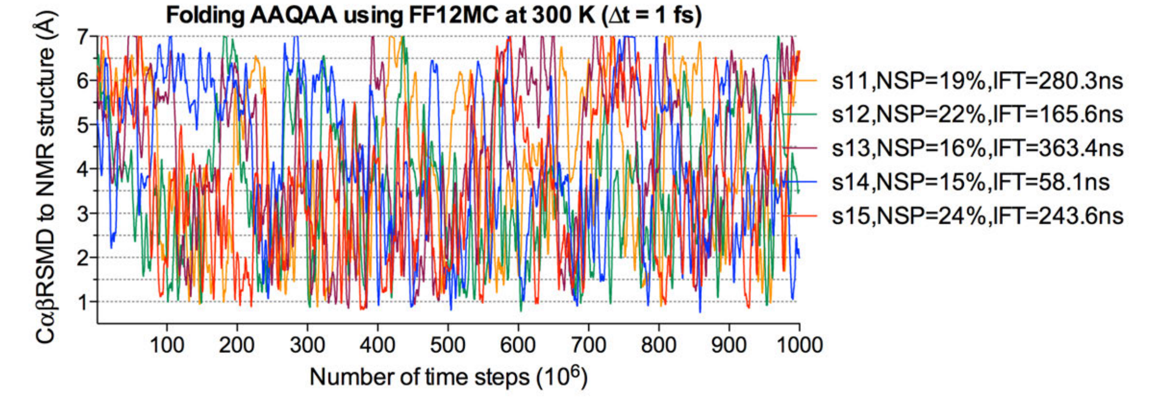
Folding AAQAA using FF12MC at 300 K (Δt = 1 fs)
CαβRSMD to NMR structure (Å)
Number of time steps (10^6)
s11,NSP=19%,IFT=280.3ns
s12,NSP=22%,IFT=165.6ns
s13,NSP=16%,IFT=363.4ns
s14,NSP=15%,IFT=58.1ns
s15,NSP=24%,IFT=243.6ns

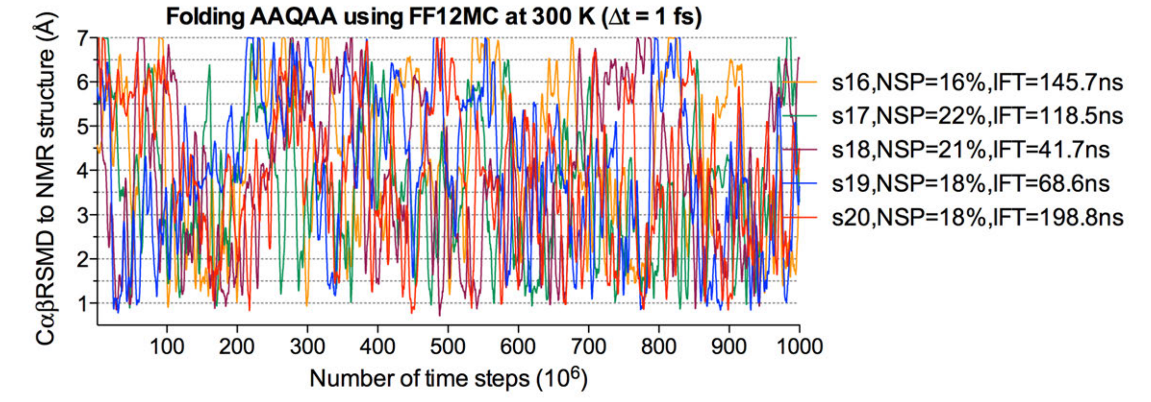
Folding AAQAA using FF12MC at 300 K (Δt = 1 fs)
CαβRSMD to NMR structure (Å)
Number of time steps (10^6)
s16,NSP=16%,IFT=145.7ns
s17,NSP=22%,IFT=118.5ns
s18,NSP=21%,IFT=41.7ns
s19,NSP=18%,IFT=68.6ns
s20,NSP=18%,IFT=198.8ns

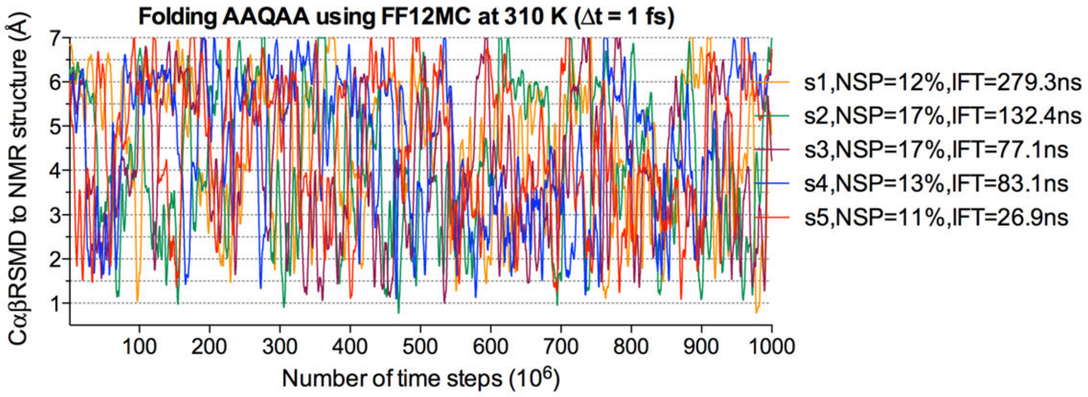
Folding AAQAA using FF12MC at 310 K (Δt = 1 fs)
CαβRSMD to NMR structure (Å)
Number of time steps (10^6)
s1,NSP=12%,IFT=279.3ns
s2,NSP=17%,IFT=132.4ns
s3,NSP=17%,IFT=77.1ns
s4,NSP=13%,IFT=83.1ns
s5,NSP=11%,IFT=26.9ns

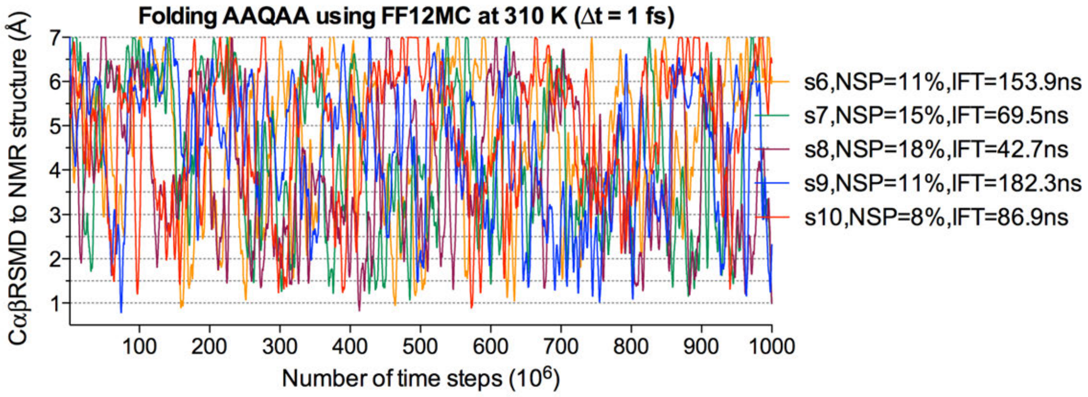
Folding AAQAA using FF12MC at 310 K (Δt = 1 fs)
CαβRSMD to NMR structure (Å)
Number of time steps (10^6)
s6,NSP=11%,IFT=153.9ns
s7,NSP=15%,IFT=69.5ns
s8,NSP=18%,IFT=42.7ns
s9,NSP=11%,IFT=182.3ns
s10,NSP=8%,IFT=86.9ns

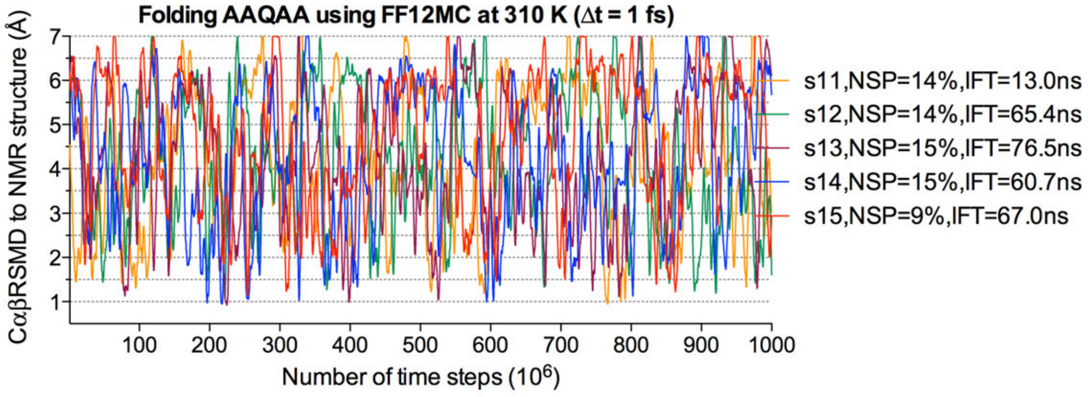
Folding AAQAA using FF12MC at 310 K (Δt = 1 fs)
CαβRSMD to NMR structure (Å)
Number of time steps (10^6)
s11,NSP=14%,IFT=13.0ns
s12,NSP=14%,IFT=65.4ns
s13,NSP=15%,IFT=76.5ns
s14,NSP=15%,IFT=60.7ns
s15,NSP=9%,IFT=67.0ns

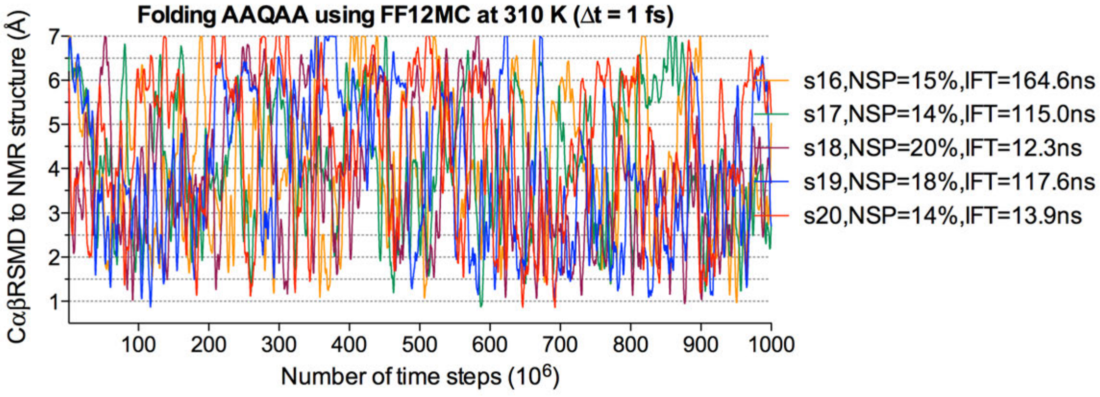
Folding AAQAA using FF12MC at 310 K (Δt = 1 fs)
CαβRSMD to NMR structure (Å)
Number of time steps (10^6)
s16,NSP=15%,IFT=164.6ns
s17,NSP=14%,IFT=115.0ns
s18,NSP=20%,IFT=12.3ns
s19,NSP=18%,IFT=117.6ns
s20,NSP=14%,IFT=13.9ns

Fig. S1*B*

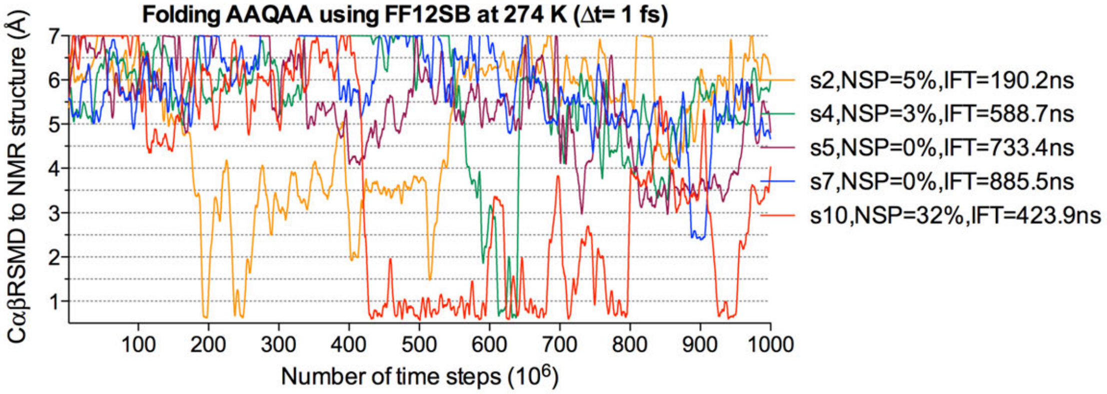

Folding AAQAA using FF12SB at 274 K (Δt= 1 fs)
CαβRSMD to NMR structure (Å)
Number of time steps (10^6)
s2,NSP=5%,IFT=190.2ns
s4,NSP=3%,IFT=588.7ns
s5,NSP=0%,IFT=733.4ns
s7,NSP=0%,IFT=885.5ns
s10,NSP=32%,IFT=423.9ns

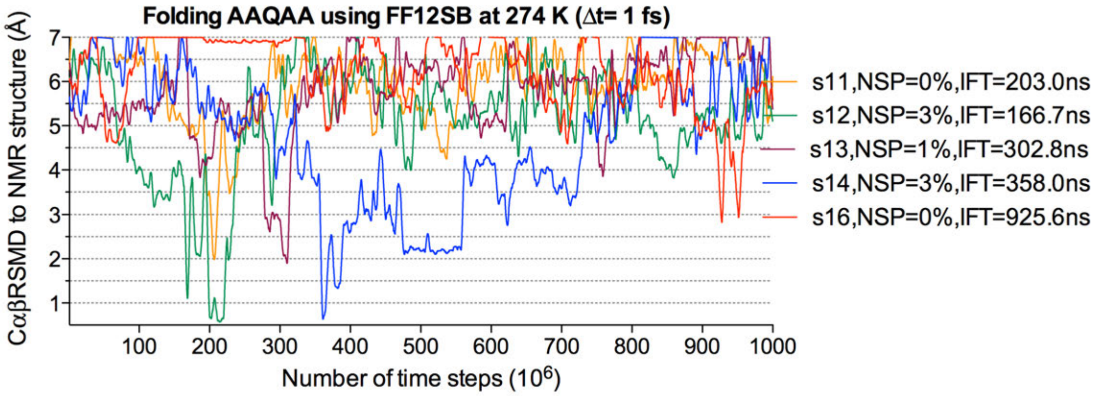

Folding AAQAA using FF12SB at 274 K (Δt= 1 fs)
CαβRSMD to NMR structure (Å)
Number of time steps (10^6)
s11,NSP=0%,IFT=203.0ns
s12,NSP=3%,IFT=166.7ns
s13,NSP=1%,IFT=302.8ns
s14,NSP=3%,IFT=358.0ns
s16,NSP=0%,IFT=925.6ns

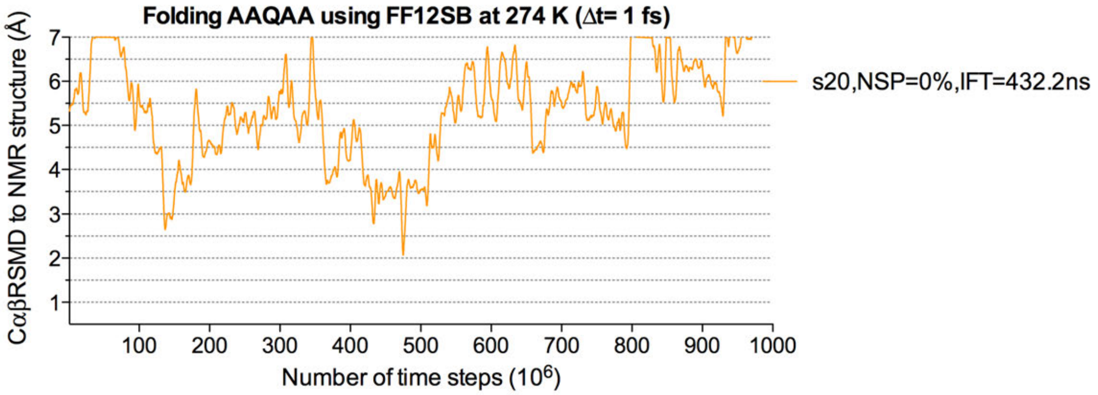

Folding AAQAA using FF12SB at 274 K (Δt= 1 fs)
CαβRSMD to NMR structure (Å)
Number of time steps (10^6)
s20,NSP=0%,IFT=432.2ns

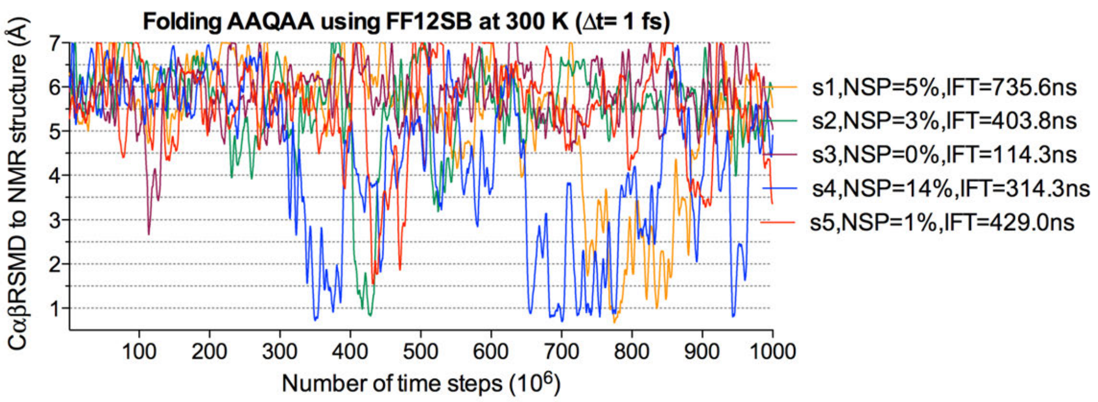
Folding AAQAA using FF12SB at 300 K (Δt= 1 fs)
CαβRSMD to NMR structure (Å)
Number of time steps (10^6)
s1,NSP=5%,IFT=735.6ns
s2,NSP=3%,IFT=403.8ns
s3,NSP=0%,IFT=114.3ns
s4,NSP=14%,IFT=314.3ns
s5,NSP=1%,IFT=429.0ns

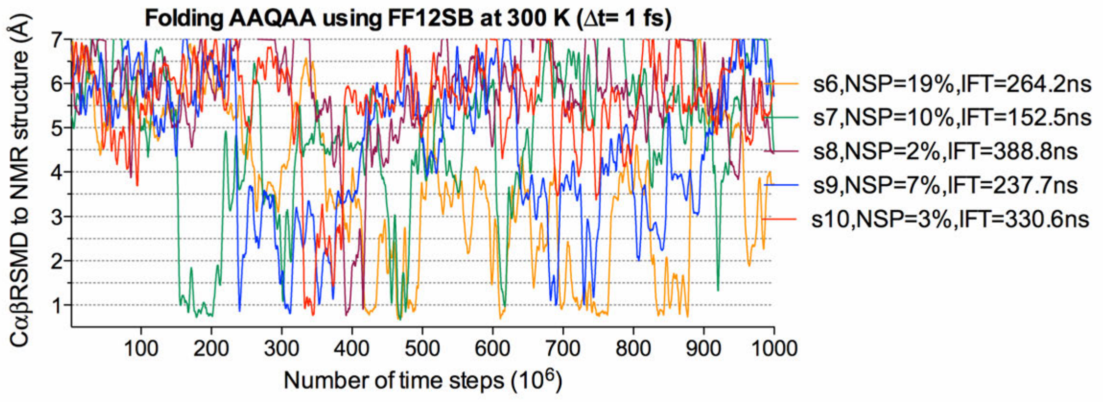
Folding AAQAA using FF12SB at 300 K (Δt= 1 fs)
CαβRSMD to NMR structure (Å)
Number of time steps (10^6)
s6,NSP=19%,IFT=264.2ns
s7,NSP=10%,IFT=152.5ns
s8,NSP=2%,IFT=388.8ns
s9,NSP=7%,IFT=237.7ns
s10,NSP=3%,IFT=330.6ns

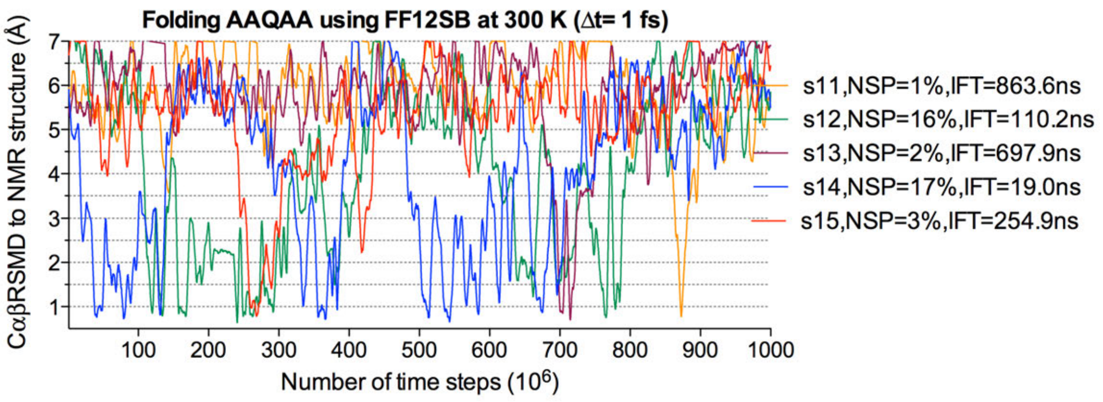
Folding AAQAA using FF12SB at 300 K (Δt= 1 fs)
CαβRSMD to NMR structure (Å)
Number of time steps (10^6)
s11,NSP=1%,IFT=863.6ns
s12,NSP=16%,IFT=110.2ns
s13,NSP=2%,IFT=697.9ns
s14,NSP=17%,IFT=19.0ns
s15,NSP=3%,IFT=254.9ns

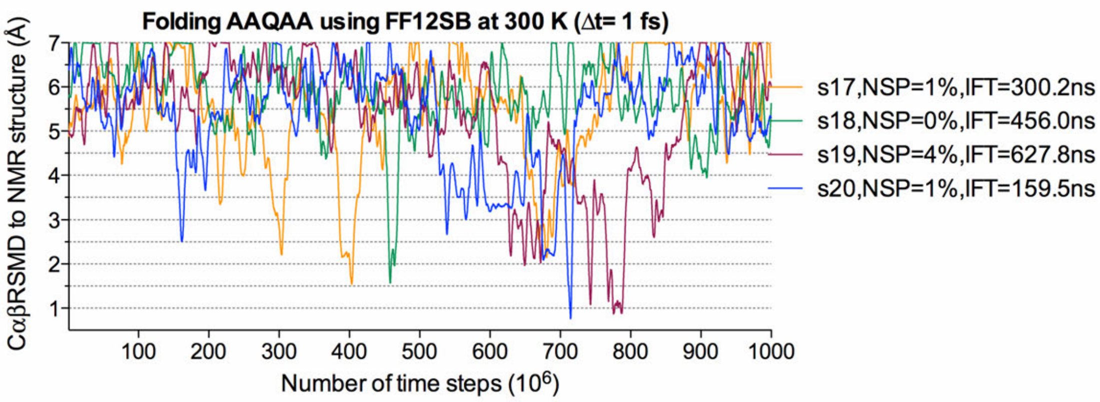
Folding AAQAA using FF12SB at 300 K (Δt= 1 fs)
CαβRSMD to NMR structure (Å)
Number of time steps (10^6)
s17,NSP=1%,IFT=300.2ns
s18,NSP=0%,IFT=456.0ns
s19,NSP=4%,IFT=627.8ns
s20,NSP=1%,IFT=159.5ns

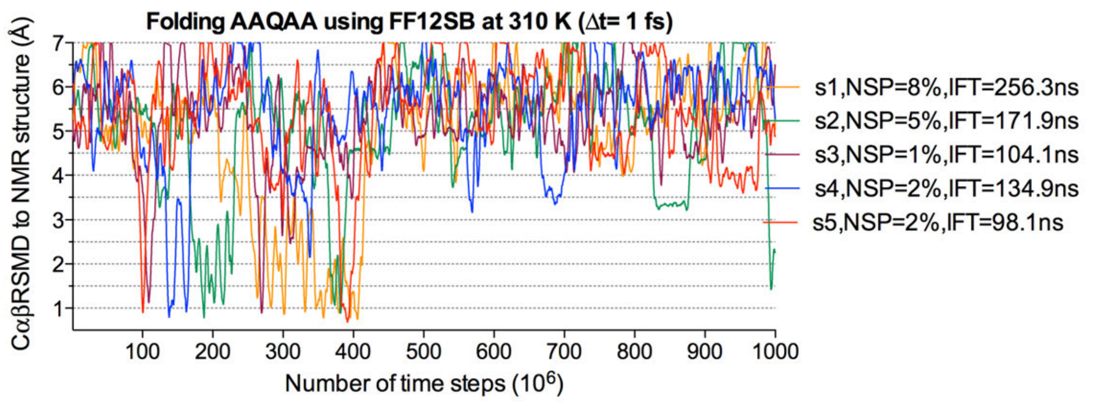
Folding AAQAA using FF12SB at 310 K (Δt= 1 fs)
CαβRSMD to NMR structure (Å)
Number of time steps (10^6)
s1,NSP=8%,IFT=256.3ns
s2,NSP=5%,IFT=171.9ns
s3,NSP=1%,IFT=104.1ns
s4,NSP=2%,IFT=134.9ns
s5,NSP=2%,IFT=98.1ns

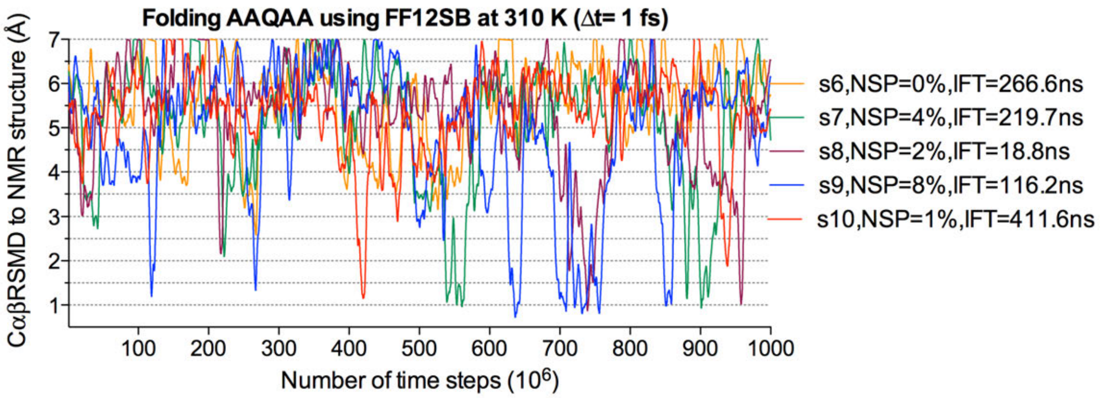
Folding AAQAA using FF12SB at 310 K (Δt= 1 fs)
CαβRSMD to NMR structure (Å)
Number of time steps (10^6)
s6,NSP=0%,IFT=266.6ns
s7,NSP=4%,IFT=219.7ns
s8,NSP=2%,IFT=18.8ns
s9,NSP=8%,IFT=116.2ns
s10,NSP=1%,IFT=411.6ns

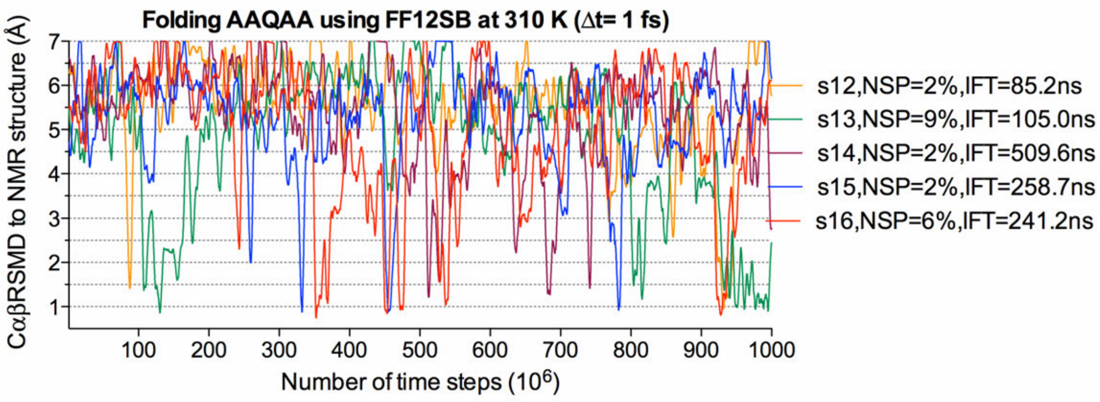
Folding AAQAA using FF12SB at 310 K (Δt= 1 fs)
CαβRSMD to NMR structure (Å)
Number of time steps (10^6)
s12,NSP=2%,IFT=85.2ns
s13,NSP=9%,IFT=105.0ns
s14,NSP=2%,IFT=509.6ns
s15,NSP=2%,IFT=258.7ns
s16,NSP=6%,IFT=241.2ns

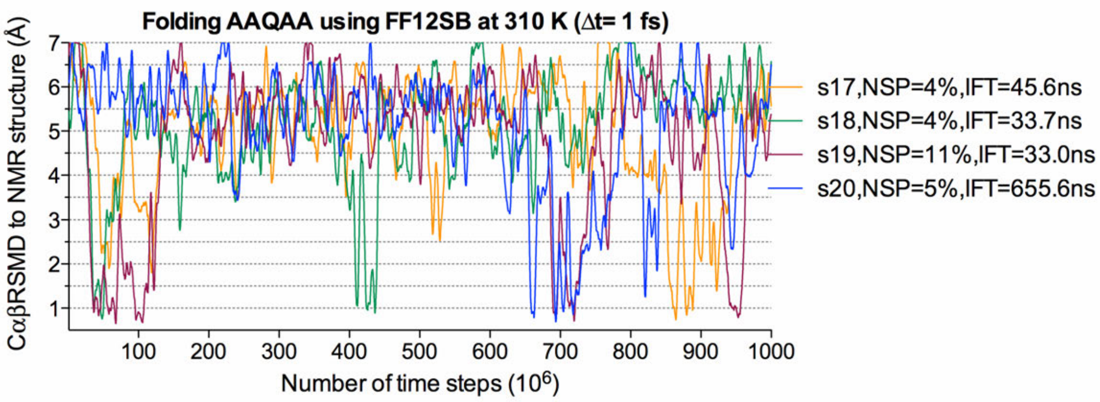
Folding AAQAA using FF12SB at 310 K (Δt= 1 fs)
CαβRSMD to NMR structure (Å)
Number of time steps (10^6)
s17,NSP=4%,IFT=45.6ns
s18,NSP=4%,IFT=33.7ns
s19,NSP=11%,IFT=33.0ns
s20,NSP=5%,IFT=655.6ns

Fig. S1C

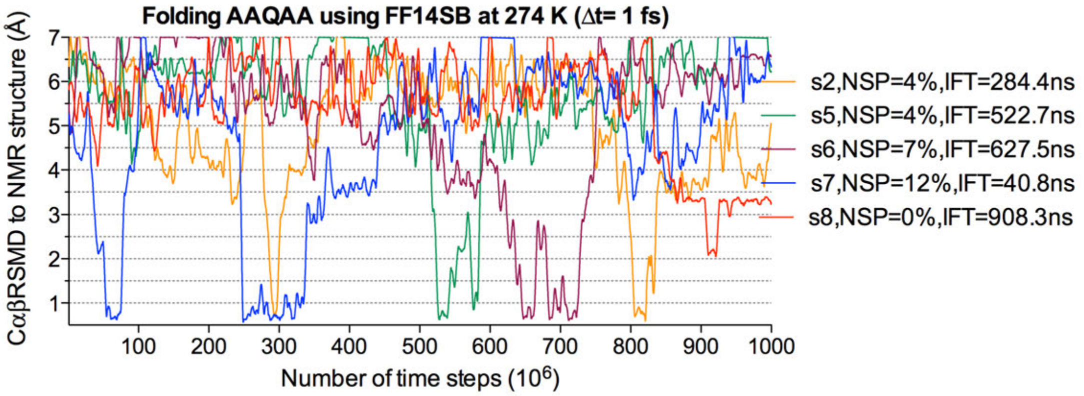

Folding AAQAA using FF14SB at 274 K (Δt= 1 fs)
CαβRSMD to NMR structure (Å)
Number of time steps (10^6)
s2,NSP=4%,IFT=284.4ns
s5,NSP=4%,IFT=522.7ns
s6,NSP=7%,IFT=627.5ns
s7,NSP=12%,IFT=40.8ns
s8,NSP=0%,IFT=908.3ns

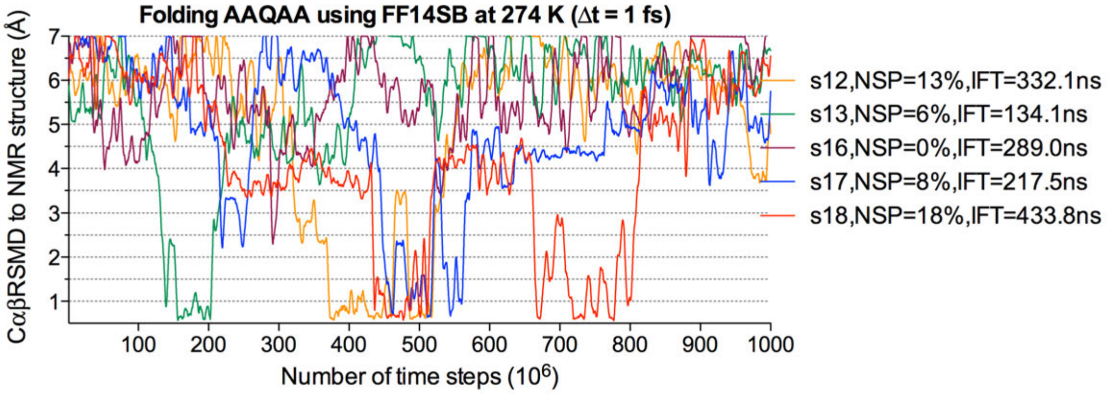

Folding AAQAA using FF14SB at 274 K (Δt = 1 fs)
CαβRSMD to NMR structure (Å)
Number of time steps (10^6)
s12,NSP=13%,IFT=332.1ns
s13,NSP=6%,IFT=134.1ns
s16,NSP=0%,IFT=289.0ns
s17,NSP=8%,IFT=217.5ns
s18,NSP=18%,IFT=433.8ns

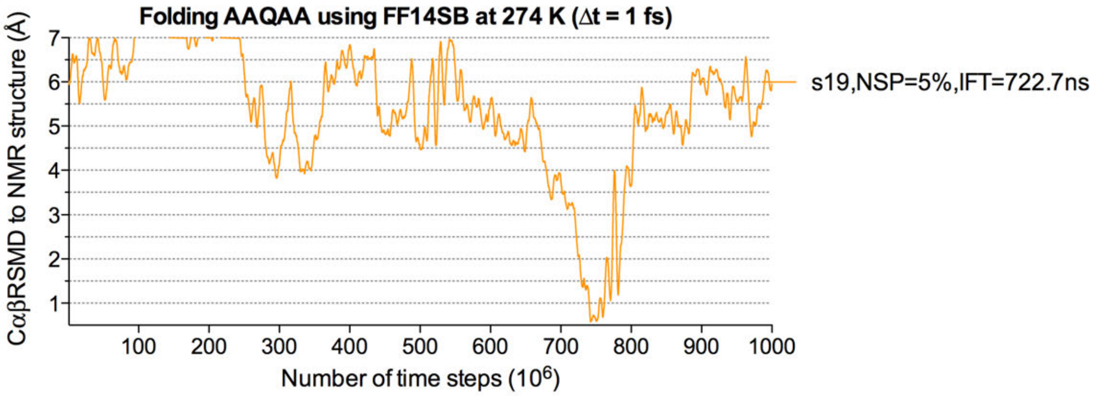

Folding AAQAA using FF14SB at 274 K (Δt = 1 fs)
CαβRSMD to NMR structure (Å)
Number of time steps (10^6)
s19,NSP=5%,IFT=722.7ns

Fig. S1*D*

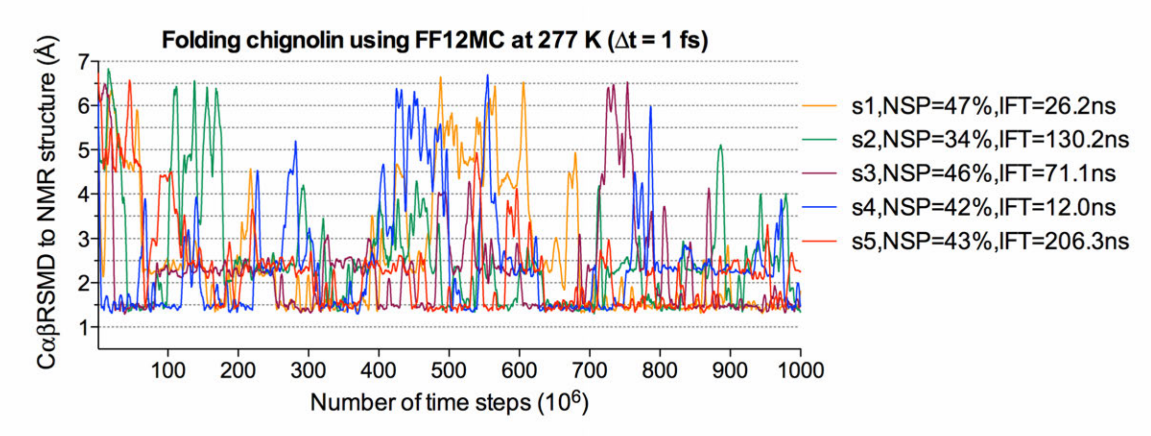
Folding chignolin using FF12MC at 277 K (Δt = 1 fs)
CαβRSMD to NMR structure (Å)
Number of time steps (10^6)
s1,NSP=47%,IFT=26.2ns
s2,NSP=34%,IFT=130.2ns
s3,NSP=46%,IFT=71.1ns
s4,NSP=42%,IFT=12.0ns
s5,NSP=43%,IFT=206.3ns

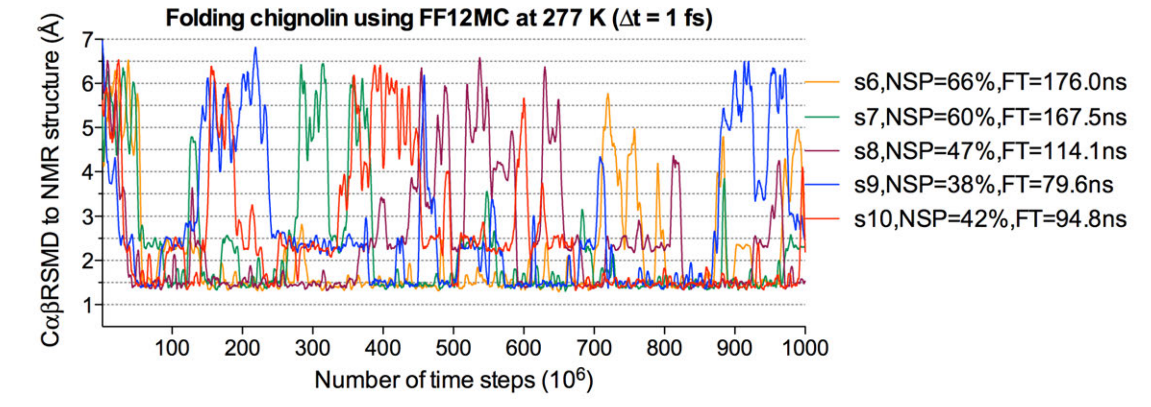
Folding chignolin using FF12MC at 277 K (Δt = 1 fs)
CαβRSMD to NMR structure (Å)
Number of time steps (10^6)
s6,NSP=66%,FT=176.0ns
s7,NSP=60%,FT=167.5ns
s8,NSP=47%,FT=114.1ns
s9,NSP=38%,FT=79.6ns
s10,NSP=42%,FT=94.8ns

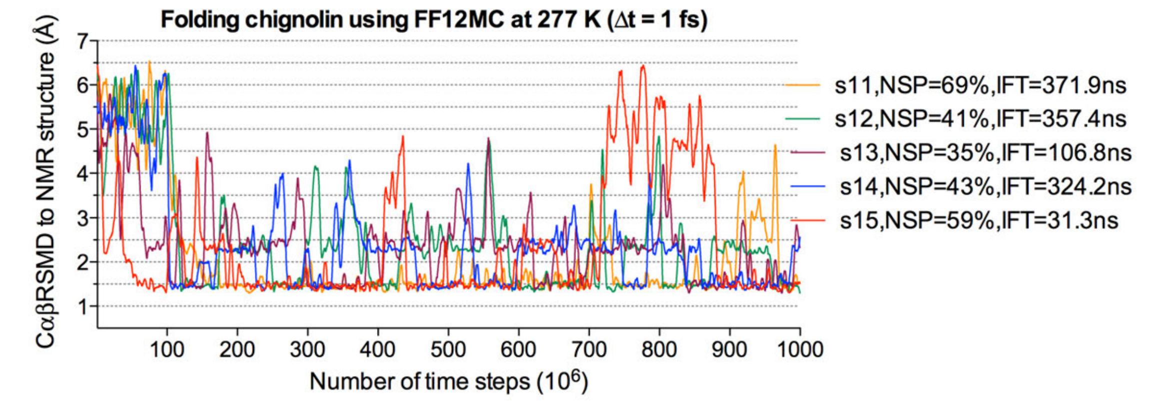
Folding chignolin using FF12MC at 277 K (Δt = 1 fs)
CαβRSMD to NMR structure (Å)
Number of time steps (10^6)
s11,NSP=69%,IFT=371.9ns
s12,NSP=41%,IFT=357.4ns
s13,NSP=35%,IFT=106.8ns
s14,NSP=43%,IFT=324.2ns
s15,NSP=59%,IFT=31.3ns

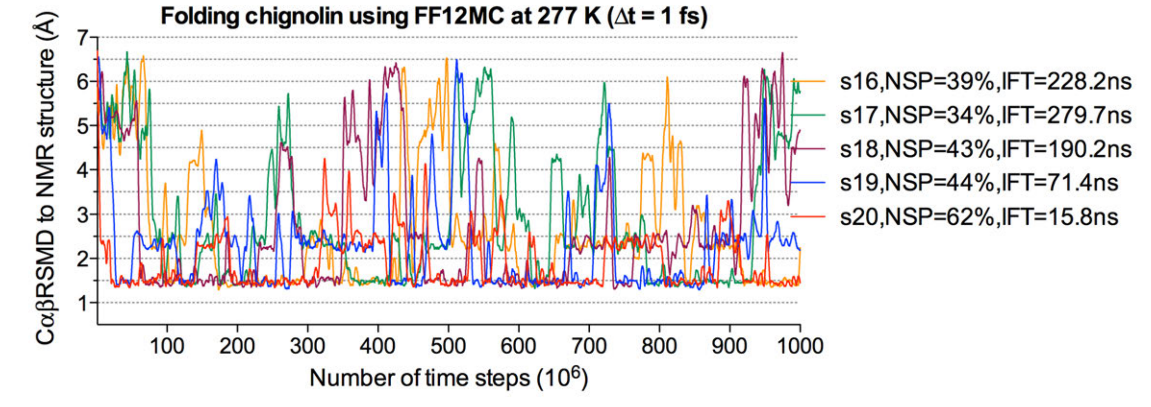
Folding chignolin using FF12MC at 277 K (Δt = 1 fs)
CαβRSMD to NMR structure (Å)
Number of time steps (10^6)
s16,NSP=39%,IFT=228.2ns
s17,NSP=34%,IFT=279.7ns
s18,NSP=43%,IFT=190.2ns
s19,NSP=44%,IFT=71.4ns
s20,NSP=62%,IFT=15.8ns

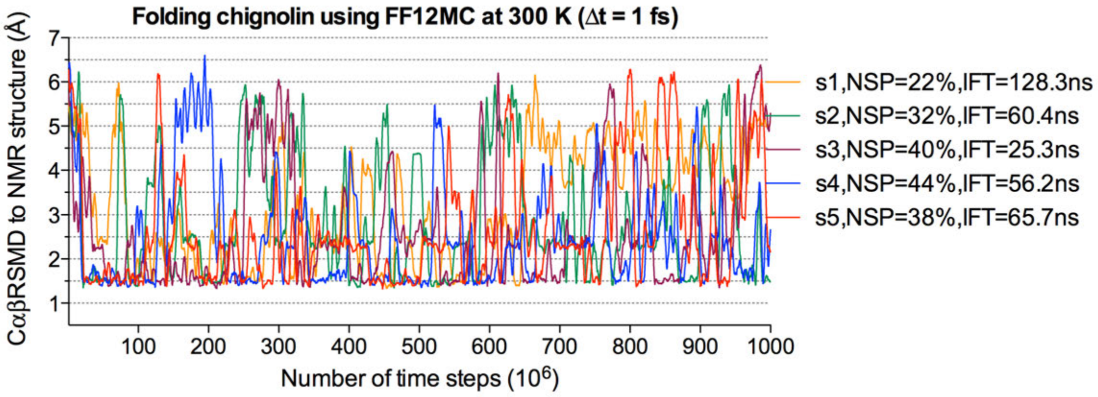
Folding chignolin using FF12MC at 300 K (Δt = 1 fs)
CαβRSMD to NMR structure (Å)
Number of time steps (10^6)
s1,NSP=22%,IFT=128.3ns
s2,NSP=32%,IFT=60.4ns
s3,NSP=40%,IFT=25.3ns
s4,NSP=44%,IFT=56.2ns
s5,NSP=38%,IFT=65.7ns

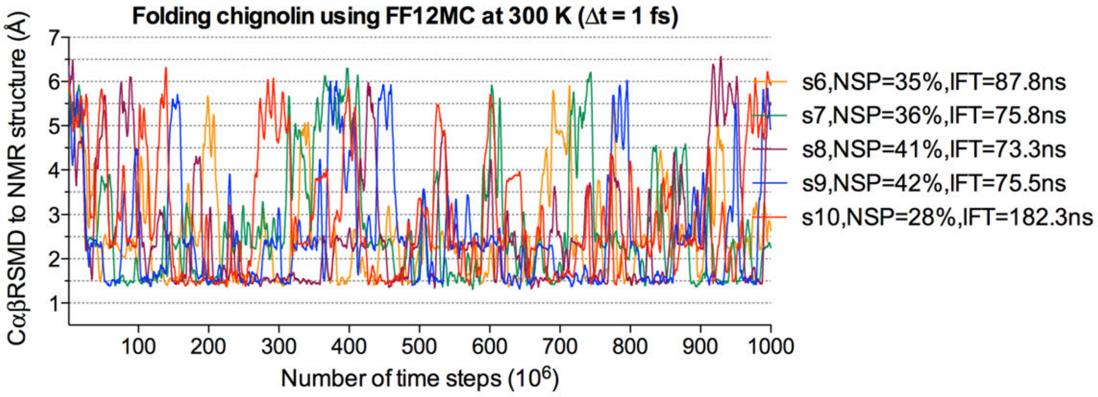
Folding chignolin using FF12MC at 300 K (Δt = 1 fs)
CαβRSMD to NMR structure (Å)
Number of time steps (10^6)
s6,NSP=35%,IFT=87.8ns
s7,NSP=36%,IFT=75.8ns
s8,NSP=41%,IFT=73.3ns
s9,NSP=42%,IFT=75.5ns
s10,NSP=28%,IFT=182.3ns

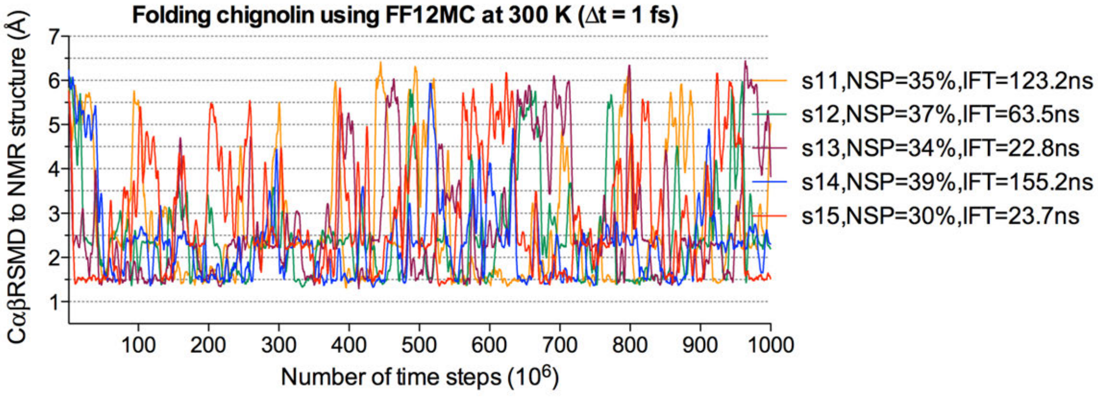
Folding chignolin using FF12MC at 300 K (Δt = 1 fs)
CαβRSMD to NMR structure (Å)
Number of time steps (10^6)
s11,NSP=35%,IFT=123.2ns
s12,NSP=37%,IFT=63.5ns
s13,NSP=34%,IFT=22.8ns
s14,NSP=39%,IFT=155.2ns
s15,NSP=30%,IFT=23.7ns

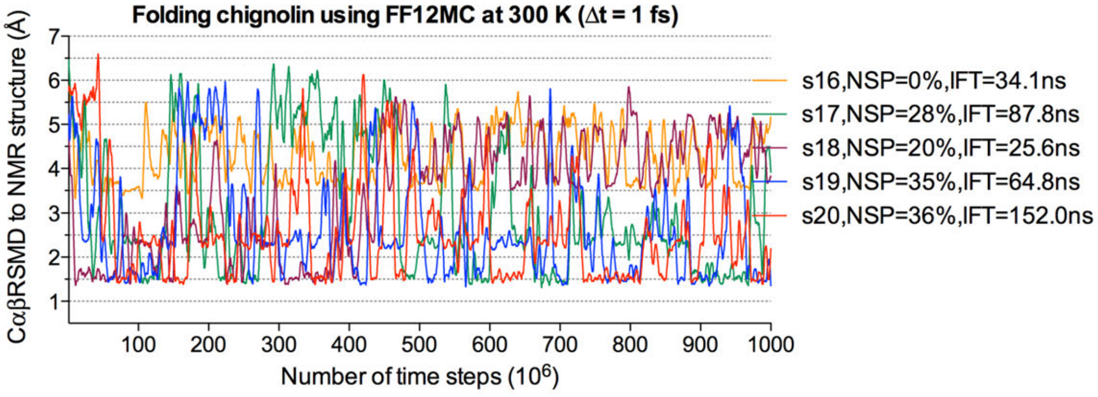
Folding chignolin using FF12MC at 300 K (Δt = 1 fs)
CαβRSMD to NMR structure (Å)
Number of time steps (10^6)
s16,NSP=0%,IFT=34.1ns
s17,NSP=28%,IFT=87.8ns
s18,NSP=20%,IFT=25.6ns
s19,NSP=35%,IFT=64.8ns
s20,NSP=36%,IFT=152.0ns

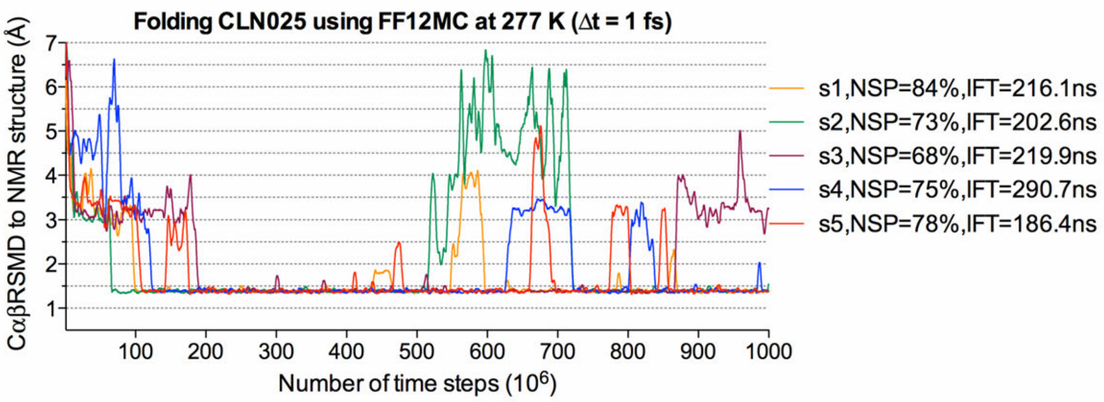
Folding CLN025 using FF12MC at 277 K (Δt = 1 fs)
CαβRSMD to NMR structure (Å)
Number of time steps (10^6)
s1,NSP=84%,IFT=216.1ns
s2,NSP=73%,IFT=202.6ns
s3,NSP=68%,IFT=219.9ns
s4,NSP=75%,IFT=290.7ns
s5,NSP=78%,IFT=186.4ns

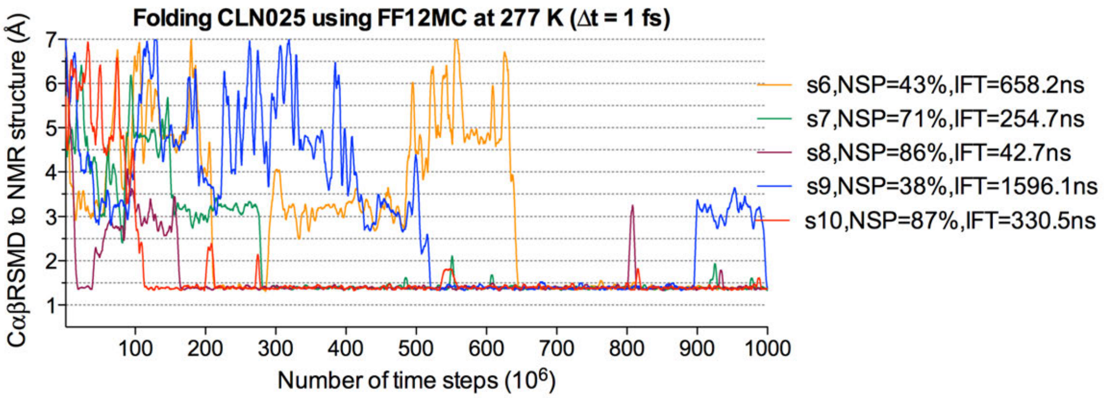
Folding CLN025 using FF12MC at 277 K (Δt = 1 fs)
CαβRSMD to NMR structure (Å)
Number of time steps (10^6)
s6,NSP=43%,IFT=658.2ns
s7,NSP=71%,IFT=254.7ns
s8,NSP=86%,IFT=42.7ns
s9,NSP=38%,IFT=1596.1ns
s10,NSP=87%,IFT=330.5ns

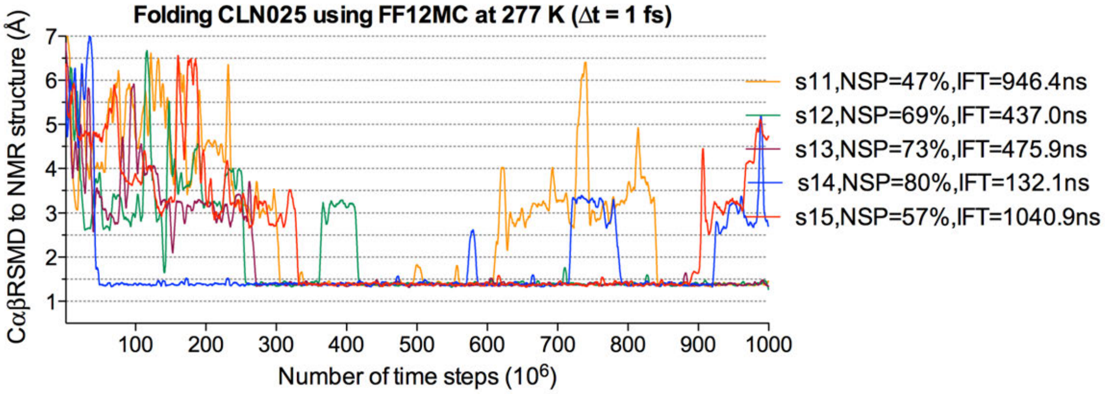
Folding CLN025 using FF12MC at 277 K (Δt = 1 fs)
CαβRSMD to NMR structure (Å)
Number of time steps (10^6)
s11,NSP=47%,IFT=946.4ns
s12,NSP=69%,IFT=437.0ns
s13,NSP=73%,IFT=475.9ns
s14,NSP=80%,IFT=132.1ns
s15,NSP=57%,IFT=1040.9ns

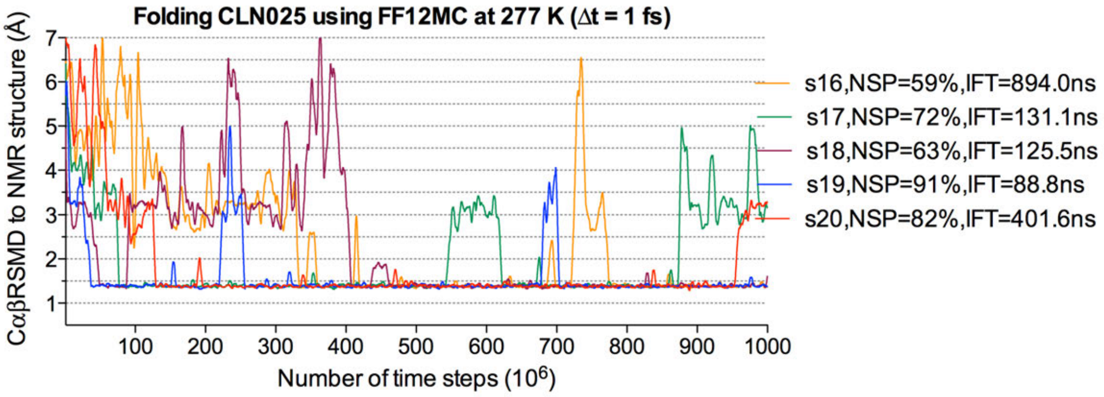
Folding CLN025 using FF12MC at 277 K (Δt = 1 fs)
CαβRSMD to NMR structure (Å)
Number of time steps (10^6)
s16,NSP=59%,IFT=894.0ns
s17,NSP=72%,IFT=131.1ns
s18,NSP=63%,IFT=125.5ns
s19,NSP=91%,IFT=88.8ns
s20,NSP=82%,IFT=401.6ns

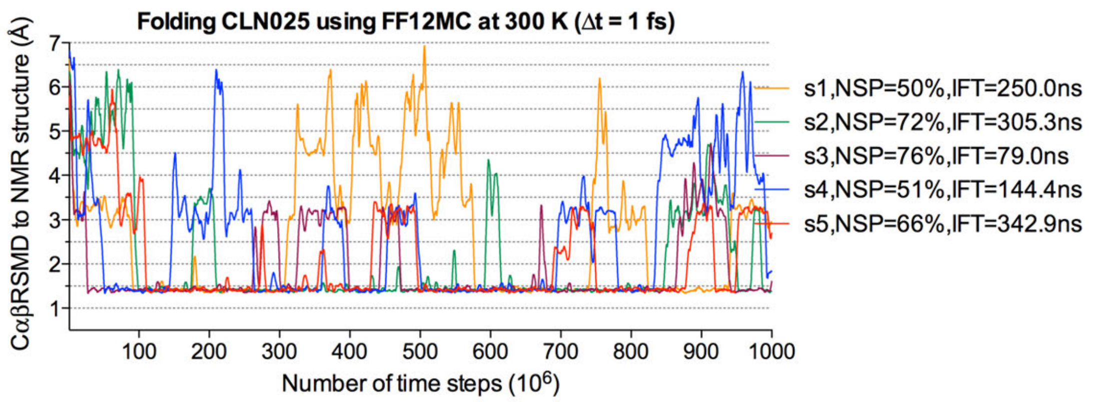

Folding CLN025 using FF12MC at 300 K (Δt = 1 fs)
CαβRSMD to NMR structure (Å)
Number of time steps (10^6)
s1,NSP=50%,IFT=250.0ns
s2,NSP=72%,IFT=305.3ns
s3,NSP=76%,IFT=79.0ns
s4,NSP=51%,IFT=144.4ns
s5,NSP=66%,IFT=342.9ns

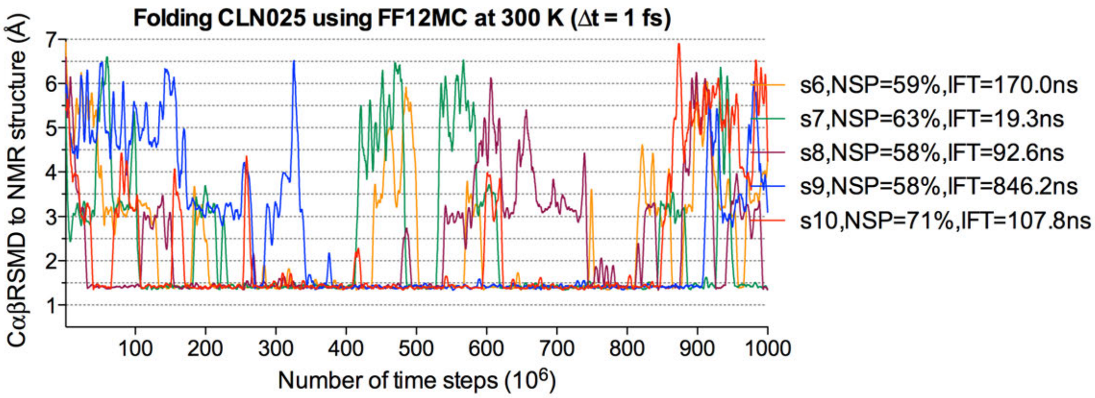

Folding CLN025 using FF12MC at 300 K (Δt = 1 fs)
CαβRSMD to NMR structure (Å)
Number of time steps (10^6)
s6,NSP=59%,IFT=170.0ns
s7,NSP=63%,IFT=19.3ns
s8,NSP=58%,IFT=92.6ns
s9,NSP=58%,IFT=846.2ns
s10,NSP=71%,IFT=107.8ns

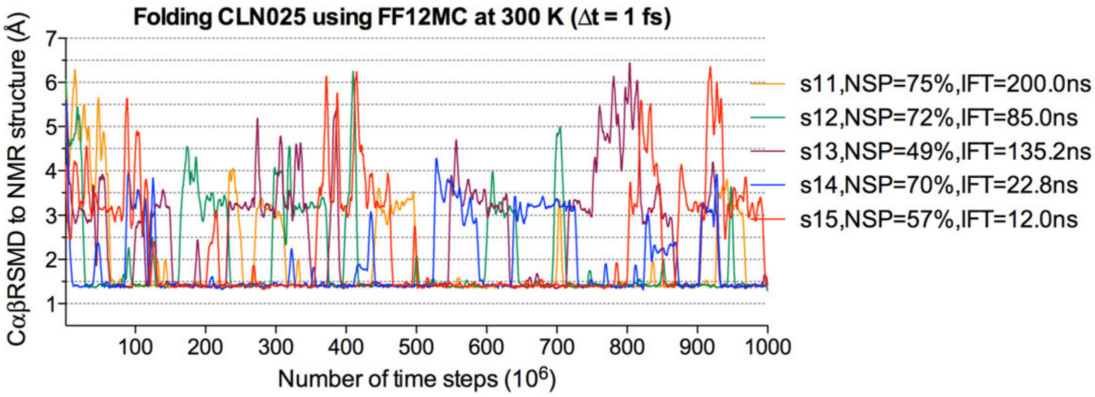

Folding CLN025 using FF12MC at 300 K (Δt = 1 fs)
CαβRSMD to NMR structure (Å)
Number of time steps (10^6)
s11,NSP=75%,IFT=200.0ns
s12,NSP=72%,IFT=85.0ns
s13,NSP=49%,IFT=135.2ns
s14,NSP=70%,IFT=22.8ns
s15,NSP=57%,IFT=12.0ns

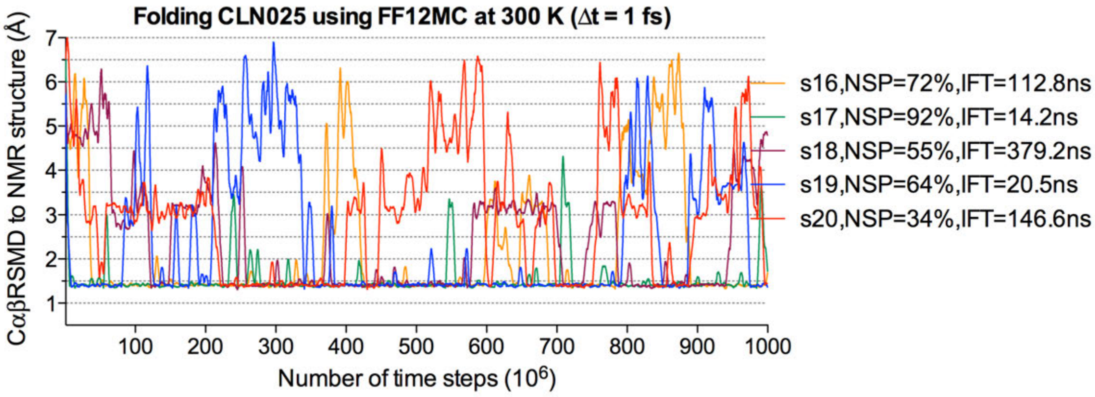

Folding CLN025 using FF12MC at 300 K (Δt = 1 fs)
CαβRSMD to NMR structure (Å)
Number of time steps (10^6)
s16,NSP=72%,IFT=112.8ns
s17,NSP=92%,IFT=14.2ns
s18,NSP=55%,IFT=379.2ns
s19,NSP=64%,IFT=20.5ns
s20,NSP=34%,IFT=146.6ns

Fig. S1*E*

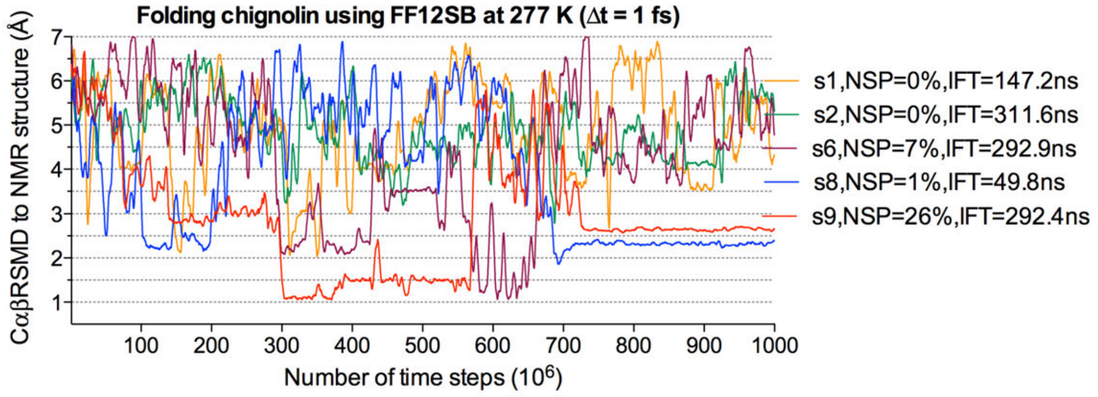
Folding chignolin using FF12SB at 277 K (Δt = 1 fs)
CαβRSMD to NMR structure (Å)
Number of time steps (10^6)
s1,NSP=0%,IFT=147.2ns
s2,NSP=0%,IFT=311.6ns
s6,NSP=7%,IFT=292.9ns
s8,NSP=1%,IFT=49.8ns
s9,NSP=26%,IFT=292.4ns

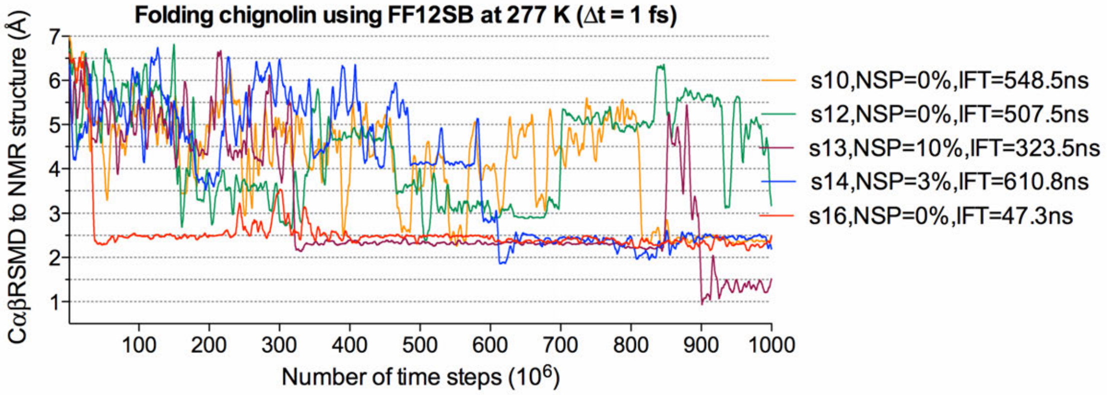
Folding chignolin using FF12SB at 277 K (Δt = 1 fs)
CαβRSMD to NMR structure (Å)
Number of time steps (10^6)
s10,NSP=0%,IFT=548.5ns
s12,NSP=0%,IFT=507.5ns
s13,NSP=10%,IFT=323.5ns
s14,NSP=3%,IFT=610.8ns
s16,NSP=0%,IFT=47.3ns

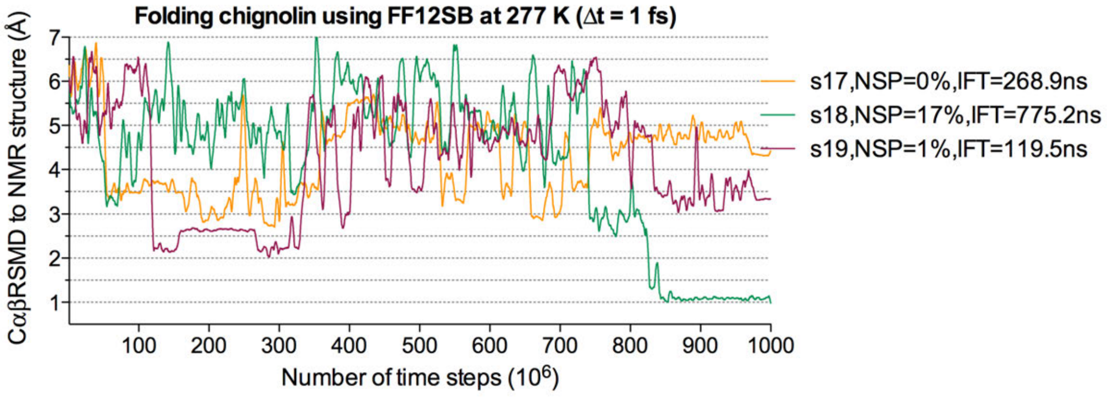
Folding chignolin using FF12SB at 277 K (Δt = 1 fs)
CαβRSMD to NMR structure (Å)
Number of time steps (10^6)
s17,NSP=0%,IFT=268.9ns
s18,NSP=17%,IFT=775.2ns
s19,NSP=1%,IFT=119.5ns

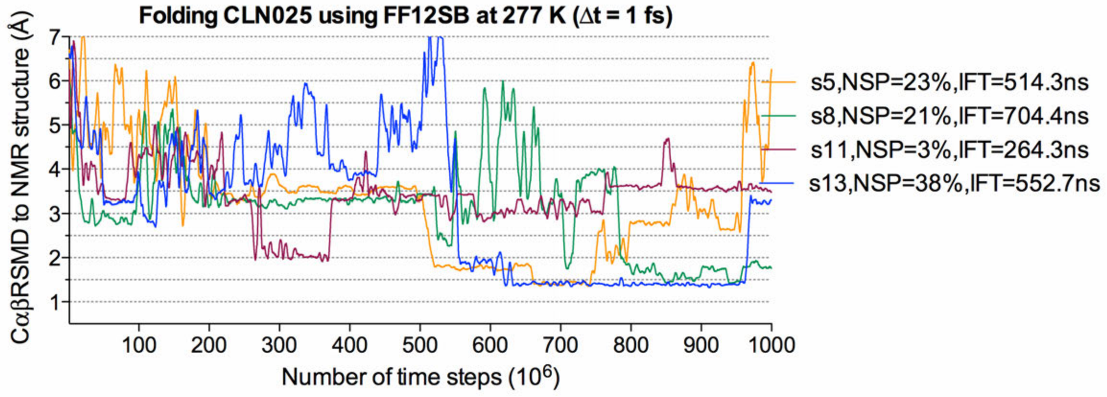
Folding CLN025 using FF12SB at 277 K (Δt = 1 fs)
CαβRSMD to NMR structure (Å)
Number of time steps (10^6)
s5,NSP=23%,IFT=514.3ns
s8,NSP=21%,IFT=704.4ns
s11,NSP=3%,IFT=264.3ns
s13,NSP=38%,IFT=552.7ns

Fig. S1*F*

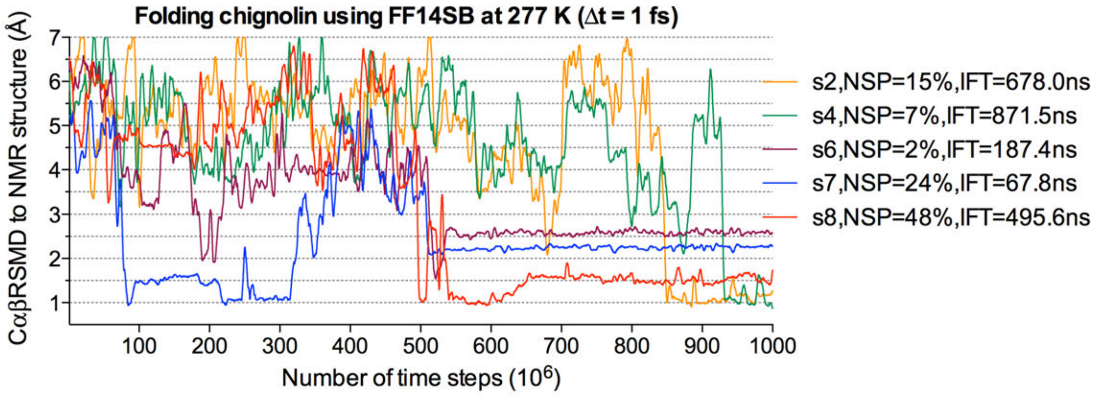
Folding chignolin using FF14SB at 277 K (Δt = 1 fs)
CαβRSMD to NMR structure (Å)
Number of time steps (10^6)
s2,NSP=15%,IFT=678.0ns
s4,NSP=7%,IFT=871.5ns
s6,NSP=2%,IFT=187.4ns
s7,NSP=24%,IFT=67.8ns
s8,NSP=48%,IFT=495.6ns

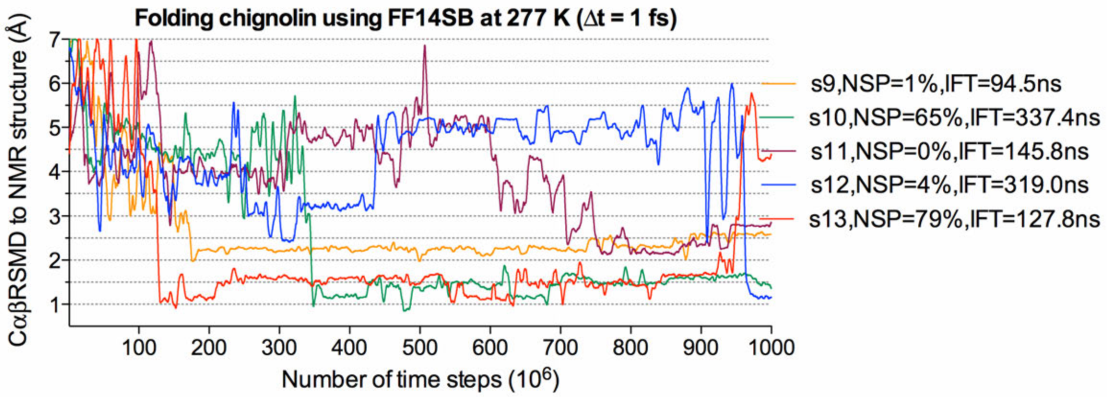
Folding chignolin using FF14SB at 277 K (Δt = 1 fs)
CαβRSMD to NMR structure (Å)
Number of time steps (10^6)
s9,NSP=1%,IFT=94.5ns
s10,NSP=65%,IFT=337.4ns
s11,NSP=0%,IFT=145.8ns
s12,NSP=4%,IFT=319.0ns
s13,NSP=79%,IFT=127.8ns

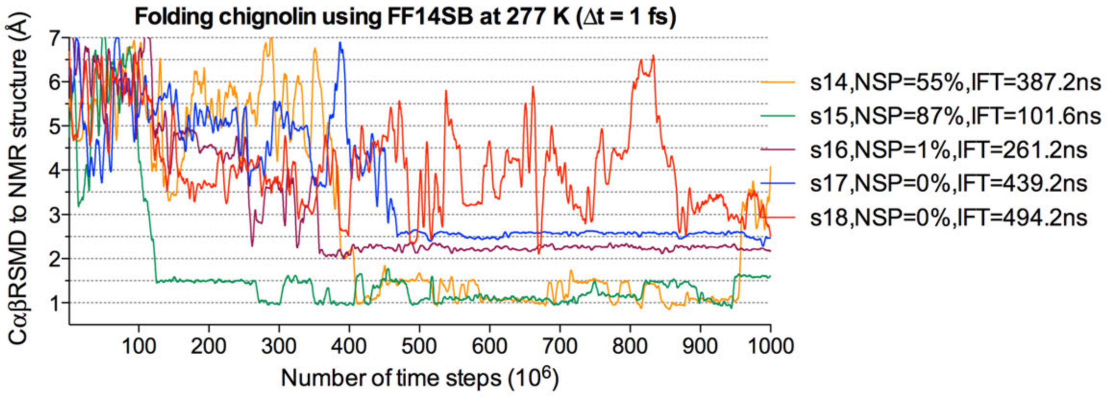
Folding chignolin using FF14SB at 277 K (Δt = 1 fs)
CαβRSMD to NMR structure (Å)
Number of time steps (10^6)
s14,NSP=55%,IFT=387.2ns
s15,NSP=87%,IFT=101.6ns
s16,NSP=1%,IFT=261.2ns
s17,NSP=0%,IFT=439.2ns
s18,NSP=0%,IFT=494.2ns

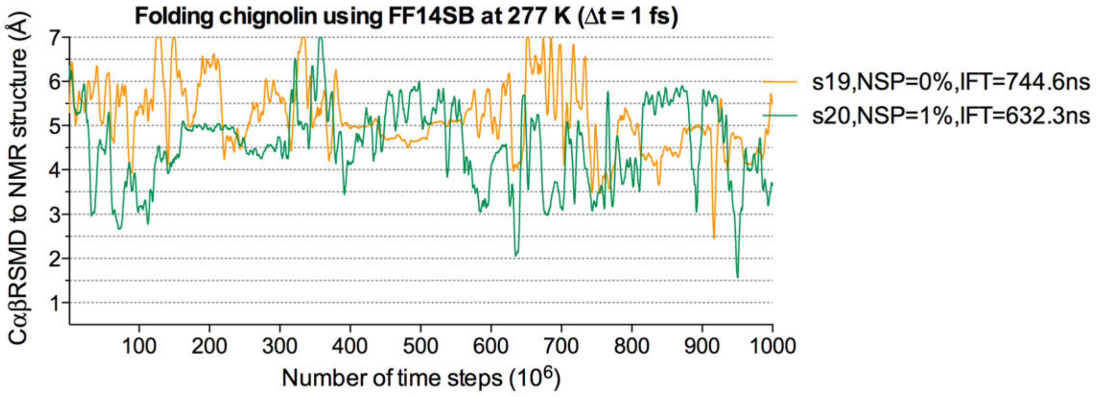
Folding chignolin using FF14SB at 277 K (Δt = 1 fs)
CαβRSMD to NMR structure (Å)
Number of time steps (10^6)
s19,NSP=0%,IFT=744.6ns
s20,NSP=1%,IFT=632.3ns

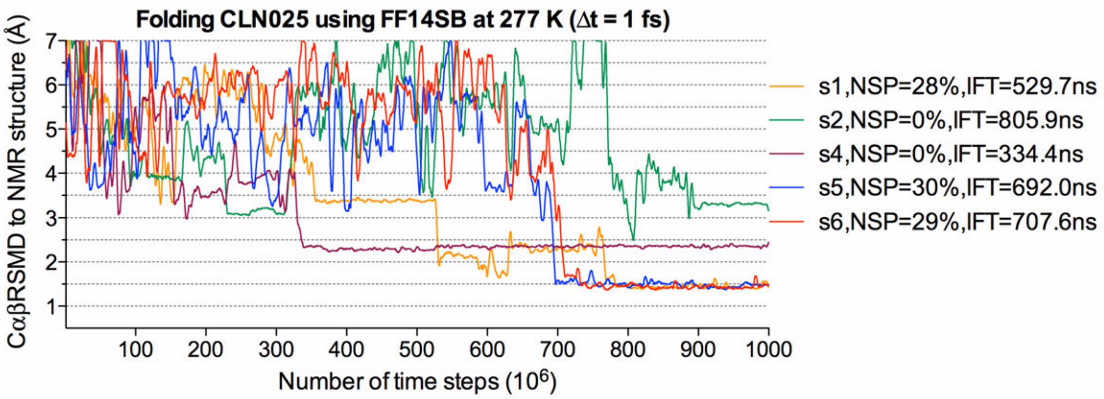
Folding CLN025 using FF14SB at 277 K (Δt = 1 fs)
CαβRSMD to NMR structure (Å)
Number of time steps (10^6)
s1,NSP=28%,IFT=529.7ns
s2,NSP=0%,IFT=805.9ns
s4,NSP=0%,IFT=334.4ns
s5,NSP=30%,IFT=692.0ns
s6,NSP=29%,IFT=707.6ns

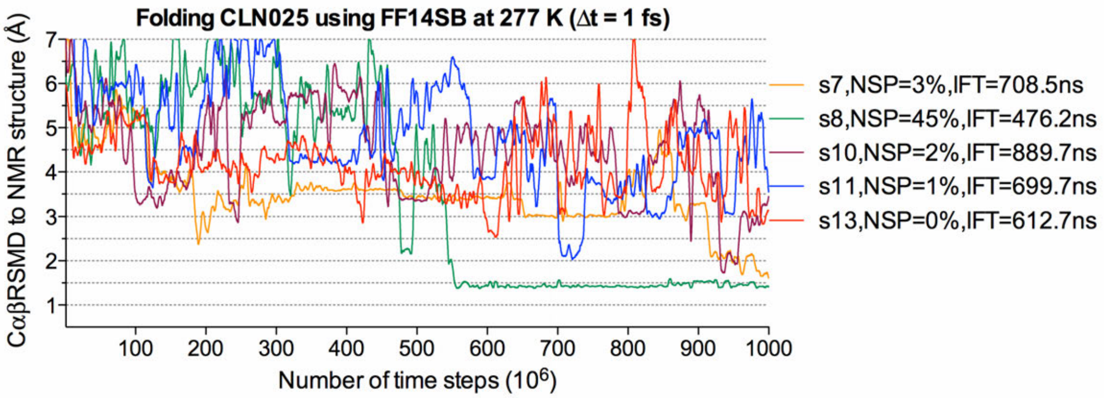
Folding CLN025 using FF14SB at 277 K (Δt = 1 fs)
CαβRSMD to NMR structure (Å)
Number of time steps (10^6)
s7,NSP=3%,IFT=708.5ns
s8,NSP=45%,IFT=476.2ns
s10,NSP=2%,IFT=889.7ns
s11,NSP=1%,IFT=699.7ns
s13,NSP=0%,IFT=612.7ns

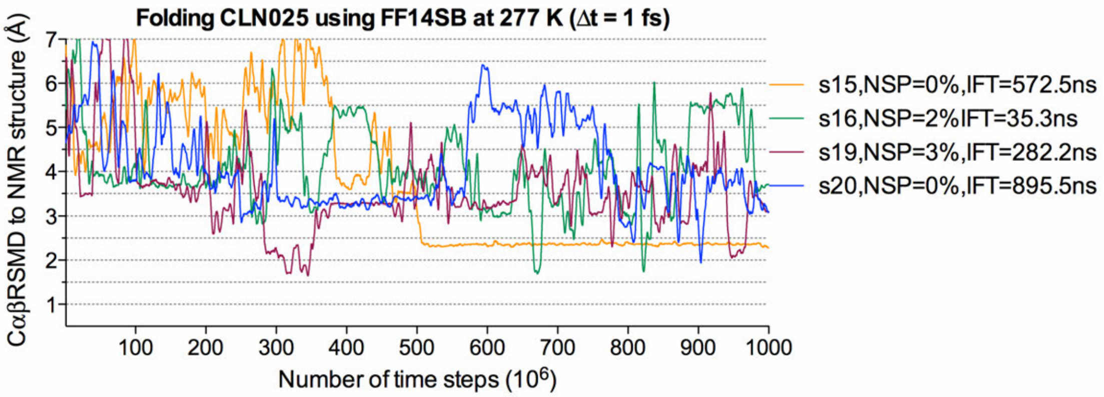
Folding CLN025 using FF14SB at 277 K (Δt = 1 fs)
CαβRSMD to NMR structure (Å)
Number of time steps (10^6)
s15,NSP=0%,IFT=572.5ns
s16,NSP=2%IFT=35.3ns
s19,NSP=3%,IFT=282.2ns
s20,NSP=0%,IFT=895.5ns

Fig. S1G

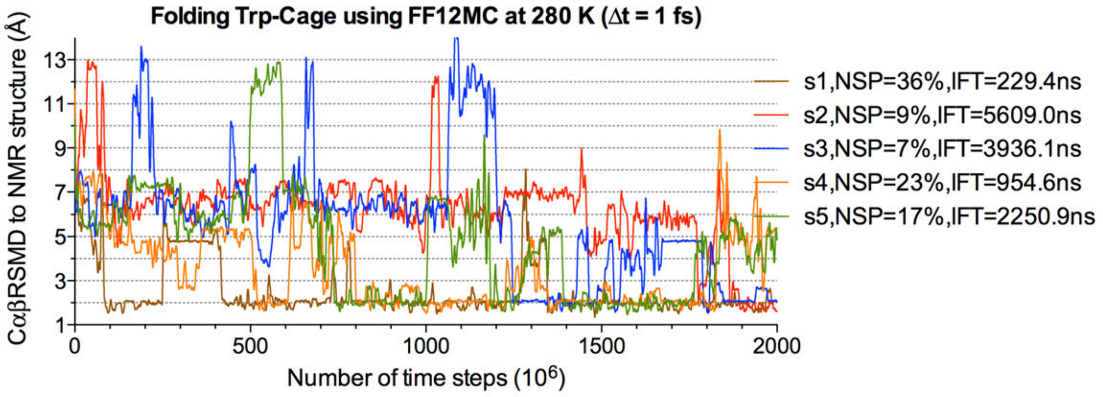
Folding Trp-Cage using FF12MC at 280 K (Δt = 1 fs)
CαβRSMD to NMR structure (Å)
Number of time steps (10^6)
s1,NSP=36%,IFT=229.4ns
s2,NSP=9%,IFT=5609.0ns
s3,NSP=7%,IFT=3936.1ns
s4,NSP=23%,IFT=954.6ns
s5,NSP=17%,IFT=2250.9ns

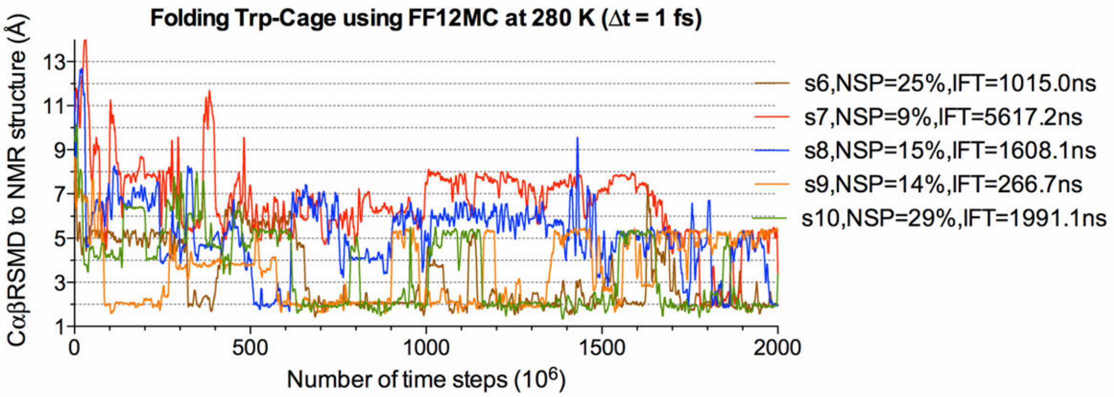
Folding Trp-Cage using FF12MC at 280 K (Δt = 1 fs)
CαβRSMD to NMR structure (Å)
Number of time steps (10^6)
s6,NSP=25%,IFT=1015.0ns
s7,NSP=9%,IFT=5617.2ns
s8,NSP=15%,IFT=1608.1ns
s9,NSP=14%,IFT=266.7ns
s10,NSP=29%,IFT=1991.1ns

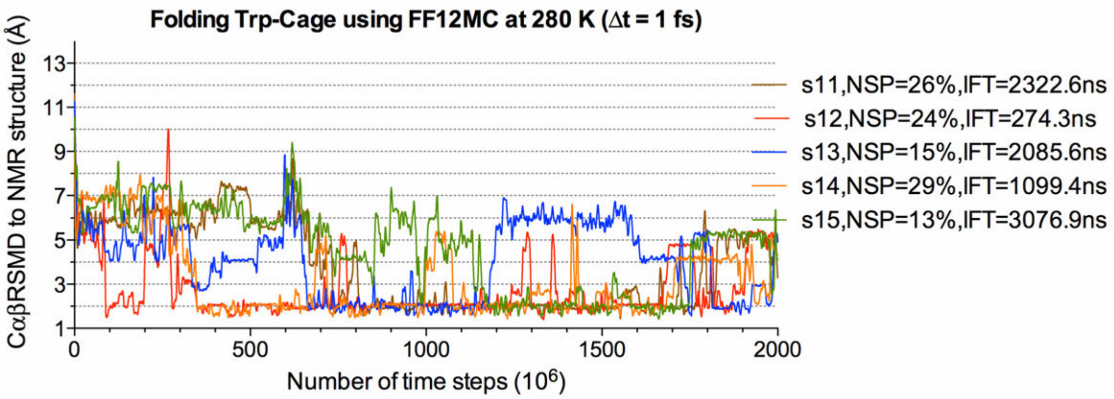
Folding Trp-Cage using FF12MC at 280 K (Δt = 1 fs)
CαβRSMD to NMR structure (Å)
Number of time steps (10^6)
s11,NSP=26%,IFT=2322.6ns
s12,NSP=24%,IFT=274.3ns
s13,NSP=15%,IFT=2085.6ns
s14,NSP=29%,IFT=1099.4ns
s15,NSP=13%,IFT=3076.9ns

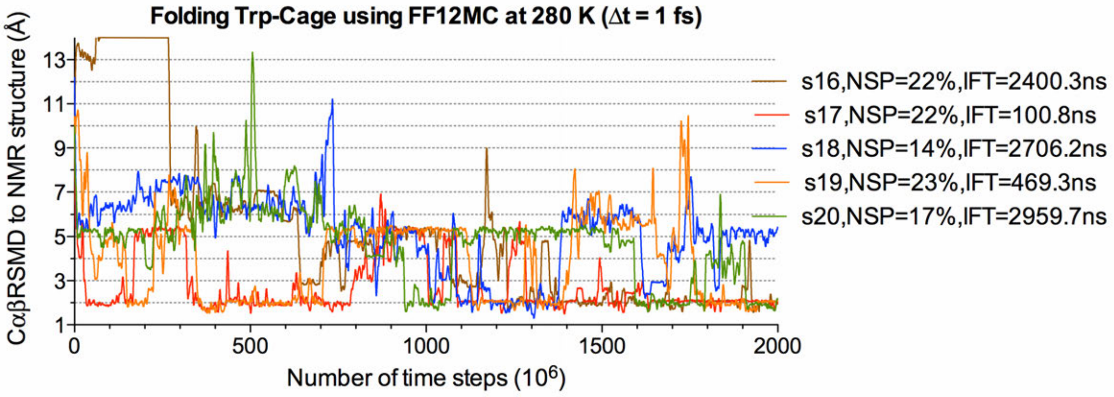
Folding Trp-Cage using FF12MC at 280 K (Δt = 1 fs)
CαβRSMD to NMR structure (Å)
Number of time steps (10^6)
s16,NSP=22%,IFT=2400.3ns
s17,NSP=22%,IFT=100.8ns
s18,NSP=14%,IFT=2706.2ns
s19,NSP=23%,IFT=469.3ns
s20,NSP=17%,IFT=2959.7ns

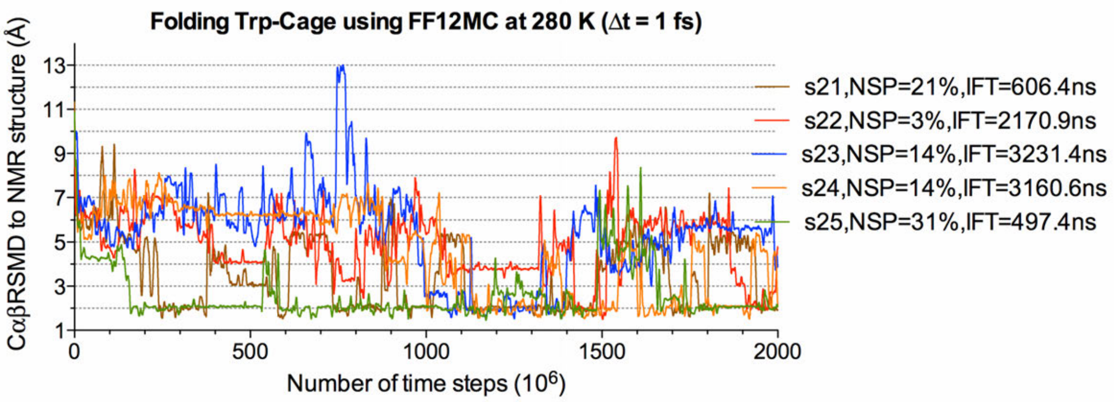
Folding Trp-Cage using FF12MC at 280 K (Δt = 1 fs)
CαβRSMD to NMR structure (Å)
Number of time steps (10^6)
s21,NSP=21%,IFT=606.4ns
s22,NSP=3%,IFT=2170.9ns
s23,NSP=14%,IFT=3231.4ns
s24,NSP=14%,IFT=3160.6ns
s25,NSP=31%,IFT=497.4ns

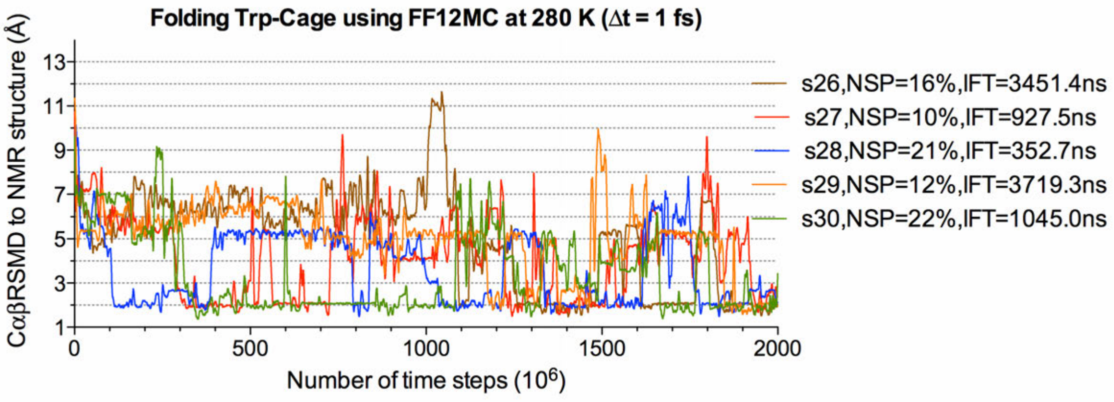
Folding Trp-Cage using FF12MC at 280 K (Δt = 1 fs)
CαβRSMD to NMR structure (Å)
Number of time steps (10^6)
s26,NSP=16%,IFT=3451.4ns
s27,NSP=10%,IFT=927.5ns
s28,NSP=21%,IFT=352.7ns
s29,NSP=12%,IFT=3719.3ns
s30,NSP=22%,IFT=1045.0ns

Fig. S2

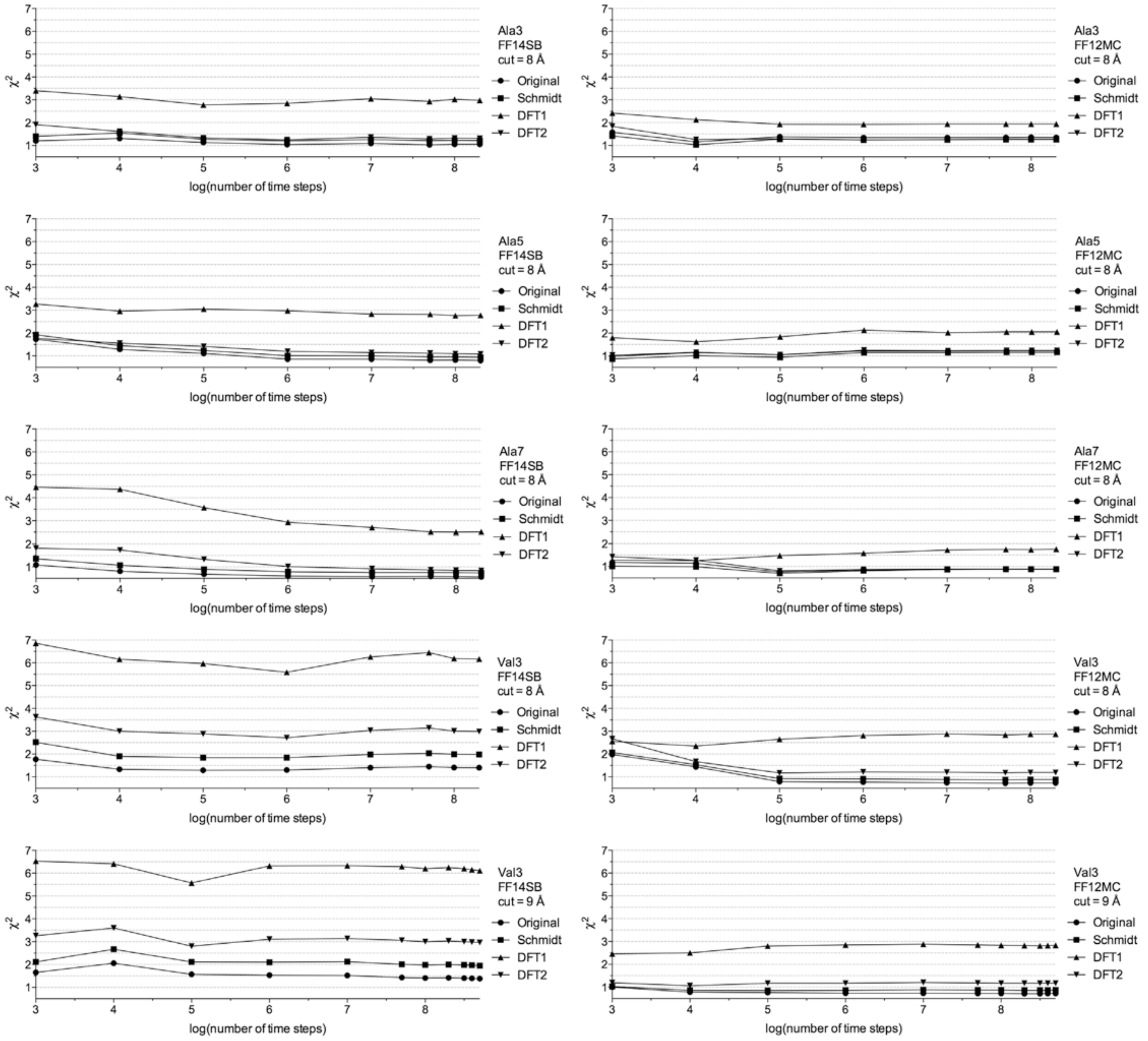
Ala3
FF14SB
cut = 8 Å
Ala3
FF12MC
cut = 8 Å
Ala5
FF14SB
cut = 8 Å
Ala5
FF12MC
cut = 8 Å
Ala7
FF14SB
cut = 8 Å
Ala7
FF12MC
cut = 8 Å
Val3
FF14SB
cut = 8 Å
Val3
FF12MC
cut = 8 Å
Val3
FF14SB
cut = 9 Å
Val3
FF12MC
cut = 9 Å
Original
Schmidt
DFT1
DFT2
χ^2
log(number of time steps)

Fig. S3A

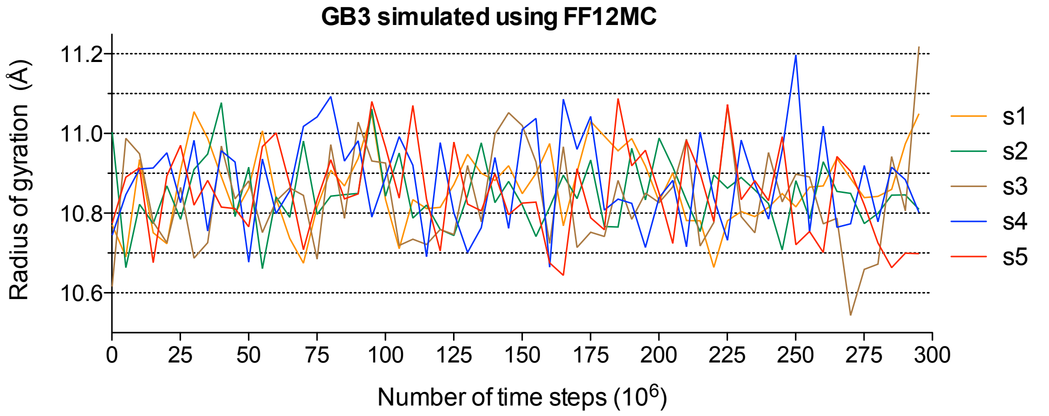
GB3 simulated using FF12MC
Radius of gyration (Å)
11.2
11.0
10.8
10.6
0
25
50
75
100
125
150
175
200
225
250
275
300
Number of time steps (10^6)
s1
s2
s3
s4
s5

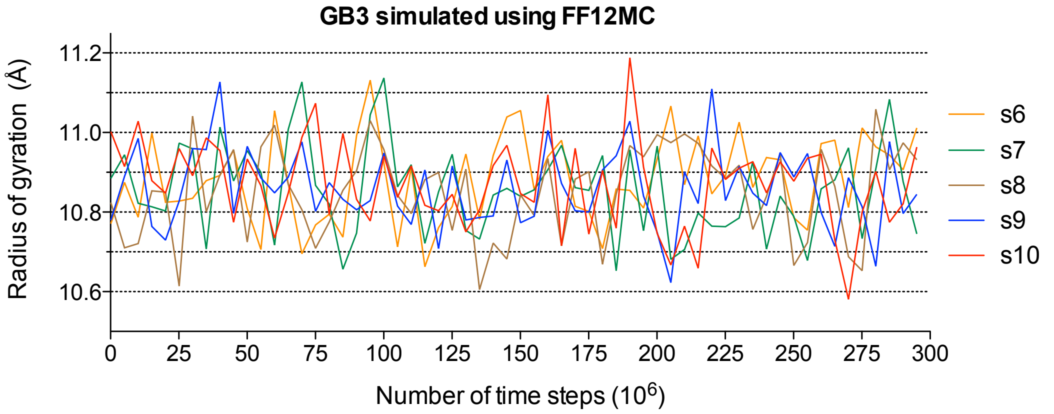
GB3 simulated using FF12MC
Radius of gyration (Å)
11.2
11.0
10.8
10.6
0
25
50
75
100
125
150
175
200
225
250
275
300
Number of time steps (10^6)
s6
s7
s8
s9
s10

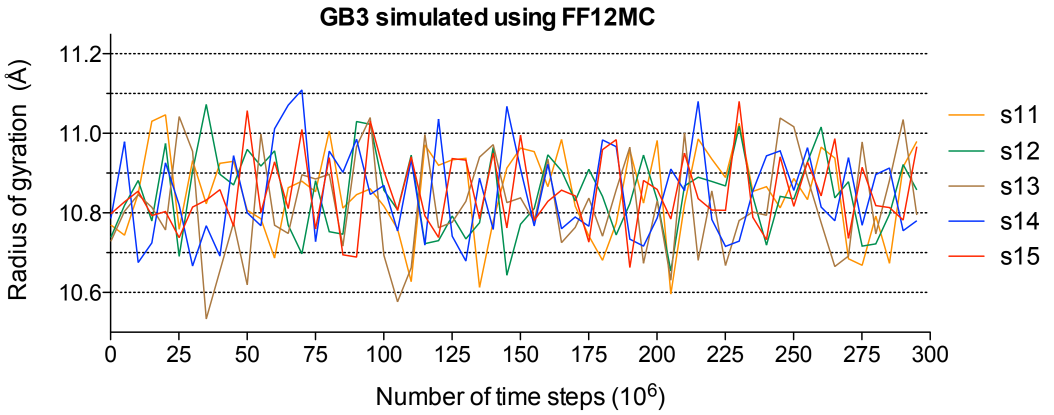
GB3 simulated using FF12MC
Radius of gyration (Å)
11.2
11.0
10.8
10.6
0
25
50
75
100
125
150
175
200
225
250
275
300
Number of time steps (10^6)
s11
s12
s13
s14
s15

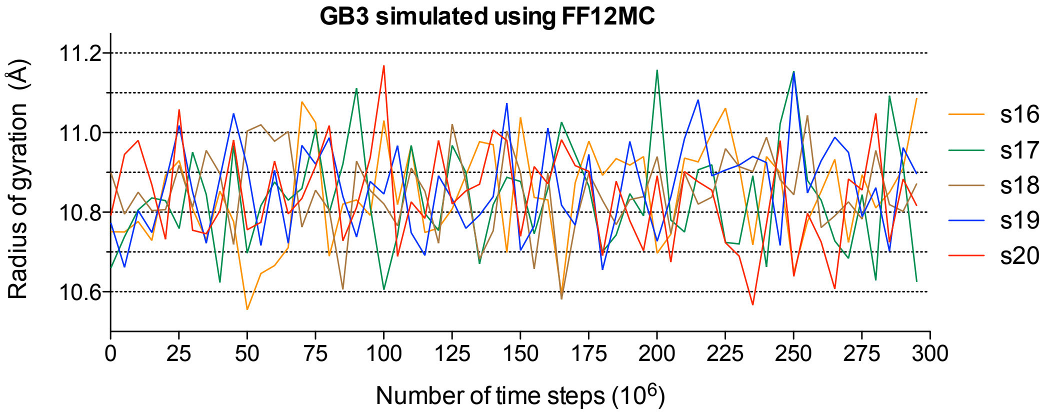
GB3 simulated using FF12MC
Radius of gyration (Å)
11.2
11.0
10.8
10.6
0
25
50
75
100
125
150
175
200
225
250
275
300
Number of time steps (10^6)
s16
s17
s18
s19
s20

Fig. S3B

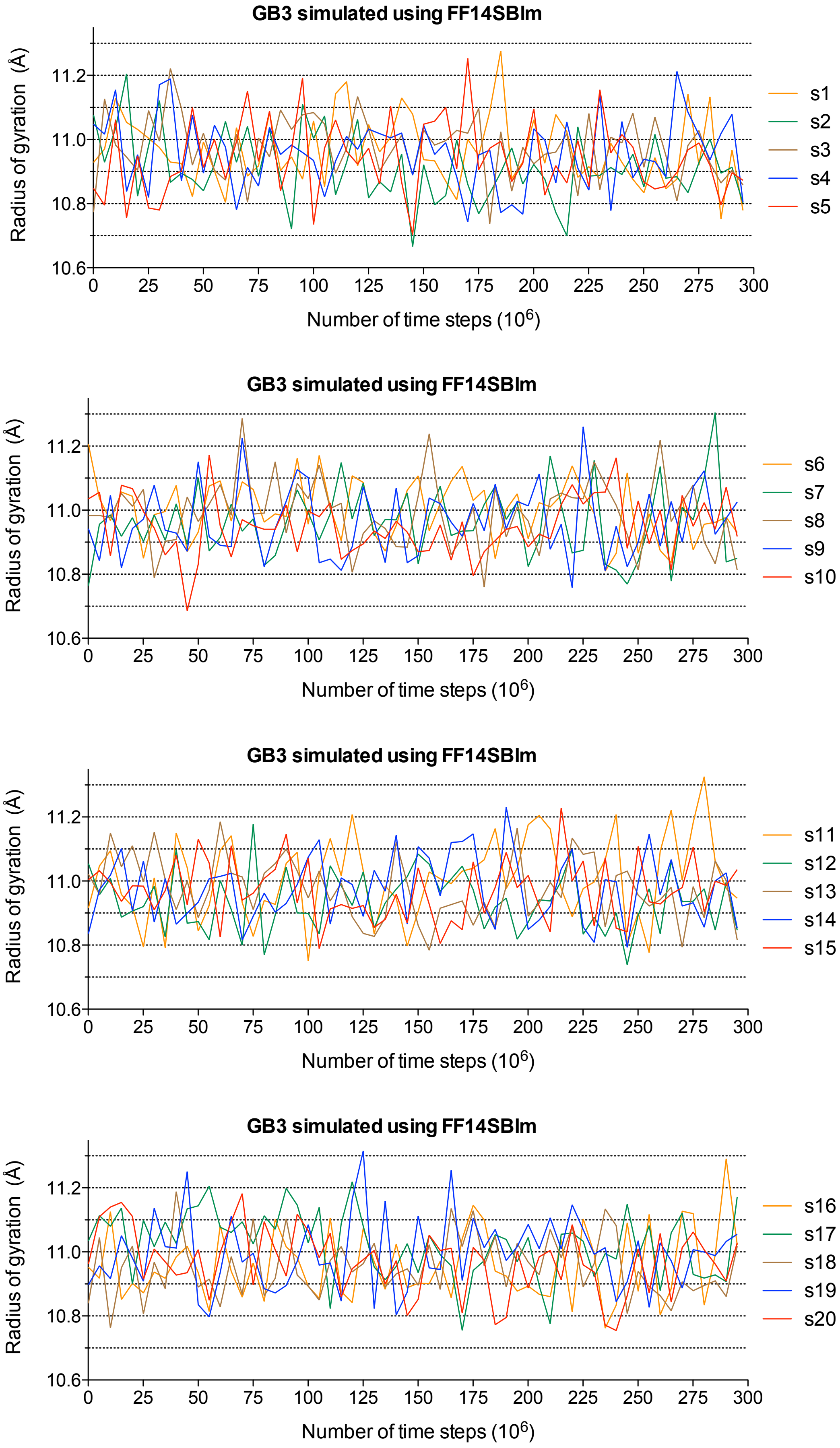
GB3 simulated using FF14SBlm
Radius of gyration (Å)
Number of time steps (10^6)
s1
s2
s3
s4
s5
GB3 simulated using FF14SBlm
s6
s7
s8
s9
s10
GB3 simulated using FF14SBlm
s11
s12
s13
s14
s15
GB3 simulated using FF14SBlm
s16
s17
s18
s19
s20

Fig. S3C

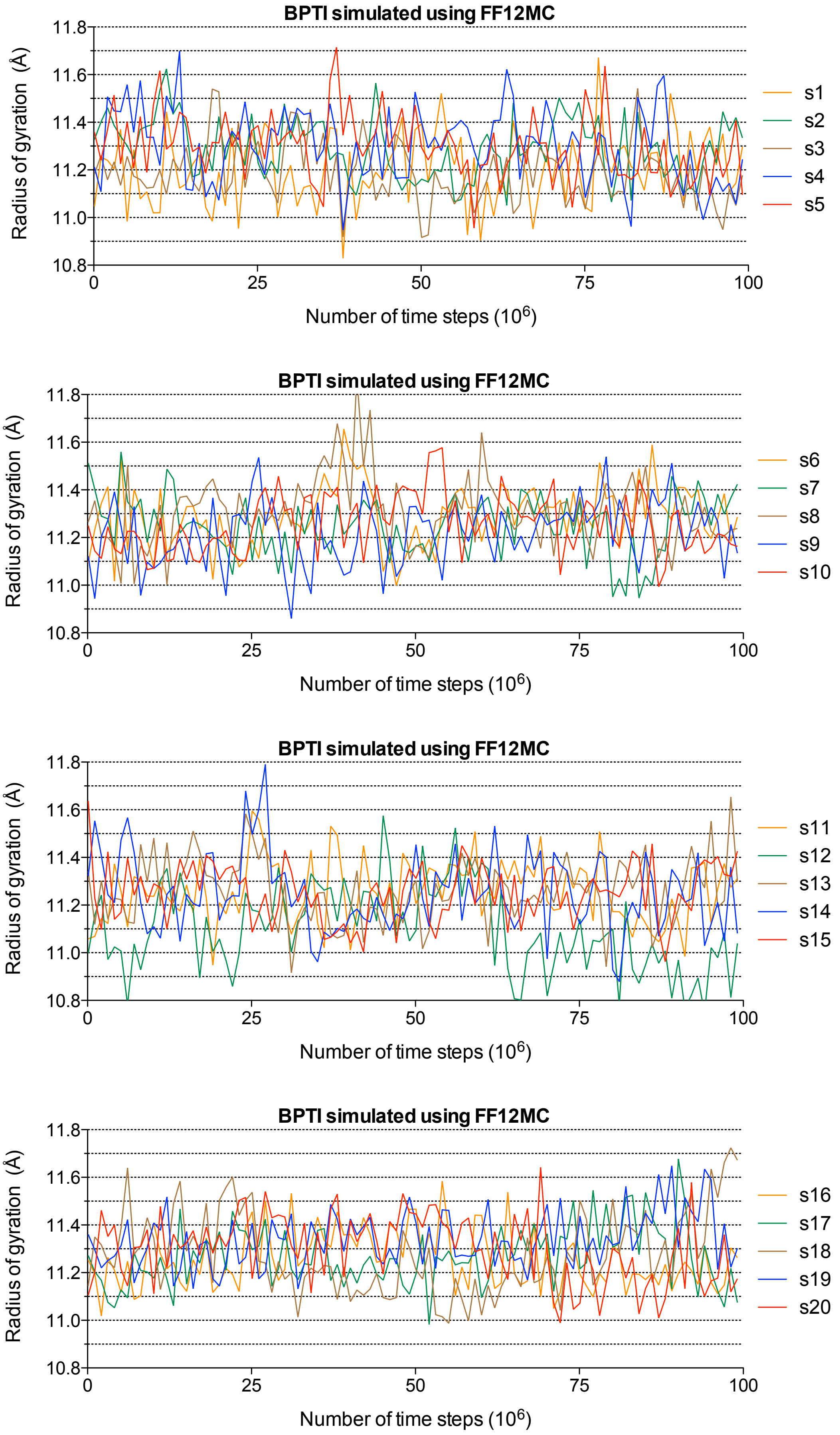
BPTI simulated using FF12MC
Radius of gyration (Å)
Number of time steps (10^6)
s1
s2
s3
s4
s5
BPTI simulated using FF12MC
Radius of gyration (Å)
Number of time steps (10^6)
s6
s7
s8
s9
s10
BPTI simulated using FF12MC
Radius of gyration (Å)
Number of time steps (10^6)
s11
s12
s13
s14
s15
BPTI simulated using FF12MC
Radius of gyration (Å)
Number of time steps (10^6)
s16
s17
s18
s19
s20

Fig. S3D

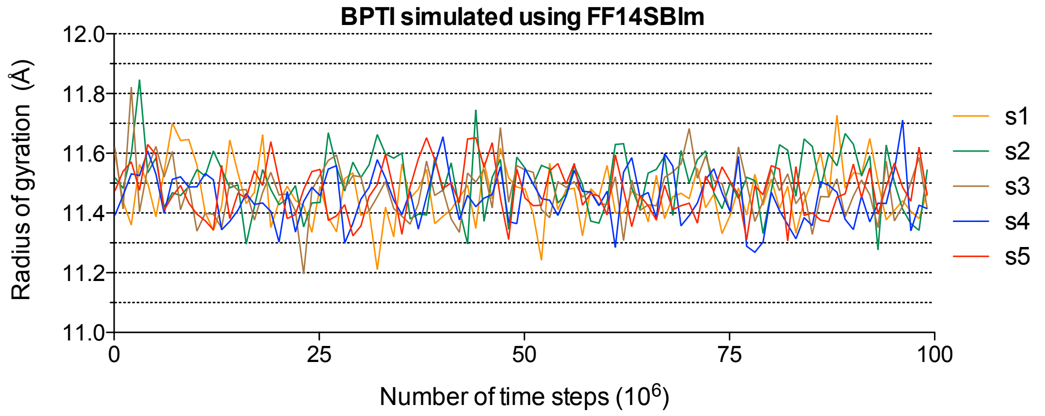
BPTI simulated using FF14SBIm
Radius of gyration (Å)
12.0
11.8
11.6
11.4
11.2
11.0
0
25
50
75
100
Number of time steps (10^6)
s1
s2
s3
s4
s5

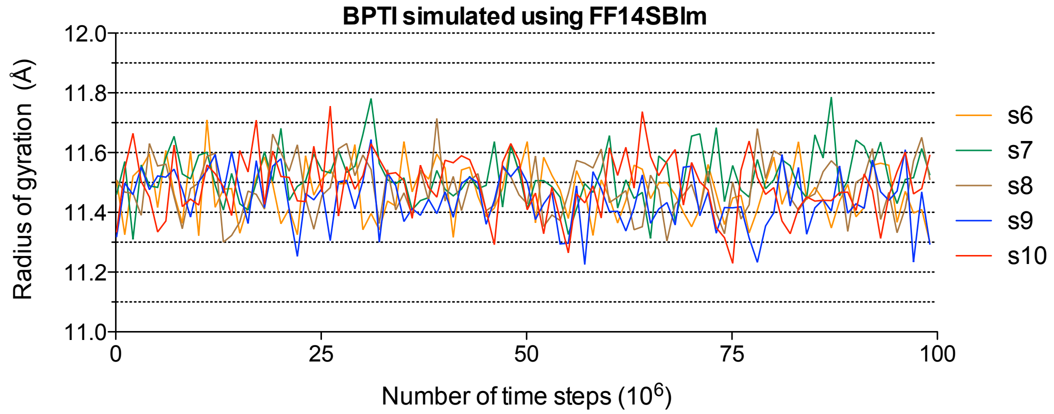
BPTI simulated using FF14SBIm
Radius of gyration (Å)
12.0
11.8
11.6
11.4
11.2
11.0
0
25
50
75
100
Number of time steps (10^6)
s6
s7
s8
s9
s10

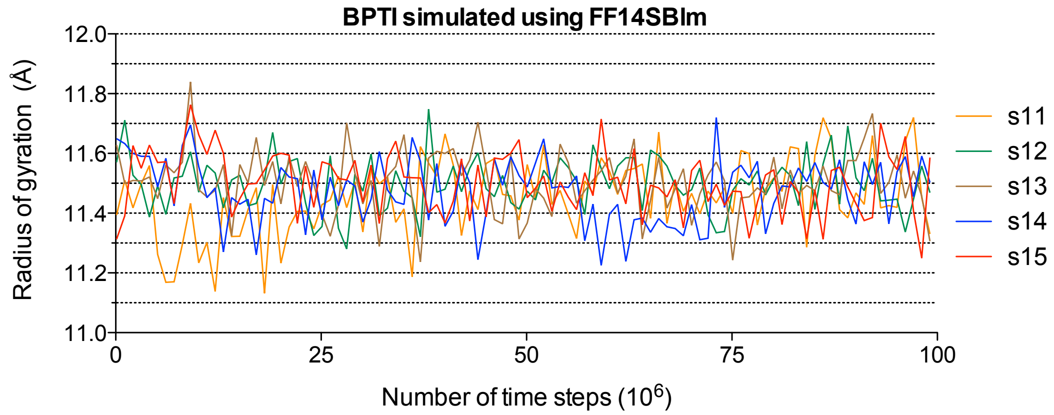
BPTI simulated using FF14SBIm
Radius of gyration (Å)
12.0
11.8
11.6
11.4
11.2
11.0
0
25
50
75
100
Number of time steps (10^6)
s11
s12
s13
s14
s15

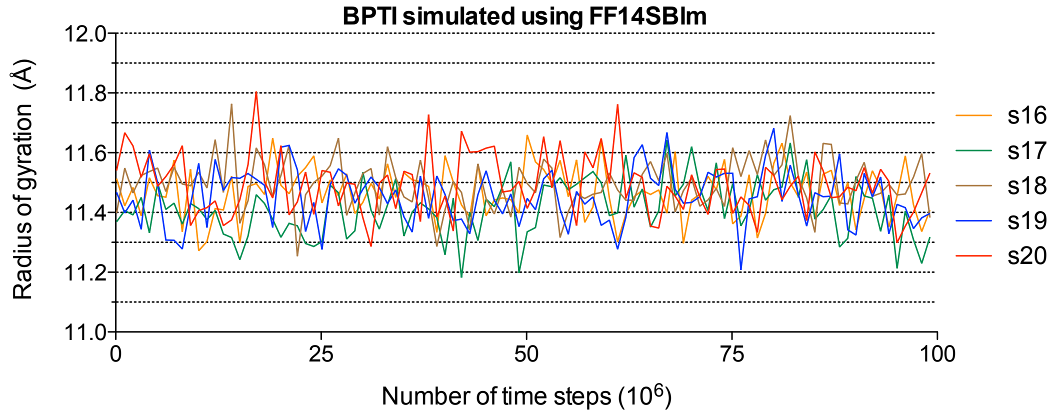
BPTI simulated using FF14SBIm
Radius of gyration (Å)
12.0
11.8
11.6
11.4
11.2
11.0
0
25
50
75
100
Number of time steps (10^6)
s16
s17
s18
s19
s20

Fig. S3E

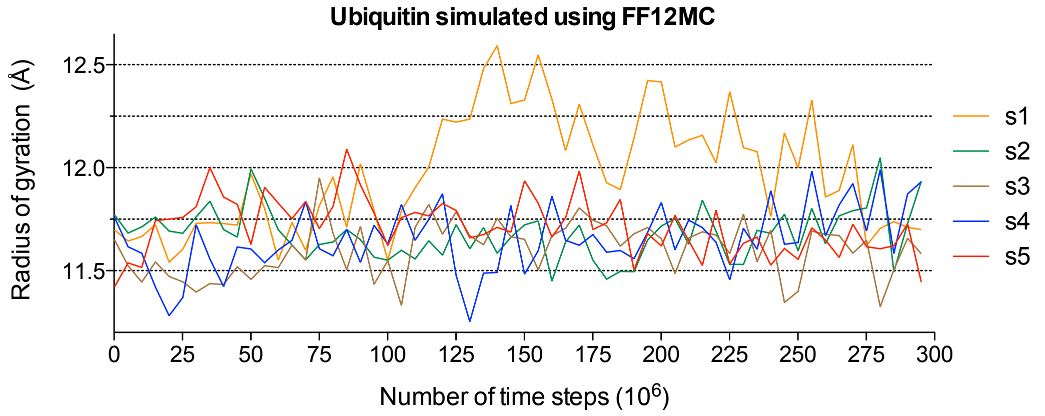
Ubiquitin simulated using FF12MC
Radius of gyration (Å)
12.5
12.0
11.5
0 25 50 75 100 125 150 175 200 225 250 275 300
Number of time steps (10^6)
s1
s2
s3
s4
s5

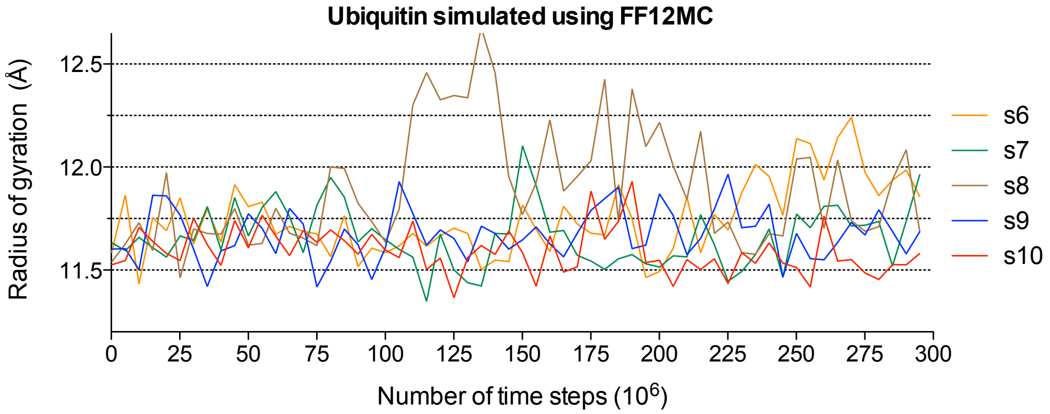
Ubiquitin simulated using FF12MC
Radius of gyration (Å)
12.5
12.0
11.5
0 25 50 75 100 125 150 175 200 225 250 275 300
Number of time steps (10^6)
s6
s7
s8
s9
s10

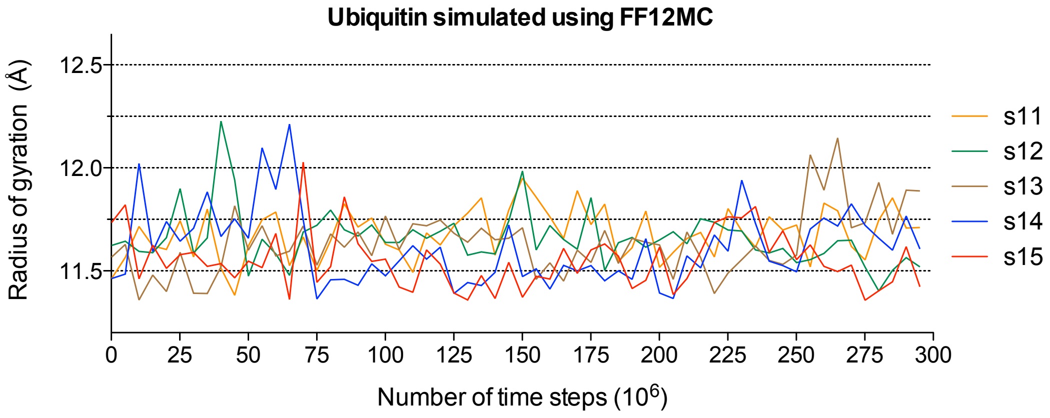
Ubiquitin simulated using FF12MC
Radius of gyration (Å)
12.5
12.0
11.5
0 25 50 75 100 125 150 175 200 225 250 275 300
Number of time steps (10^6)
s11
s12
s13
s14
s15

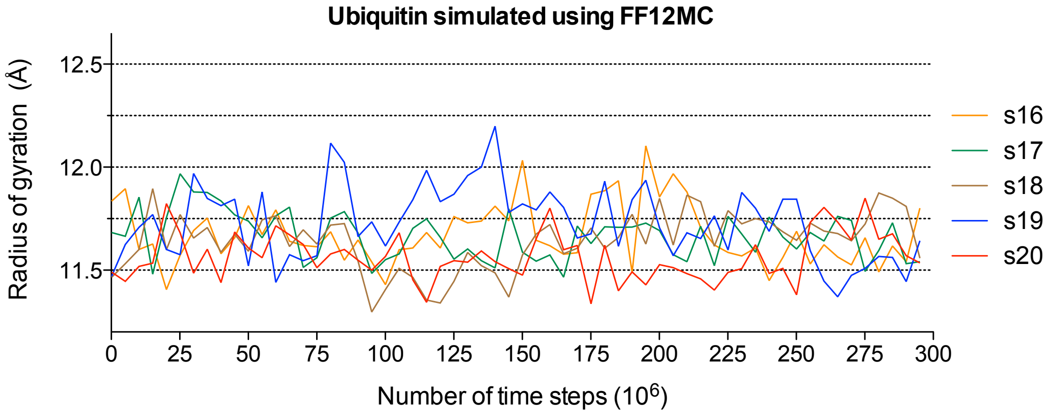
Ubiquitin simulated using FF12MC
Radius of gyration (Å)
12.5
12.0
11.5
0 25 50 75 100 125 150 175 200 225 250 275 300
Number of time steps (10^6)
s16
s17
s18
s19
s20

Fig. S3F

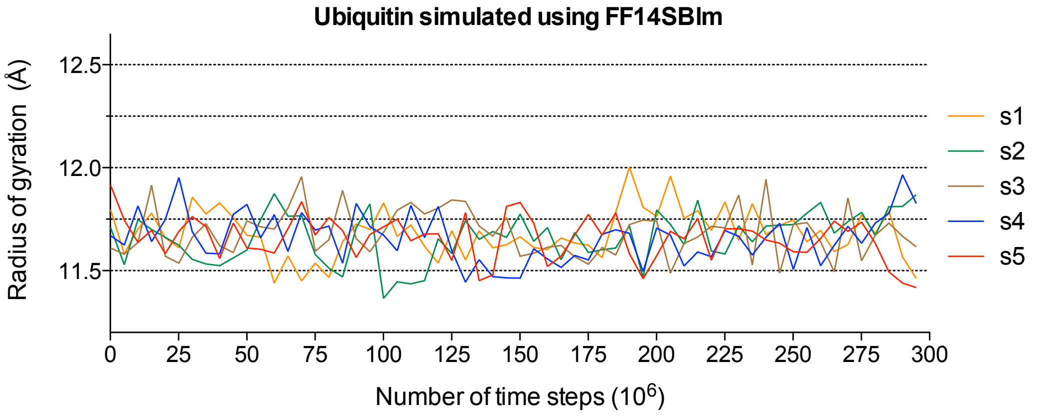

Ubiquitin simulated using FF14SBlm
Radius of gyration (Å)
12.5
12.0
11.5
0
25
50
75
100
125
150
175
200
225
250
275
300
Number of time steps (10^6)
s1
s2
s3
s4
s5

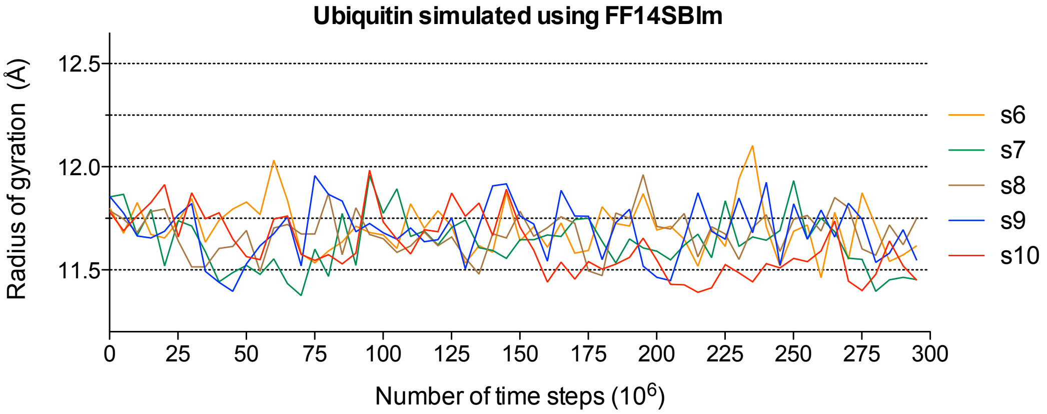

Ubiquitin simulated using FF14SBlm
Radius of gyration (Å)
12.5
12.0
11.5
0
25
50
75
100
125
150
175
200
225
250
275
300
Number of time steps (10^6)
s6
s7
s8
s9
s10

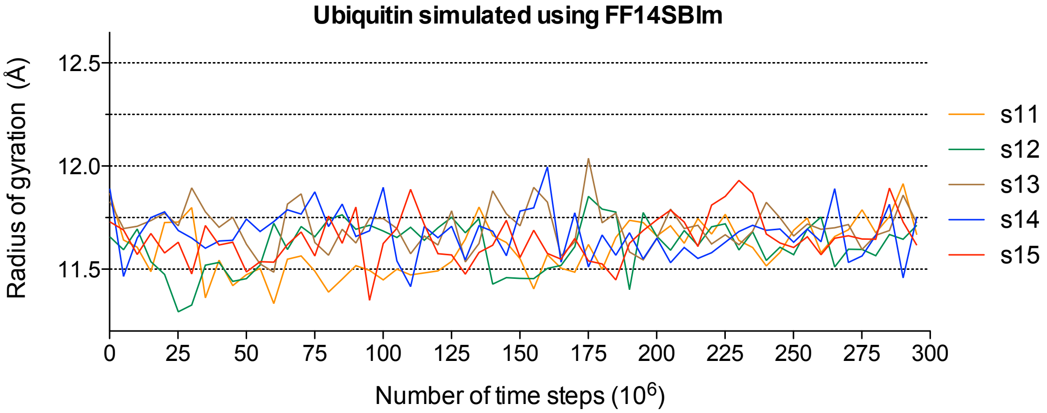

Ubiquitin simulated using FF14SBlm
Radius of gyration (Å)
12.5
12.0
11.5
0
25
50
75
100
125
150
175
200
225
250
275
300
Number of time steps (10^6)
s11
s12
s13
s14
s15

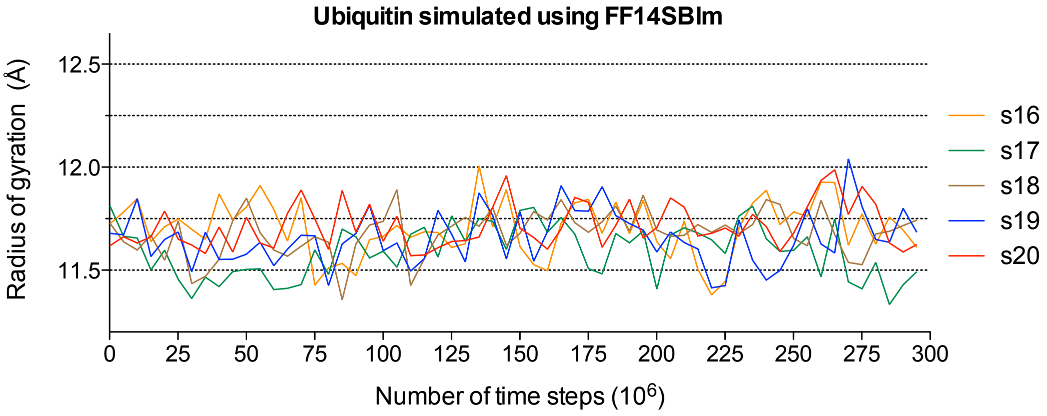

Ubiquitin simulated using FF14SBlm
Radius of gyration (Å)
12.5
12.0
11.5
0
25
50
75
100
125
150
175
200
225
250
275
300
Number of time steps (10^6)
s16
s17
s18
s19
s20

Fig. S3G

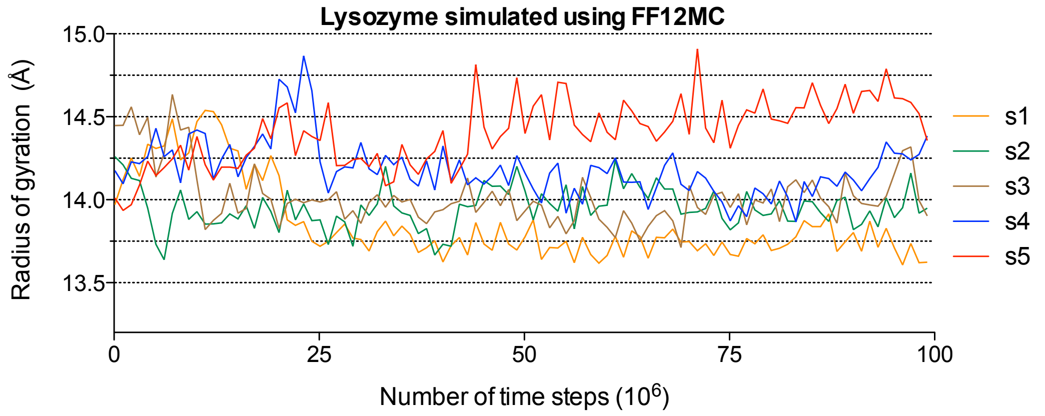
Lysozyme simulated using FF12MC
Radius of gyration (Å)
15.0
14.5
14.0
13.5
0
25
50
75
100
Number of time steps (10^6)
s1
s2
s3
s4
s5

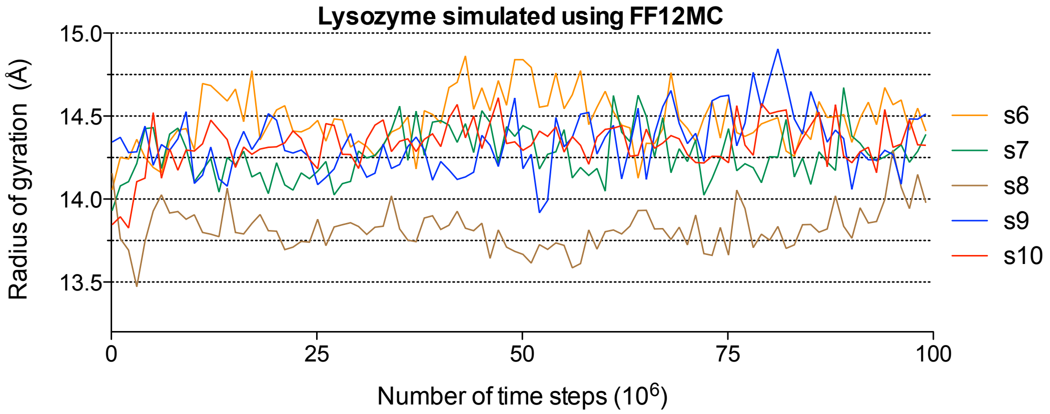
Lysozyme simulated using FF12MC
Radius of gyration (Å)
15.0
14.5
14.0
13.5
0
25
50
75
100
Number of time steps (10^6)
s6
s7
s8
s9
s10

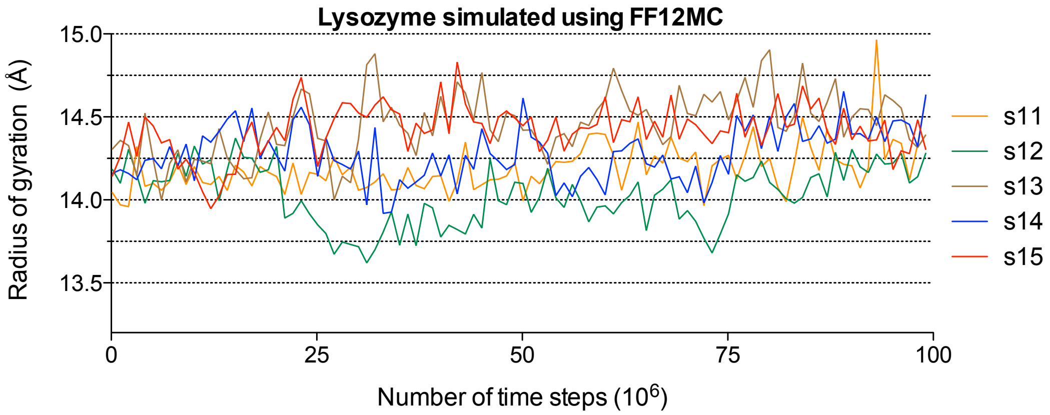
Lysozyme simulated using FF12MC
Radius of gyration (Å)
15.0
14.5
14.0
13.5
0
25
50
75
100
Number of time steps (10^6)
s11
s12
s13
s14
s15

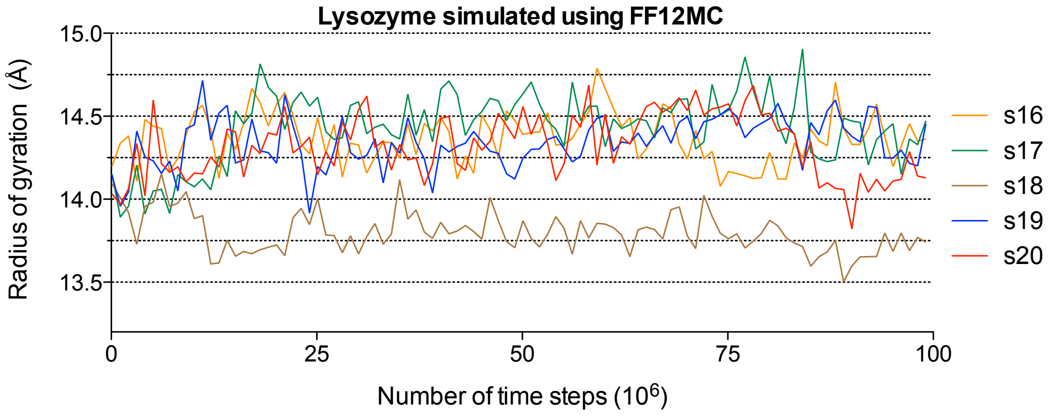
Lysozyme simulated using FF12MC
Radius of gyration (Å)
15.0
14.5
14.0
13.5
0
25
50
75
100
Number of time steps (10^6)
s16
s17
s18
s19
s20

Fig. S3H

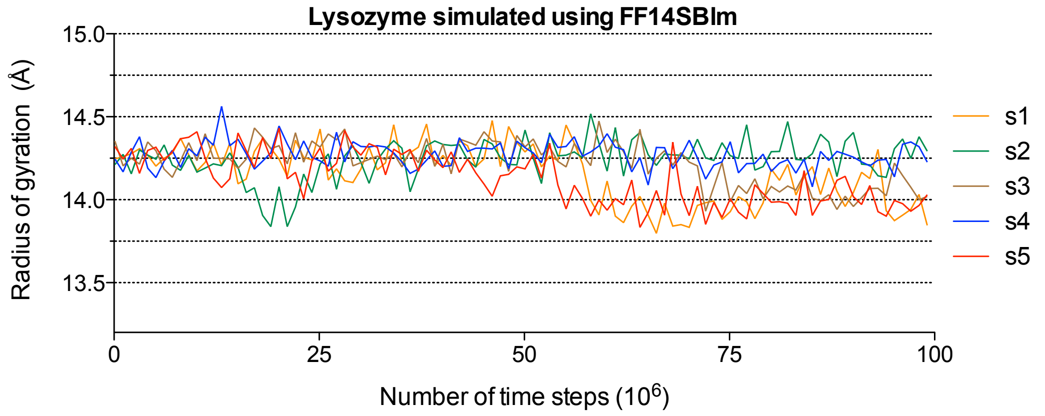
Lysozyme simulated using FF14SBlm
Radius of gyration (Å)
15.0
14.5
14.0
13.5
0
25
50
75
100
Number of time steps (10^6)
s1
s2
s3
s4
s5

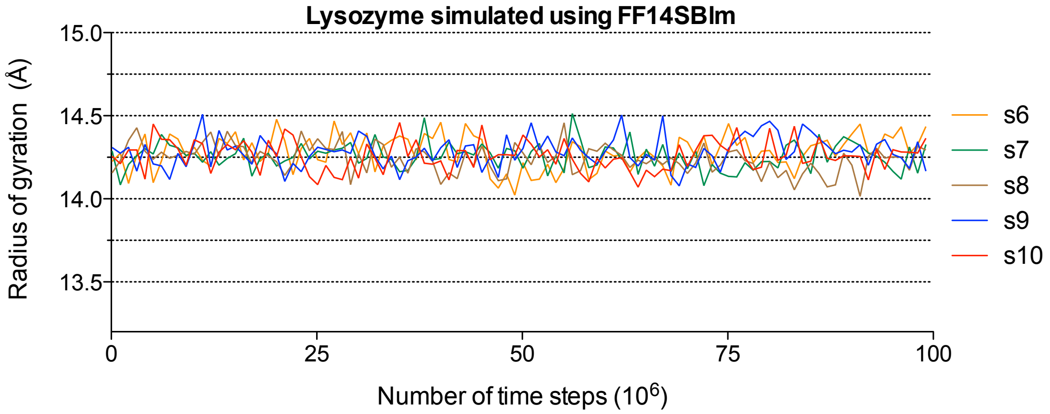
Lysozyme simulated using FF14SBlm
Radius of gyration (Å)
15.0
14.5
14.0
13.5
0
25
50
75
100
Number of time steps (10^6)
s6
s7
s8
s9
s10

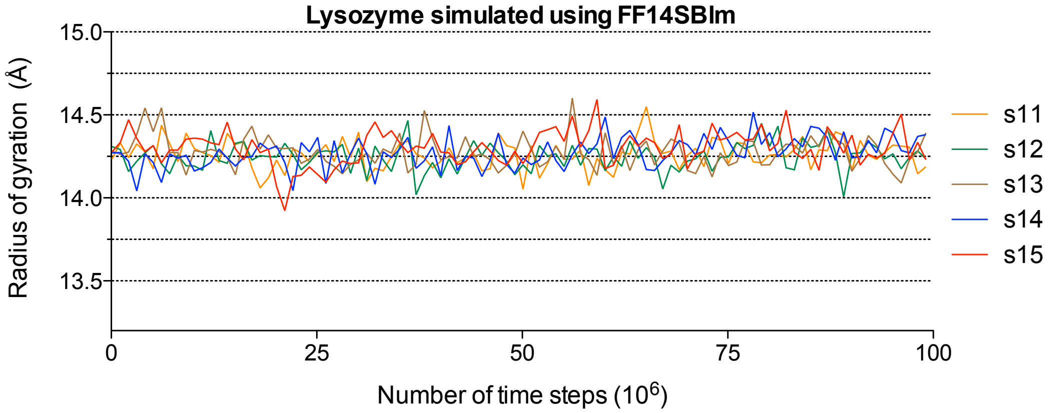
Lysozyme simulated using FF14SBlm
Radius of gyration (Å)
15.0
14.5
14.0
13.5
0
25
50
75
100
Number of time steps (10^6)
s11
s12
s13
s14
s15

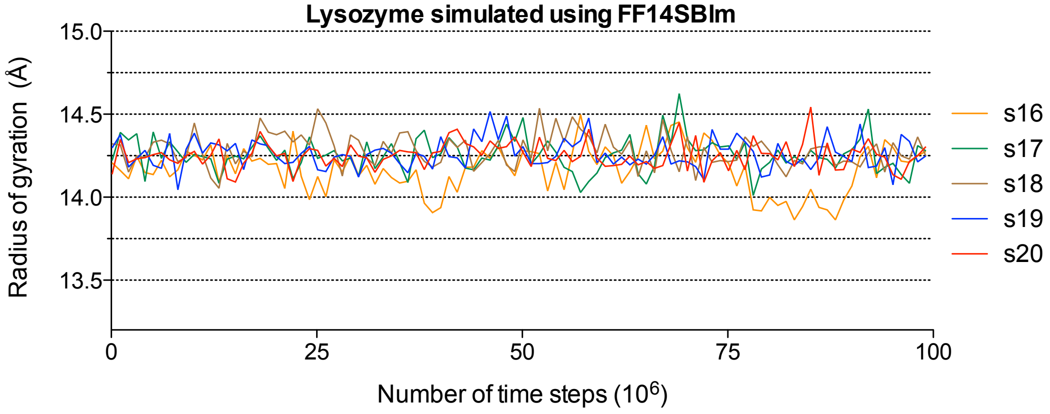
Lysozyme simulated using FF14SBlm
Radius of gyration (Å)
15.0
14.5
14.0
13.5
0
25
50
75
100
Number of time steps (10^6)
s16
s17
s18
s19
s20

Fig. S4

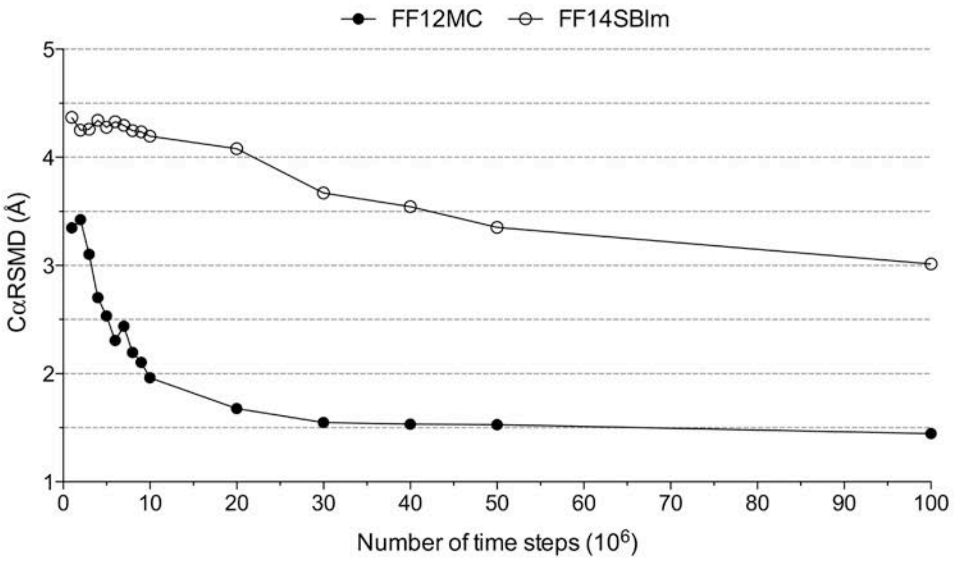
FF12MC
FF14SBlm
CαRSMD (Å)
Number of time steps (10^6)

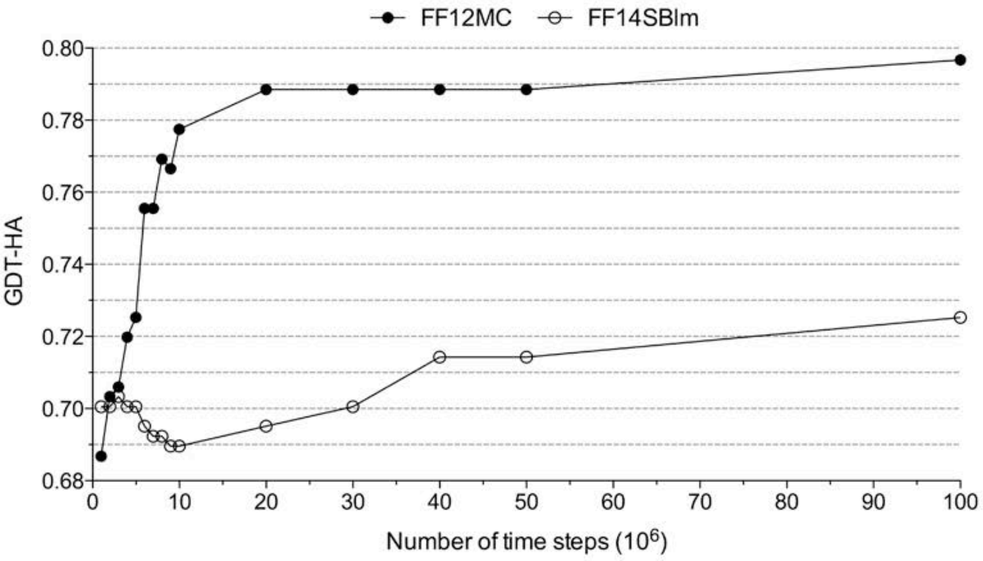
FF12MC
FF14SBlm
GDT-HA
Number of time steps (10^6)
